# The Identification of Blood Biomarkers of Chronic Neuropathic Pain by Comparative Transcriptomics

**DOI:** 10.1007/s12017-021-08694-8

**Published:** 2021-11-05

**Authors:** Barira Islam, John Stephenson, Bethan Young, Maurizio Manca, David A. Buckley, Helen Radford, Panagiotis Zis, Mark I. Johnson, David P. Finn, Patrick C. McHugh

**Affiliations:** 1 Centre for Biomarker Research, University of Huddersfield, Huddersfield, HD1 3DH UK; 2 School of Applied Sciences, University of Huddersfield, Huddersfield, HD1 3DH UK; 3 School of Human and Health Sciences, University of Huddersfield, Huddersfield, HD1 3DH UK; 4 St. James University Hospital, Leeds, LS9 7TF UK; 5 Medical School, University of Cyprus, Nicosia, Cyprus; 6 Centre for Pain Research, School of Clinical and Applied Sciences, Leeds Beckett University, Leeds, LS1 3HE UK; 7 Pharmacology & Therapeutics, School of Medicine, Galway, Neuroscience Centre and Centre for Pain Research, National University of Ireland Galway, University Road, Galway, Ireland

**Keywords:** Affymetrix microarray, Neuropathic, Chronic pain, Biomarkers, AUROC curve analysis, Inflammation

## Abstract

In this study, we recruited 50 chronic pain (neuropathic and nociceptive) and 43 pain-free controls to identify specific blood biomarkers of chronic neuropathic pain (CNP). Affymetrix microarray was carried out on a subset of samples selected 10 CNP and 10 pain-free control participants. The most significant genes were cross-validated using the entire dataset by quantitative real-time PCR (qRT-PCR). In comparative analysis of controls and CNP patients, *WLS *(*P* = 4.80 × 10^–7^), *CHPT1* (*P* = 7.74 × 10^–7^) and *CASP5* (*P* = 2.30 × 10^–5^) were highly significant, whilst *FGFBP2* (*P* = 0.00162), *STAT1* (*P* = 0.00223), *FCRL6* (*P* = 0.00335), *MYC* (*P* = 0.00335), *XCL2* (*P* = 0.0144) and *GZMA* (*P* = 0.0168) were significant in all CNP patients. A three-arm comparative analysis was also carried out with control as the reference group and CNP samples differentiated into two groups of high and low S-LANSS score using a cut-off of 12. *STAT1*, *XCL2* and *GZMA* were not significant but *KIR3DL2* (*P* = 0.00838), *SH2D1B* (*P* = 0.00295) and *CXCR31* (*P* = 0.0136) were significant in CNP high S-LANSS group (S-LANSS score > 12), along with *WLS (P* = 8.40 × 10^–5^), *CHPT1* (*P* = 7.89 × 10^–4^)*, CASP5* (*P* = 0.00393)*, FGFBP2* (*P* = 8.70 × 10^–4^) and *FCRL6* (*P* = 0.00199), suggesting involvement of immune pathways in CNP mechanisms. None of the genes was significant in CNP samples with low (< 12) S-LANSS score. The area under the receiver operating characteristic (AUROC) analysis showed that combination of *MYC*, *STAT1*, *TLR4*, *CASP5* and *WLS* gene expression could be potentially used as a biomarker signature of CNP (AUROC − 0.852, (0.773, 0.931 95% CI)).

**Supplementary Information:**

The online version contains supplementary material available at 10.1007/s12017-021-08694-8.

## Introduction

Chronic neuropathic pain (CNP) is a debilitating condition caused by lesion or disease of the peripheral and central divisions of the somatosensory system (Colloca et al., 2017; van Hecke et al., 2014). Drug management of CNP provides symptomatic relief in some but not all patients and is associated with hazardous side effects (Colloca et al., 2017; Hoffman et al., 2017). This is largely due to the lack of objective biomarkers to guide diagnosis and choice of treatment (Backryd, 2015). The mechanism of CNP is also complicated by multiple and obscure pain aetiologies and involvement of many molecular and cellular pathways contributing to the perception of pain (Campbell & Meyer, 2006). It is suggested that different clinical pain manifestations and prognosis may be due to different mechanisms of CNP (Campbell & Meyer, 2006). To help improve pain management, there is a need to develop translational tools, such as well-validated quantitative biomarkers as indicators of disease phenotype and drug response.

A plethora of inflammatory molecules dominate the gene expression (transcriptome) profile of patient blood in CNP (Backryd, 2015). Various inflammatory molecules are associated with gender (Lopes et al., 2017; Sorge et al., 2011), Leeds Assessment of Neuropathic Symptoms and Signs (S-LANSS) scores (Bennett et al., 2005) and other clinical presentations (Lasselin et al., 2016; Sommer et al., 2018). Many non-inflammatory proteins have also been suggested to act as molecular mediators of CNP and are attracting interest for drug discovery and development of more selective analgesic drugs (Sommer et al., 2018). Examples of proteins that have been tested as potential biomarkers for pain include neurotrophic factors (Boucher & McMahon, 2001; Kelleher et al., 2017), cytokines (Ellis & Bennett, 2013; Uceyler & Sommer, 2012), neuropeptides (Carniglia et al., 2017), endothelin (Hans et al., 2008), heat shock proteins (Hutchinson et al., 2009; Lei et al., 2017; Zou et al., 2012), and GTP-cyclohydrolase 1 (GCH1) (Latremoliere & Costigan, 2011; Tegeder et al., 2006), although this list is not exhaustive. Studies utilising genetic tools such as genome-wide association studies (GWAS), transcriptome analysis with quantitative real-time PCR (qRT-PCR), protein expression studies and animal models along with other techniques have vastly contributed to the understanding of the molecular biological mechanisms contributing to CNP. Nevertheless, there is a need for comprehensive studies to contextualise how signalling pathways and different molecules synchronise in CNP. Gene expression studies coupled with newly developed statistical and bioinformatics tools can be useful for acquiring information about the molecular regulation of transcriptional responses of the peripheral nervous system to traumatic nerve injury.

Recently, we carried out a DNA microarray of 10 patients with chronic lower back pain and 10 pain-free controls to identify differentially expressed genes (Buckley et al., 2018). These genes were cross-validated using qRT-PCR of samples of dorsal horn tissue of rats having undergone spinal nerve ligation (SNL) or sham (placebo) surgery (Buckley et al., 2018). In this work, we have carried out Affymetrix microarray and qRT-PCR to differentiate the gene expression profiles of pain-free control and CNP patients. It is noteworthy that most of the studies in pain biomarker and drug-target discovery had been carried out in rat models and cross-species validation could be a major challenge due to fundamental metabolic differences between rats and humans (Buckley et al., 2018; Denk et al., 2016; Hutchinson et al., 2009; Latremoliere & Costigan, 2018; Lopes et al., 2017; Xue et al., 2014). Our study is more powerful than previous studies because it is based on clinical samples and presents a more realistic and accurate outlook of the transcriptional changes associated with pain in humans.

## Materials and Methods

### Sample Acquisition

Samples for this study were collected at the University of Huddersfield (control subjects) and through the Pain Management Services at Seacroft Hospital, Leeds, UK. A total of 50 CNP patients and 43 pain-free healthy controls participated in the study (Supplemental Tables 1 and 2). The pain patients were recruited based on the CNP (> 3 months) as their clinical diagnosis. According to the 7-item Leeds Assessment of Neuropathic Symptoms and Signs Pain Scale (S-LANSS) questionnaire, pain with S-LANSS score ≥ 12 is considered as neuropathic pain (Bennett et al., 2005). However, some CNP participants recruited in this study had SLANSS score < 12 although the clinical diagnosis was the pain of neuropathic origin. We consider these pain patients as CNP participants with nociceptive components as well. Both the controls and pain patients were excluded for any current diagnosis of diabetes, cancer, osteoarthritis, fibromyalgia and other complex metabolic diseases which could significantly alter their gene profiles. Controls did not have any symptoms of pain.

**Table 1:** Characteristics of patients and controls recruited for the present study

Patient characteristics	Patient (*n* = 50)
**Age (years)**	
Median	45
Range	21–79
**Gender**	
Male	23
Female	27
**S-LANSS**^a^** score**	
Median	17.5
Range	0–24
Patients with S-LANSS score < 12	28%
Patients with S-LANSS score ≥ 12	72%
**PHQ-9**^b^ **score**	
Median	14
Range	0–29
CPG^c ^	
Median	IV
Range	I–IV

Drug categories	Patients taking the drugs (%)
Anti-inflammatory (NSAIDS^d^)	48
Antidepressants with analgesic properties	52
Anticonvulsants	48
Opioids	62

Control characteristics	Control (n = 43)
**Age (years)**	
Median	44
Range	18–68
**Gender**	
Male	15
Female	28
**PHQ-9 score**	
Median	2
Range	0–8

^a^Leeds Assessment of Neuropathic Symptoms and Signs (S-LANSS) pain scale

^b^Patient Health Questionnaire (PHQ)-9 (depression score)

^c^Chronic Pain Grade

^d^Non-steroidal anti-inflammatory drugs

**Table 2:** Fold changes and *P*-values of top nine upregulated and downregulated genes in Affymetrix microarray^a^ and qRT-PCR^b^

Transcript cluster ID	Gene symbol	Description	Microarray^a^		qRT-PCR^b^		
			Fold-change (linear)	ANOVA *P*-value	Fold-change (log scale)^c^	Fold-change^d^	*P*-value^e^
Genes upregulated in microarray							
TC11002692.hg.1	*MS4A2*	Membrane-spanning 4-domains, subfamily A, member 2	1.83	0.015	− 0.03	0.978	0.824
TC12002538.hg.1	*CHPT1*	Choline phosphotransferase 1	1.66	0.008	1.38	2.61	7.74E−07
TC0X001829.hg.1	*AMMECR1*	Alport syndrome, mental retardation, midface hypoplasia and elliptocytosis chromosomal region gene 1	1.51	0.043	− 0.08	0.930	0.745
TC01002763.hg.1	*WLS*	Wntless Wnt ligand secretion mediator	1.51	0.038	1.34	2.55	4.8E−07
TC05000307.hg.1	*NAIP*	NLR family, apoptosis inhibitory protein	1.46	0.024	0.06	1.04	0.600
TC11002819.hg.1	*TRIM51EP*	Tripartite motif-containing 51E, pseudogene	1.46	0.012	0.22	1.17	0.656
TC01001351.hg.1	*FCER1A*	Fc fragment of IgE, high affinity I, receptor for; alpha polypeptide	1.45	0.008	− 0.13	0.91	0.297
TC02004639.hg.1	*TNFAIP6*	Tumour necrosis factor, alpha-induced protein 6	1.43	0.019	0.14	1.12	0.446
TC11003322.hg.1	*CASP5*	Caspase 5	1.41	0.009	0.48	1.41	2.30E−05

Genes downregulated in microarray							
TC05000231.hg.1	*GZMA*	Granzyme A	− 1.37	0.027	− 0.38	0.770	0.0168
TC01003490.hg.1	*XCL2*	Chemokine (C motif) ligand 2	− 1.37	0.0005	− 0.40	0.772	0.014
TC04002941.hg.1	*FGFBP2*	Fibroblast growth factor binding protein 2	− 1.42	0.012	− 0.58	0.664	0.00162
TC03001304.hg.1	*CX3CR1*	Chemokine (C-X3-C motif) receptor 1	− 1.42	0.002	− 0.32	0.813	0.0978
TC01001356.hg.1	*FCRL6*	Fc receptor-like 6	− 1.48	0.01	− 0.49	0.716	0.00335
TC19002658.hg.1	*KIR3DL2*	Killer cell immunoglobulin-like receptor, three domains, long cytoplasmic tail, 2	− 1.33	0.048	− 0.45	0.745	0.0368
TC01003455.hg.1	*SH2D1B*	SH2 domain containing 1B	− 1.51	0.01	− 0.31	0.810	0.0417
TC12001202.hg.1	*KLRB1*	Killer cell lectin-like receptor subfamily B, member 1	− 1.63	0.002	− 0.21	0.871	0.588
TC07001296.hg.1	*TARP*	TCR gamma alternate reading frame protein	− 1.96	0.015	− 0.30	0.816	0.0740

^a^*n* = 10 controls, 10 CNP patients. Relative gene expression in microarray was calculated as a ratio of CNP average (log 2) and control average (log 2)

^b^*n* = 43 controls and *N* = 50 CNP participants

^c^Positive and negative values indicate upregulation and downregulation of genes, respectively, similar to fold-change output in microarray

^d^Values > 1 and < 1 indicate upregulation and downregulation of the gene, respectively. The relative gene expression in qRT-PCR is expressed as fold-change according to the 2^−∆∆Ct^ method

^e^*P*-values were derived using age and gender as control

We performed sample size calculations based on gene expression analyses of a previous study of 10 disease individuals and 10 non-disease controls. We included gene expression data generated both by microarray and by qPCR. Independent calculations based on four significant transcripts resulted in corresponding sample size requirements of 21, 16, 27 and 15 participants per group to be able to reject the null hypothesis that the population means of the diseased and control groups were equal with probability (power) 0.8. The Type I error probability associated with this test of this null hypothesis is 0.05. Conservatively the required sample size for the current investigation was taken as being the largest of these figures; i.e. 27 per group or 54 in total. Other non-selected genes, with correspondingly larger effects, would lead to lower requirements, whereas those genes with lower effects would in turn require larger numbers in the study, and hence we conservatively aimed to analyse 35 participants per group (70 in total). Allowing for 20% attrition loss, we aimed to recruit a total of 86 individuals to the current 2-arm study (43 per group). As CNP is often associated with co-morbidities, our main focus in patient recruitment was to use a relatively homogenous population without any comorbidity to identify changes associated with CNP. A similar strategy for patient recruitment was used in a previous study on transcriptomic analysis of lower back pain patients (Dorsey et al., 2019).

Data for age, gender, Patient Health Questionnaire (PHQ-9) and State-Trait Anxiety Inventory (STAI) questionnaires, STAI-I and STAI-II for current and trait anxiety, respectively, were collected for all the participants (See Supplementary file for details). Clinical data related to pain including pain duration in months, self-reported pain, Leeds Assessment of Neuropathic Symptoms and Signs (S-LANSS), Chronic Pain Grade (CPG) and current medications were also recorded for CNP participants. The medication histories of patients were collected at the time of sample acquisition. Current medications were categorised as follows: (a) anti-inflammatory drugs, which included nonsteroidal anti-inflammatory drugs (NSAIDS), (b) antidepressants such as tricyclic antidepressants (TCAs), norepinephrine-serotonergic reuptake inhibitors (NSRIs) and selective serotonin reuptake inhibitors (SSRIs), (c) anticonvulsants and (d) opioid analgesics.

Venous blood samples were collected from the antecubital fossa of all participants using standard phlebotomy technique. Blood for RNA isolation was collected in 2.5 mL PAXgene Blood RNA tubes (BD Diagnostics, Wokingham, Berkshire, United Kingdom), which were stored at − 20 °C for < 24 h before transfer to − 80 °C for long-term storage.

### Transcriptomic Analysis

A total of 10 samples from CNP (with a S-LANSS score > 12) and control groups (*n* = 20) were included for transcriptomic analysis. For transcriptomics, RNA was extracted using the PAXgene Blood RNA Kit (PreAnalytiX GmbH, Switzerland) as per the manufacturer’s instructions. Total RNA was labelled using an Ambion WT Expression kit (Life Technologies, Bleiswijk, Netherlands). The gene expression profiling was carried out using the GeneChip Human Transcriptome Array (HTA) 2.0 (Affymetrix, Santa Clara, CA, USA). The Affymetrix HTA 2.0 covered about 67,500 transcript clusters (genes), both coding and non-coding included. The labelling, hybridization, scanning and data extraction of microarray were performed by AROS Applied Biotechnology (Aarhus, Denmark) according to the recommended Affymetrix protocols. The digital signals obtained as CEL files were then processed using Affymetrix expression console (v1.4.1.46) with the standard configuration for expression arrays, including robust multi-array average (RMA) background correction (Irizarry et al., 2003), median polish probe-level signal summarization and quantile normalisation. The detectable genes were defined as significant detection signals [*P-*value (*P*) < 0.05] for more than 50% of probe sets in at least one of all samples. The normalised files were then followed by pairwise comparison analysis for obtaining differential gene expression between pain and control participants in Transcription analysis console v-4.0. Annotated transcripts, *P* < 0.05 (ANOVA) were considered suitable for further analysis.

### Bioinformatics Analysis

Mapping of pathways, networks and biological functions of differentially expressed genes was carried out using Ingenuity Pathway Analysis (IPA) (Qiagen, Redwood City, CA, USA). *P* < 0.05 was used as a cut-off and 1069 annotated genes were analysed (494 downregulated and 575 upregulated) (Supplemental Table 3). Core analysis was carried out and both direct and indirect relationships were considered to generate the gene networks (Kramer et al., 2014). When generating networks, we used the settings of a maximum of 140 genes per network and 10 networks per analysis, as the higher number of genes allows for the possibility that the same network can include all focus genes. The molecules or pathways specific to humans, experimentally observed and predicted with high confidence were used in IPA to analyse gene expression data in the context of known biological response and regulatory networks. IPA returns *P*-values (*P*) for the networks and regulators based on Fisher’s exact test, which is a measure of the probability that the association between a set of focus genes in the experiment and a given process or pathway is due to random chance alone (Kramer et al., 2014). Activation *z*-score was used to predict directionality based on the underlying findings, relationship bias and dataset bias (Kramer et al., 2014). The transcription factors with maximum interconnected genes in IPA were further analysed by qRT-PCR. IPA and String *version* 10.5 (Szklarczyk et al., 2017) were used to generate the figures showing gene networks.

**Table 3:** Top diseases and biological functions associated with the dataset in IPA

Top diseases and biofunctions		
Name	*P*-value range^*a*^	Number of molecules
**Diseases and disorders**		
Inflammatory response	2.89E−03 to 5.29E−10	222
Immunological disease	2.89E−03 to 1.65E−06	134
Gastrointestinal disease	2.89E−03 to 2.32E−06	148
Organismal injury and abnormalities	2.94E−03 to 3.19E−06	291
Cancer	2.94E−0.3 to 4.28E−06	177
**Molecular and cellular functions**		
Cell death and survival	2.51E−03 to 1.64E−10	274
Cellular compromise	2.80E−03 to 1.64E−10	75
Cell-to-cell signalling and interaction	2.91E−03 to 1.41E−07	197
Cellular movement	2.62E−03 to 4.10E−07	190
Cellular function and maintenance	2.81E−03 to 7.99E−07	141
**Physiological system development and function**		
Hematological system development and function	2.91E−03 to 7.82E−10	185
Tissue morphology	2.81E−03 to 7.82E−10	161
Lymphoid tissue structure and development	2.51E−03 to 1.97E−07	125
Immune cell trafficking	2.62E−03 to 4.75E−06	115
Digestive system Development and function	2.40E−03 to 6.27E−06	44

^a^The *P*-value indicates significance with which the attributes were associated with the dataset and the number of molecules indicates the number of genes from the dataset that were associated with it

### Quantitative PCR (qRT-PCR)

The qRT-PCR of selected genes was carried out for all participants. A total of 300 ng of RNA from each human sample was reverse transcribed using the Verso cDNA Synthesis Kit (Thermo Scientific™, UK) according to the manufacturer’s instructions. The complementary DNA was subsequently diluted tenfold. Amplification was performed in triplicate with a Roche LightCycler® 480 II (Roche Diagnostics Ltd., Burgess Hill, West Sussex, United Kingdom). Each 10 μl reaction mixture contained 3 μl of iTaq™ Universal SYBR® Green Supermix (Bio-Rad Laboratories, Berkeley, CA, USA), 300 nM of each forward and reverse primer (Supplemental Table 4) and 1 μl of diluted cDNA. Amplification protocol was as follows: Polymerase activation and DNA denaturation at 95 °C for 2 min, 40 cycles of denaturation at 95 °C for 5 s with annealing and extension at 60 °C for 30 s followed by fluorescence detection. Upon completion of thermal cycling, melt-curve analysis was carried out to confirm reaction specificity. The relative gene expression of the markers was normalised to the geometric mean of *GAPDH* and *SDHA* (Supplemental Table 4) and then to the control group, according to the 2^–ΔΔ*Ct*^ method (Livak & Schmittgen, 2001). The levels of expression for each gene are presented as fold-changes in comparison to controls.

**Table 4:** Ten networks associated with the dataset in the IPA analysis

Network ID	Score^a^	Number of focus molecules	Top diseases and functions
1	161	123	Developmental disorder, hereditary disorder, neurological disease
2	88	86	Cancer, organismal injury and abnormalities, cell death and survival
3	88	86	Cell-to-cell signalling and interaction, cellular compromise, inflammatory response
4	66	72	Inflammatory disease, organismal injury and abnormalities, respiratory disease
5	64	71	Developmental disorder, neurological disease, organismal injury and abnormalities
6	63	70	Post-translational modification, protein degradation, protein synthesis
7	61	69	Cancer, cardiovascular disease, developmental disorder
8	58	67	Post-translational modification, cellular compromise, nutritional disease
9	57	66	Hematological system development and function, tissue morphology, cell-to-cell signalling and interaction
10	53	63	Cancer, organismal injury and abnormalities, gastrointestinal disease

^a^The score for each network is a measure of the fit of that network to the user-defined set of focus Genes. The score is derived from a *P*-value and indicates the likelihood of the focus Genes in a network being found together due to random chance

### Statistical Analysis

Statistical analyses were carried out using in IBM SPSS Statistics version 26 taking into account qRT-PCR-based normalised gene expression, age and gender of the patients/control, the number of months patients have experienced pain, S-LANSS score, PHQ-9 score, CPG and ongoing medications of the patients. Medication data were recorded as binary variables, with intake of one or more individual medications within each medication group corresponding to a positive response. Primary outcome data were represented by gene expression levels, recorded on 23 genes of interest (See “Results” section for further details). Secondary outcome data were represented by the PHQ-9 scores, which was considered as both a potential predictor and outcome measure, due to uncertainties about its position on the causal pathway.

The sample was summarised descriptively.

The extent and nature of missing data were examined. Very small proportions of missing data were recorded. Little’s test revealed no evidence that this data was not missing completely at random (MCAR) (*P* = 0.207).

The *P*-values obtained from the ANCOVAs associated with the grouping variable were ordered and ranked. The Benjamini–Hochberg (B–H) critical value associated with each test was calculated, with the critical value for the *i*th *P*-value given by the expression $Q\left( {i/m} \right)$, where *m* is the number of genes to be tested (23 in all analyses) and *Q* is the false discovery rate (FDR); set at 5% for all analyses. Any gene for which the *P*-value was lower than the corresponding B–H critical value was deemed to be significant (and highlighted in tabulated data); as were all genes above it in the ordered list (i.e. those with lower *P*-values). Significance was also determined using familywise error rate (FWER) control via the Bonferroni method (using a critical significance level of 0.217%); and using uncorrected *P*-values, to assess the sensitivity of the gene selection to the method used.

A series of univariate main effects analyses of covariance (ANCOVAs) were conducted on the data. The *P*-values obtained from the ANCOVAs associated with the grouping variable were ordered and ranked. The false discovery rate (FDR) was set at 5% for all analyses. Expression levels for each gene were considered in turn as the outcome. The PHQ-9 depression score was also considered as a secondary outcome. Goodness-of-fit of the models was assessed from the range of adjusted-*R*^2^ statistics obtained.

We carried out three series of univariate main effects ANCOVAs on the data:

1. The first series of analyses included all CNP pain and control participants. Predictor variables included in these analyses were participant type (CNP or control), with age and gender included as controlling variables.
2. In the second series of analyses a distinction was made between 50 CNP patients based on the S-LANSS score. Hence the predictor variables included in these analyses were patient type; CNP with SLANSS score ≥ 12, CNP with SLANSS score < 12, control (considered to represent the reference category). Age and gender were additionally included as controlling variables.
3. A further series of univariate main effects ANCOVAs were conducted on the data arising from CNP patients only. In these analyses, the grouping variable was data available for pain patients only including CPG, months of pain experienced and medication in addition to gender and age. To avoid model overfitting, a sequential modelling strategy was utilised to eliminate predictor variables of no substantive importance. Predictor variables were assigned to blocks. The first block comprised the patient-reported PHQ-9 and CPG scores, plus the number of months for which the patient had experienced pain. The second block comprised the variables related to patient medications. The third block included age, gender and the grouping variable. In each of the lower blocks, patterns of significance amongst considered variables were examined, and any exhibiting greater levels of significance than expected were carried forward for inclusion as higher-priority variables in the next block.

The significant genes were grouped together for calculation of the area under the receiver operator characteristic (AUROC) curve both for the control versus CNP group comparison, and the high versus low S-LANSS group comparison. Following standard procedures, the ROC Curves were derived from predicted probabilities of a logistic regression analysis using the grouping variable as the state variable.

### Ethical Approval

The blood samples used in this study were obtained with informed consent from the patients. ﻿All the methods were performed in compliance with the institutional protocols. This study was approved by the Yorkshire & The Humber – Bradford Leeds Research Ethics Committee (14/YH/0117) and adapted to the NIHR Clinical Research Network (Portfolio ID: 16774).

## Results

### Descriptive and Exploratory Analysis of the Participants

The characteristics of study participants are presented in Table 1 and Supplemental Tables 1 and 2. Amongst the 50 CNP participants, 36 patients had SLANSS score ≥ 12 whilst the remaining 14 patients had SLANSS score < 12 (Supplemental Table 2).

The pain group comprised 23 men (46.0%) and 27 women (54.0%); with a mean age of 46.5 years (SD 12.5 years). The control group comprised 15 men (34.9%) and 28 women (65.1%); with a mean age of 38.4 years (SD 14.8 years). One missing value was recorded on each of the PHQ-9 score and CPG. Missing values were not imputed.

In the CNP group, 24 patients (48.0%) took analgesics (non-steroidal anti-inflammatory drugs); 25 patients (50.0%) took antidepressants with significant analgesic properties; 22 patients (44.0%) took anticonvulsants; 33 patients (66.0%) took opioid analgesics. 48 out of 50 patients took medication from at least one group; with 4 patients taking medications from all 4 groups. The median number of groups from which medication taken was 2.

Within the pain group, the mean S-LANSS score was 15.3 (SD 7.96; range 0–24). The mean PHQ-9 score was 13.2 (SD 7.34; range 0–26); the median CPG was IV (range I-IV). The mean PHQ-9 score in controls was 2.21 (SD 2.34; range 0–8). Four patients (8.2%) had a chronic pain grade (CPG) of I; 8 patients (16.3%) had a CPG of II; 10 patients (20.4%) had a CPG of III; 27 patients (55.1%) had a CPG of IV.

Considering the 50 patients in the CNP group, 36 (72.0%) had SLANSS score ≥ 12 whilst 14 (28.0%) had SLANSS score < 12. The CNP patients with SLANSS score ≥ 12 comprised 15 men (41.7%) and 21 women (58.3%); with a mean age of 43.9 years (SD 11.4 years). The CNP patients with SLANSS score < 12 comprised 8 men (57.18%) and 6 women (42.9%); with a mean age of 53.1 years (SD 13.1 years).

In the CNP group patients with SLANSS score ≥ 12, 17 patients (47.2%) took analgesics (non-steroidal anti-inflammatory drugs); 18 patients (50%) took antidepressants with significant analgesic properties; 19 patients (52.7%) took anticonvulsants; 23 patients (63.8%) took opioid analgesics. 33 out of 36 patients took medication from at least one group; with 5 patients taking medications from all 4 groups.

Amongst the CNP group with SLANSS score < 12, 7 patients (50%) took analgesics (non-steroidal anti-inflammatory drugs); 3 patients (21.4%) took antidepressants with significant analgesic properties; 5 patients (35.7%) took anticonvulsants; 8 patients (57.14%) took opioid analgesics. 12 out of 14 patients took medication from at least one group; no patients took medications from all 4 groups.

### Transcriptomic Analysis

**Fig. 1:**
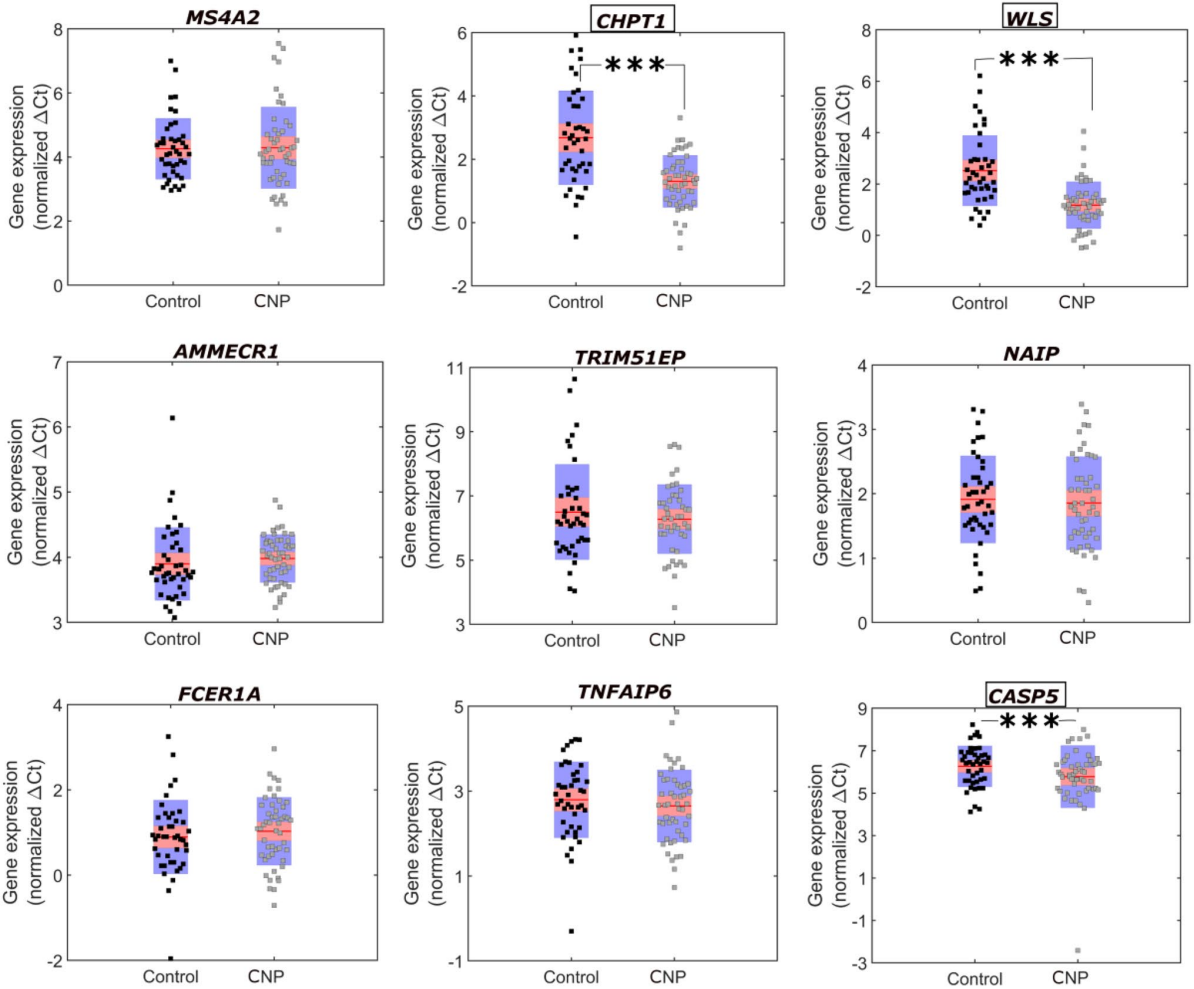
**Validation of top upregulated genes observed in the Affymetrix analysis of CNP vs control samples in larger subset by qRT-PCR.** ∆Ct is inversely related to the gene expression. In the box red line shows the mean and pink and blue area indicates values within 95% confidence interval and standard deviation 1, respectively. The markers indicate the normalised ∆*C*_*t*_ values of the genes in the samples. The names of statistically significant genes are outlined in a box and their *P*-values are marked with asterisks within the figure; ***P < 0.001

**Fig. 2:**
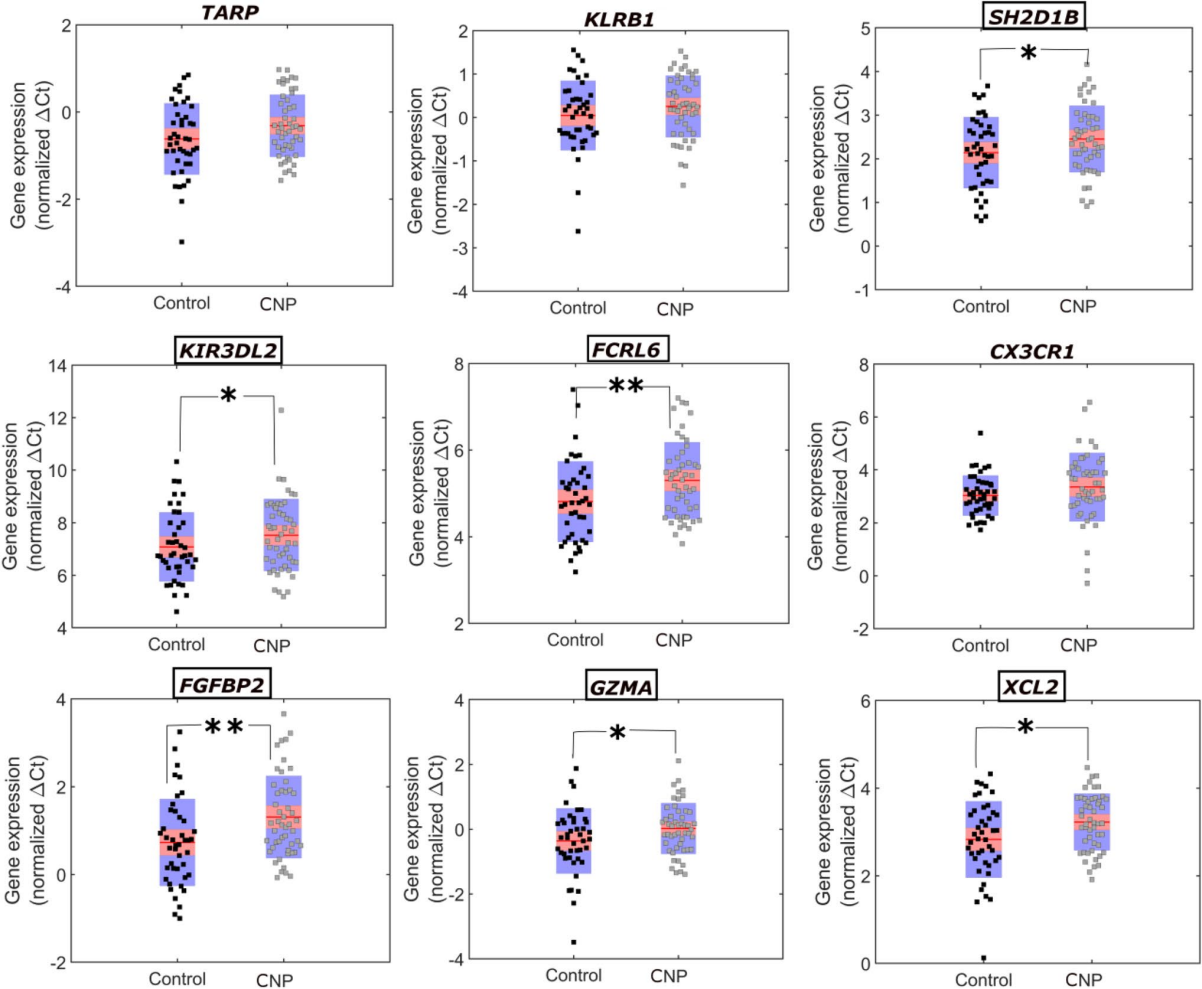
**Validation of top downregulated genes observed in the Affymetrix analysis of CNP vs control samples in larger subset by qRT-PCR.** ∆Ct is inversely related to the gene expression. In the box red line shows the mean and pink and blue area indicates values within 95% confidence interval and standard deviation 1, respectively. The markers indicate the normalised ∆Ct values of genes in the samples. The names of statistically significant genes are outlined in a box and their *P*-values are marked with asterisks within the figure; **P < 0.01 and *P < 0.05

All the arrays (10 CNP and 10 controls) passed the quality control tests carried out using Affymetrix console. The differential gene expression analysis of CNP vs control revealed the fold-change, *P*-value (*P*) and FDR-corrected *P*-value of the gene. None of the genes in the microarray could pass the criteria of FDR < 0.1. The principal component analysis (PCA) revealed that CNP and control groups were not clearly segregated across the first two PCAs (Supplemental Fig. 1). Therefore, we refrained from deriving any strict quantitative conclusions based on the microarray data and also cross-validated the top upregulated and downregulated genes using qRT-PCR. The top significant genes (*P* < 0.05) are presented in Table 2 and normalised gene expression as estimated by qRT-PCR is shown in Figs. 1 and 2.

**Fig. 3:**
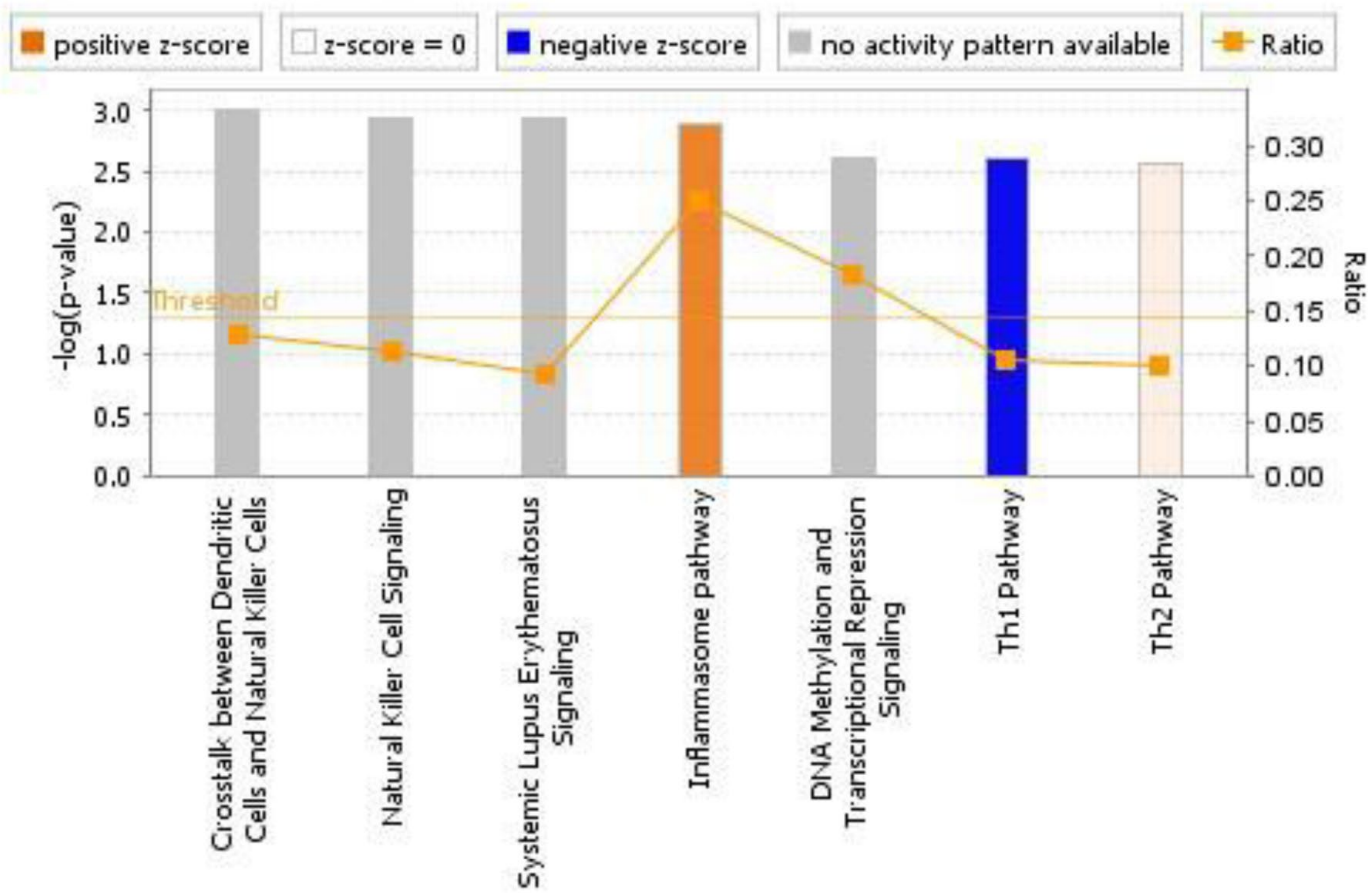
**Canonical pathways in IPA analysis which are most significant to the dataset using a *P*-value 1 × 10**^**–4**^** as the cut-off**. The ratio of the number of genes from the dataset that map to the pathway divided by the total number of genes that map to the respective pathway is indicated on the right-side *y*-axis

IPA core analysis was carried out on annotated differentially expressed genes obtained from microarray analysis using a cut-off of *P* < 0.05 (Supplemental Table 3). The core analysis associated the dataset with several canonical pathways each of which correlated with a *P*-value as described in the “Methods” section. A low *P*-value implies over-representation of focus genes in the pathway. We used the *P* < 1 × 10^−4^ as a cut-off which revealed the seven most significant pathways (Fig. 3). The inflammasome pathway (*z*-score = 2.236) and Th1 pathway (*z*-score = − 2.233) showed the highest and lowest *z*-score, respectively (Supplemental Figs. 2–4). The positive and negative *z*-score implied the predicted activation and inactivation of pathway, respectively. It also calculated a ratio of the number of genes from the list included in the canonical pathway and the total number of genes that make up the canonical pathway. The inflammasome pathway ranked the highest with a ratio of 0.25 (Fig. 3).

The IPA core analysis also provided the most significant diseases and disorders that could be linked to the dataset across all the genes (Table 3). The top disease identified, with 222 molecules, was in general related to the inflammatory response which further reflects the role of inflammatory genes in pain phenotype. The smaller *P*-values imply that the association is non-random.

**Fig. 4:**
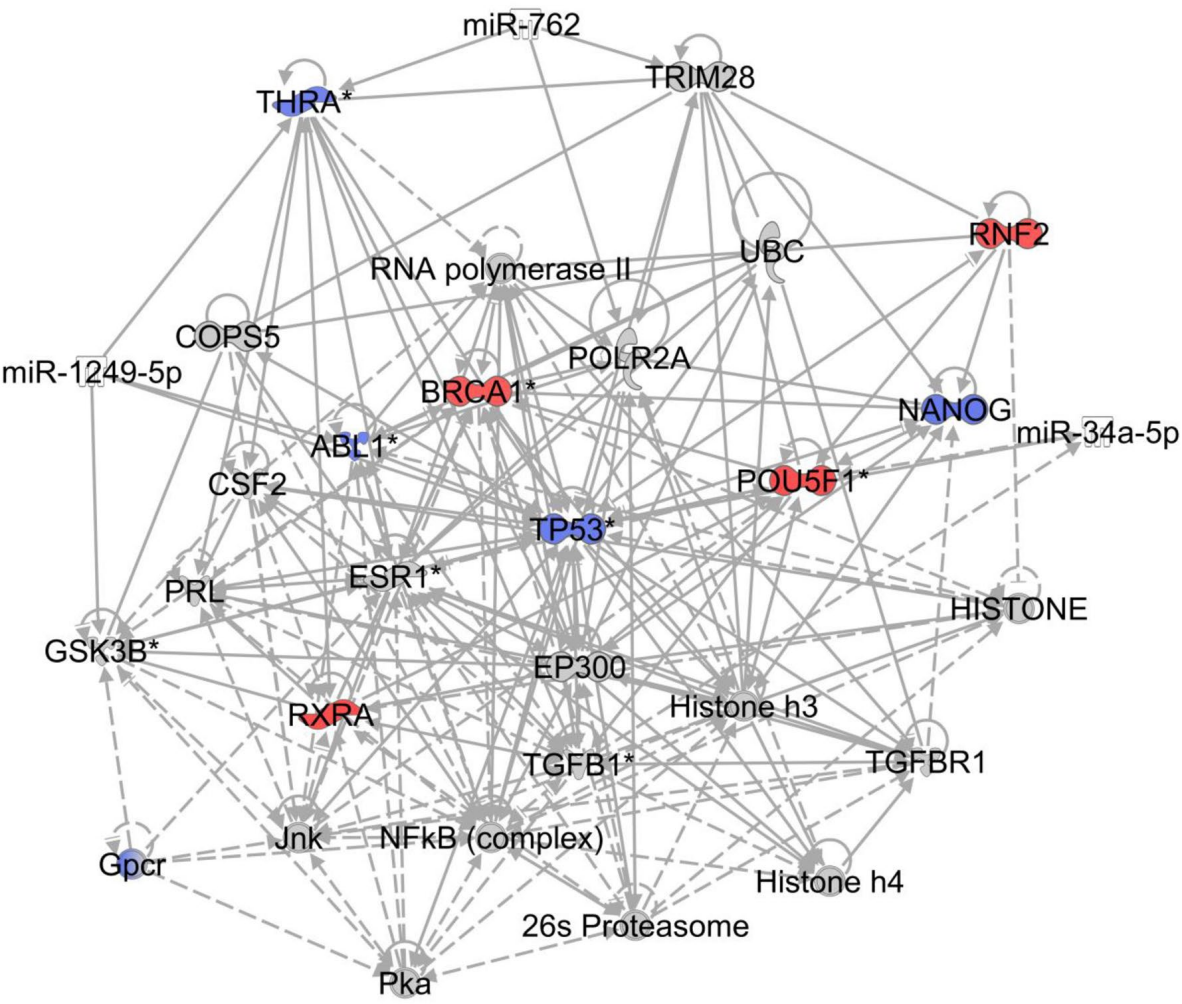
**Highly connected nodes in IPA based on the Affymetrix microarray data.** A master network generated by merging top five networks identified the above nodes with maximum connectivity (> 35 connections). The nodes that were upregulated and downregulated in the dataset are coloured red and blue, respectively. The nodes that have been predicted by IPA and were not present in the input dataset for microarray are filled in grey. The solid and dashed lines show direct and indirect connections, respectively. The circle around the node itself indicates self-regulation. The legend to the node shapes is presented in Supplemental Fig. 3

We limited our further IPA analysis to ten network functions (Table 4). All the generated networks were interconnected by one or more genes. The most significant network was associated with biological functions of developmental disorders, hereditary disorders and neurological diseases and included 123 focus molecules (Table 4). We merged the top five generated networks to obtain a master network of 687 focus molecules. The molecules with more than thirty-five interconnections are presented in Fig. 4 and these could be important in pain manifestations since it has been hypothesised that highly connected molecules are most likely associated with diseases or biological functions (Barabasi et al., 2011; Jeong et al., 2000). This also included Glycogen synthase kinase 3 beta (*GSK3B*) which had more than 50 connections in the generated master network.

### Key Transcription Factors in CNP Networks

**Fig. 5:**
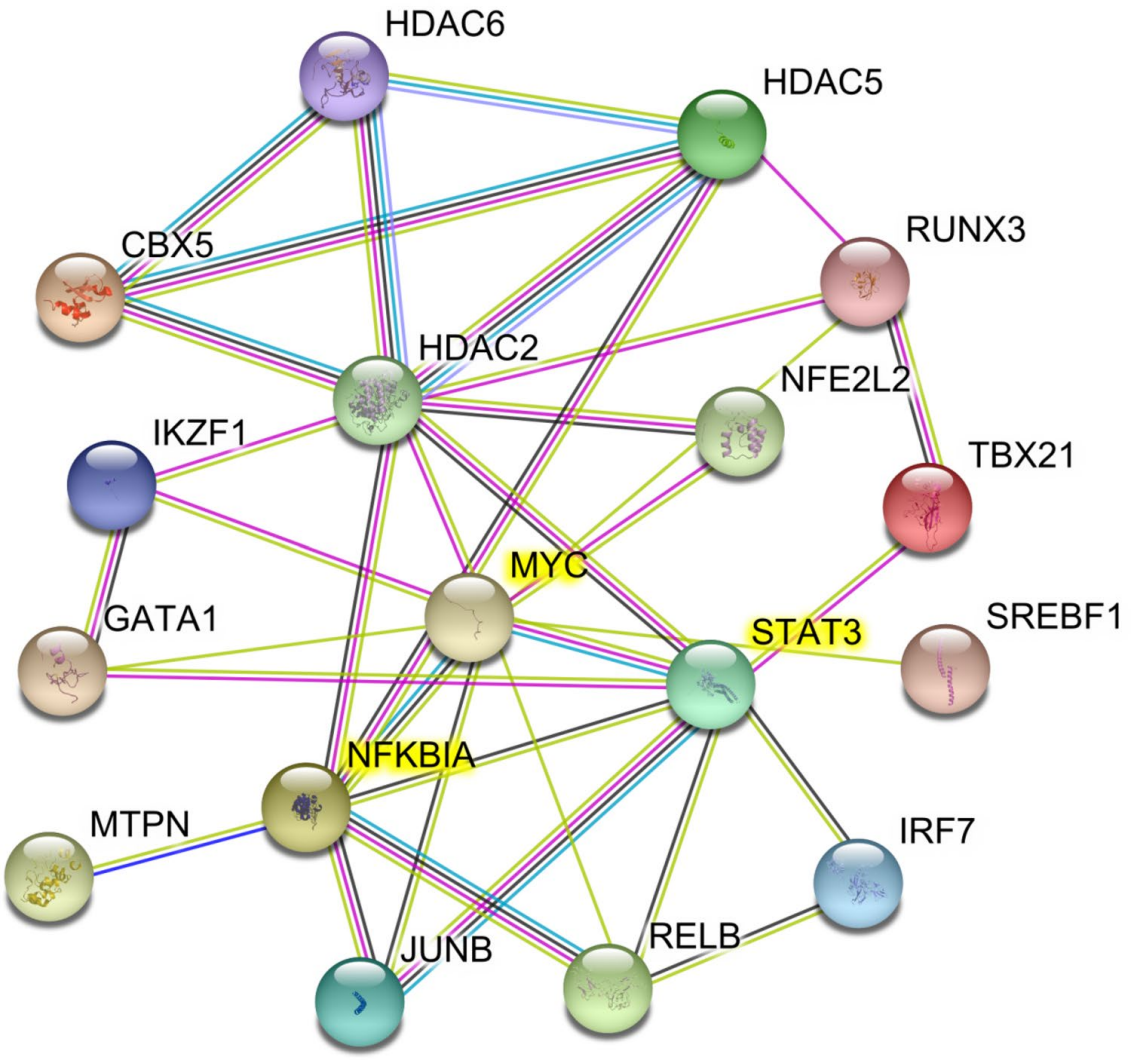
**Merged networks of transcription regulators with *z*-score >|2.0| in IPA.** The transcription factors connected to more than 25 nodes are highlighted in yellow. The figure was generated in the program STRING. The coloured lines between the transcription factors are related to source of the database/relation between the genes; cyan: Curated databases, magenta: experimentally determined, blue: gene co-occurrence, black: co-expression, light blue: protein homology and light green: text mining

The IPA analysis predicted various upstream regulators based on the differential gene dataset. We filtered the upstream regulators using transcription regulator as molecule type and *z*-score >|2| to predict key transcription regulators. Also, as the target molecules of many upstream regulators show an overlap, we merged the regulatory networks to display as one network. These included *STAT3*, *HDAC5*, *JUNB*, *CBX5*, *IRF7*, *HDAC6*, *NFE2L2*, *HDAC2*, *IKZF1*, *GATA1*, *NFκBIA*, *RUNX3*, *SREBF1*, *TBX21*, *RELB*, *MTPN* and *MYC* (Fig. 5). The transcription factors *STAT3*, *NFκBIA* and *MYC* showed more than 25 interconnections. NFκB pathway has been long associated with inflammation (Lawrence, 2009; Tak & Firestein, 2001), a hallmark of CNP (Hartung et al., 2015; Shih et al., 2015) so we did not analyse it any further. The transcription factors *STAT3* and *MYC* were analysed by qRT-PCR for cross-validation. We also carried out the qRT-PCR of *STAT1* as it has been suggested to play a role in CNP (Denk et al., 2016). Also, *STAT1* directly regulates T-box 21 (TBX21) which is one of the top regulatory molecules predicted by IPA based on our dataset.

We also carried out pathway analysis of our microarray dataset by the in-built WikiPathways (Slenter et al., 2018) function in Transcription analysis console v-4.0. The PI3-Akt pathway was the top pathway by count linked to the dataset (Supplemental Fig. 5). PI3-Akt pathway includes both *GSK3B* and *MYC* which have been suggested as the important nodes in the IPA analysis (Supplemental Fig. 5).

### qRT-PCR-Based Validation of Differentially Expressed Genes (CNP vs Control)

We carried out the qRT-PCR of the eighteen most frequent upregulated and downregulated genes observed in the microarray. A direct comparison of fold-change between the two methods (microarray and qRT-PCR) was not possible (Dallas et al., 2005; Morey et al., 2006), although some broad conclusions could be derived. Table 2 shows that the directionality of differential gene expression was nearly the same in the two methods for all of the upregulated and downregulated genes. The qRT-PCR-based normalised gene expression of these genes in CNP and control samples is presented in Figs. 1 and 2. We also estimated the age- and gender-controlled *p*-values of qRT-PCR data between CNP and control participants and an FDR of 5% was applied to filter the significant genes (Supplemental Table 5). Genes with a *P* < 0.05 were considered significant in the qRT-PCR analysis. Amongst the upregulated genes, choline phosphotransferase 1 (*CHPT1*) gene was highly significant in both the microarray (*P* = 0.0086) and qRT-PCR (*P* = 7.74 × 10^–7^, respectively). Wntless Wnt ligand secretion mediator (*WLS*) was significant in the microarray (*P* = 0.038) whilst it was found to be strongly significant in qRT-PCR (*P* = 4.80 × 10^–7^). Amongst the downregulated genes, chemokine (C motif) ligand 2 gene (*XCL2*) was highly significant in the microarray (*P* = 0.0005) but showed less significance in qRT-PCR (*P* = 0.0144). Fibroblast growth factor binding protein 2 (*FGFBP2*) was significant in microarray (*P* = 0.013) and highly significant in qRT-PCR (*P* = 0.00162). The Fc receptor-like 6 gene (*FCRL6*) was significant in both microarray (*P* = 0.010) and qRT-PCR (*P* = 0.00335).

We carried out the qRT-PCR of Toll-like receptor 4 (*TLR4*) as previous studies have suggested its direct or indirect role in CNP (Hutchinson et al., 2009; Shah & Choi, 2017; Sorge et al., 2011). In the microarray, *TLR4* was overexpressed in CNP patients (*P* = 0.036) and showed a similar significance in CNP vs control qRT-PCR analysis (*P* = 0.0368) but was not significant when an FDR cut-off of 5% was applied (Supplemental Fig. 6, Supplemental Table 5). *GSK3B* was identified by IPA analysis of microarray data (Fig. 4) and previous studies have suggested its role in CNP (Gobrecht et al., 2014; Maixner & Weng, 2013). In the present work, CNP *vs* control differential expression of *GSK3B* was non-significant at the transcriptional level both in the microarray and qRT-PCR (Supplemental Fig. 6, Supplemental Table 5). IPA suggested signal transducer and activator of transcription 1 and 3 (*STAT1* and *STAT3)* and *MYC* as important transcription factors in our dataset. *STAT1* and *MYC* were significantly upregulated (*P* = 0.001 for both) in the patients with CNP compared to the controls whilst *STAT3* did not show any significant differential expression (Supplemental Fig. 6, Supplemental Table 5).

We carried out a series of statistical analyses of qRT-PCR gene expression data from all participants. The qRT-PCR data of the eighteen differentially expressed genes derived from the microarray data and *TLR4*, *STAT1* and *3, GSK3B* and *MYC* were included in this analysis. Analysis carried out between controls and all CNP samples with age and gender as control revealed *WLS*, *CHTP1*, *CASP5*, *FGFBP2*, *STAT1*, *FCRL6*, *MYC*, *XCL2* and *GZMA* to be significantly associated with CNP under an FDR of 5% (*P* = 4.80 × 10^–7^, 7.74 × 10^–7^, 2.30 × 10^–5^, 0.0016, 0.0022, 0.00335, 0.00335, 0.0014 and 0.0168, respectively) (Supplemental Table 5).

We also carried out a three-arm analysis, with CNP grouped into two categories based on S-LANSS score cut-off of 12, and the control group used as the reference category. Ranking of *p*-values for multiple comparisons, under an FDR of 5%, revealed *WLS*, *CHTP1*, *FGFBP2*, *FCRL6*, *SH2D1B*, *CASP5*, *KIR3DL2* and *CXCR31* revealed to be significantly (*P* = 8.40 × 10^–5^, 7.89 × 10^–4^, 8.70 × 10^–4^, 0.002, 0.003, 0.004, 0.0084, 0.0136, respectively) associated with CNP with S-LANSS score ≥ 12 (Supplemental Table 6). However, none of the genes was significant with CNP with S-LANSS score < 12 (Supplemental Table 7). The genes *SH2D1B*, *KIR3DL2* and *CXCR31* were only associated with CNP samples with S-LANSS score ≥ 12.

**Fig. 6:**
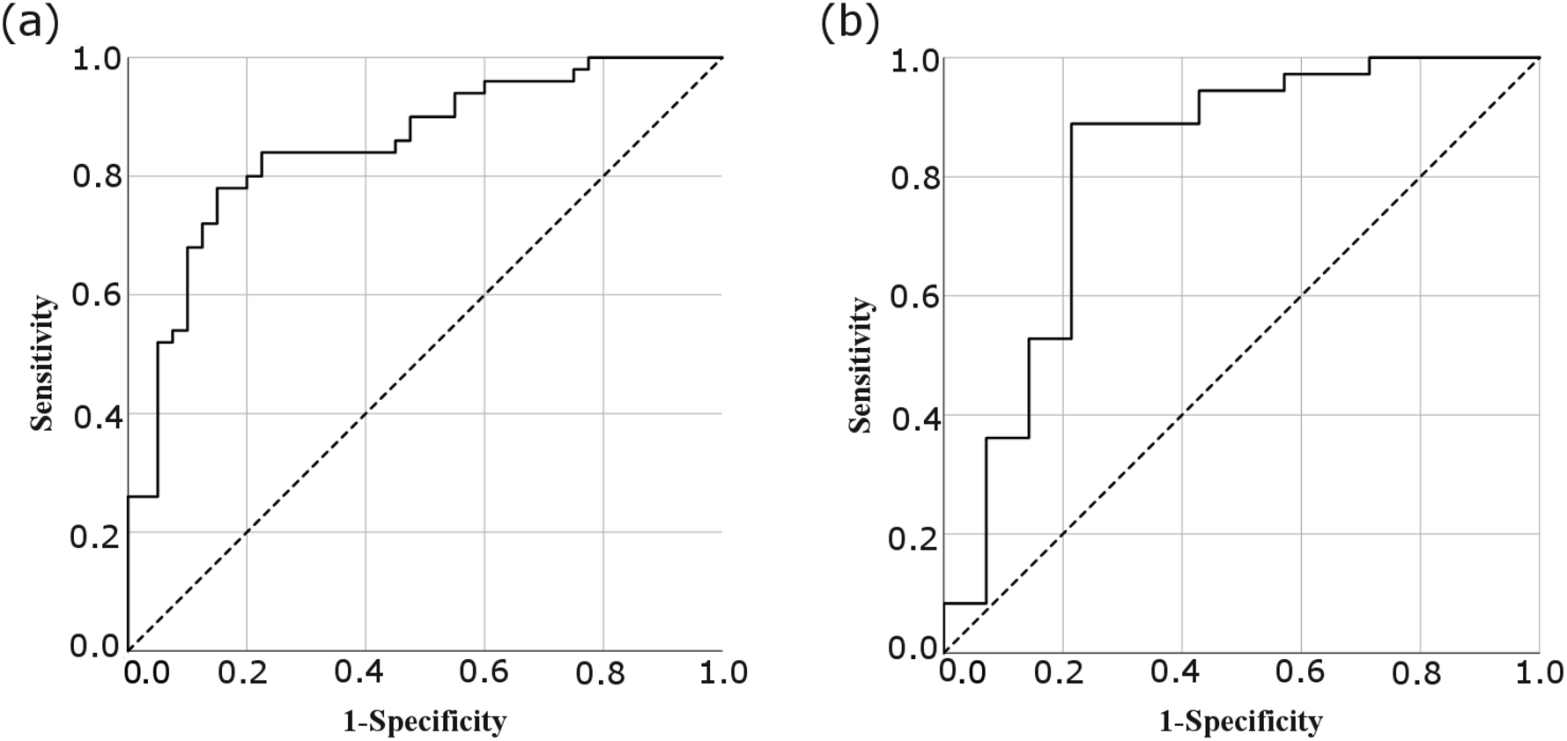
**Area under the ROC curve (AUROC) analysis.** AUROC formed using *WLS, MYC, STAT1, CASP5 and TLR4 gene expression between ***a*** control and all CNP patients and ***b*** high (*≥ *12) and low (*< *12) S-LANSS of CNP group. The combination of these gene expressions are robust enough to diagnose CNP*

A combination of expression data of genes significant between control and CNP groups that were also associated with the PI3-Akt pathway namely; *MYC, STAT1, TLR4, CASP5* and *WLS* showed the AUROC of 0.852 (0.773, 0.931, 95% CI) suggesting that it could be used as a biomarker signature for CNP (Fig. 6a). The perturbations of these genes are robust to also compare high and low S-LANSS CNP patients (AUROC-0.819 (0.666, 0.973, 95% CI)) (Fig. 6b).

### Effect of Medications on the Gene Expression

The series of ANCOVAs conducted on gene expression data using medication variables as predictors, revealed that anti-inflammatory, antidepressants and anticonvulsants drugs were all statistically significant at the 5% level in 1 out of 26 genes. These findings were consistent with a uniform distribution of *P*-values that would be expected from repeated instances of tests under a null hypothesis of no association. Hence, none of these variables were carried forward for inclusion in subsequent blocks. Opioid analgesics were statistically significant at the 5% level in 6 out of 26 genes. This finding was not consistent under a null hypothesis of no association, so this variable was carried forward for inclusion in the final blocks together with the grouping variable of CNP and the controlling variable age, gender and pain type (nociceptive and neuropathic pain).

**Fig. 7:**
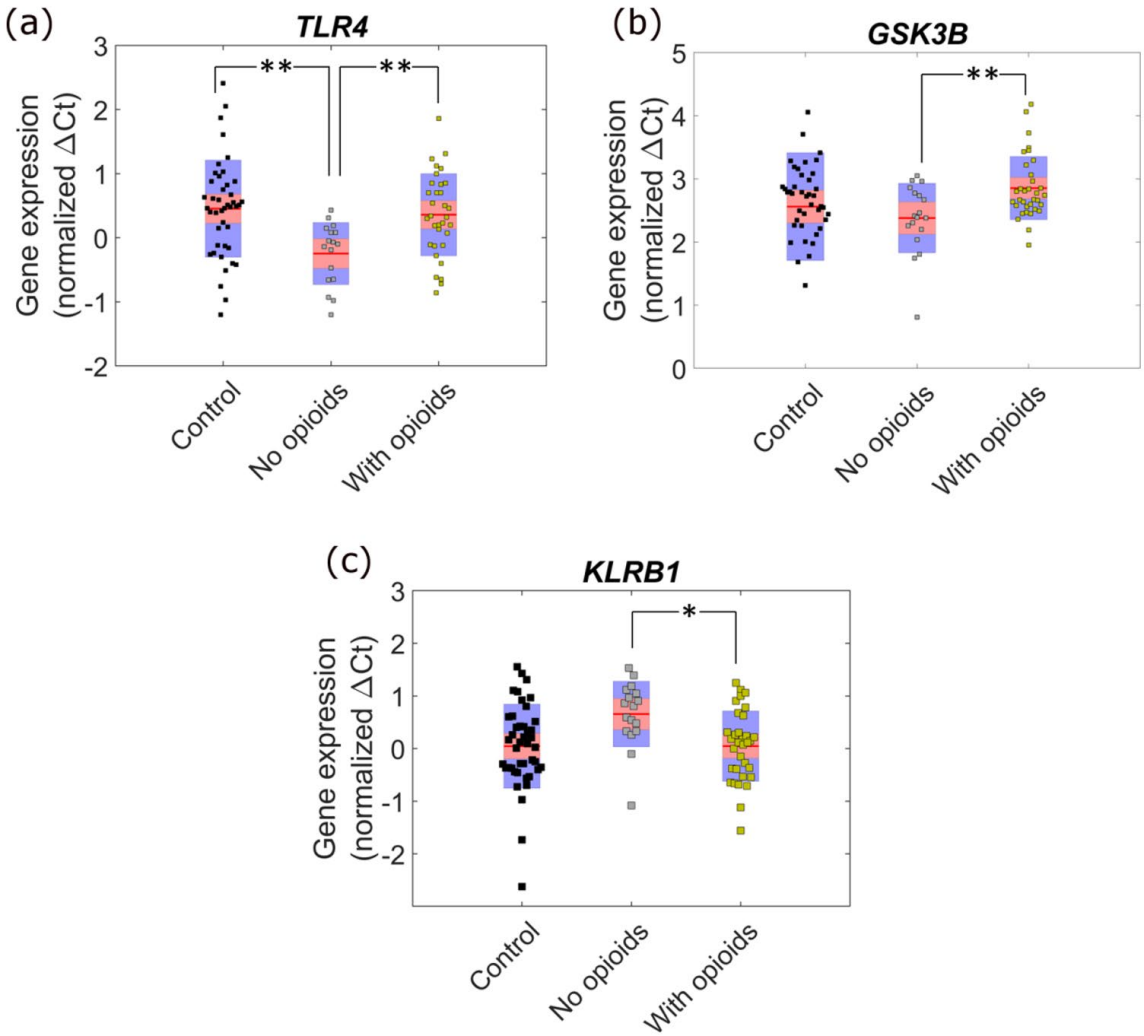
**Comparison of *TLR4*,** ***GSK3B* and** ***KLRB1*** **gene expression of CNP patients without and with opioids as the medication and controls.** In the box, red line shows the mean and pink and blue area indicate values within 95% confidence interval and standard deviation 1, respectively. The markers in the box indicate normalised ∆*C*_*t*_ values of the genes in the group. ∆*C*_*t*_ is inversely related to the gene expression. The no opioids and with opioids in the figure refer to the gene expression in patients without and with opioid medications. The statistically significant *P*-values are shown in the figure, **P < 0.01 and *P < 0.05

Assuming an FDR of 5%, the genes *TLR4* (*P* = 8.58 × 10^–4^), *GSK3B* (*P* = 0.00176) and *KLRB1* (*P* = 0.00529) were significantly associated with the intake of opioid analgesics (Supplemental Table 8). The comparison of gene expression of *TLR4* and *GSK3B* in the CNP patients showed that the expression of these genes was decreased in patients taking opioids against those not on opioid medications (Fig. 7). On the other hand, *KLRB1* expression was higher in patients taking opioid medications.

## Discussion and Conclusions

The screening of CNP is largely based on clinical diagnoses and questionnaires that have been used in the epidemiological studies (Bouhassira & Attal, 2011; Torrance et al., 2006). In the present study, our aim was to identify potential blood biomarkers for effective diagnoses and treatment of CNP. Blood biomarkers can also serve to identify potential perturbations in molecules and pathways that maybe linked to disease processes related to pain including in the CNS (Buckley et al., 2018; Yamamotova et al., 2010). We used a relatively homogeneous population for this study by excluding patients with cancer, fibromyalgia, osteoarthritis, diabetes and other complex metabolic disorders as these can change the landscape of expressed genes significantly and which may be correlated to CNP.

A critique of methodological limitations is necessary before discussing the main outcomes of the study. In our Affymetrix microarray results, the linear fold-change of most of the genes was between − 1.5 and 1.5. One reason for this could be that CNP and other nervous system disorders such as schizophrenia and autism are caused by subtle changes in many genes (Barnes et al., 2011; James, 2013). When comparing few genes with low fold-changes across a large number of genes, the calculated FDR values are very high (Tusher et al., 2001). Therefore, none of the genes in the CNP *vs* control comparison of microarray data could pass an FDR of 0.1. Also, each patient had a different medical history including ongoing medications. These medications can lead to variation within the group and affect the average value of gene expression in the group. Therefore, we have used a large sample set for confirming the gene perturbations by qRT-PCR and used a rigorous statistical approach for the analysis of qRT-PCR data.

Despite these limitations, our study provides some interesting results. The most significantly perturbed genes in CNP samples were associated with inflammation ascertaining that the state of inflammation was maintained in all the pain patients. This has also been observed in the recent studies carried out using human neural tissues of CNP patients (North et al., 2019; Tavares-Ferreira et al., 2019). The fact that inflammation-related perturbations could be detected in the blood of CNP samples in this study establishes that inflammation is a hallmark of CNP, and that blood is not only a source of tissue can not only provide easily accessible, but also potential insights into disease processes. The genes *CHTP1*, *WLS*, *MYC and CASP5* that were significant in all CNP samples are associated with inflammation. *CHPT1* regulates phosphatidylcholine biosynthesis and its involvement in pain manifestation has yet not been demonstrated (Jia et al., 2016). On the other hand, indirect roles of *WLS* and *MYC* in inflammation have been suggested (Chen et al., 2018). *WLS* regulates secretion and function of Wnt proteins which are crucial for neuronal development (Patapoutian & Reichardt, 2000). WLS interacts with mu-opioid receptor (MOR) and it has been suggested that in the presence of opioids, the interaction of WLS with MOR can lead to decreased Wnt secretion further causing decreased neurogenesis (Jin et al., 2010). WLS can also lead to inflammation by activating NF-κB signalling (Wang et al., 2012a, 2012b). A potential role of *MYC* in inflammation has also been suggested (Descalzi et al., 2017; Liu et al., 2015; Sipos et al., 2016). *CASP5* is a proinflammatory caspase linked to the formation of inflammasome and is upregulated in various neuroinflammatory conditions including multiple sclerosis and osteoarthritis (An et al., 2020; Venero et al., 2013). Previous studies, including one from our group, have shown role of caspases and *CASP5* in CNP (Buckley et al., 2018; Joseph & Levine, 2004).

The three-arm analysis with CNP separated into two groups based on S-LANSS scores (SLANSS score 12 as a cut-off) and control group as a reference showed eight genes to be significantly associated with CNP with S-LANSS score ≥ 12. These also include *WLS*, *CHTP1*, *FGFBP2, FCRL6* and *CASP5* which are upregulated in all CNP samples as well (Supplemental Tables 5 and 6). The additional genes associated only with CNP with S-LANNS ≥ 12 are *SH2D1B*, *KIR3DL2* and *CXCR31* (Supplemental Table 6).

The genes *STAT1* and *FCRL6* were significantly upregulated in the CNP compared to the controls (Supplemental Table 5). *STAT1* is the downstream target of IFN-γ and also regulates expression of many genes that cause inflammation, survival of the cell, viability or pathogen response (Busch-Dienstfertig & González-Rodríguez, 2013; Kim et al., 2015; Tsuda et al., 2009). *STAT1* expression has been found to increase in microglia of SNL compared to sham rats (Denk et al., 2016). Also, there is growing evidence to suggest that *STAT3* activation is one of the key mediators of inflammation and CNP (Tsuda et al., 2011; Xue et al., 2014). In the present study, *STAT3* perturbation was not significant in any analysis (Supplemental Tables 5–7,). It could be possible that CNP and *STAT3* correlation could be more significant at the protein level as STAT proteins are regulated by tyrosine phosphorylation (Lim & Cao, 2006). FCRL6 is a cell surface glycoprotein which is selectively expressed by cytotoxic T-cells and natural killer (NK) cells (Rostamzadeh et al., 2018). It is upregulated in diseases characterised by chronic immune inflammation (Rostamzadeh et al., 2018) although its role in pain has not yet been validated.

*FGFBP2*, *XCL2* and *GZMA* were downregulated in CNP. These proteins are related to the immune response. FGFBP2 is a serum protein that is selectively secreted by cytotoxic lymphocytes and may be involved in cytotoxic lymphocyte-mediated immunity (Ogawa et al., 2001). *FGFBP2* downregulation has been associated with idiopathic frozen shoulders and developing ankylosing spondylitis (Fang et al., 2015; Hagiwara et al., 2012). XCL2 is a chemokine, and increased activity of chemokines is suggested to directly or indirectly contribute to CNP (Ji et al., 2014; Kwiatkowski & Mika, 2018; White et al., 2007; Zychowska et al., 2016). Interestingly, we found that the *XCL2* was downregulated in CNP compared to controls. Further investigation would be needed to determine whether the expression of *XCL2* was reflective of CNP or a response to drug.

GZMA is a serine protease present constitutively in the cytotoxic T-cells and NK cells. It acts through a caspase-independent pathway by inducing reactive oxygen species and targets infected cells as well as tumour cells (Rchiad et al., 2020; Zhou et al., 2020). GZMA has been associated with the regulation of inflammation and GZMA-deficient animal cells have shown increased levels of proinflammatory cytokines such as TNF-α (Garcia-Laorden et al., 2016).

Three additional genes, *SH2D1B*, *KIR3DL2* and *CXCR31*, significant in only CNP samples with S-LANSS score ≥ 12, are also associated with immune response. The expression of these genes was also decreased in CNP samples. In summary, the profile of differentially expressed genes suggests that CNP patients have increased inflammation and a perturbed immune system (Baddack-Werncke et al., 2017; Costigan et al., 2009). In common with many of the other diseases are several potential confounding factors that might influence the results of our study. Perhaps the most difficult to correct for is depression suggested by a high-PHQ-9 score in CNP patients (Table 1). Hence, we cannot exclude the possibility that some changes in the gene expression could be due to underlying depression. For example, along with CNP overexpression of *GSK3B* has also been widely linked to anxiety, depression, neurological and neurodegenerative disorders (Beurel et al., 2015; Chen et al., 2015; Gobrecht et al., 2014; Liu et al., 2017; Maixner & Weng, 2013; Mazzardo-Martins et al., 2012; Ronai et al., 2014), possibly through neuroinflammation (Beurel et al., 2015; Jope et al., 2007; Kremer et al., 2011).

When comparing control and CNP samples, the AUROC curve analysis that functions as a clinical risk prediction model, and showed that the combination of *MYC, STAT1, TLR4, CASP5* and *WLS* gene expression has a high sensitivity as well as specificity and thus considered a strong biomarker for CNP (Fig. 6a). These genes were robust enough to distinguish high and low S-LANSS CNP samples (Fig. 6b). All these genes are associated with PI3k-Akt pathway suggesting the involvement of this pathway in the pathogenesis of CNP.

We observed that expression of *GSK3B*, *TLR4* and *KLRB1* varies with the opioid intake (Fig. 7 and Supplemental Table 8). This may reflect the broad-spectrum analgesic actions of the opioids (Smith, 2012). It is well established that opioids can interact with TLR4 and GSK3B (Maixner & Weng, 2013; Shah & Choi, 2017). Interaction of morphine and other opioids with TLR4 has been shown to activate TLR4 pathway, resulting in the release of cytokines that can exacerbate inflammation leading to a state of hyperalgesia (Wang et al., 2012a, 2012b). In the present study, the expression of both *TLR4* and *GSK3B* were reduced whilst the expression of *KLRB1* was increased in CNP patients taking opioid medication compared to the patients that were not taking any opioid medication (Fig. 6). KLRB1 belongs to a lectin superfamily group that binds to other proteins and are calcium-dependent (Kirkham & Carlyle, 2014). Similar to GSK3B and TLR4, KLRB1 are also involved in PI3k-Akt pathway (Kirkham & Carlyle, 2014). Previous reports suggest that opioids activate TLR4 pathway of inflammation but their effect on *TLR4* expression is not clear (El-Hage et al., 2011; Shah & Choi, 2017; Wang et al., 2012a, 2012b). Franchi et al. have shown that *TLR4* mRNA expression was decreased in murine macrophages due to the activation of the Mu-opioid receptor (Franchi et al., 2012). A similar mechanism could be possible here, although there are various limitations to this conclusion; firstly, other medications can also affect *TLR4* expression. Secondly, nearly all studies investigating the association between *TLR4* and opioids have been carried out on brain cells, whereas our study used blood samples. The possibility that *TLR4* shows different tissue-specific response to the opioids cannot be ruled out. Thirdly, the number of patients with and without opioids was not equal (33 vs 17) in the CNP cohort of our study. Nevertheless, it is an interesting outcome and we aim to investigate the opioid effect on the *TLR4* expression on a larger cohort in the future.

Our study shows that *GSK3B* could be perturbed in CNP. Recent studies suggested that GSK3B levels are elevated in CNP (Maixner & Weng, 2013; Martins et al., 2011; Mazzardo-Martins et al., 2012). We observed that opioids decreased *GSK3B* expression in the patients. Previous studies have also shown that opioids like morphine can inhibit *GSK3B* in cancer cells and rat microglia (Xie et al., 2010; Zhao et al., 2009). However, effects of opioids on *GSK3B* requires further investigation for long-term implications on patients as GSK3B itself is a tightly regulated protein and master regulator of key neuronal signalling proteins (Beurel et al., 2015). We could not carry out protein analysis on the present samples as GSK3B could not be detected in the circulating plasma. However, it remains a key molecule for investigation for our future studies on CNP.

In conclusion, our results demonstrated that *MYC, STAT1, TLR4, CASP5* and *WLS* gene expression function as strong clinical risk predictors and could be purposed as a potential biomarker signature for CNP. The effects of confounding factors, i.e. medications and depression cannot be fully ruled out in patient samples. As our study has been carried out on the patients rather than animal models, it provides us with an actual outlook of these potential biomarkers that could provide insight into CNP. We aim to validate our findings and evaluate the clinical utility of the potential predictive and prognostic biomarkers identified.

## Supplementary Information

Below is the link to the electronic supplementary material.

Supplementary file1 (DOCX 1940 kb)

Supplementary file2 (DOCX 295 kb)
