# Supplementary material for: The Identification of Blood Biomarkers of Chronic Neuropathic Pain by Comparative Transcriptomics
Source: Neuromolecular Med. 2021 Nov 5;24(3):320–38. doi: 10.1007/s12017-021-08694-8 (PMC9402512; doi:10.1007/s12017-021-08694-8)
Supplement: Supplementary file 1 — Supplementary file1 (DOCX 1940 kb) [file 12017_2021_8694_MOESM1_ESM.docx]

**SUPPLEMENTARY MATERIAL**

**Questionnaires**

The S-LANSS, Chronic Pain Grade (CPG) and PHQ-9 questionnaires used in this study are presented in the following pages.

**S-LANSS Pain Questionnaire:**

**Subject code: _________**

**• This questionnaire can tell us about the type of pain that you may be experiencing. This can help in deciding how best to treat it.**

**• Please draw on the diagram below where you feel your pain. If you have pain in more than one area, only shade in the one main area where your worst pain is.**

**On the scale below, please indicate how bad your pain (that you have shown on the above diagram) has been in the last week where:'0' means no pain and '10' means pain as severe as it could be.**

**NONE 0 1 2 3 4 5 6 7 8 9 10 SEVERE PAIN**

**Subject code: _________**

**• Below are 7 questions about your pain (the one in the diagram above).**

**• Think about how your pain that you showed in the diagram has felt over the last week. Put a tick against the descriptions that best match your pain. These descriptions may, or may not, match your pain no matter how severe it feels.**

**• Only circle responses that describe your pain.**

| **1. In the area where you have pain, do you also have 'pins and needles', tingling or prickling sensations?** | |
| --- | --- |
| **a) NO - I don't get these sensations** |  |
| **b) YES - I get these sensations often** |  |
| **2. Does the painful area change colour (perhaps looks mottled or more red) when the pain is particularly bad?** | |
| **a) NO - The pain does not affect the colour of my skin** |  |
| **b) YES - I have noticed that the pain does make my skin look different from normal** |  |
| **Subject code: _________**  **3. Does your pain make the affected skin abnormally sensitive to touch? Getting unpleasant sensations or pain when lightly stroking the skin might describe this.** | |
| **a) NO - The pain does not make my skin in that area abnormally sensitive to touch** |  |
| **b) YES - My skin in that area is particularly sensitive to touch** |  |
| **4. Does your pain come on suddenly and in bursts for no apparent reason when you are completely still? Words like 'electric shocks', jumping and bursting might describe this.** | |
| **a) NO - My pain doesn't really feel like this** |  |
| **b) YES - I get these sensations often** |  |

| **5. In the area where you have pain, does your skin feel unusually hot like a burning pain?** | | |
| --- | --- | --- |
| **a) NO - I don't have burning pain** |  | |
| **b) YES - I get burning pain often** |  | |
| **6. Gently rub the painful area with your index finger and then rub a non-painful area (for example, an area of skin further away or on the opposite side from the painful area). How does this rubbing feel in the painful area?** | | |
| **a) The painful area feels no different from the non-painful area** | |  |
| **b) I feel discomfort, like pins and needles, tingling or burning in the painful area that is different from the non-painful area** |  | |
| **7. Gently press on the painful area with your finger tip then gently press in the same way onto a non-painful area (the same non-painful area that you chose in the last question). How does this feel in the painful area?** | | |
| **a) The painful area does not feel different from the non-painful area** |  | |
| **b) I feel numbness or tenderness in the painful area that is different from the non-painful area** |  | |

**Subject code: _________**

**SCORE:……………………………………………..**

**Chronic Pain Grade (CPG) questionnaire**

**Subject code: _________**

**For the following questions, please circle the number that best describes your pain**

| **1. How would you rate your pain, on a 0-10 scale, at its worst in the last 24 hours, where 0 is ‘No Pain’ and 10 is ‘Pain as bad as it could be?**    0          1          2          3          4          5          6          7          8          9          10    No pain                                                                                                          Pain as bad                                                                                                                        as it could be |
| --- |
| **2. How would you rate your pain, on a 0-10 scale, at its least in the last 24 hours?**              0          1          2          3          4          5          6          7          8          9          10    No pain                                                                                                          Pain as bad                                                                                                                      as it could be |
| **3. How would you rate your pain on a 0-10 scale at the present time, that is,   right now?**               0          1          2          3          4          5          6          7          8          9          10    No pain                                                                                                          Pain as bad                                                                                                                      as it could be |
| **4. In the past 3 months, how intense was your worst pain?**                0          1          2          3          4          5          6          7          8          9          10    No pain                                                                                                            Pain as bad                                                                                                                        as it could be |
| **5. In the past 3 months, on average, how intense was your pain?**                 0          1          2          3          4          5          6          7          8          9          10    No pain                                                                                                           Pain as bad                                                                                                                       as it could be |
| **6. About how many days in the past three months have you been kept from your usual activities (work, school or housework) because of pain?***Place a tick in the appropriate box*          0-6 days                      7-14 days                   15-30 days                 31 or more days |
| **7. In the past 3 months how much has pain interfered with your daily activities where 0 is “no interference” and 10 is “unable to carry on any activities”**                 0          1          2          3          4          5          6          7          8          9          10    No interference                                                                                          Unable to carry                                                                                                                    on any activities |
| **8. In the past 3 months how much has pain interfered with your ability to take part in recreational, social and family activities?**              0          1          2          3          4          5          6          7          8          9          10    No interference                                                                                     Unable to take part                                                                                                               in recreational or                                                                                                               social activities |
| **9. In the past three months, how much has pain interfered with your ability to work (including college, housework)?**                 0          1          2          3          4          5          6          7          8          9        10    No interference                                                                                             Extreme                                                                                                                      interference |
| **10. In the past three months, how much has pain interfered with your concentration, memory, problem solving or decision making?**    0 1 2 3 4 5 6 7 8 9 10    No interference              Extreme                                                                                                                     interference |

**Subject code: _________**

**
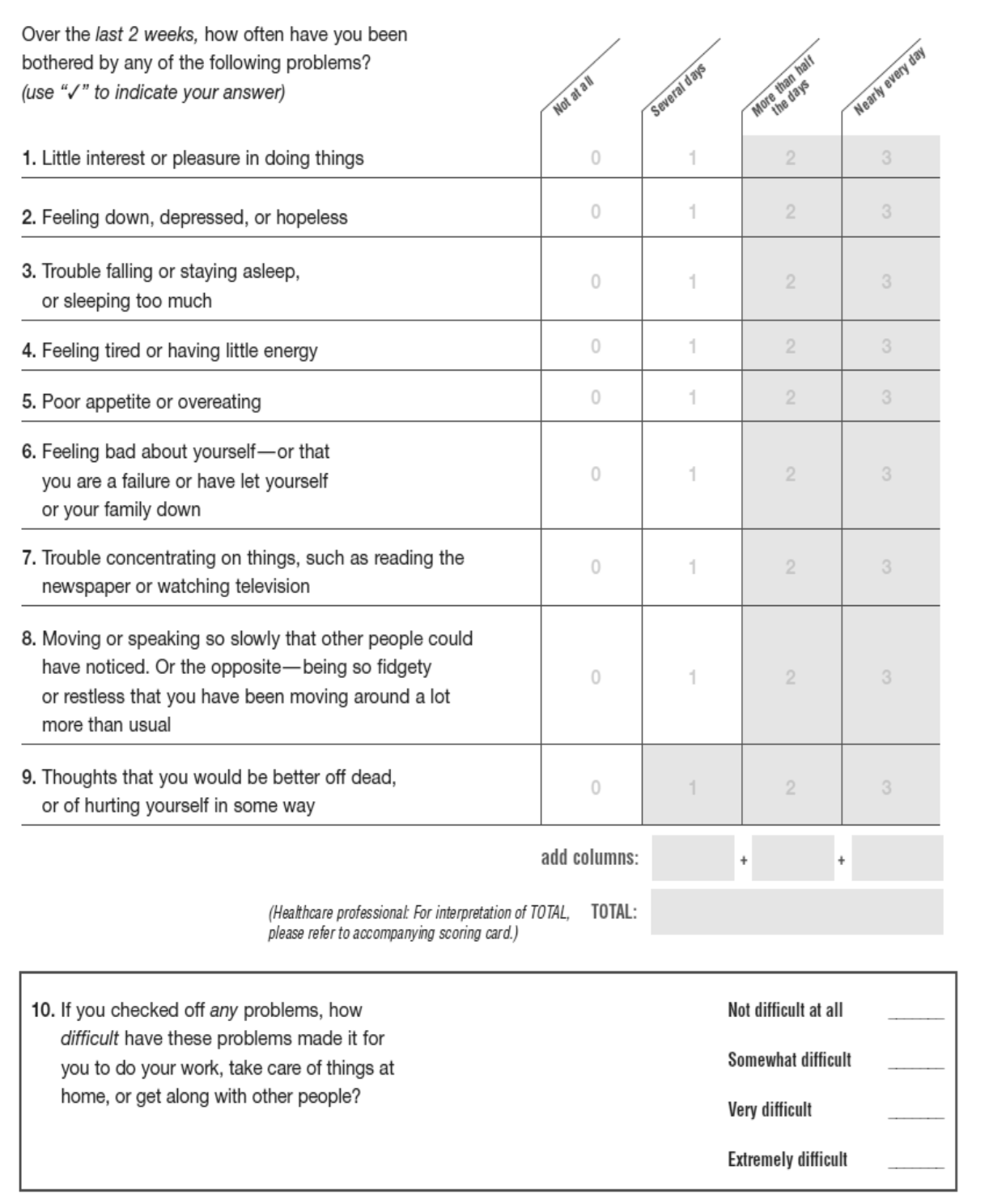
PHQ-9 questionnaire**

**Subject code: _________**

**Supplemental Figures**

**Supplemental Figure 1.** Principal component analysis (PCA) score plot of the microarray data across PCA1 and PCA2. Only the genes with a fold change < -1 and >1 and p <0.05 were used for PCA analysis.


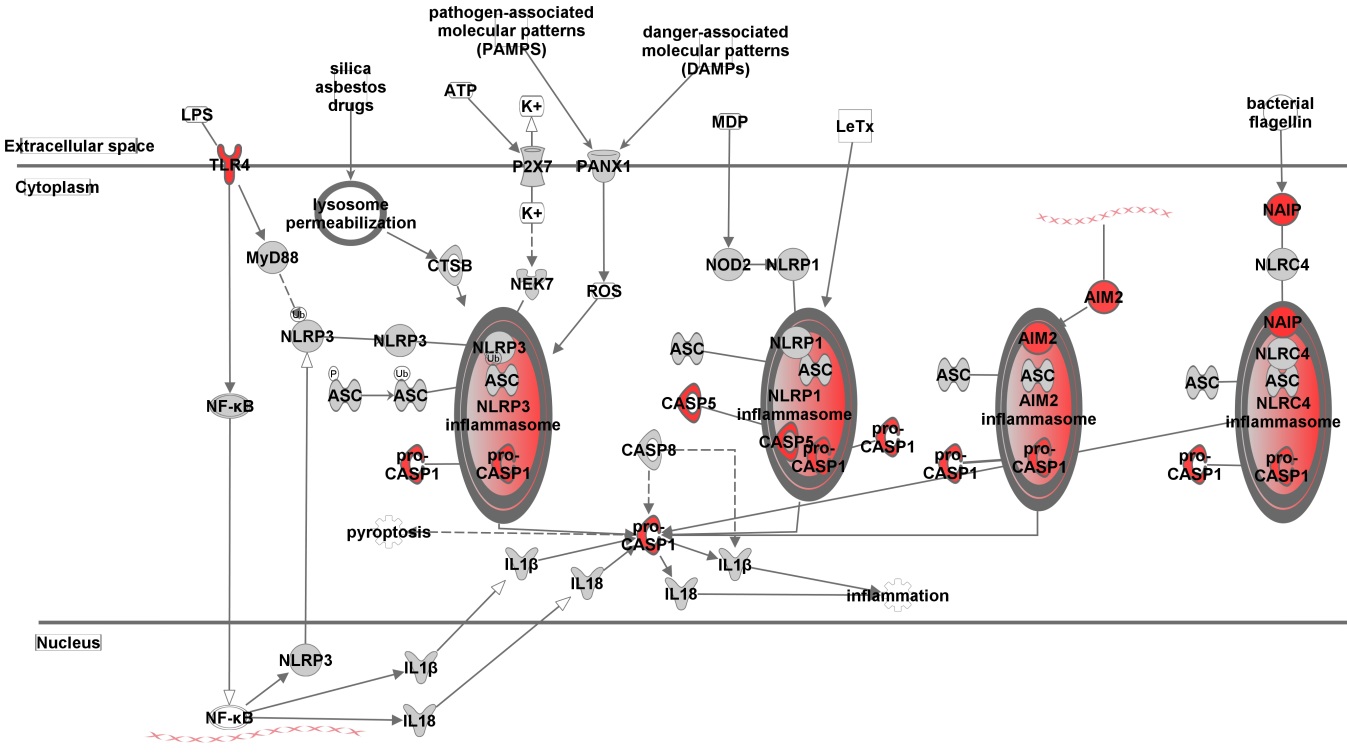


**Supplemental Figure 2.** Inflammasome pathway was identified as the top canonical pathway by IPA analysis of the chronic neuropathic pain (CNP) *vs* control (10 samples each) Affymetrix microarray differential gene expression. Red shading indicates increased expression in CCNPNP relative to the control. White and gray shading indicates non-expression and non-differential expression, respectively.


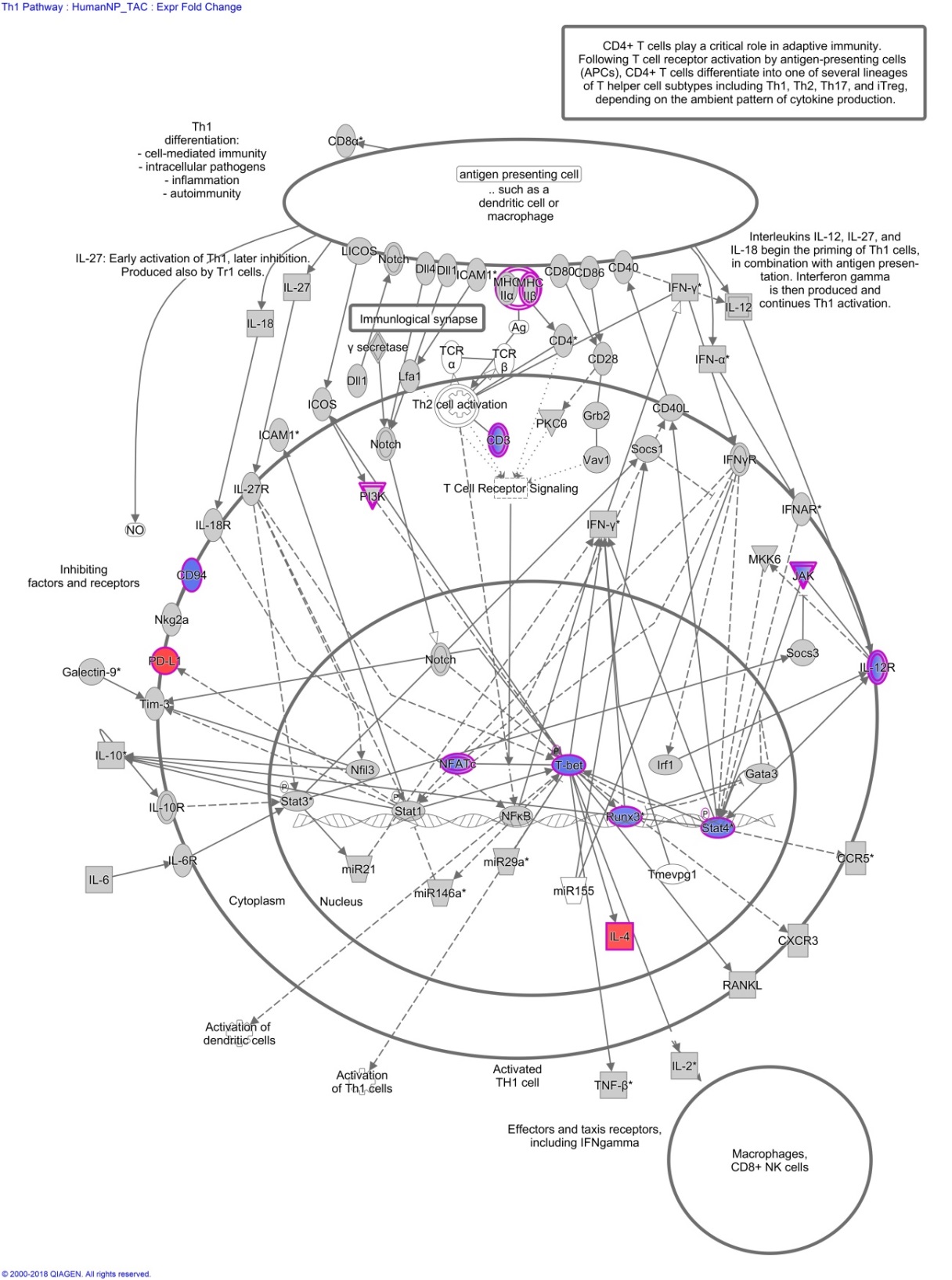


**Supplemental Figure 3**. Th1 pathway was the second most scoring canonical pathway in the IPA analysis of the CNP *vs* control (10 samples each) Affymetrix microarray differential gene expression. Red and blue shading indicate increased and decreased expression in pain relative to the control. White and gray shading indicates non-expression and non-differential expression, respectively. Magenta outline indicates that the direction of gene expression does not agree with IPA prediction. See Supplemental Figure S3 for explanation of the shapes used to represent the molecules.


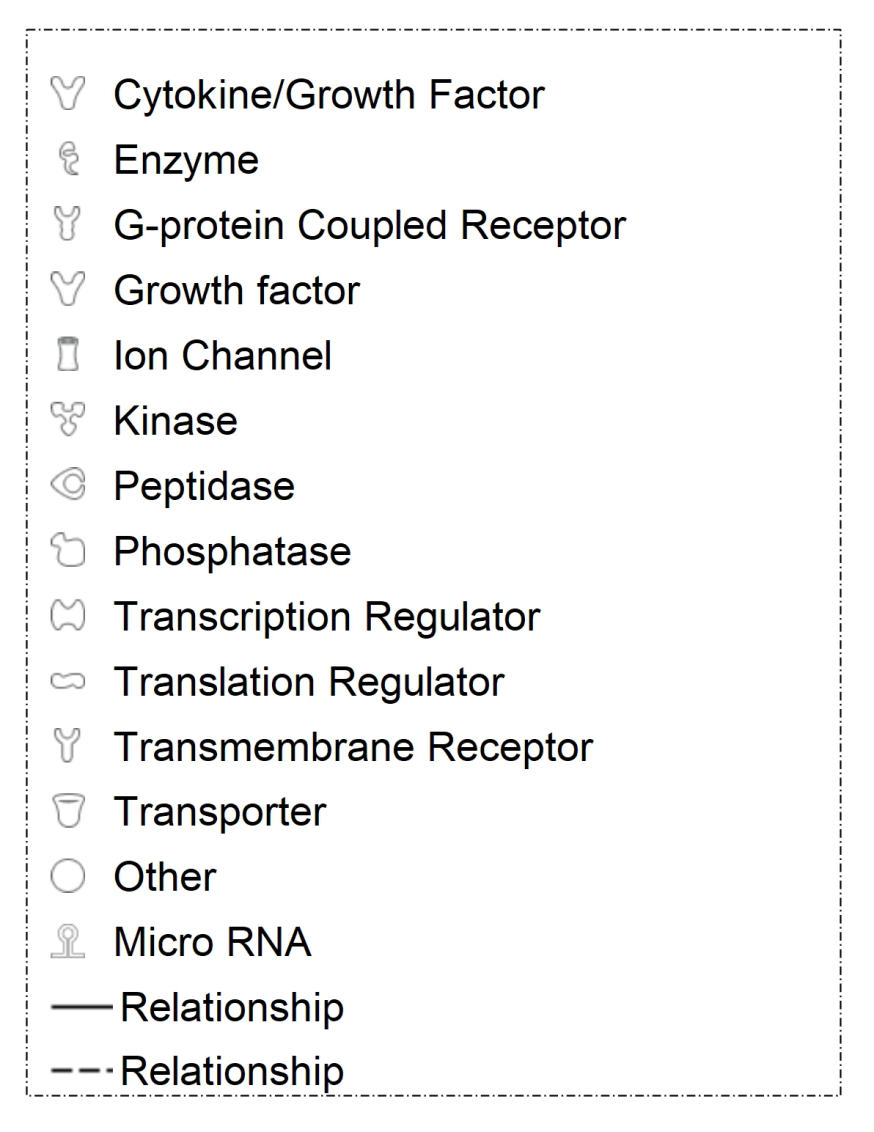


**Supplemental Figure 4**. Detailed legend to various shapes that represent the functional class of the gene product in IPA figures, Figure 4, Supplemental Figures 1 and 2.


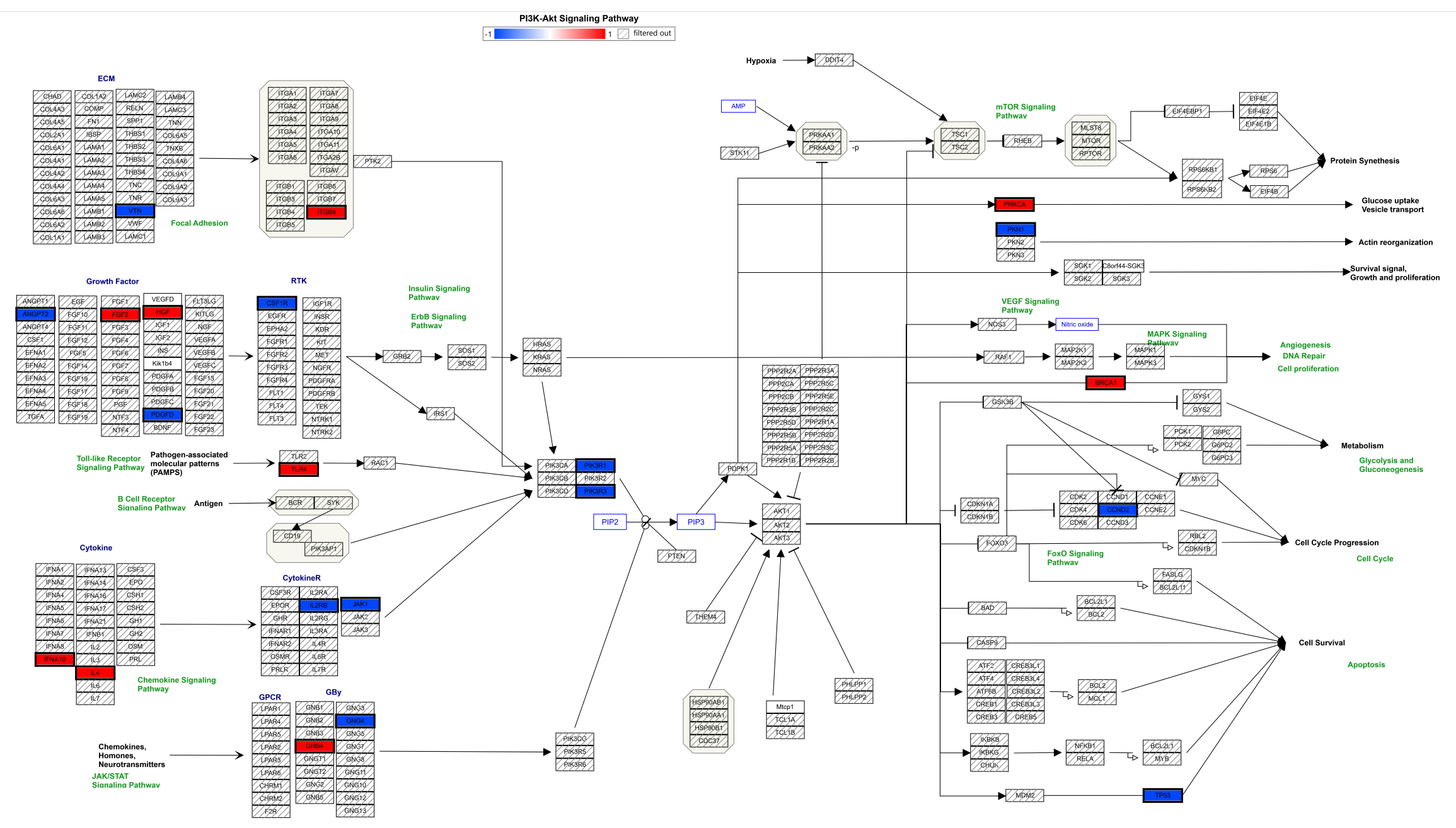


**Supplemental Figure 5.** Analysis of differential gene expression of CNP *vs* Control Affymetrix microarray data identified PI3K-Akt Signalling pathway as the most important pathway based on the number of participating genes. The upregulated and downregulated genes are shown in red and blue, respectively. The important nodes identified by IPA i.e. *GSK3B* and *MYC* are also a part of PI3K-Akt pathway.


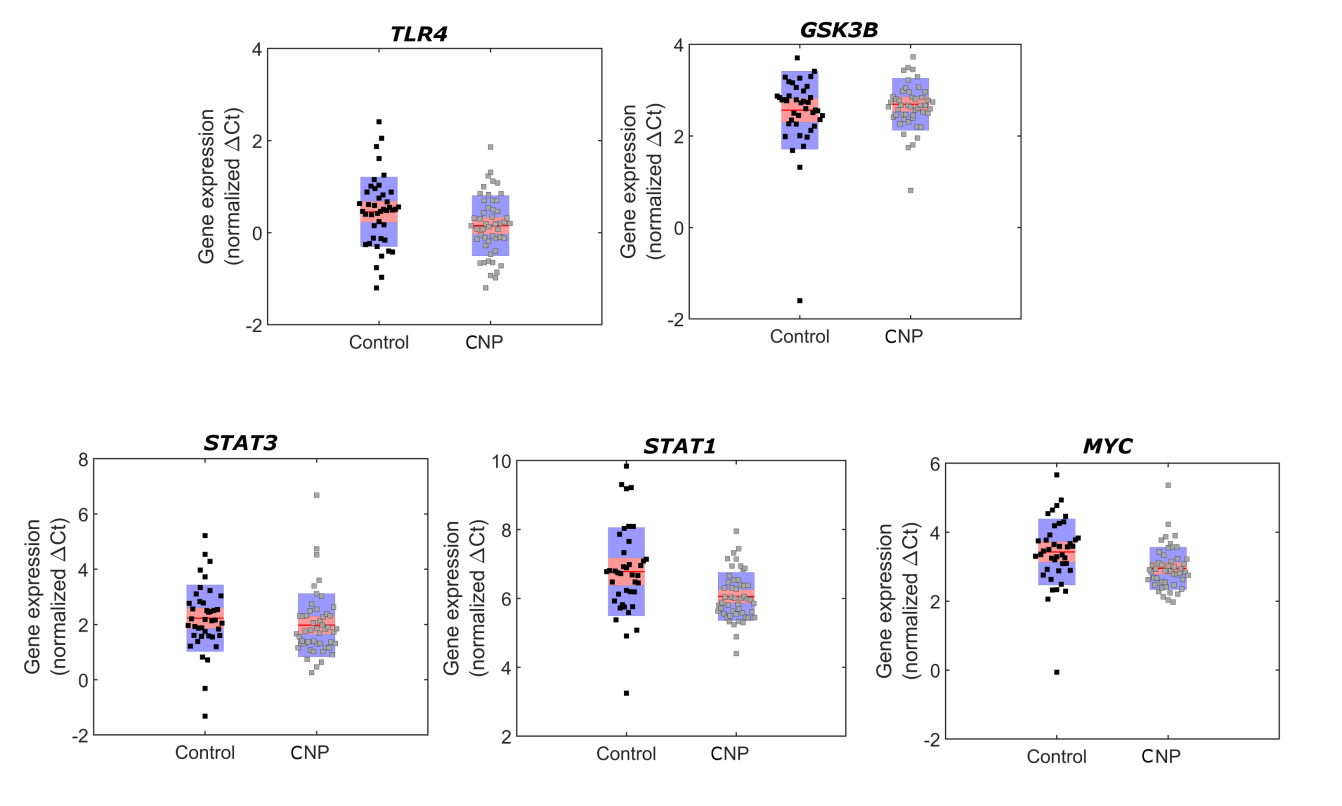


**Supplemental Figure 6.** Comparison of gene expression in the CNP vs control samples by qRT-PCR. ∆Ct is inversely related to the gene expression. The genes suggested to play a role in CNP and important transcription factors identified in the Affymetrix microarray were crossverified by qRT-PCR. In the box red line shows the mean and pink and blue area indicates values within 95% confidence interval and standard deviation 1. The markers indicate the normalized ∆Ct values of the genes in the samples.

Supplemental Tables

**Supplemental Table 1**. Characteristics of pain-free control samples used in the present study

| **CODE** | **AGE** | **GENDER**  **CODE*^b^*** | **PHQ9** | **STAI-I** | **STAI-II** |
| --- | --- | --- | --- | --- | --- |
| CHL01 | 45 | 2 | 2 | 21 | 31 |
| CHL02 | 53 | 2 | 1 | 23 | 29 |
| CHL03 | 22 | 2 | 0 | 22 | 37 |
| CHL04 | 61 | 2 | 2 | 23 | 41 |
| CHL05 | 27 | 2 | 4 | 48 | 53 |
| CHL06 | 52 | 1 | 0 | 20 | 20 |
| CHL07 | 43 | 1 | 2 | 31 | 41 |
| CHL08 | 49 | 2 | 0 | 25 | 22 |
| CHL09 | 21 | 2 | 2 | 41 | NR |
| CHL10 | 24 | 1 | 8 | 24 | 46 |
| CHL11 | 62 | 2 | 0 | 20 | 29 |
| CHL12 | 23 | 2 | 2 | 33 | 42 |
| CHL13 | 25 | 1 | 7 | 35 | 36 |
| CHL14 | 46 | 1 | 8 | 34 | 41 |
| CHL15 | 21 | 2 | 0 | 20 | 45 |
| CHL16 | 30 | 2 | 1 | 25 | 36 |
| CHL17 | 21 | 2 | 8 | 50 | 44 |
| CHL18 | 26 | 2 | 3 | 34 | 37 |
| CHL19 | 51 | 2 | 0 | 24 | 34 |
| CHL20 | 24 | 1 | 0 | 20 | 24 |
| CHL21 | 48 | 1 | 0 | 20 | 25 |
| CHL22 | 21 | 1 | 5 | 42 | 51 |
| CHL23 | 18 | 1 | 3 | 33 | NR |
| CHL24 | 51 | 2 | 1 | 32 | 30 |
| CHL25 | 23 | 2 | 1 | 20 | NR |
| CHL26 | 25 | 2 | 2 | 20 | NR |
| CHL27 | 21 | 1 | 1 | 22 | 25 |
| CHL28 | 21 | 2 | 4 | 36 | 43 |
| CHL29 | 51 | 1 | 5 | 31 | 53 |
| CHL30 | 41 | 2 | 5 | 32 | 51 |
| CHL31 | 53 | 2 | 2 | 29 | 28 |
| CHL32 | 45 | 2 | 2 | 25 | 26 |
| CHL34 | 48 | 2 | 0 | 22 | 30 |
| CHL35 | 68 | 2 | 0 | 21 | 25 |
| CHL36 | 51 | 2 | 0 | 23 | 29 |
| CHL37 | 46 | 2 | 2 | 41 | 37 |
| CHL38 | 57 | 2 | 2 | 21 | 35 |
| CHL39 | 59 | 2 | 1 | 22 | NR |
| CHL40 | 22 | 1 | 1 | 20 | 28 |
| CHL41 | 33 | 1 | 3 | 28 | 38 |
| CHL42 | 37 | 1 | 4 | 28 | 34 |
| CHL43 | 47 | 2 | 0 | 28 | NR |

*^a^* The information if not provided in the questionnaire or not assayed in the experiments is marked as NR (not recorded)

*^b^* Male and Female are coded as 1 and 2, respectively

**Supplemental Table 2.** Characteristics of CNP patients used in the study

| **CODE** | **AGE** | **GENDER**  **CODE*^b^*** | **DURATION**  **(months)** | **S-LANSS** | **PHQ9** | **CPG** | **STAI-I** | **STAI-II** | **ANTIINF*^c^*** | **ANTIDEP**^c^ | **ANTICONV*^c^*** | **OPIOIDS*^c^*** |
| --- | --- | --- | --- | --- | --- | --- | --- | --- | --- | --- | --- | --- |
| NPL001 | 68 | 1 | 24 | 0 | 4 | II | 44 | NR | 0 | 0 | 1 | 0 |
| NPL002 | 64 | 1 | 36 | 17 | 6 | II | 23 | NR | 1 | 1 | 0 | 0 |
| NPL003 | 40 | 2 | 84 | 16 | 13 | II | 45 | NR | 0 | 1 | 1 | 0 |
| NPL004 | 38 | 2 | 36 | 23 | 10 | IV | 36 | NR | 0 | 1 | 1 | 0 |
| NPL005 | 48 | 2 | 108 | 18 | 11 | IV | 42 | NR | 0 | 1 | 0 | 0 |
| NPL006 | 49 | 1 | 60 | 17 | 14 | II | 39 | NR | 1 | 1 | 1 | 0 |
| NPL007 | 21 | 1 | 18 | 13 | 12 | III | 56 | NR | 0 | 0 | 0 | 1 |
| NPL008 | 68 | 2 | 120 | 19 | 7 | III | 39 | NR | 0 | 0 | 1 | 1 |
| NPL009 | 56 | 1 | 96 | 5 | 3 | I | 26 | NR | 1 | 0 | 1 | 0 |
| NPL010 | 40 | 2 | 38 | 22 | 25 | IV | 67 | NR | 0 | 0 | 0 | 0 |
| NPL011 | 60 | 1 | 228 | 10 | 6 | III | 49 | NR | 1 | 0 | 0 | 0 |
| NPL012 | 44 | 2 | 24 | 17 | 16 | IV | 43 | NR | 1 | 0 | 1 | 0 |
| NPL013 | 42 | 2 | 24 | 6 | 12 | IV | 60 | NR | 0 | 0 | 0 | 0 |
| NPL014 | 49 | 2 | 120 | 4 | 8 | IV | 63 | NR | 0 | 0 | 0 | 0 |
| NPL015 | 59 | 2 | 132 | 19 | 18 | IV | 32 | NR | 0 | 0 | 0 | 0 |
| NPL016 | 40 | 1 | 168 | 24 | 19 | III | 71 | NR | 0 | 1 | 0 | 0 |
| NPL017 | 74 | 1 | 588 | 0 | 6 | IV | 26 | NR | 1 | 1 | 0 | 0 |
| NPL018 | 40 | 2 | 192 | 23 | 16 | IV | 53 | NR | 0 | 1 | 0 | 1 |
| NPL019 | 36 | 2 | 84 | 24 | 18 | IV | 58 | NR | 1 | 0 | 0 | 0 |
| NPL020 | 37 | 1 | 42 | 18 | 14 | IV | 45 | NR | 0 | 0 | 0 | 0 |
| NPL021 | 41 | 1 | 24 | 12 | 19 | III | 64 | NR | 0 | 1 | 1 | 0 |
| NPL022 | 48 | 1 | 18 | 24 | 21 | IV | 55 | NR | 1 | 0 | 0 | 0 |
| NPL023 | 43 | 2 | 36 | 9 | 20 | IV | 69 | NR | 0 | 0 | 0 | 1 |
| NPL024 | 50 | 2 | 13 | 5 | 1 | I | 21 | NR | 1 | 0 | 0 | 1 |
| NPL025 | 37 | 2 | 36 | 2 | 20 | IV | 56 | NR | 1 | 0 | 0 | 1 |
| NPL026 | 61 | 1 | 240 | 24 | 23 | IV | 27 | NR | 1 | 1 | 1 | 1 |
| NPL027 | 51 | 1 | 48 | 24 | 25 | IV | 40 | NR | 0 | 0 | 1 | 1 |
| NPL028 | 39 | 1 | 96 | 15 | 21 | III | 48 | NR | 0 | 0 | 0 | 1 |
| NPL029 | 43 | 2 | 84 | 18 | 3 | II | 27 | 26 | 1 | 1 | 0 | 1 |
| NPL030 | 49 | 1 | 72 | 24 | 2 | II | 23 | 31 | 0 | 1 | 0 | 1 |
| NPL031 | 48 | 2 | 42 | 19 | 12 | IV | 45 | 49 | 1 | 1 | 1 | 1 |
| NPL032 | 68 | 2 | 24 | 22 | 8 | IV | 31 | 24 | 0 | 1 | 0 | 1 |
| NPL033 | 34 | 1 | 204 | 19 | 20 | IV | 61 | 57 | 0 | 1 | 1 | 1 |
| NPL034 | 24 | 2 | 36 | 19 | 17 | IV | 51 | 64 | 1 | 0 | 1 | 1 |
| NPL037 | 45 | 2 | 180 | 19 | 21 | III | 65 | 60 | 0 | 0 | 0 | 1 |
| NPL038 | 51 | 2 | 156 | 18 | 14 | IV | 66 | 67 | 1 | 0 | 1 | 1 |
| NPL039 | 48 | 2 | 132 | 16 | 14 | I | 47 | 51 | 0 | 0 | 1 | 1 |
| NPL040 | 45 | 1 | 18 | 3 |  | NR | 57 | 54 | 0 | 0 | 1 | 1 |
| NPL041 | 33 | 1 | 96 | 24 | 11 | III | 48 | 43 | 1 | 1 | 1 | 1 |
| NPL042 | 43 | 1 | 72 | 17 | 17 | IV | 38 | 36 | 1 | 0 | 1 | 1 |
| NPL043 | 38 | 2 | 180 | 24 | 9 | III | 54 | 49 | 1 | 0 | 0 | 1 |
| NPL044 | 37 | 2 | 54 | 2 | 21 | III | 50 | 61 | 0 | 1 | 1 | 1 |
| NPL045 | 47 | 2 | 108 | 14 | 11 | IV | 40 | 45 | 1 | 1 | 1 | 1 |
| NPL046 | 54 | 1 | 24 | 0 | 0 | II | 37 | 41 | 0 | 1 | 1 | 1 |
| NPL047 | 49 | 1 | 18 | 3 | 3 | I | 32 | 34 | 1 | 0 | 0 | 1 |
| NPL048 | 21 | 2 | 11 | 24 | 21 | IV | 37 | 60 | 1 | 1 | 0 | 1 |
| NPL049 | 79 | 1 | 48 | 7 | 3 | II | 46 | 42 | 1 | 0 | 0 | 1 |
| NPL050 | 50 | 2 | 300 | 24 | 24 | IV | 59 | 58 | 1 | 0 | 1 | 1 |
| NPL052 | 29 | 2 | 30 | 24 | 26 | IV | 51 | 71 | 1 | 1 | 1 | 1 |
| NPL053 | 44 | 1 | 204 | 15 | 4 | IV | 33 | 38 | 0 | 0 | 1 | 1 |
|  |  |  |  |  |  |  |  |  |  |  |  |  |

*^a^* The information if not provided in the questionnaire or not assayed in the experiments is marked as NR (not recorded); Pain duration and CPG scores were irrelevant for control samples

*^b^* Male and Female are coded as 1 and 2, respectively

*^c^*Intake of anti-inflammatory, anticonvulsant and antidepressant drugs were recorded as binary variables under the column ANTIINF, ANTICONV and ANTIDEP, respectively. Drug if taken is marked as 1 or else 0.

**Supplemental Table 3.** (see separate file)

**Supplemental Table 4.** Primers used for qRT-PCR

| **Gene** | **Forward 5' -> 3'** | **Reverse 5' -> 3'** |
| --- | --- | --- |
|  |  |  |
| ***MS4A2*** | CCAAGTGCTTTATGGCTTCCT | GAGTTCTTCCCCAGCTCCA |
| ***CHPT1*** | AGCTCTTTGACCATGGCTGT | TAAGTTCCTAAGCGAGCGGC |
| ***WLS*** | CCTTACGCCCAGCATCTTCA | CCCTGTCGGATGTCACCAAA |
| ***AMMECR1 [S***[***1***](#_ENREF_1)***]*** | TGTATGGATACCAGCAGCCC | GCAGCTCATCCCTTGTCATT |
| ***TRIM51EP*** | ATCACAGACACTGTCCCATTG | GGTGGTTTCTGTGTTCAGGT |
| ***NAIP*** | AGTCTCAGGGACAATCCAG | TCTTACAGGAAACAAGCATAGTC |
| ***FCER1A*** | GAGAGTGAACCTGTGTACCT | ACTTGAGAGCTTCACCATCC |
| ***TNFAIP6*** | CTACAACCCACACGCAAAG | GTCTAATGTGCCAGTAGCAG |
| ***CASP5*** | ACTTTGTCCTCGTGAAGAATTCC | CGTCTGCGGTCCTCTCTC |
| ***TARP*** | AGCTCACAAACACCTCTGCA | AGCCGTTCTTCTAAGCAGACA |
| ***KLRB1*** | CTTACCCACAGACTCAGGCC | GCACAGCTAAGTTTCAGGGC |
| ***SH2D1B*** | ACCAAGCAAGACTGTGAGAC | CTCTGAAGATTCGGTATGTGTAGAC |
| ***KIR3DL2*** | TAATGGACCAAGAGCCTGC | GGCGACTGATTTTTCTCTGTATG |
| ***FCRL6*** | GGCAGGTGATGTATATTCCACA | TCAGGGTCACCAGGCTAC |
| ***CX3CR1*** | GAGGCGTTTAAGTTGGCAGA | AAAGACCACGATGTCCCCAAT |
| ***FGFBP2*** | GCTGCTGACCCCAAACCTTA | ACACGGATGGCCTAAGCAC |
| ***GZMA*** | CCCTACATGGTCCTACTTAGTC | TCCCTGGTTATTGAGTGAGC |
| ***XCL2*** | CCAGCGACTGCCAGTTAG | CATGCTCCTGACCACGTC |
| ***PHB1*** | ACCACGTAATGTGCCAGTCA | TGAAGATGCGAGGAAGCTG |
| ***PHB2*** | AAGAGTGTGGTGGCCAAGTT | AAGTCCTTGGCCCCTCTCTGT |
| ***GCHFR*** | TCTGCCTTGCTCCTCTCTTC | CCCTCTCCCACTGCTTGAC |
| ***TLR4*** | CCAAGAACCTGGACCTGAGC | AGGCTCTGATATGCCCCATC |
| ***STAT3*** | GGGAGAGATTGACCAGCAGTAT | TGGCTTCTCAAGATACCTGCTC |
| ***STAT1*** | PRIMER DESIGN REFERENCE GENE/GENORM 600rx | |
| ***GSK3B*** | CTGTGTGTTGGCTGAGCTGT | TTTGCTCCCTTGTTGGAGTT |
| ***MYC*** | TACATCCTGTCCGTCCAAGC | CCTTACGCACAAGAGTTCCG |
| ***GAPDH*** | PRIMER DESIGN REFERENCE GENE/GENORM 600rx | |
| ***SDHA*** | PRIMER DESIGN REFERENCE GENE/GENORM 600rx | |

**Supplemental Table 5.** Ordered *p*-values and inferences of significance derived from CNP (controlling for age and gender): all samples included

| Gene*^1^* | p-value | Rank (i) | B-H critical value | Significance | | |
| --- | --- | --- | --- | --- | --- | --- |
|  |  |  |  | Uncorrected | FWER-corrected*^2^* | FDR-corrected*^3^* |
| ***WLS*** | 4.80 x 10^-7^ | 1 | 0.00217 | Yes | Yes | **Yes** |
| ***CHTP1*** | 7.74 x10^-7^ | 2 | 0.00435 | Yes | Yes | **Yes** |
| ***CASP5*** | 2.30 x 10^-5^ | 3 | 0.00652 | Yes | Yes | **Yes** |
| ***FGFBP2*** | 0.00162 | 4 | 0.00870 | Yes | Yes | **Yes** |
| ***STAT1*** | 0.00223 | 5 | 0.0109 | Yes | No | **Yes** |
| ***FCRL6*** | 0.00335 | 6 | 0.0130 | Yes | No | **Yes** |
| ***MYC*** | 0.00335 | 7 | 0.0152 | Yes | No | **Yes** |
| ***XCL2*** | 0.0144 | 8 | 0.0174 | Yes | No | **Yes** |
| ***GZMA*** | 0.0168 | 9 | 0.0196 | Yes | No | **Yes** |
| ***KIR3DL2*** | 0.0368 | 10 | 0.0217 | Yes | No | No |
| ***TLR4*** | 0.0368 | 11 | 0.0239 | Yes | No | No |
| ***SH2D1B*** | 0.0417 | 12 | 0.0261 | Yes | No | No |
| ***TARP*** | 0.0740 | 13 | 0.0283 | No | No | No |
| ***CXCR31*** | 0.0978 | 14 | 0.0304 | No | No | No |
| ***FCER1A*** | 0.297 | 15 | 0.0326 | No | No | No |
| ***STAT3*** | 0.335 | 16 | 0.0348 | No | No | No |
| ***TNFAIP*** | 0.446 | 17 | 0.0370 | No | No | No |
| ***GSK3B*** | 0.491 | 18 | 0.0391 | No | No | No |
| ***KLRB1*** | 0.588 | 29 | 0.0413 | No | No | No |
| ***NAIP*** | 0.600 | 20 | 0.0435 | No | No | No |
| ***TRIM51EP*** | 0.656 | 21 | 0.0457 | No | No | No |
| ***AMMECR1*** | 0.745 | 22 | 0.0478 | No | No | No |
| ***MS4A2*** | 0.824 | 23 | 0.0500 | No | No | No |

*^1^Genes with p-values under an FDR of 5% and tested against Benjamini-Hochberg (B-H) critical values are highlighted in yellow*

*^2^Bonferroni corrected*

*^3^Assuming an FDR of 5%*

**Supplemental Table 6.** Ordered p-values, Benjamini-Hochberg (B-H) critical values and inferences of significance derived from CNP samples with S-LANSS score ≥12 (controlling for age and gender): all patients

| Gene*^1^* | p-value | B-H critical value | Significance | | |
| --- | --- | --- | --- | --- | --- |
|  |  |  | Uncorrected | FWER-corrected*^2^* | FDR-corrected*^3^* |
| ***WLS*** | 8.40 x 10^-5^ | 0.00217 | Yes | Yes | **Yes** |
| ***CHTP1*** | 7.89 x 10^-4^ | 0.00435 | Yes | Yes | **Yes** |
| ***FGFBP2*** | 8.70 x 10^-4^ | 0.00652 | Yes | Yes | **Yes** |
| ***FCRL6*** | 0.00199 | 0.00870 | Yes | Yes | **Yes** |
| ***SH2D1B*** | 0.00295 | 0.0109 | Yes | No | **Yes** |
| ***CASP5*** | 0.00393 | 0.0130 | Yes | No | **Yes** |
| ***KIR3DL2*** | 0.00838 | 0.0152 | Yes | No | **Yes** |
| ***CXCR31*** | 0.0136 | 0.0174 | Yes | No | **Yes** |
| ***STAT1*** | 0.0290 | 0.0196 | Yes | No | No |
| ***GZMA*** | 0.0628 | 0.0217 | No | No | No |
| ***TARP*** | 0.117 | 0.0239 | No | No | No |
| ***GSK3B*** | 0.151 | 0.0261 | No | No | No |
| ***MYC*** | 0.155 | 0.0283 | No | No | No |
| ***KLRB1*** | 0.506 | 0.0304 | No | No | No |
| ***AMMECR1*** | 0.513 | 0.0326 | No | No | No |
| ***MS4A2*** | 0.543 | 0.0348 | No | No | No |
| ***XCL2*** | 0.556 | 0.0370 | No | No | No |
| ***FCER1A*** | 0.585 | 0.0391 | No | No | No |
| ***TLR4*** | 0.692 | 0.0413 | Mo | No | No |
| ***NAIP*** | 0.843 | 0.0435 | No | No | No |
| ***STAT3*** | 0.928 | 0.0457 | No | No | No |
| ***TNFAIP*** | 0.951 | 0.0478 | No | No | No |
| ***TRIM51EP*** | 0.949 | 0.0500 | No | No | No |

*^1^Genes with p-values under an FDR of 5% and tested against Benjamini-Hochberg (B-H) critical values are highlighted in yellow*

*^2^Bonferroni corrected*

*^3^Assuming an FDR of 5%*

**Supplemental Table 7.** Ordered p-values, Benjamini-Hochberg (B-H) critical values and inferences of significance derived from CNP samples with S-LANSS score <12 (controlling for age and gender): all patients

| Gene | p-value | B-H critical value^1^ | Significance | | |
| --- | --- | --- | --- | --- | --- |
|  |  |  | Uncorrected | FWER-corrected*^1^* | FDR-corrected*^2^* |
| ***TLR4*** | 0.0184 | 0.00217 | Yes | No | No |
| ***MYC*** | 0.0483 | 0.00435 | Yes | No | No |
| ***CHTP1*** | 0.0726 | 0.00652 | Yes | No | No |
| ***CASP5*** | 0.111 | 0.00870 | No | No | No |
| ***STAT3*** | 0.140 | 0.0109 | No | No | No |
| ***SH2D1B*** | 0.162 | 0.0130 | No | No | No |
| ***CXCR31*** | 0.238 | 0.0152 | No | No | No |
| ***WLS*** | 0.250 | 0.0174 | No | No | No |
| ***TNFAIP*** | 0.246 | 0.0196 | No | No | No |
| ***GSK3B*** | 0.270 | 0.0217 | No | No | No |
| ***STAT1*** | 0.312 | 0.0239 | No | No | No |
| ***KIR3DL2*** | 0.396 | 0.0261 | No | No | No |
| ***TRIM51EP*** | 0.467 | 0.0283 | No | No | No |
| ***FCER1A*** | 0.502 | 0.0304 | No | No | No |
| ***GZMA*** | 0.520 | 0.0326 | No | No | No |
| ***MS4A2*** | 0.573 | 0.0348 | No | No | No |
| ***AMMECR1*** | 0.627 | 0.0370 | No | No | No |
| ***XCL2*** | 0.647 | 0.0391 | No | No | No |
| ***NAIP*** | 0.654 | 0.0413 | No | No | No |
| ***FGFBP2*** | 0.722 | 0.0435 | No | No | No |
| ***FCRL6*** | 0.745 | 0.0457 | No | No | No |
| ***TARP*** | 0.813 | 0.0478 | No | No | No |
| ***KLRB1*** | 0.841 | 0.0500 | No | No | No |

*^1^Bonferroni corrected*

*^2^Assuming an FDR of 5%*

**Supplemental Table 8.** Ordered *p*-values and inferences of significance derived from opioid analgesics (controlling for gender, age): CNP patients only

| Gene*^1^* | p-value | B-H critical value^1^ | Significance | | |
| --- | --- | --- | --- | --- | --- |
|  |  |  | Uncorrected | FWER-corrected*^2^* | FDR-corrected*^3^* |
| ***TLR4*** | 8.58 x 10^-4^ | 0.00217 | Yes | Yes | **Yes** |
| ***GSK3B*** | 0.00176 | 0.00435 | Yes | Yes | **Yes** |
| ***KLRB1*** | 0.00529 | 0.00652 | Yes | No | **Yes** |
| ***CHTP1*** | 0.00895 | 0.00870 | Yes | No | No |
| ***MS4A2*** | 0.0298 | 0.0109 | Yes | No | No |
| ***GZMA*** | 0.0352 | 0.0130 | Yes | No | No |
| ***MYC*** | 0.0731 | 0.0152 | No | No | No |
| ***TRIM51EP*** | 0.0763 | 0.0174 | No | No | No |
| ***STAT1*** | 0.0966 | 0.0196 | No | No | No |
| ***STAT3*** | 0.133 | 0.0217 | No | No | No |
| ***CXCR31*** | 0.185 | 0.0239 | No | No | No |
| ***NAIP*** | 0.205 | 0.0261 | No | No | No |
| ***WLS*** | 0.227 | 0.0283 | No | No | No |
| ***FCER1A*** | 0.341 | 0.0304 | No | No | No |
| ***AMMECR1*** | 0.390 | 0.0326 | No | No | No |
| ***FGFBP2*** | 0.415 | 0.0348 | No | No | No |
| ***FCRL6*** | 0.546 | 0.0370 | No | No | No |
| ***SH2D1B*** | 0.633 | 0.0391 | No | No | No |
| ***CASP5*** | 0.666 | 0.0413 | No | No | No |
| ***TNFAIP*** | 0.709 | 0.0435 | No | No | No |
| ***XCL2*** | 0.727 | 0.0457 | No | No | No |
| ***TARP*** | 0.732 | 0.0478 | No | No | No |
| ***KIR3DL2*** | 0.928 | 0.0500 | No | No | No |

*^1^Genes with p-values under an FDR of 5% and tested against Benjamini-Hochberg (B-H) critical values are highlighted in yellow*

*^2^Bonferroni corrected*

*^3^Assuming an FDR of 5%*

Supplementary Reference:

[S1] Zeisel A, Yitzhaky A, Bossel Ben-Moshe N, Domany E. An accessible database for mouse and human whole transcriptome qPCR primers. Bioinformatics 2013;29(10):1355-1356.
