# Supplementary material for: The Identification of Blood Biomarkers of Chronic Neuropathic Pain by Comparative Transcriptomics
Source: Neuromolecular Med. 2021 Nov 5;24(3):320–38. doi: 10.1007/s12017-021-08694-8 (PMC9402512; doi:10.1007/s12017-021-08694-8)
Supplement: Supplementary file 2 — Supplementary file2 (DOCX 295 kb) [file 12017_2021_8694_MOESM2_ESM.docx]

| **Transcript ID** | **Public Gene IDs** | **Gene Symbol** | **Gene name** | **NP average signal (log2)** | **Control average signal (log2)** | **Expression Fold Change** | **Expression p-val** |
| --- | --- | --- | --- | --- | --- | --- | --- |
| TC11002008.hg.1 | NM_001039656; NM_004923; ENST00000255087; ENST00000443940; ENST00000543240; ENST00000544963; AK074817; BC027593; BC064579; BC070301; uc001ooc.3; uc001ood.1; uc001ooe.3; uc009ysi.1 | *MTL5* | metallothionein-like 5, testis-specific (tesmin) | 3.68 | 3.53 | 1.11 | 3.86E-05 |
| TC22001113.hg.1 | NR_040535 | *LOC100506472* | uncharacterized LOC100506472 | 4.09 | 3.95 | 1.11 | 0.0002 |
| TC04001344.hg.1 | NM_001130715; NM_001130716; NM_016619; ENST00000311507; ENST00000411416; ENST00000426923; ENST00000505406; ENST00000509973; ENST00000515389; BC012205; EU570985; EU570986; OTTHUMT00000252637; OTTHUMT00000363077; OTTHUMT00000363078; OTTHUMT00000363079; OTTHUMT00000363080; OTTHUMT00000363081; uc003hod.2; uc003hoe.3; uc010ijy.3; uc010ijz.3; uc011cco.2 | *PLAC8* | placenta specific 8 | 7.44 | 7.77 | -1.26 | 0.0004 |
| TC08000207.hg.1 | NM_004103; NM_173174; NM_173175; NM_173176; ENST00000346049; ENST00000397497; ENST00000397501; ENST00000420218; ENST00000517339; ENST00000519650; AK128371; AY429564; BC036651; BC042599; OTTHUMT00000219916; OTTHUMT00000255056; OTTHUMT00000259137; OTTHUMT00000259138; OTTHUMT00000259139; OTTHUMT00000259140; OTTHUMT00000259141; OTTHUMT00000259142; OTTHUMT00000376113; OTTHUMT00000376114; OTTHUMT00000376115; OTTHUMT00000376116; OTTHUMT00000376117; OTTHUMT00000376118; OTTHUMT00000376119; OTTHUMT00000378060; uc003xfn.2; uc003xfp.2; uc003xfq.2; uc003xfr.1; uc003xfs.1; uc010luq.1; uc022ate.1 | *PTK2B* | protein tyrosine kinase 2 beta | 7.36 | 7.26 | 1.07 | 0.0005 |
| TC11003034.hg.1 | AL833496 | *TAF10* | TAF10 RNA polymerase II, TATA box binding protein (TBP)-associated factor, 30kDa | 6.02 | 5.87 | 1.11 | 0.0005 |
| TC01003490.hg.1 | NM_003175; ENST00000367819; BC069360; BC070308; OTTHUMT00000083613; uc001gfn.4 | *XCL2* | chemokine (C motif) ligand 2 | 5.26 | 5.71 | -1.37 | 0.0005 |
| TC01002507.hg.1 | NM_032881; ENST00000315732; ENST00000476041; BC007623; OTTHUMT00000022294; OTTHUMT00000022295; OTTHUMT00000022296; uc001cao.1 | *LSM10* | LSM10, U7 small nuclear RNA associated | 5.67 | 5.57 | 1.08 | 0.0006 |
| TC01001012.hg.1 | NM_001172411; NM_001172412; NM_138959; ENST00000310260; ENST00000355485; ENST00000369509; ENST00000369510; ENST00000474344; BC065272; OTTHUMT00000033094; OTTHUMT00000033095; OTTHUMT00000033096; OTTHUMT00000033097; OTTHUMT00000033098; uc001efv.1; uc009wgy.1; uc021ose.1 | *VANGL1* | VANGL planar cell polarity protein 1 | 4.56 | 4.7 | -1.1 | 0.0006 |
| TC11000950.hg.1 | NM_020802; ENST00000263468; AK095004; AK296350; AK310418 | *CEP126* | centrosomal protein 126kDa | 3.81 | 3.99 | -1.14 | 0.0007 |
| TC03000538.hg.1 | NR_028301; NR_028302; ENST00000466734 | *DUBR* | DPPA2 upstream binding RNA | 3.04 | 3.17 | -1.1 | 0.0007 |
| TC22001331.hg.1 | NR_033531 | *IFT27* | intraflagellar transport 27 | 4.08 | 4.29 | -1.16 | 0.0007 |
| TC12003069.hg.1 | NR_037629 | *LOC728739* | programmed cell death 2 pseudogene | 3.25 | 3.38 | -1.09 | 0.0007 |
| TC20000208.hg.1 | NM_015352; NM_172236; ENST00000375730; ENST00000375749; ENST00000408740; ENST00000434904; ENST00000486717; AK300810; AK303068; BC000582; OTTHUMT00000078610; OTTHUMT00000078611; OTTHUMT00000078613; OTTHUMT00000078614; uc002wxo.3; uc002wxp.3; uc010ztt.2; uc010ztu.2 | *POFUT1; MIR1825* | protein O-fucosyltransferase 1; microRNA 1825 | 4.97 | 5.07 | -1.07 | 0.0007 |
| TC0X001087.hg.1 | NM_019003; ENST00000374906; ENST00000374908; BC071694; OTTHUMT00000058915; OTTHUMT00000058916; uc004dvb.3 | *SPIN2A* | spindlin family, member 2A | 3.5 | 3.63 | -1.1 | 0.0007 |
| TC11002880.hg.1 | ENST00000539685; ENST00000540545; ENST00000543182 | *LOC105369507* | uncharacterized LOC105369507 | 1.27 | 1.16 | 1.08 | 0.0008 |
| TC04001347.hg.1 | NM_001098540; NM_001166498; NM_001199830; NM_006665; ENST00000311412; ENST00000405413; ENST00000512196; ENST00000513463; BC051321; FJ517659; FJ517660; GQ337902; HM187643; OTTHUMT00000252812; OTTHUMT00000252813; OTTHUMT00000363967; OTTHUMT00000363968; OTTHUMT00000363969; OTTHUMT00000363970; OTTHUMT00000363971; uc003hoi.3; uc003hoj.4; uc003hok.4; uc011ccq.2; uc011ccr.2; uc011ccs.2; uc011cct.2; uc021xpr.1 | *HPSE* | heparanase | 5.91 | 5.7 | 1.16 | 0.0009 |
| TC05001431.hg.1 | AK128486 | *LOC101928885* | uncharacterized LOC101928885 | 2.88 | 2.81 | 1.05 | 0.0009 |
| TC02004999.hg.1 | NM_001178015; NM_001178016; NM_022058; ENST00000272716; ENST00000375514; ENST00000415876; ENST00000421911; ENST00000446997; ENST00000493021; AK294767; AK308529; BC136269; BC143714; OTTHUMT00000333087; OTTHUMT00000333088; OTTHUMT00000333089; OTTHUMT00000333090; OTTHUMT00000333091; OTTHUMT00000333092; OTTHUMT00000338546; OTTHUMT00000338547; uc002ubx.4; uc002uby.4; uc010fpa.1; uc010zcr.1; uc010zcs.2 | *SLC4A10* | solute carrier family 4, sodium bicarbonate transporter, member 10 | 3.35 | 3.65 | -1.23 | 0.0009 |
| TC11001015.hg.1 | NM_000615; NM_001076682; NM_001242607; NM_001242608; NM_181351; ENST00000316851; ENST00000401611; ENST00000531044; AK057509; AL832563; BC029119; BC047244; uc001pno.3; uc001pnp.3; uc001pnq.3; uc001pnr.3; uc001pns.3; uc001pnt.3; uc021qqo.1; uc021qqp.1 | *NCAM1* | neural cell adhesion molecule 1 | 5.31 | 5.61 | -1.23 | 0.001 |
| TC17001576.hg.1 | NM_001076683; NM_001076684; NM_014233; NR_045058; ENST00000302904; ENST00000343638; ENST00000393606; ENST00000436088; ENST00000526094; ENST00000527034; ENST00000529383; ENST00000533177; ENST00000537550; AK308321; BC042297; uc002igc.3; uc002igd.3; uc002ige.2; uc010czs.3; uc010czt.3 | *UBTF* | upstream binding transcription factor, RNA polymerase I | 7.09 | 7.15 | -1.04 | 0.001 |
| TC19001593.hg.1 | NM_001005376; NM_001005377; NM_002659; ENST00000221264; ENST00000339082; ENST00000340093; BC002788; uc002oxd.2; uc002oxf.2; uc002oxg.2 | *PLAUR* | plasminogen activator, urokinase receptor | 7.24 | 6.96 | 1.21 | 0.0011 |
| TC18000168.hg.1 | NM_152470; ENST00000269439; ENST00000543885; AK055997; AK074561; AK122819; BC012190; uc002lby.1; uc002lcb.1 | *RNF165* | ring finger protein 165 | 4.54 | 4.78 | -1.19 | 0.0011 |
| TC09000641.hg.1 | NR_029611; ENST00000384863; uc004bot.3 | *MIR181A2* | microRNA 181a-2 | 3.44 | 3.79 | -1.27 | 0.0012 |
| TC12003285.hg.1 | NR_002809; AK095700; AK311480 | *LINC01089* | long intergenic non-protein coding RNA 1089 | 6.16 | 5.99 | 1.12 | 0.0013 |
| TC17002023.hg.1 | NR_034082 | *LOC100130950* | uncharacterized LOC100130950 | 4.61 | 4.74 | -1.09 | 0.0014 |
| TC11002932.hg.1 | ENST00000499143; ENST00000529392 | *LOC100507283* | uncharacterized LOC100507283 | 3.75 | 3.6 | 1.11 | 0.0014 |
| TC02000788.hg.1 | NM_032740; ENST00000310981; OTTHUMT00000254389; uc002tpf.3 | *SFT2D3* | SFT2 domain containing 3 | 6.24 | 6 | 1.18 | 0.0014 |
| TC07001800.hg.1 | ENST00000426413; ENST00000431071 | *LVCAT5* | liver cancer-associated transcript 5 | 2.14 | 1.98 | 1.12 | 0.0015 |
| TC07001264.hg.1 | NM_001002010; NM_001166118; NM_016489; ENST00000242210; ENST00000381626; ENST00000396152; ENST00000405342; ENST00000409467; ENST00000409787; BC015856; BC066914; BC071652; OTTHUMT00000328880; OTTHUMT00000328881; OTTHUMT00000328882; OTTHUMT00000328883; OTTHUMT00000328884; OTTHUMT00000328885; OTTHUMT00000328886; OTTHUMT00000328887; OTTHUMT00000328888; OTTHUMT00000328889 | *NT5C3A* | 5-nucleotidase, cytosolic IIIA | 5.67 | 5.45 | 1.17 | 0.0015 |
| TC03001720.hg.1 | NM_020733; ENST00000311127; ENST00000477536; AK074987; OTTHUMT00000355732; OTTHUMT00000355733; OTTHUMT00000355778; OTTHUMT00000355779; OTTHUMT00000355780; OTTHUMT00000355781; uc003ehr.4; uc003ehs.4 | *HEG1* | heart development protein with EGF-like domains 1 | 6.24 | 6.52 | -1.22 | 0.0016 |
| TC13000053.hg.1 | BC038727 | *LINC00566* | long intergenic non-protein coding RNA 566 | 4.23 | 4.05 | 1.14 | 0.0016 |
| TC12003128.hg.1 | BC038786 | *LINC01089* | long intergenic non-protein coding RNA 1089 | 6.29 | 6.13 | 1.12 | 0.0016 |
| TC01005174.hg.1 | X89666 | *OR2M4* | olfactory receptor, family 2, subfamily M, member 4 | 3.52 | 3.78 | -1.2 | 0.0016 |
| TC10000484.hg.1 | NM_001174156; NM_144660; ENST00000372687; ENST00000372690; ENST00000542569; OTTHUMT00000048780; OTTHUMT00000048781; OTTHUMT00000048782; uc001jwx.2; uc001jwy.2 | *SAMD8* | sterile alpha motif domain containing 8 | 5.83 | 5.67 | 1.11 | 0.0016 |
| TC22000722.hg.1 | NM_000878; ENST00000216223; BC025691; uc003aqv.1 | *IL2RB* | interleukin 2 receptor, beta | 7.23 | 7.62 | -1.31 | 0.0017 |
| TC06001531.hg.1 | NM_001145466; NM_001145467; NM_147130; ENST00000340027; ENST00000376071; ENST00000376072; ENST00000376073; ENST00000491161; BC018752; BC052582; OTTHUMT00000076210; OTTHUMT00000076211; OTTHUMT00000076212; OTTHUMT00000076213; OTTHUMT00000259317; OTTHUMT00000259318; uc003nuv.2; uc003nuw.2; uc003nux.1 | *NCR3* | natural cytotoxicity triggering receptor 3 | 5.78 | 6.02 | -1.18 | 0.0017 |
| TC6_apd_hap1000082.hg.1 | NM_001145466; NM_001145467; NM_147130; BC018752; BC052582; OTTHUMT00000309348; OTTHUMT00000309349; OTTHUMT00000309350; OTTHUMT00000309351; OTTHUMT00000309352; OTTHUMT00000309353; OTTHUMT00000309354; uc011enh.1; uc011eni.1; uc011enj.1 | *NCR3* | natural cytotoxicity triggering receptor 3 | 5.78 | 6.02 | -1.18 | 0.0017 |
| TC6_cox_hap2000160.hg.1 | NM_001145466; NM_001145467; NM_147130; BC018752; BC052582; OTTHUMT00000076852; OTTHUMT00000076853; OTTHUMT00000076854; OTTHUMT00000076855; OTTHUMT00000314844; OTTHUMT00000314845; OTTHUMT00000314846; uc011fev.1; uc011few.1; uc011fex.1 | *NCR3* | natural cytotoxicity triggering receptor 3 | 5.78 | 6.02 | -1.18 | 0.0017 |
| TC6_dbb_hap3000149.hg.1 | NM_001145466; NM_001145467; NM_147130; BC018752; BC052582; OTTHUMT00000310101; OTTHUMT00000310102; OTTHUMT00000310103; OTTHUMT00000310104; OTTHUMT00000310105; OTTHUMT00000310106; OTTHUMT00000310107; uc011gcs.1; uc011gct.1; uc011gcu.1 | *NCR3* | natural cytotoxicity triggering receptor 3 | 5.78 | 6.02 | -1.18 | 0.0017 |
| TC6_mann_hap4000137.hg.1 | NM_001145466; NM_001145467; NM_147130; BC018752; BC052582; OTTHUMT00000311251; OTTHUMT00000311252; OTTHUMT00000311253; OTTHUMT00000311254; OTTHUMT00000311255; OTTHUMT00000311256; OTTHUMT00000311257; uc011gzb.1; uc011gzc.1; uc011gzd.1 | *NCR3* | natural cytotoxicity triggering receptor 3 | 5.78 | 6.02 | -1.18 | 0.0017 |
| TC6_qbl_hap6000152.hg.1 | NM_001145466; NM_001145467; NM_147130; BC018752; BC052582; OTTHUMT00000035660; OTTHUMT00000035661; OTTHUMT00000035662; OTTHUMT00000035663; OTTHUMT00000035664; OTTHUMT00000035665; OTTHUMT00000035666; uc011ipb.1; uc011ipc.1; uc011ipd.1 | *NCR3* | natural cytotoxicity triggering receptor 3 | 5.78 | 6.02 | -1.18 | 0.0017 |
| TC6_ssto_hap7000132.hg.1 | NM_001145466; NM_001145467; NM_147130; BC018752; BC052582; OTTHUMT00000099363; OTTHUMT00000099364; OTTHUMT00000099365; OTTHUMT00000099366; OTTHUMT00000099367; OTTHUMT00000099368; OTTHUMT00000099369; uc011jko.1; uc011jkp.1; uc011jkq.1 | *NCR3* | natural cytotoxicity triggering receptor 3 | 5.78 | 6.02 | -1.18 | 0.0017 |
| TC03003335.hg.1 | NM_005677; NM_080538; NM_080539; ENST00000383781; ENST00000383786; ENST00000383788; AJ225895; BC074828; BC144004; OTTHUMT00000343575; OTTHUMT00000343576; OTTHUMT00000343577; OTTHUMT00000343578; OTTHUMT00000345418; OTTHUMT00000345419; OTTHUMT00000346318; uc003bzv.3; uc003bzx.3; uc003cad.1; uc003cae.1; uc010heo.3 | *COLQ* | collagen-like tail subunit (single strand of homotrimer) of asymmetric acetylcholinesterase | 4.95 | 5.09 | -1.1 | 0.0018 |
| TC6_mcf_hap5000137.hg.1 | NM_001145466; NM_001145467; NM_147130; BC018752; BC052582; OTTHUMT00000312144; OTTHUMT00000312145; OTTHUMT00000312146; OTTHUMT00000312147; OTTHUMT00000312148; OTTHUMT00000312149; OTTHUMT00000312150; uc011hrr.1; uc011hrs.1; uc011hrt.1 | *NCR3* | natural cytotoxicity triggering receptor 3 | 5.75 | 6 | -1.19 | 0.0018 |
| TC03001613.hg.1 | NM_016247; ENST00000193391; AF319972; BC144092; DQ980602; OTTHUMT00000353256; uc003duq.2; uc010hpj.1; uc011bhe.2; uc021xbv.1 | *IMPG2* | interphotoreceptor matrix proteoglycan 2 | 2.61 | 2.54 | 1.05 | 0.0019 |
| TC18000418.hg.1 | NM_001242508; NM_018030; NM_080597; ENST00000319481; ENST00000357041; ENST00000399441; ENST00000399443; BC136452; BC144117; BX647893; OTTHUMT00000254902; OTTHUMT00000254903; uc002kvd.3; uc002kve.3; uc002kvf.3; uc010xbc.2 | *OSBPL1A* | oxysterol binding protein-like 1A | 4.73 | 4.57 | 1.12 | 0.0019 |
| TC02000568.hg.1 | NM_001017396; NM_021088; AK295739; BC005068; CR749650; OTTHUMT00000338595; OTTHUMT00000338596; OTTHUMT00000338597; OTTHUMT00000338598; OTTHUMT00000338599; uc002suf.3; uc002sug.3; uc010fhs.3; uc010yue.2 | *ZNF2* | zinc finger protein 2 | 4.73 | 4.83 | -1.07 | 0.0019 |
| TC03001304.hg.1 | NM_001171171; NM_001171172; NM_001171174; NM_001337; ENST00000358309; ENST00000399220; ENST00000541347; ENST00000542107; BC028078; OTTHUMT00000343613; OTTHUMT00000343615; OTTHUMT00000343616; uc003cjl.3; uc021wwa.1; uc021wwb.1; uc021wwc.1 | *CX3CR1* | chemokine (C-X3-C motif) receptor 1 | 8.16 | 8.67 | -1.42 | 0.002 |
| TC0X001091.hg.1 | NR_015353; ENST00000433061; AK130294; AL080202 | *LINC01278* | long intergenic non-protein coding RNA 1278 | 6.74 | 6.96 | -1.16 | 0.002 |
| TC09000865.hg.1 | NM_152569; ENST00000382387; OTTHUMT00000055436; uc003zge.4 | *C9orf66* | chromosome 9 open reading frame 66 | 2.42 | 2.23 | 1.14 | 0.0021 |
| TC02000501.hg.1 | NM_020122; ENST00000409785; BC000178; OTTHUMT00000328942; OTTHUMT00000328976; OTTHUMT00000328977; OTTHUMT00000328978; uc002sox.4 | *KCMF1* | potassium channel modulatory factor 1 | 7.07 | 7 | 1.05 | 0.0022 |
| TC12001202.hg.1 | NM_002258; ENST00000229402; BC114516; uc010sgt.2 | *KLRB1* | killer cell lectin-like receptor subfamily B, member 1 | 5.93 | 6.63 | -1.63 | 0.0022 |
| TC05001140.hg.1 | NR_036242; uc021xwi.1 | *MIR4278* | microRNA 4278 | 1.61 | 1.87 | -1.2 | 0.0022 |
| TC08001850.hg.1 | AY429564 | *PTK2B* | protein tyrosine kinase 2 beta | 8.2 | 8.08 | 1.09 | 0.0022 |
| TC19000553.hg.1 | NM_001190441; ENST00000392051; uc021uun.1 | *LGALS16* | lectin, galactoside-binding, soluble, 16 | 2.45 | 2.6 | -1.11 | 0.0023 |
| TC06002541.hg.1 | BC070230 | *RPS18* | ribosomal protein S18 | 1.88 | 2 | -1.09 | 0.0023 |
| TC07001243.hg.1 | NM_001145513; NM_001145514; NM_001145515; NM_014766; ENST00000242059; ENST00000409497; ENST00000409570; ENST00000425819; ENST00000426154; ENST00000434476; ENST00000494620; AK289514; AK294891; AK295800; BC040492; OTTHUMT00000214231; OTTHUMT00000327695; OTTHUMT00000327696; OTTHUMT00000327697; OTTHUMT00000327698; OTTHUMT00000327699; OTTHUMT00000327700; OTTHUMT00000376257; uc003tak.3; uc010kvp.3; uc011jzw.2; uc011jzx.2; uc011jzy.2; uc011jzz.2; uc011kaa.2 | *SCRN1* | secernin 1 | 5.5 | 5.64 | -1.1 | 0.0023 |
| TC06001132.hg.1 | NM_001007466; NM_020245; ENST00000367094; ENST00000367097; AK304056; OTTHUMT00000042869; OTTHUMT00000042870; uc003qrf.3; uc003qrg.3; uc011efo.2 | *TULP4* | tubby like protein 4 | 5.92 | 6.07 | -1.1 | 0.0023 |
| TC13000669.hg.1 | NR_037407; uc021rjp.1 | *MIR3613* | microRNA 3613 | 2.96 | 2.54 | 1.34 | 0.0024 |
| TC12002089.hg.1 | NM_145058; ENST00000280571; BC013042; uc001uey.1 | *RILPL2* | Rab interacting lysosomal protein-like 2 | 6.36 | 6.2 | 1.12 | 0.0024 |
| TC09001258.hg.1 | NM_005077; ENST00000376463; ENST00000376472; ENST00000376484; ENST00000376499; ENST00000464999; AB209854; AK293606; AK304565; BC000228; BC010100; BC015747; OTTHUMT00000055407; OTTHUMT00000055408; OTTHUMT00000055409; OTTHUMT00000055410; OTTHUMT00000055411; OTTHUMT00000055412; uc004aly.3; uc004alz.3; uc004ama.1; uc011lsr.2; uc011lss.1 | *TLE1* | transducin-like enhancer of split 1 (E(sp1) homolog, Drosophila) | 5.23 | 5.33 | -1.07 | 0.0024 |
| TC12000788.hg.1 | NM_020244; ENST00000229266; ENST00000549872; ENST00000550385; AF195624; AK093067; BC020819; BC050429; uc001tin.3; uc001tio.3; uc001tip.1 | *CHPT1* | choline phosphotransferase 1 | 6.23 | 5.78 | 1.37 | 0.0025 |
| TC11003375.hg.1 | BC127775 | *KMT2A* | lysine (K)-specific methyltransferase 2A | 4.77 | 4.92 | -1.11 | 0.0025 |
| TC09001100.hg.1 | NM_033160; AK292150; BC031626; uc004abs.2; uc010mmm.2; uc010mmn.1 | *ZNF658* | zinc finger protein 658 | 4.37 | 4.27 | 1.08 | 0.0025 |
| TC12000119.hg.1 | NM_016184; NM_194447; NM_194448; NM_194450; ENST00000229332; ENST00000345999; ENST00000352620; ENST00000360500; BC074822; BC117439; BC117441; EU196761; uc001qtz.1; uc001qub.1; uc001quc.1; uc009zga.1; uc009zgb.1 | *CLEC4A* | C-type lectin domain family 4, member A | 6.11 | 5.75 | 1.28 | 0.0026 |
| TC07001761.hg.1 | NM_014705; ENST00000428084; ENST00000437633; ENST00000476846; ENST00000494651; AB018259; AK294722; AK294820; AK309605; BC117688; BC117689; BC117694; OTTHUMT00000338369; OTTHUMT00000338940; OTTHUMT00000338941; OTTHUMT00000338942; OTTHUMT00000338943; OTTHUMT00000339157; OTTHUMT00000339158; OTTHUMT00000339159; OTTHUMT00000339160; OTTHUMT00000339161; OTTHUMT00000339162; OTTHUMT00000339163; OTTHUMT00000339164; OTTHUMT00000339165; OTTHUMT00000339166; OTTHUMT00000339167; uc003vfv.3; uc003vfw.3; uc003vfx.3; uc003vfy.3; uc010ljt.1; uc011kml.2; uc011kmm.2 | *DOCK4* | dedicator of cytokinesis 4 | 5.14 | 4.8 | 1.27 | 0.0026 |
| TC09000691.hg.1 | NM_001242352; NM_001242353; NM_001242354; NM_002540; NM_153432; NM_153433; NM_153435; NM_153436; NM_153437; NM_153439; NM_153440; ENST00000351030; ENST00000372791; ENST00000372814; ENST00000393533; ENST00000448249; ENST00000488909; ENST00000546203; AK126816; AK295662; AK308278; AK309917; BC010629; BC091500; OTTHUMT00000054439; OTTHUMT00000054440; OTTHUMT00000054441; OTTHUMT00000054442; OTTHUMT00000054443; OTTHUMT00000054444; OTTHUMT00000054445; OTTHUMT00000054446; OTTHUMT00000054447; OTTHUMT00000054448; OTTHUMT00000054449; OTTHUMT00000054450; OTTHUMT00000054452; OTTHUMT00000054453; OTTHUMT00000054454; uc004bva.3; uc004bvb.3; uc004bvc.3; uc004bvd.4; uc004bve.3; uc004bvh.3; uc010myb.3; uc010myc.3; uc011maz.2; uc011mba.2; uc011mbc.2; uc011mbd.2; uc011mbe.2; uc011mbf.2; uc022boj.1 | *ODF2* | outer dense fiber of sperm tails 2 | 5.62 | 5.69 | -1.05 | 0.0026 |
| TC01001469.hg.1 | NM_002995; ENST00000367818; BC069817; BC070309; OTTHUMT00000083612; uc001gfo.2 | *XCL1* | chemokine (C motif) ligand 1 | 3.81 | 4.24 | -1.35 | 0.0026 |
| TC16000357.hg.1 | NR_002966; ENST00000384028; uc002dzh.1 | *SNORA30* | small nucleolar RNA, H/ACA box 30 | 3.21 | 2.91 | 1.23 | 0.0028 |
| TC03003389.hg.1 | NM_022135; ENST00000264231; ENST00000468801; ENST00000474523; ENST00000493094; AK314819; BC026911; BC044929; OTTHUMT00000355377; OTTHUMT00000355378; OTTHUMT00000355379; OTTHUMT00000355488; OTTHUMT00000355489; OTTHUMT00000355491; OTTHUMT00000355492; OTTHUMT00000355726; OTTHUMT00000355727; uc003ecx.1; uc010hqw.1 | *POPDC2* | popeye domain containing 2 | 4.82 | 4.89 | -1.05 | 0.0029 |
| TC04000410.hg.1 | NM_002620; ENST00000226524; BC130653; OTTHUMT00000252495; uc003hhg.1 | *PF4V1* | platelet factor 4 variant 1 | 5.78 | 5.38 | 1.32 | 0.0031 |
| TC17001433.hg.1 | NM_003559; BC027459; uc002hqs.3; uc021twj.1 | *PIP4K2B* | phosphatidylinositol-5-phosphate 4-kinase, type II, beta | 6.17 | 6.24 | -1.05 | 0.0031 |
| TC01001490.hg.1 | NM_002022; ENST00000367749; ENST00000462992; BC002780; OTTHUMT00000086223; OTTHUMT00000086224; OTTHUMT00000086225; OTTHUMT00000086226; OTTHUMT00000086227; uc001gho.3 | *FMO4* | flavin containing monooxygenase 4 | 3.16 | 3.33 | -1.12 | 0.0032 |
| TC12000677.hg.1 | NM_032230; ENST00000248306; ENST00000547357; BC029120; uc001szq.3 | *METTL25* | methyltransferase like 25 | 4.77 | 4.63 | 1.1 | 0.0032 |
| TC02001118.hg.1 | NM_001128928; NM_002194; ENST00000322522; ENST00000392329; ENST00000417336; AK093560; BC015496; BC106006; OTTHUMT00000255932; OTTHUMT00000334960; OTTHUMT00000334961; OTTHUMT00000334962; OTTHUMT00000334963; OTTHUMT00000334964; OTTHUMT00000334965; OTTHUMT00000334966; OTTHUMT00000334967; OTTHUMT00000334968; OTTHUMT00000334969; OTTHUMT00000334970; OTTHUMT00000334971; OTTHUMT00000334972; uc002urx.4; uc002ury.4; uc010fsb.3 | *INPP1* | inositol polyphosphate-1-phosphatase | 4.75 | 4.81 | -1.04 | 0.0033 |
| TC12001478.hg.1 | NM_001012300; NM_006337; ENST00000343810; ENST00000357123; ENST00000546244; ENST00000547182; ENST00000550165; AF068007; BC011794; BX538079; uc001rui.1; uc001ruj.2; uc001ruk.1; uc009zlj.1 | *MCRS1* | microspherule protein 1 | 5.87 | 5.95 | -1.06 | 0.0033 |
| TC11001145.hg.1 | ENST00000499143; BC036195 | *LOC100507283* | uncharacterized LOC100507283 | 3.66 | 3.5 | 1.12 | 0.0034 |
| TC10001459.hg.1 | ENST00000412019 | *C1DP3* | C1D nuclear receptor corepressor pseudogene 3 | 1.98 | 1.82 | 1.11 | 0.0035 |
| TC14000874.hg.1 | NM_001004714; ENST00000315693; uc010tkz.2 | *OR4K13* | olfactory receptor, family 4, subfamily K, member 13 | 2.01 | 2.24 | -1.17 | 0.0035 |
| TC17001385.hg.1 | NM_002983; BC071834; BC171831; uc002hkv.3 | *CCL3* | chemokine (C-C motif) ligand 3 | 4.21 | 4.41 | -1.15 | 0.0036 |
| TC10002105.hg.1 | BC104671 | *DDX21* | DEAD (Asp-Glu-Ala-Asp) box helicase 21 | 5.81 | 5.97 | -1.12 | 0.0036 |
| TC06000918.hg.1 | NM_001080976; NM_013352; ENST00000331677; ENST00000359564; ENST00000452085; AK095123; AK128607; AK293872; AK301513; BC039245; BC043526; OTTHUMT00000041940; OTTHUMT00000041941; uc003pwq.1; uc003pwr.3; uc003pws.3; uc003pwt.3; uc003pwu.3; uc011ebf.1; uc011ebg.2 | *DSE* | dermatan sulfate epimerase | 4.07 | 3.97 | 1.07 | 0.0036 |
| TC02004734.hg.1 | BC047057 | *PDE1A* | phosphodiesterase 1A, calmodulin-dependent | 1.9 | 1.71 | 1.15 | 0.0036 |
| TC13001487.hg.1 | BC039553; ENST00000433480; ENST00000436963 | *LINC00441* | long intergenic non-protein coding RNA 441 | 3.39 | 3.25 | 1.11 | 0.0038 |
| TC14000305.hg.1 | NM_000953; ENST00000306051; ENST00000553372; BC040968; OTTHUMT00000276889; uc001wzq.3 | *PTGDR* | prostaglandin D2 receptor (DP) | 6.6 | 7.03 | -1.35 | 0.0038 |
| TC07000288.hg.1 | DQ574672 | *LINC01061* | long intergenic non-protein coding RNA 1061 | 7.55 | 7.07 | 1.4 | 0.0039 |
| TC0X001092.hg.1 | NM_001173479; NM_001173480; NM_015185; ENST00000253401; ENST00000374870; ENST00000374872; ENST00000374878; ENST00000437457; ENST00000495564; AK295178; AL832116; BC018106; BC043497; BC117406; OTTHUMT00000056937; OTTHUMT00000056938; OTTHUMT00000056939; OTTHUMT00000056940; OTTHUMT00000056941; OTTHUMT00000056942; uc004dvj.2; uc004dvk.1; uc004dvl.2; uc004dvm.1; uc004dvn.3; uc011mos.1; uc011mot.2 | *ARHGEF9* | Cdc42 guanine nucleotide exchange factor 9 | 5.23 | 5.4 | -1.12 | 0.004 |
| TC19001415.hg.1 | NR_033982; uc002nwj.3 | *LOC400685* | uncharacterized LOC400685 | 4.14 | 4.33 | -1.14 | 0.004 |
| TC02002865.hg.1 | NM_006056; ENST00000305141; BC036543; BC051914; OTTHUMT00000256961; uc002vry.4 | *NMUR1* | neuromedin U receptor 1 | 6.07 | 6.34 | -1.2 | 0.0042 |
| TC15002377.hg.1 | NR_037600; NR_037601; NR_037602 | *LINC01578* | long intergenic non-protein coding RNA 1578 | 7.04 | 6.82 | 1.17 | 0.0044 |
| TC02001193.hg.1 | NM_173511; ENST00000392238; AB053315; BC106906; BC106907; OTTHUMT00000335888; OTTHUMT00000335890; uc010zhw.2; uc010zhx.2 | *FAM117B* | family with sequence similarity 117, member B | 7.66 | 7.86 | -1.15 | 0.0045 |
| TC03002192.hg.1 | AK126307; ENST00000442809 | *LINC01266* | long intergenic non-protein coding RNA 1266 | 1.77 | 1.7 | 1.06 | 0.0045 |
| TC03000331.hg.1 | NM_001122870; NM_144641; ENST00000296487; ENST00000323588; ENST00000409502; AK056894; AK129647; BC009644; OTTHUMT00000329230; OTTHUMT00000329231; OTTHUMT00000329232; OTTHUMT00000329233; OTTHUMT00000329234; OTTHUMT00000329235; OTTHUMT00000329236; OTTHUMT00000350206; uc003ddf.4; uc003ddg.4; uc003ddh.4; uc011bed.2 | *PPM1M* | protein phosphatase, Mg2+/Mn2+ dependent, 1M | 6.35 | 6.27 | 1.06 | 0.0045 |
| TC11002744.hg.1 | AY548972 | *TCIRG1* | T-cell, immune regulator 1, ATPase, H+ transporting, lysosomal V0 subunit A3 | 6.03 | 5.94 | 1.07 | 0.0045 |
| TC07000684.hg.1 | NM_152750; ENST00000317716; ENST00000470188; ENST00000478080; AK074414; AK125261; AK131293; AK304118; AK304352; BC111696; OTTHUMT00000349022; OTTHUMT00000349023; OTTHUMT00000349024; OTTHUMT00000349025; OTTHUMT00000349026; OTTHUMT00000349027; OTTHUMT00000349028; OTTHUMT00000349029; OTTHUMT00000349030; OTTHUMT00000349031; OTTHUMT00000349032; OTTHUMT00000349033; OTTHUMT00000349034; OTTHUMT00000349035; uc003vdk.3; uc003vdl.4; uc003vdm.4; uc003vdn.3; uc011kls.1; uc011klt.2 | *CDHR3* | cadherin-related family member 3 | 3.28 | 3.36 | -1.06 | 0.0046 |
| TC12000108.hg.1 | NM_014718; ENST00000266546; ENST00000331148; ENST00000537408; AY753302; BC104767; BC111491; BC112283; uc001qsr.3; uc001qss.3 | *CLSTN3* | calsyntenin 3 | 6.04 | 6.2 | -1.12 | 0.0046 |
| TC02003128.hg.1 | AK127582 | *LOC105374363* | uncharacterized LOC105374363 | 5.51 | 5.59 | -1.06 | 0.0046 |
| TC19000008.hg.1 | NM_005317; ENST00000264553; BC025701 | *GZMM* | granzyme M | 6.57 | 6.8 | -1.17 | 0.0048 |
| TC05001028.hg.1 | AK126616 | *LOC100128340* | uncharacterized LOC100128340 | 5.03 | 4.91 | 1.09 | 0.0048 |
| TC04002692.hg.1 | BC070391 | *LOC101926918* | uncharacterized LOC101926918 | 3.8 | 3.51 | 1.23 | 0.0048 |
| TC01002764.hg.1 | NR_031664; ENST00000408276; uc021oos.1 | *MIR1262* | microRNA 1262 | 3.64 | 3.05 | 1.5 | 0.0048 |
| TC0X001624.hg.1 | NR_027783 | *SAT1* | spermidine/spermine N1-acetyltransferase 1 | 8.07 | 7.88 | 1.15 | 0.0049 |
| TC08001248.hg.1 | NM_014729; ENST00000361421; BC016665; OTTHUMT00000378307; uc003xtw.1 | *TOX* | thymocyte selection-associated high mobility group box | 6.38 | 6.72 | -1.27 | 0.0049 |
| TC01002442.hg.1 | NR_039616; uc021okj.1 | *MIR4420* | microRNA 4420 | 5.18 | 4.98 | 1.15 | 0.005 |
| TC12000212.hg.1 | NR_036619; uc021qvt.1 | *SKP1P2* | S-phase kinase-associated protein 1 pseudogene 2 | 3.22 | 3.03 | 1.14 | 0.005 |
| TC07000646.hg.1 | NM_006349; ENST00000305105; ENST00000492315; AK125123; BC017333; OTTHUMT00000347488; OTTHUMT00000347489; OTTHUMT00000347490; OTTHUMT00000347492; uc003uye.3; uc003uyf.3 | *ZNHIT1* | zinc finger, HIT-type containing 1 | 6.45 | 6.34 | 1.08 | 0.005 |
| TC14000165.hg.1 | NM_001002000; NM_001002001; NM_001002002; NM_016576; ENST00000348719; ENST00000355299; ENST00000399440; ENST00000420554; ENST00000456667; ENST00000557854; ENST00000558701; ENST00000559104; ENST00000559836; ENST00000559910; ENST00000560517; AK226166; BC003053; BC008021; BC009832; BC093039; BX161518; BX247993; uc001wnr.3; uc001wns.3; uc001wnu.2; uc001wnv.3; uc001wnw.3; uc001wnx.3; uc010all.3; uc010toe.1 | *GMPR2* | guanosine monophosphate reductase 2 | 5.86 | 5.72 | 1.1 | 0.0051 |
| TC0X000589.hg.1 | NM_004541; ENST00000371437; BC000266; OTTHUMT00000058080; uc004esc.4 | *NDUFA1* | NADH dehydrogenase (ubiquinone) 1 alpha subcomplex, 1, 7.5kDa | 4.87 | 4.99 | -1.09 | 0.0051 |
| TC11000957.hg.1 | NM_001166; ENST00000227758; ENST00000527910; ENST00000530675; ENST00000532672; AK303197; BC016174; BC028578 | *BIRC2* | baculoviral IAP repeat containing 2 | 6.75 | 6.6 | 1.11 | 0.0052 |
| TC09000240.hg.1 | BC118602; BC121813 | *FAM74A1; FAM74A4* | family with sequence similarity 74, member A1; family with sequence similarity 74, member A4 | 2.27 | 2.21 | 1.04 | 0.0052 |
| TC01004073.hg.1 | NM_198074; NR_027309; ENST00000366487; BC030717; OTTHUMT00000097626; uc001idd.3; uc009xgy.3 | *OR2C3; GCSAML-AS1* | olfactory receptor, family 2, subfamily C, member 3; GCSAML antisense RNA 1 | 2.25 | 2.18 | 1.05 | 0.0052 |
| TC01003621.hg.1 | NM_015101; ENST00000361927; ENST00000367520; ENST00000367521; ENST00000486375; AK095090; AK095192; AK127287; AK293973; BC035672; OTTHUMT00000086128; OTTHUMT00000086129; OTTHUMT00000086130; OTTHUMT00000086131; uc001gqp.3; uc001gqq.3; uc001gqr.3; uc001gqs.3; uc010poj.1 | *COLGALT2* | collagen beta(1-O)galactosyltransferase 2 | 3.86 | 4.06 | -1.15 | 0.0054 |
| TC12001413.hg.1 | NM_001242397; NM_002822; ENST00000395510; ENST00000547564; ENST00000548315; ENST00000552521; AK127868; BC022344; BC043148; uc001rob.3; uc001roc.3 | *TWF1* | twinfilin actin binding protein 1 | 5.67 | 5.57 | 1.07 | 0.0054 |
| TC07002665.hg.1 | DQ995344 | *ATP6V0E2* | ATPase, H+ transporting V0 subunit e2 | 6.58 | 6.44 | 1.1 | 0.0057 |
| TC16001088.hg.1 | NM_001201477; NM_018092; ENST00000303155; AK027630; OTTHUMT00000256766; uc002eeq.2; uc002eer.2; uc010vgf.2 | *NETO2* | neuropilin (NRP) and tolloid (TLL)-like 2 | 3.85 | 3.71 | 1.1 | 0.0057 |
| TC07001170.hg.1 | NM_175886; ENST00000506618; BC062797; OTTHUMT00000327667; uc003stz.3 | *PRPS1L1* | phosphoribosyl pyrophosphate synthetase 1-like 1 | 3.27 | 3.54 | -1.21 | 0.0057 |
| TC02005039.hg.1 | NM_174898; ENST00000308528; ENST00000409448; AY358988; BC029126; OTTHUMT00000253127; OTTHUMT00000330315; uc002szy.3; uc010yvo.2 | *LYG1* | lysozyme G-like 1 | 3.01 | 3.07 | -1.04 | 0.0058 |
| TC17000813.hg.1 | NM_000891; ENST00000243457; ENST00000535240; AK314081; uc002jir.3; uc010dfg.3 | *KCNJ2* | potassium channel, inwardly rectifying subfamily J, member 2 | 7 | 6.6 | 1.32 | 0.0059 |
| TC11002053.hg.1 | NM_017907; ENST00000278671; ENST00000535107; ENST00000538404; ENST00000539797; ENST00000545249; BC001706; uc001ort.3 | *LAMTOR1* | late endosomal/lysosomal adaptor, MAPK and MTOR activator 1 | 6.03 | 5.97 | 1.04 | 0.0059 |
| TC22001485.hg.1 | NR_038911; OTTHUMT00000320008 | *MIF-AS1* | MIF antisense RNA 1 | 4.6 | 4.78 | -1.13 | 0.0061 |
| TC09000846.hg.1 | NM_015456; ENST00000343053; BC011892; OTTHUMT00000254710; uc004cmm.4 | *NELFB* | negative elongation factor complex member B | 6.45 | 6.53 | -1.06 | 0.0061 |
| TC01002024.hg.1 | ENST00000355281; OTTHUMT00000096851 | *OR2L5* | olfactory receptor, family 2, subfamily L, member 5 | 2.57 | 2.08 | 1.41 | 0.0061 |
| TC09002250.hg.1 | BC030772; NR_038975 | *MIR181A2HG* | MIR181A2 host gene | 3.91 | 4.37 | -1.38 | 0.0062 |
| TC01003884.hg.1 | NR_039896; uc021pjq.1 | *MIR4742* | microRNA 4742 | 3.36 | 3.08 | 1.21 | 0.0062 |
| TC07000439.hg.1 | AK127026 | *POM121C* | POM121 transmembrane nucleoporin C | 5.15 | 5.28 | -1.1 | 0.0062 |
| TC17002868.hg.1 | NM_001130842; NM_020652; ENST00000395893; ENST00000395894; ENST00000421016; ENST00000464847; AF217226; AK092259; BC128259; BC128411; OTTHUMT00000130696; OTTHUMT00000130698; OTTHUMT00000130699; OTTHUMT00000131013; OTTHUMT00000131014; uc002goz.4; uc002gpa.3; uc010cot.3; uc010vwa.2 | *ZNF286A* | zinc finger protein 286A | 4.65 | 4.72 | -1.05 | 0.0062 |
| TC15002153.hg.1 | NR_040059; NR_040060; NR_040061; NR_040062 | *SRP14-AS1* | SRP14 antisense RNA1 (head to head) | 3.6 | 3.55 | 1.04 | 0.0063 |
| TC08000348.hg.1 | NM_003350; ENST00000517630; ENST00000520595; ENST00000520809; ENST00000521346; ENST00000521628; ENST00000523111; BC007051; BC016332; BC016710; BC028673; BC062418; OTTHUMT00000377808; OTTHUMT00000377809; OTTHUMT00000377810; OTTHUMT00000377811; OTTHUMT00000377812; OTTHUMT00000377813; OTTHUMT00000377815; OTTHUMT00000378401; uc003xqm.3 | *UBE2V2* | ubiquitin conjugating enzyme E2 variant 2 | 5.22 | 5.3 | -1.06 | 0.0063 |
| TC01004021.hg.1 | NM_022469; ENST00000318160; BC046632; OTTHUMT00000096286; uc001hys.3 | *GREM2* | gremlin 2, DAN family BMP antagonist | 4.2 | 4.28 | -1.06 | 0.0064 |
| TC13001099.hg.1 | BC036321 | *LOC101929657* | uncharacterized LOC101929657 | 2.37 | 2.19 | 1.13 | 0.0065 |
| TC20000942.hg.1 | NM_001136021; NM_012340; NM_173091; ENST00000371564; ENST00000396009; ENST00000414705; BC136418; BC144074; EU887575; EU887576; EU887577; EU887579; EU887580; OTTHUMT00000079729; OTTHUMT00000079730; OTTHUMT00000258917 | *NFATC2* | nuclear factor of activated T-cells, cytoplasmic, calcineurin-dependent 2 | 6.75 | 7.05 | -1.23 | 0.0065 |
| TC18000201.hg.1 | ENST00000440667 | *RSL24D1P11* | ribosomal L24 domain containing 1 pseudogene 11 | 2.84 | 2.59 | 1.2 | 0.0066 |
| TC19001963.hg.1 | NR_030717 | *SNAPC2* | small nuclear RNA activating complex polypeptide 2 | 6.05 | 6.14 | -1.07 | 0.0066 |
| TC21000317.hg.1 | NR_038872; ENST00000419069; uc021wht.1 | *LINC00317* | long intergenic non-protein coding RNA 317 | 1.82 | 1.72 | 1.07 | 0.0067 |
| TC18000162.hg.1 | BC038429 | *LOC284263* | uncharacterized LOC284263 | 2.11 | 2.3 | -1.14 | 0.0067 |
| TC05003295.hg.1 | AK054753 | *LOC102546294* | uncharacterized LOC102546294 | 3.37 | 3.24 | 1.09 | 0.0067 |
| TC18000698.hg.1 | AK055997 | *RNF165* | ring finger protein 165 | 4.62 | 4.93 | -1.24 | 0.0067 |
| TC0X000112.hg.1 | NM_002970; NR_027783; ENST00000379251; ENST00000379253; ENST00000379254; ENST00000379270; ENST00000489394; AK310094; AK315888; BC002503; BC008424; OTTHUMT00000056056; OTTHUMT00000056057; OTTHUMT00000056058; OTTHUMT00000056059; OTTHUMT00000056060; OTTHUMT00000056061; OTTHUMT00000056062; OTTHUMT00000056063; OTTHUMT00000056064; uc004dau.3; uc004dav.3; uc010nfv.3 | *SAT1* | spermidine/spermine N1-acetyltransferase 1 | 6.98 | 6.78 | 1.15 | 0.0067 |
| TC02000649.hg.1 | NM_001004720; NM_001004722; NM_003581; ENST00000233154; ENST00000393349; ENST00000451463; ENST00000522586; BC000103; BC007195; OTTHUMT00000329634; OTTHUMT00000329635; OTTHUMT00000329636; OTTHUMT00000329637; OTTHUMT00000376091; uc002tdg.3; uc002tdh.3; uc002tdi.3 | *NCK2* | NCK adaptor protein 2 | 7.27 | 7.17 | 1.07 | 0.0068 |
| TC03000518.hg.1 | NM_017819; ENST00000309922; BC035967; OTTHUMT00000353400; OTTHUMT00000353401 | *TRMT10C* | tRNA methyltransferase 10C, mitochondrial RNase P subunit | 4.13 | 3.98 | 1.11 | 0.0068 |
| TC01005450.hg.1 | NR_024174; NR_024176 | *MKNK1* | MAP kinase interacting serine/threonine kinase 1 | 7.42 | 7.21 | 1.16 | 0.0069 |
| TC09002678.hg.1 | AB212827 | *PTCH1* | patched 1 | 5.22 | 5.44 | -1.16 | 0.0069 |
| TC17002655.hg.1 | NR_045058 | *UBTF* | upstream binding transcription factor, RNA polymerase I | 7.07 | 7.16 | -1.07 | 0.0069 |
| TC07002185.hg.1 | BC042028 | *ITGB8* | integrin beta 8 | 1.51 | 1.19 | 1.25 | 0.007 |
| TC13001370.hg.1 | BC015677 | *LOC646482* | uncharacterized LOC646482 | 4.03 | 3.83 | 1.15 | 0.007 |
| TC18000774.hg.1 | BC039507 | *LOC101927606* | uncharacterized LOC101927606 | 5.36 | 5.2 | 1.12 | 0.007 |
| TC05001875.hg.1 | NM_002587; NM_032420; ENST00000287008; ENST00000394536; ENST00000503492; ENST00000511044; AK304231; BC035812; OTTHUMT00000251862; OTTHUMT00000320587; OTTHUMT00000370621; OTTHUMT00000370622; OTTHUMT00000370623; OTTHUMT00000370624; OTTHUMT00000370625; OTTHUMT00000370626; uc003llp.3; uc003llq.3; uc011dbf.2 | *PCDH1* | protocadherin 1 | 5.16 | 5.27 | -1.08 | 0.007 |
| TC12000857.hg.1 | NM_013300; ENST00000377673; ENST00000538285; ENST00000546396; ENST00000547539; ENST00000548869; AK297684; AK308802; BC020967; uc001tqu.4; uc009zvo.2; uc010sxz.1 | *FAM216A* | family with sequence similarity 216, member A | 3.96 | 3.88 | 1.05 | 0.0071 |
| TC08000702.hg.1 | ENST00000509350; AK057448 | *LOC101927543* | uncharacterized LOC101927543 | 3.54 | 3.4 | 1.1 | 0.0071 |
| TC10002544.hg.1 | BC042008; NR_024284 | *ZEB1-AS1* | ZEB1 antisense RNA 1 | 4.4 | 4.34 | 1.04 | 0.0071 |
| TC0Y000063.hg.1 | NM_001001722; NM_004825; ENST00000250838; ENST00000426790; BC069087; BC130426; OTTHUMT00000099658; uc004ftm.1 | *CDY2B; CDY2A* | chromodomain protein, Y-linked, 2B; chromodomain protein, Y-linked, 2A | 2.23 | 2.1 | 1.1 | 0.0072 |
| TC15000194.hg.1 | NM_001146094; NM_001146095; NM_001146096; NM_014967; ENST00000362065; BC047882; CR936727; uc001zfc.3; uc001zfd.3; uc001zfe.3; uc001zff.3; uc010azw.2 | *FAN1* | FANCD2/FANCI-associated nuclease 1 | 5.05 | 4.95 | 1.07 | 0.0072 |
| TC11002470.hg.1 | ENST00000530957 | *IGSF9B* | immunoglobulin superfamily, member 9B | 4.58 | 4.43 | 1.1 | 0.0072 |
| TC06003476.hg.1 | AL831898 | *LOC285812* | uncharacterized LOC285812 | 4.79 | 5.1 | -1.24 | 0.0072 |
| TC08001602.hg.1 | NM_014751; ENST00000325064; ENST00000378017; ENST00000431961; ENST00000518547; ENST00000523587; ENST00000524090; AK027015; AK126651; AK295681; AK302036; BC023998; EF560715; OTTHUMT00000109623; OTTHUMT00000109624; OTTHUMT00000109625; OTTHUMT00000381613; OTTHUMT00000381614; OTTHUMT00000381615; OTTHUMT00000381616; OTTHUMT00000381617; OTTHUMT00000381618; OTTHUMT00000381619; OTTHUMT00000381620; OTTHUMT00000381621; OTTHUMT00000381622; OTTHUMT00000381623; OTTHUMT00000381624; OTTHUMT00000381625; OTTHUMT00000381626; uc003yrh.2; uc003yri.2; uc003yrj.2; uc003yrk.2; uc003yrl.2; uc011lin.1; uc011lio.1 | *MTSS1* | metastasis suppressor 1 | 6.49 | 6.75 | -1.2 | 0.0072 |
| TC12000060.hg.1 | NM_001759; ENST00000261254; ENST00000541542; BC010958; BC089384; uc001qmo.3 | *CCND2* | cyclin D2 | 7.32 | 7.5 | -1.13 | 0.0073 |
| TC15001692.hg.1 | NR_026813; uc002bcp.4 | *LINC00597* | long intergenic non-protein coding RNA 597 | 3.05 | 3.29 | -1.17 | 0.0073 |
| TC01004933.hg.1 | AJ277915 | *LHX9* | LIM homeobox 9 | 3.86 | 4.05 | -1.14 | 0.0074 |
| TC01002026.hg.1 | NM_001004686; ENST00000366479; BC104792; BC104794; OTTHUMT00000096871; uc001idw.3 | *OR2L2* | olfactory receptor, family 2, subfamily L, member 2 | 2.21 | 1.9 | 1.25 | 0.0074 |
| TC20001464.hg.1 | AK055580 | *RALGAPA2* | Ral GTPase activating protein, alpha subunit 2 (catalytic) | 7.4 | 7.05 | 1.28 | 0.0074 |
| TC0X001369.hg.1 | NM_021183; ENST00000342983; ENST00000370874; ENST00000460462; AK310997; BC003403; BC035382; BX647223; OTTHUMT00000058312; OTTHUMT00000058313; OTTHUMT00000058314; OTTHUMT00000058315 | *RAP2C* | RAP2C, member of RAS oncogene family | 5.86 | 5.81 | 1.04 | 0.0074 |
| TC06003477.hg.1 | NR_038459 | *GFOD1* | glucose-fructose oxidoreductase domain containing 1 | 3.76 | 4 | -1.18 | 0.0075 |
| TC16002053.hg.1 | NM_001199107; NM_020705; ENST00000293970; BC112389; BC127014; BC127015; BX648283; uc002cqk.3; uc002cql.3; uc010bsm.3 | *TBC1D24* | TBC1 domain family, member 24 | 4.99 | 5.13 | -1.1 | 0.0075 |
| TC05003107.hg.1 | BC047373 | *CTD-2201I18.1* | uncharacterized LOC101929215 | 4.25 | 4.11 | 1.1 | 0.0076 |
| TC04000367.hg.1 | ENST00000440470 | *GCOM2* | GRINL1B complex locus 2, pseudogene | 5.58 | 5.34 | 1.18 | 0.0076 |
| TC08000012.hg.1 | NM_014867; ENST00000320248; OTTHUMT00000374601; uc003wpw.4 | *KBTBD11* | kelch repeat and BTB (POZ) domain containing 11 | 5.74 | 5.65 | 1.06 | 0.0076 |
| TC07001559.hg.1 | NM_006379; ENST00000265361; ENST00000419255; ENST00000487621; AK299322; AK304294; BC030690; OTTHUMT00000253279; OTTHUMT00000339901; OTTHUMT00000339902; OTTHUMT00000339903; OTTHUMT00000339904; OTTHUMT00000339905; OTTHUMT00000339906; OTTHUMT00000339907; OTTHUMT00000339908; uc003uhj.3; uc011kgw.2; uc011kgx.1 | *SEMA3C* | sema domain, immunoglobulin domain (Ig), short basic domain, secreted, (semaphorin) 3C | 4.18 | 3.94 | 1.18 | 0.0076 |
| TC01001673.hg.1 | NM_001017403; NM_001017404; NM_021636; ENST00000255432; ENST00000308543; ENST00000367278; ENST00000439764; AK123055; BC038795; BC047905; OTTHUMT00000099143; OTTHUMT00000099144; OTTHUMT00000099145; OTTHUMT00000099148; OTTHUMT00000099149; OTTHUMT00000359436; OTTHUMT00000359437; OTTHUMT00000359438; uc001gxu.3; uc001gxv.3; uc001gxw.3; uc009xab.3; uc009xac.1 | *LGR6* | leucine-rich repeat containing G protein-coupled receptor 6 | 5.32 | 5.44 | -1.09 | 0.0077 |
| TC11002538.hg.1 | NR_002777 | *TRIM78P* | tripartite motif containing 78, pseudogene | 4.09 | 3.96 | 1.09 | 0.0077 |
| TC02002926.hg.1 | NR_026664; uc010zob.1 | *MGC16025* | uncharacterized LOC85009 | 5.37 | 5.23 | 1.1 | 0.0078 |
| TC16001083.hg.1 | NM_024745; ENST00000303383; BC030699; OTTHUMT00000255740; uc002eec.4 | *SHCBP1* | SHC SH2-domain binding protein 1 | 2.57 | 2.47 | 1.07 | 0.0078 |
| TC11001932.hg.1 | NM_001077241; NM_182556; ENST00000294187; ENST00000398802; ENST00000526432; ENST00000527174; ENST00000534028; AY597807; BC036869; BC041100; uc001odr.1; uc001ods.1; uc001odt.1; uc009yqi.1 | *SLC25A45* | solute carrier family 25, member 45 | 5.56 | 5.66 | -1.07 | 0.0079 |
| TC12002967.hg.1 | AF088017 | *NAP1L1* | nucleosome assembly protein 1-like 1 | 5.47 | 5.26 | 1.16 | 0.0082 |
| TC01002517.hg.1 | NM_022756; ENST00000296214; ENST00000373073; ENST00000373074; ENST00000373075; ENST00000448519; ENST00000475828; AK225434; AK309663; BC016328; BC043239; BC056406; DQ099384; OTTHUMT00000012161; OTTHUMT00000012162; OTTHUMT00000012163; OTTHUMT00000012164; OTTHUMT00000012165; OTTHUMT00000012166; OTTHUMT00000012167; OTTHUMT00000091269 | *MEAF6* | MYST/Esa1-associated factor 6 | 5.63 | 5.73 | -1.07 | 0.0083 |
| TC22000022.hg.1 | OTTHUMT00000316085 | *CECR9* | cat eye syndrome chromosome region, candidate 9 (non-protein coding) | 2.53 | 2.67 | -1.1 | 0.0084 |
| TC08000396.hg.1 | ENST00000519714; ENST00000521446; BC048118 | *LOC101929488* | uncharacterized LOC101929488 | 3.32 | 3.38 | -1.04 | 0.0084 |
| TC19001051.hg.1 | NM_002378; NM_139354; NM_139355; ENST00000310132; ENST00000395040; ENST00000395045; BC000114; L18974; uc002lyt.3; uc002lyu.3; uc002lyv.3; uc010dtq.3 | *MATK* | megakaryocyte-associated tyrosine kinase | 5.8 | 5.95 | -1.11 | 0.0084 |
| TC19000238.hg.1 | NM_030818; ENST00000221554; ENST00000540216; AK293927; BC002905; uc002mxc.1; uc010xnf.2 | *CCDC130* | coiled-coil domain containing 130 | 6.45 | 6.62 | -1.12 | 0.0085 |
| TC01001351.hg.1 | NM_002001; ENST00000368114; ENST00000368115; BC005912; BC015195; OTTHUMT00000090328; OTTHUMT00000090329; uc001ftq.3 | *FCER1A* | Fc fragment of IgE, high affinity I, receptor for; alpha polypeptide | 5.43 | 4.89 | 1.45 | 0.0085 |
| TC22000796.hg.1 | NM_014876; ENST00000216039; ENST00000462610; BC015026; OTTHUMT00000321047; OTTHUMT00000321048; OTTHUMT00000321049; OTTHUMT00000321050; OTTHUMT00000321051; OTTHUMT00000321052; OTTHUMT00000321054; OTTHUMT00000321055; OTTHUMT00000321056; uc003awf.3 | *JOSD1* | Josephin domain containing 1 | 5.91 | 5.99 | -1.06 | 0.0085 |
| TC17002095.hg.1 | AL080124 | *TRIM16L* | tripartite motif containing 16-like | 3.8 | 3.96 | -1.12 | 0.0085 |
| TC12002538.hg.1 | AY166718; AY294627 | *CHPT1* | choline phosphotransferase 1 | 6.77 | 6.04 | 1.66 | 0.0086 |
| TC17001633.hg.1 | NM_016429; BC015924; uc002imy.3 | *COPZ2* | coatomer protein complex subunit zeta 2 | 4.18 | 4.27 | -1.07 | 0.0086 |
| TC04002039.hg.1 | AK094703 | *ADGRL3* | adhesion G protein-coupled receptor L3 | 1.54 | 1.35 | 1.15 | 0.0087 |
| TC17000119.hg.1 | NM_144607; ENST00000332439; AK125598; BC060779; uc002gjb.4 | *CYB5D1* | cytochrome b5 domain containing 1 | 4.15 | 4.24 | -1.06 | 0.0087 |
| TC17002558.hg.1 | AK090937 | *C17orf51* | chromosome 17 open reading frame 51 | 3.74 | 3.83 | -1.06 | 0.0088 |
| TC17002188.hg.1 | NR_036551 | *LOC100131347* | RAD52 motif containing 1 pseudogene | 4.25 | 4.39 | -1.1 | 0.0088 |
| TC16002068.hg.1 | NM_001030288; NM_003123; ENST00000360121; ENST00000395389; BC012350 | *SPN* | sialophorin | 6.93 | 7.15 | -1.17 | 0.0089 |
| TC0X000906.hg.1 | NR_024440; uc011mjh.1 | *LOC729609* | uncharacterized LOC729609 | 4.39 | 4.26 | 1.1 | 0.009 |
| TC03001694.hg.1 | BC032918 | *LOC101926983* | uncharacterized LOC101926983 | 1.61 | 1.51 | 1.07 | 0.009 |
| TC09000600.hg.1 | ENST00000450938 | *LOC101928797* | uncharacterized LOC101928797 | 2.05 | 1.96 | 1.06 | 0.009 |
| TC08001469.hg.1 | NR_030329; ENST00000385069; uc022azd.1 | *MIR599* | microRNA 599 | 2.55 | 2.17 | 1.3 | 0.009 |
| TC22000546.hg.1 | NM_013313; ENST00000339468; ENST00000403503; BC034486; BC048303; BC074501; OTTHUMT00000320245; OTTHUMT00000320246; OTTHUMT00000320247; uc002zvl.3; uc002zvm.3 | *YPEL1* | yippee like 1 | 5.52 | 5.59 | -1.05 | 0.0092 |
| TC19001758.hg.1 | NM_002257; ENST00000301420; AK293363; BC005313; uc002ptk.1; uc010ycg.1 | *KLK1* | kallikrein 1 | 4.81 | 4.99 | -1.13 | 0.0093 |
| TC13000111.hg.1 | NM_000059; ENST00000380152; ENST00000544455; BC047568; OTTHUMT00000046000; OTTHUMT00000046001; uc001uua.1; uc001uub.1 | *BRCA2* | breast cancer 2, early onset | 2.9 | 2.77 | 1.1 | 0.0094 |
| TC08002559.hg.1 | AF321824 | *LYNX1* | Ly6/neurotoxin 1 | 5 | 5.28 | -1.22 | 0.0094 |
| TC01006353.hg.1 | NM_001135553; NM_003684; NM_198973; NR_024174; NR_024176; ENST00000341183; ENST00000371945; ENST00000371946; ENST00000428112; ENST00000465783; ENST00000525888; AK096423; AK310566; BC002755; OTTHUMT00000021897; OTTHUMT00000021898; OTTHUMT00000021900; OTTHUMT00000021901; OTTHUMT00000021902; OTTHUMT00000021903; OTTHUMT00000021904; OTTHUMT00000021905; OTTHUMT00000021906; OTTHUMT00000021907; OTTHUMT00000021908; OTTHUMT00000021909; OTTHUMT00000021910; uc001cqd.2; uc009vyj.3 | *MKNK1* | MAP kinase interacting serine/threonine kinase 1 | 6.33 | 6.1 | 1.17 | 0.0094 |
| TC17000373.hg.1 | NM_001033566; NM_001033568; NM_018307; ENST00000333942; ENST00000354266; ENST00000358365; ENST00000394692; ENST00000545287; AK294407; AL136929; BC029029; BC060781; BC068463; BC092401; BC110895; BC125104; uc002hgv.3; uc002hgw.3; uc002hgx.3; uc002hgy.3; uc002hgz.3; uc002hha.3; uc002hhb.3; uc010csv.3; uc010wby.2 | *RHOT1* | ras homolog family member T1 | 6.85 | 6.66 | 1.14 | 0.0094 |
| TC0X001286.hg.1 | NM_001113490; NM_133265; NR_036255; ENST00000304758; ENST00000371958; ENST00000371959; ENST00000371962; ENST00000462114; ENST00000524145; AK301573; BC094712; BC130294; OTTHUMT00000057950; OTTHUMT00000057951; OTTHUMT00000096600; OTTHUMT00000096601; OTTHUMT00000378570; uc004epr.3; uc004eps.3; uc004ept.1; uc011mtc.1; uc022ccu.1 | *AMOT; MIR4329* | angiomotin; microRNA 4329 | 4.01 | 4.11 | -1.08 | 0.0095 |
| TC15001295.hg.1 | NR_034170; NR_034171; uc001ztu.3; uc021skh.1 | *EIF3J-AS1* | EIF3J antisense RNA 1 (head to head) | 5.25 | 5.12 | 1.09 | 0.0095 |
| TC10002958.hg.1 | NM_018425; ENST00000370631; AK309343; BC003167; OTTHUMT00000049735; uc001kog.1 | *PI4K2A* | phosphatidylinositol 4-kinase type 2 alpha | 5.92 | 6.04 | -1.09 | 0.0095 |
| TC01001831.hg.1 | NM_207468; ENST00000360827; ENST00000445590; AK125494; OTTHUMT00000092149; OTTHUMT00000092150; OTTHUMT00000092151; OTTHUMT00000316736; OTTHUMT00000316737; uc001hnt.3; uc009xeb.3 | *FAM177B* | family with sequence similarity 177, member B | 3.42 | 3.31 | 1.08 | 0.0096 |
| TC02004889.hg.1 | uc010zob.1 | *MGC16025* | uncharacterized LOC85009 | 5.35 | 5.2 | 1.11 | 0.0096 |
| TC19000886.hg.1 | NM_001145457; NM_001145458; NM_001242356; NM_001242357; NM_004829; ENST00000291890; ENST00000338835; ENST00000350790; ENST00000357397; AY346373; BC064806; uc002qib.2; uc002qic.2; uc002qid.2; uc002qie.2; uc002qif.2; uc010esj.2 | *NCR1* | natural cytotoxicity triggering receptor 1 | 6 | 6.27 | -1.2 | 0.0096 |
| TC20001680.hg.1 | AK094347 | *NELFCD* | negative elongation factor complex member C/D | 5.36 | 5.15 | 1.15 | 0.0096 |
| TC02001152.hg.1 | NM_006226; ENST00000428675; ENST00000437704; AK127514; BC101531; BC111985; OTTHUMT00000256113; OTTHUMT00000340210; uc002uuv.4; uc010fsp.3 | *PLCL1* | phospholipase C-like 1 | 4.87 | 4.68 | 1.13 | 0.0096 |
| TC11003322.hg.1 | NR_024239; NR_036562 | *CASP5* | caspase 5 | 6.34 | 5.84 | 1.41 | 0.0097 |
| TC04001570.hg.1 | NM_014331; ENST00000280612; BC012087; OTTHUMT00000257251; OTTHUMT00000364387; uc021xrw.1 | *SLC7A11* | solute carrier family 7 (anionic amino acid transporter light chain, xc- system), member 11 | 2.53 | 2.36 | 1.12 | 0.0097 |
| TC10002489.hg.1 | AK123677 | *STAM-AS1* | STAM antisense RNA 1 (head to head) | 2.63 | 2.83 | -1.15 | 0.0097 |
| TC07001579.hg.1 | NM_000927; ENST00000265724; ENST00000488737; ENST00000543898; AY425005; AY425006; BC130424; EU854148; OTTHUMT00000335444; OTTHUMT00000335739; OTTHUMT00000335740; OTTHUMT00000335741; OTTHUMT00000335742; OTTHUMT00000335743; OTTHUMT00000335744; OTTHUMT00000335745; OTTHUMT00000335746; uc003uiz.2; uc003uja.2; uc010lei.2; uc011khc.2 | *ABCB1* | ATP binding cassette subfamily B member 1 | 3.69 | 3.9 | -1.16 | 0.0098 |
| TC11000876.hg.1 | NM_007173; ENST00000280258; ENST00000531521; ENST00000533902; AK304301; AL832007; BC001278; BC063022; uc001pcb.3; uc001pcc.1; uc010rts.1 | *PRSS23* | protease, serine, 23 | 4.01 | 4.15 | -1.1 | 0.0098 |
| TC07002541.hg.1 | NR_002332 | *ST7-OT3* | ST7 overlapping transcript 3 | 3.64 | 3.77 | -1.09 | 0.0098 |
| TC11002074.hg.1 | NM_003355; ENST00000310473; ENST00000536983; ENST00000542615; BC011737; uc001oup.1 | *UCP2* | uncoupling protein 2 (mitochondrial, proton carrier) | 8.26 | 8.42 | -1.12 | 0.0098 |
| TC01006217.hg.1 | AK074383 | *GNG4* | guanine nucleotide binding protein (G protein), gamma 4 | 2.49 | 2.7 | -1.16 | 0.0099 |
| TC12002770.hg.1 | AY037867 | *LOH12CR2* | loss of heterozygosity, 12, chromosomal region 2 (non-protein coding) | 2.62 | 2.74 | -1.09 | 0.0099 |
| TC08001264.hg.1 | NM_001164750; NM_001164751; NM_001164752; NM_001164753; NM_001164754; NM_001164755; NM_001164756; NM_004318; NM_020164; NM_032466; NM_032467; NM_032468; ENST00000356457; ENST00000379449; ENST00000379454; ENST00000389204; ENST00000445642; ENST00000517661; ENST00000517847; ENST00000517856; ENST00000517903; ENST00000518068; ENST00000519234; ENST00000522603; ENST00000522835; ENST00000522919; ENST00000523897; ENST00000541428; FJ461473; OTTHUMT00000378510; OTTHUMT00000378511; OTTHUMT00000378573; OTTHUMT00000378574; OTTHUMT00000378575; OTTHUMT00000378576; OTTHUMT00000378578; OTTHUMT00000378580; OTTHUMT00000378581; OTTHUMT00000378582; OTTHUMT00000378583; OTTHUMT00000378584; OTTHUMT00000378585; OTTHUMT00000378586; OTTHUMT00000378604; OTTHUMT00000378605; OTTHUMT00000378606; OTTHUMT00000378607; OTTHUMT00000378608; OTTHUMT00000378609; OTTHUMT00000378610; OTTHUMT00000378611; OTTHUMT00000378612; OTTHUMT00000378613; OTTHUMT00000378614; OTTHUMT00000378615; OTTHUMT00000378616; uc003xuj.3; uc003xul.3; uc003xum.3; uc003xun.3; uc003xuo.2; uc003xur.3; uc011leg.2; uc011leh.2; uc011lei.2; uc011lej.2; uc011lek.2; uc011lel.2; uc011lem.2 | *ASPH* | aspartate beta-hydroxylase | 4.35 | 4.1 | 1.19 | 0.0101 |
| TC04000360.hg.1 | ENST00000408636 | *MIR1269A* | microRNA 1269a | 5.26 | 5.51 | -1.19 | 0.0101 |
| TC14000882.hg.1 | NR_002312; uc001vxa.1 | *RPPH1* | ribonuclease P RNA component H1 | 7.08 | 6.85 | 1.17 | 0.0101 |
| TC01003455.hg.1 | NM_053282; ENST00000367929; ENST00000493550; BC022407; BC066595; OTTHUMT00000076794; OTTHUMT00000076795; uc001gbz.1; uc001gca.1 | *SH2D1B* | SH2 domain containing 1B | 4.79 | 5.38 | -1.51 | 0.0101 |
| TC17002770.hg.1 | AK093963 | *KCNJ2-AS1* | KCNJ2 antisense RNA 1 (head to head) | 5.13 | 4.85 | 1.21 | 0.0102 |
| TC04001465.hg.1 | NM_030821; ENST00000243501; ENST00000502283; ENST00000502772; AF332892; BC017218; OTTHUMT00000254868; OTTHUMT00000363795; OTTHUMT00000363796; OTTHUMT00000363797; uc003hzp.3; uc010img.3 | *PLA2G12A* | phospholipase A2, group XIIA | 4.7 | 4.63 | 1.05 | 0.0102 |
| TC04000396.hg.1 | NM_001098484; NM_001134742; NM_003759; ENST00000264485; ENST00000340595; ENST00000351898; ENST00000425175; ENST00000512686; ENST00000514331; AF004813; AF069510; AF157492; BC030977; CR749482; OTTHUMT00000252158; OTTHUMT00000362090; OTTHUMT00000362091; OTTHUMT00000362107; OTTHUMT00000362108; uc003hfy.3; uc003hfz.3; uc003hga.2; uc003hgb.3; uc003hgc.4; uc010iib.3; uc010iic.3; uc010iid.3 | *SLC4A4* | solute carrier family 4 (sodium bicarbonate cotransporter), member 4 | 3.43 | 3.63 | -1.15 | 0.0102 |
| TC01001356.hg.1 | NM_001004310; ENST00000321935; ENST00000339348; ENST00000368106; ENST00000392235; AK131201; AK301270; AY212514; AY654627; OTTHUMT00000085593; OTTHUMT00000276852; OTTHUMT00000276853; OTTHUMT00000276854; uc001fuc.2; uc001fud.4; uc009wsz.1; uc009wta.3; uc010pix.1 | *FCRL6* | Fc receptor-like 6 | 6.44 | 7.01 | -1.48 | 0.0103 |
| TC14001956.hg.1 | NR_038356 | *LOC100506071* | uncharacterized LOC100506071 | 2.91 | 2.72 | 1.14 | 0.0104 |
| TC13000215.hg.1 | NR_039852; uc021rju.1 | *MIR4703* | microRNA 4703 | 1.42 | 1.26 | 1.11 | 0.0104 |
| TC11001116.hg.1 | NM_019604; ENST00000227348; ENST00000533709; AB209830; BC070266; uc001pyj.3; uc001pyk.3 | *CRTAM* | cytotoxic and regulatory T-cell molecule | 3.46 | 3.69 | -1.17 | 0.0105 |
| TC08002510.hg.1 | U50573 | *MTSS1* | metastasis suppressor 1 | 6.31 | 6.63 | -1.24 | 0.0105 |
| TC22000144.hg.1 | NM_001199281; NM_001201429; NM_012295; ENST00000263119; ENST00000337989; ENST00000398319; ENST00000405822; ENST00000485008; AK307522; AL390166; BC013099; BC054497; OTTHUMT00000320161; OTTHUMT00000320162; OTTHUMT00000320163; OTTHUMT00000320164; OTTHUMT00000320165; OTTHUMT00000320166; OTTHUMT00000320167; OTTHUMT00000320169; OTTHUMT00000320170; OTTHUMT00000320171; OTTHUMT00000320172; OTTHUMT00000320278; OTTHUMT00000340949; uc002zzi.1; uc002zzj.1; uc002zzk.2; uc002zzl.2; uc002zzm.1; uc010guk.1; uc010gul.1; uc021wnc.1 | *CABIN1* | calcineurin binding protein 1 | 6.21 | 6.28 | -1.05 | 0.0107 |
| TC10000459.hg.1 | NR_038373; ENST00000440197; OTTHUMT00000048622; OTTHUMT00000048635; uc021ptm.1 | *DNAJC9-AS1* | DNAJC9 antisense RNA 1 | 2.5 | 2.61 | -1.07 | 0.0108 |
| TC10000469.hg.1 | NM_173540; ENST00000372841; ENST00000394790; ENST00000465695; AK095482; BC036037; BC100994; BC100995; BC100996; BC100997; OTTHUMT00000048689; OTTHUMT00000048690; OTTHUMT00000048692; uc001juy.1; uc001juz.1; uc001jva.3 | *FUT11* | fucosyltransferase 11 (alpha (1,3) fucosyltransferase) | 5.77 | 5.97 | -1.15 | 0.0108 |
| TC19000055.hg.1 | NM_015675; ENST00000215631; AY615270; BC113466; uc002lwb.2; uc002lwc.1 | *GADD45B* | growth arrest and DNA-damage-inducible, beta | 6.9 | 6.74 | 1.12 | 0.0108 |
| TC17001339.hg.1 | NR_039886; uc021tui.1 | *MIR4733* | microRNA 4733 | 3.81 | 3.66 | 1.11 | 0.0108 |
| TC01003113.hg.1 | NM_007053; ENST00000235933; ENST00000369288; ENST00000401557; AK128370; BC014465; EU016101; OTTHUMT00000038531; OTTHUMT00000038532; uc001eol.1; uc001eom.1; uc010oyz.1 | *CD160* | CD160 molecule | 4.7 | 5.03 | -1.26 | 0.0109 |
| TC09001205.hg.1 | NM_001025780; NM_016014; ENST00000333421; ENST00000377041; BC038390; BC044576; OTTHUMT00000052624; OTTHUMT00000052625; uc004ail.3; uc004aim.1 | *ABHD17B* | abhydrolase domain containing 17B | 5.83 | 5.93 | -1.07 | 0.011 |
| TC12001626.hg.1 | ENST00000385293 | *MIR616* | microRNA 616 | 2.27 | 2.06 | 1.16 | 0.0111 |
| TC15000664.hg.1 | NM_001172623; NM_001172624; NM_002499; ENST00000261908; ENST00000339362; ENST00000558964; ENST00000560262; ENST00000560328; ENST00000560352; BC117161; BC143270; BC143271; BC143279; OTTHUMT00000257472; uc002avm.4; uc010ukx.2; uc010uky.2; uc010ukz.2 | *NEO1* | neogenin 1 | 4.69 | 4.86 | -1.12 | 0.0112 |
| TC05003038.hg.1 | L26969 | *PIK3R1* | phosphoinositide-3-kinase, regulatory subunit 1 (alpha) | 2.27 | 2.39 | -1.09 | 0.0112 |
| TC21000398.hg.1 | NR_002996; ENST00000363922 | *SNORA80A* | small nucleolar RNA, H/ACA box 80A | 5.41 | 5.64 | -1.17 | 0.0112 |
| TC16000349.hg.1 | NM_001114380; NM_002209; ENST00000356798; ENST00000358164; ENST00000433423; AK298674; AK303190; BC008777; uc002dyi.4; uc002dyj.4; uc010veu.1; uc010vev.2 | *ITGAL* | integrin alpha L | 8.53 | 8.72 | -1.14 | 0.0113 |
| TC06001280.hg.1 | NM_001242628; NM_001242629; NM_001242630; NM_018988; NR_038459; ENST00000379278; ENST00000379284; ENST00000379287; AF264036; BC066553; BC119005; BC148295; OTTHUMT00000039899; OTTHUMT00000039902; OTTHUMT00000039903; uc003nas.2; uc003nat.2; uc003nau.3; uc003nav.3; uc021ylt.1; uc021ylu.1 | *GFOD1* | glucose-fructose oxidoreductase domain containing 1 | 4.99 | 5.22 | -1.17 | 0.0114 |
| TC07001171.hg.1 | ENST00000408466 | *MIR1302-6* | microRNA 1302-6 | 1.35 | 1.22 | 1.1 | 0.0115 |
| TC0X001610.hg.1 | AK097484 | *REPS2* | RALBP1 associated Eps domain containing 2 | 5.68 | 5.23 | 1.37 | 0.0115 |
| TC11001034.hg.1 | ENST00000539685; ENST00000540545; ENST00000543182 | *LOC105369507* | uncharacterized LOC105369507 | 1.26 | 1.15 | 1.08 | 0.0116 |
| TC11000105.hg.1 | NM_001004755; ENST00000321543; OTTHUMT00000142812; uc010qyu.2 | *OR51L1* | olfactory receptor, family 51, subfamily L, member 1 | 1.83 | 2.04 | -1.15 | 0.0116 |
| TC06000015.hg.1 | ENST00000457049 | *HMGN2P28* | high mobility group nucleosomal binding domain 2 pseudogene 28 | 6.41 | 6.56 | -1.11 | 0.0118 |
| TC19001497.hg.1 | NM_001039671; NM_001039672; NM_001039673; NM_001145461; NM_001145462; NM_001145463; NM_033557; ENST00000329420; ENST00000337679; ENST00000339413; ENST00000392124; BC014974; BC091477; uc002ohw.2; uc002ohx.2; uc002ohy.2; uc002ohz.2; uc002oia.2; uc002oib.3; uc010xtx.1; uc010xty.1 | *YIF1B* | Yip1 interacting factor homolog B (S. cerevisiae) | 7.14 | 7.07 | 1.06 | 0.0118 |
| TC11000505.hg.1 | NM_000139; ENST00000278888; AK310142; BC074800; JF411082; uc001nop.3; uc009ymu.3; uc021qka.1 | *MS4A2* | membrane-spanning 4-domains, subfamily A, member 2 | 4.36 | 3.6 | 1.69 | 0.0119 |
| TC07000491.hg.1 | NR_029411; ENST00000418663; BC078169; BC090058; uc003ufs.2 | *LOC100133091* | uncharacterized LOC100133091 | 6.2 | 6.33 | -1.09 | 0.012 |
| TC11002819.hg.1 | NR_038146 | *TRIM51EP* | tripartite motif-containing 51E, pseudogene | 4.87 | 4.33 | 1.46 | 0.012 |
| TC19000581.hg.1 | NM_001098506; NM_033543; ENST00000187608; ENST00000401445; ENST00000407170; ENST00000482870; AK023602; BC012001; BC106727; OTTHUMT00000321136; OTTHUMT00000321137; OTTHUMT00000321138; OTTHUMT00000321139; OTTHUMT00000321140; OTTHUMT00000321144; uc002orc.1; uc002ore.4; uc002orf.2; uc002org.4 | *CEACAM21* | carcinoembryonic antigen-related cell adhesion molecule 21 | 4.87 | 4.76 | 1.08 | 0.0121 |
| TC04002601.hg.1 | AF352781 | *CXCL11* | chemokine (C-X-C motif) ligand 11 | 2.37 | 2.24 | 1.09 | 0.0121 |
| TC20000537.hg.1 | NM_001037732; ENST00000334391; BC140936; OTTHUMT00000101361; uc002wcz.1 | *DEFB128* | defensin, beta 128 | 2.17 | 2.33 | -1.11 | 0.0121 |
| TC04002440.hg.1 | BC035129 | *LOC100134937* | uncharacterized LOC100134937 | 5.37 | 5.21 | 1.12 | 0.0121 |
| TC15002768.hg.1 | NR_037600; NR_037601; NR_037602; AK024092 | *LINC01578* | long intergenic non-protein coding RNA 1578 | 7.04 | 6.81 | 1.17 | 0.0122 |
| TC19000644.hg.1 | NM_001199867; NM_031417; ENST00000262891; ENST00000300843; AK074578; BC071948; uc002paz.2; uc002pba.2; uc002pbb.2; uc002pbc.1 | *MARK4* | MAP/microtubule affinity-regulating kinase 4 | 6.2 | 6.32 | -1.09 | 0.0122 |
| TC09002677.hg.1 | AB233423 | *PTCH1* | patched 1 | 5.88 | 6.28 | -1.32 | 0.0122 |
| TC07000250.hg.1 | NM_005402; ENST00000005257; ENST00000468201; BC039858; OTTHUMT00000250696; OTTHUMT00000258922; OTTHUMT00000339288; OTTHUMT00000339289; OTTHUMT00000339290; uc003thd.3 | *RALA* | v-ral simian leukemia viral oncogene homolog A (ras related) | 4.48 | 4.57 | -1.07 | 0.0122 |
| TC01001236.hg.1 | NM_001025231; OTTHUMT00000034522; uc001fal.1 | *KPRP* | keratinocyte proline-rich protein | 4.53 | 4.78 | -1.18 | 0.0123 |
| TC11000325.hg.1 | NM_001144030; NM_024662; ENST00000257829; ENST00000527971; ENST00000531159; ENST00000532555; BC035558; uc001mvk.3; uc010ren.2 | *NAT10* | N-acetyltransferase 10 (GCN5-related) | 5.93 | 6.01 | -1.06 | 0.0123 |
| TC17001895.hg.1 | NM_052916; ENST00000269391; ENST00000319945; OTTHUMT00000255874; uc002jqz.3 | *RNF157* | ring finger protein 157 | 5.65 | 5.49 | 1.12 | 0.0123 |
| TC11003236.hg.1 | uc001oqx.1 | *ALG1L9P* | asparagine-linked glycosylation 1-like 9, pseudogene | 4.79 | 4.95 | -1.11 | 0.0124 |
| TC02000760.hg.1 | NM_002881; ENST00000272519; ENST00000420510; ENST00000470417; AK127675; AK303214; AK304588; BC018163; OTTHUMT00000254232; OTTHUMT00000332358; OTTHUMT00000332359; OTTHUMT00000332362; OTTHUMT00000338460; OTTHUMT00000338461; OTTHUMT00000338462; uc002tmk.3; uc002tml.3; uc010yys.2; uc010yyt.2 | *RALB* | v-ral simian leukemia viral oncogene homolog B | 7.9 | 7.78 | 1.09 | 0.0124 |
| TC08002164.hg.1 | NR_026974 | *ZNF252P-AS1* | ZNF252P antisense RNA 1 | 4.06 | 3.94 | 1.08 | 0.0124 |
| TC02001604.hg.1 | NM_001099218; ENST00000399080; AK310498; OTTHUMT00000323801; uc002rcl.1; uc010exn.1 | *RAD51AP2* | RAD51 associated protein 2 | 1.55 | 1.49 | 1.05 | 0.0126 |
| TC04002941.hg.1 | NM_031950; ENST00000259989; BC025720; OTTHUMT00000250324; uc003gon.3 | *FGFBP2* | fibroblast growth factor binding protein 2 | 6.94 | 7.45 | -1.42 | 0.0127 |
| TC01001228.hg.1 | NM_178434; ENST00000333881; BC130365; BC130367; OTTHUMT00000040061; uc001fac.2 | *LCE3C* | late cornified envelope 3C | 2.64 | 2.8 | -1.11 | 0.0127 |
| TC10002925.hg.1 | NM_001253908; NM_001253909; NM_003739; ENST00000380554; ENST00000439082; ENST00000470862; AK296829; BC001479; BC019230; OTTHUMT00000046533; OTTHUMT00000046534; OTTHUMT00000046535; OTTHUMT00000046536; uc001ihu.3; uc010qap.2; uc010qaq.1; uc021pml.1 | *AKR1C3* | aldo-keto reductase family 1, member C3 | 3.65 | 3.84 | -1.14 | 0.0128 |
| TC10000653.hg.1 | NM_173497; NM_182765; ENST00000298068; ENST00000371667; ENST00000371681; ENST00000446394; ENST00000498446; AK122629; AK295723; BC036487; BC040187; BX537856; OTTHUMT00000049367; OTTHUMT00000049369; OTTHUMT00000049370; OTTHUMT00000098620; uc001khk.2; uc001khl.2; uc001khm.2; uc001khn.1; uc009xty.1; uc010qnm.1 | *HECTD2* | HECT domain containing E3 ubiquitin protein ligase 2 | 1.72 | 1.64 | 1.06 | 0.0128 |
| TC01001229.hg.1 | NM_178433; ENST00000335633; OTTHUMT00000034515; uc010pds.2 | *LCE3B* | late cornified envelope 3B | 6.3 | 6.4 | -1.07 | 0.0128 |
| TC0X002269.hg.1 | NR_027455 | *LINC00893* | long intergenic non-protein coding RNA 893 | 5.14 | 5.23 | -1.06 | 0.0128 |
| TC12001295.hg.1 | NM_021094; NM_134431; ENST00000307378; ENST00000458504; ENST00000473830; AF085224; AK296044; AK316249; BC042452; BC144256; OTTHUMT00000343648; OTTHUMT00000343649; OTTHUMT00000343650; OTTHUMT00000343651; OTTHUMT00000343652; OTTHUMT00000343665; OTTHUMT00000343666; OTTHUMT00000343667; OTTHUMT00000343690; OTTHUMT00000343691; OTTHUMT00000343692; OTTHUMT00000343693; OTTHUMT00000343694; OTTHUMT00000343695; OTTHUMT00000343696; uc001rer.3; uc001res.3; uc001ret.3; uc001reu.2; uc010sio.2; uc010sip.2; uc010siq.2 | *SLCO1A2* | solute carrier organic anion transporter family, member 1A2 | 1.63 | 1.58 | 1.03 | 0.0128 |
| TC18000286.hg.1 | NR_026659; uc010wyu.2 | *LOC727896* | cysteine and histidine rich domain containing 1 pseudogene | 5.97 | 5.8 | 1.12 | 0.0129 |
| TC11000474.hg.1 | NM_001005186; BC151146; BC151147; uc010rjz.2 | *OR6Q1* | olfactory receptor, family 6, subfamily Q, member 1 (gene/pseudogene) | 2.95 | 3.15 | -1.14 | 0.0129 |
| TC19000647.hg.1 | NM_001114171; NM_006732; ENST00000353609; ENST00000417353; ENST00000443841; AK225070; BC036724; BC040197; EU178110; EU178111; EU178112; EU178113; EU178114; EU178115; EU178116; uc002pbw.3; uc002pbx.4; uc002pby.4; uc010eka.1; uc010ekb.1; uc010ekd.1; uc010eke.3; uc010ekf.3; uc010ekg.3 | *FOSB* | FBJ murine osteosarcoma viral oncogene homolog B | 4.59 | 4.75 | -1.11 | 0.013 |
| TC05001033.hg.1 | NM_004499; NM_031266; ENST00000355836; ENST00000358344; ENST00000504898; ENST00000506259; ENST00000506339; ENST00000514633; ENST00000515193; AK054600; BC001616; BC004561; BC009359; BC036708; OTTHUMT00000373519; OTTHUMT00000373520; OTTHUMT00000373521; OTTHUMT00000373522; OTTHUMT00000373523; OTTHUMT00000373524; OTTHUMT00000373526; OTTHUMT00000373527; uc003miu.3; uc003miv.3; uc003mix.3 | *HNRNPAB* | heterogeneous nuclear ribonucleoprotein A/B | 6.59 | 6.7 | -1.08 | 0.013 |
| TC01006348.hg.1 | NM_001136265; ENST00000455833; OTTHUMT00000007099; OTTHUMT00000007100; uc001bbd.2 | *IFFO2* | intermediate filament family orphan 2 | 5.73 | 5.87 | -1.1 | 0.013 |
| TC17001833.hg.1 | NR_036534; uc021uci.1 | *KCNJ2-AS1* | KCNJ2 antisense RNA 1 (head to head) | 5.38 | 5.15 | 1.18 | 0.013 |
| TC13000351.hg.1 | NM_001144072; NM_177967; NR_026644; ENST00000376440; ENST00000403766; ENST00000460562; AK075516; AK124404; AK300908; BC053346; BC121138; BC121139; OTTHUMT00000045587; OTTHUMT00000045588; OTTHUMT00000045589; OTTHUMT00000045590; OTTHUMT00000045591; OTTHUMT00000045592; OTTHUMT00000045593; OTTHUMT00000045594; OTTHUMT00000045595; OTTHUMT00000045596; OTTHUMT00000045597; uc001voa.4; uc001vob.4; uc001voc.3; uc001vod.3; uc001voh.3; uc010tiu.2; uc010tiv.2; uc010tiw.2 | *UBAC2* | UBA domain containing 2 | 6.52 | 6.47 | 1.04 | 0.013 |
| TC11001502.hg.1 | X57147 | *ERV9-1* | endogenous retrovirus group 9, member 1 | 3.96 | 3.87 | 1.07 | 0.0132 |
| TC05000641.hg.1 | NM_000589; NM_172348; ENST00000231449; ENST00000350025; ENST00000495905; BC066277; BC067514; BC070123; OTTHUMT00000132786; OTTHUMT00000132787; OTTHUMT00000157346 | *IL4* | interleukin 4 | 2.7 | 2.61 | 1.06 | 0.0132 |
| TC17001520.hg.1 | NM_001096; NM_198830; ENST00000352035; ENST00000353196; ENST00000393896; ENST00000537919; AB210035; AK295675; AK304802; BC006195; OTTHUMT00000257465; OTTHUMT00000257466; uc002hyg.3; uc002hyh.3; uc002hyi.3; uc010wfx.2; uc010wfy.2 | *ACLY* | ATP citrate lyase | 6.99 | 7.14 | -1.11 | 0.0133 |
| TC02001247.hg.1 | NR_002763; uc002vef.3 | *CPS1-IT1* | CPS1 intronic transcript 1 | 1.64 | 1.53 | 1.08 | 0.0133 |
| TC01000173.hg.1 | NM_001045480; BC144662; uc001aux.3 | *PRAMEF16; PRAMEF17* | PRAME family member 16; PRAME family member 17 | 3.39 | 3.32 | 1.04 | 0.0133 |
| TC22001311.hg.1 | X77690 | *TIMP3* | TIMP metallopeptidase inhibitor 3 | 1.97 | 1.79 | 1.14 | 0.0133 |
| TC16001442.hg.1 | uc010bwr.1 | *CRYM-AS1* | CRYM antisense RNA 1 | 2.3 | 2.23 | 1.05 | 0.0134 |
| TC20000371.hg.1 | ENST00000413818; BC047609 | *LOC101927377* | uncharacterized LOC101927377 | 3.18 | 3.12 | 1.04 | 0.0134 |
| TC11000884.hg.1 | BC038205 | *LOC101929174* | uncharacterized LOC101929174 | 1.75 | 1.63 | 1.09 | 0.0134 |
| TC19001233.hg.1 | NM_001008701; NM_014921; ENST00000340736; ENST00000361434 | *ADGRL1* | adhesion G protein-coupled receptor L1 | 5.4 | 5.5 | -1.07 | 0.0135 |
| TC09002192.hg.1 | AF131806 | *RGS3* | regulator of G-protein signaling 3 | 5.16 | 5.59 | -1.35 | 0.0135 |
| TC17001548.hg.1 | ENST00000363640 | *RNY4P2* | RNA, Ro-associated Y4 pseudogene 2 | 4.29 | 3.82 | 1.39 | 0.0135 |
| TC07000972.hg.1 | ENST00000516507 | *RNY4* | RNA, Ro-associated Y4 | 5.34 | 4.86 | 1.39 | 0.0136 |
| TC06002432.hg.1 | AF043342; AF098751 | *TNF* | tumor necrosis factor | 1.31 | 1.42 | -1.08 | 0.0136 |
| TC11001867.hg.1 | NM_001620; NM_024060; ENST00000257247; ENST00000378024; ENST00000525875; ENST00000530124; uc001ntl.3 | *AHNAK* | AHNAK nucleoprotein | 7.36 | 7.52 | -1.12 | 0.0137 |
| TC17001151.hg.1 | ENST00000423323; OTTHUMT00000129955 | *COX10-AS1* | COX10 antisense RNA 1 | 1.9 | 2.07 | -1.12 | 0.0138 |
| TC0X001037.hg.1 | NM_001013742; BC137319; OTTHUMT00000368187; uc010njr.2 | *DGKK* | diacylglycerol kinase, kappa | 3.24 | 3.61 | -1.3 | 0.0138 |
| TC02000414.hg.1 | NM_006196; ENST00000303577; BC039742; OTTHUMT00000251844; uc002sgf.3 | *PCBP1* | poly(rC) binding protein 1 | 7.74 | 7.66 | 1.06 | 0.0138 |
| TC16001236.hg.1 | NM_006927; ENST00000342907; ENST00000393640; AK127322; BC036777; OTTHUMT00000268968; uc002eyw.2; uc002eyx.2 | *ST3GAL2* | ST3 beta-galactoside alpha-2,3-sialyltransferase 2 | 6.58 | 6.48 | 1.08 | 0.0138 |
| TC06003415.hg.1 | M25325; M83770 | *HLA-DQB1* | major histocompatibility complex, class II, DQ beta 1 | 3.22 | 3.13 | 1.06 | 0.0139 |
| TC11000507.hg.1 | NM_139249; ENST00000300182; BC069322; BC101783; uc001npd.3 | *MS4A6E* | membrane-spanning 4-domains, subfamily A, member 6E | 1.69 | 1.59 | 1.07 | 0.0139 |
| TC12000406.hg.1 | NM_001039960; NM_004858; ENST00000319957; ENST00000358657; ENST00000453097; ENST00000514353; ENST00000535225; ENST00000546663; ENST00000547697; ENST00000551071; AK128321; AK295315; AL831915; BC025994; DQ975204; DQ996398; DQ996537; uc001ryp.1; uc001ryq.4; uc001ryr.3; uc010snk.2 | *SLC4A8* | solute carrier family 4, sodium bicarbonate cotransporter, member 8 | 4.01 | 4.08 | -1.05 | 0.014 |
| TC01002972.hg.1 | NM_006402; ENST00000256644; ENST00000474861; ENST00000483260; BC062619; OTTHUMT00000031966; OTTHUMT00000031967; OTTHUMT00000031968; OTTHUMT00000035929; uc001dzr.3 | *LAMTOR5* | late endosomal/lysosomal adaptor, MAPK and MTOR activator 5 | 5.07 | 4.97 | 1.07 | 0.0141 |
| TC01002475.hg.1 | NM_003680; ENST00000373477; ENST00000469100; AK127182; BC001933; BC016689; BC035242; OTTHUMT00000011225; OTTHUMT00000011226; OTTHUMT00000011227; OTTHUMT00000011228; OTTHUMT00000011229; OTTHUMT00000011230; OTTHUMT00000011231; OTTHUMT00000011232; OTTHUMT00000011233; uc001bvw.1; uc001bvx.1; uc001bvy.1 | *YARS* | tyrosyl-tRNA synthetase | 5.19 | 5.32 | -1.1 | 0.0141 |
| TC0Y000069.hg.1 | NM_001001877; NM_033108; NM_152584; NM_153716; NR_003509; NR_003510; ENST00000307393; ENST00000309834; ENST00000382856; BC036567; BC055414; BC117380; OTTHUMT00000100036; OTTHUMT00000100037; OTTHUMT00000100038; OTTHUMT00000100039; uc004ftp.3; uc004ftq.3; uc004ftr.3 | *HSFY2; HSFY1* | heat shock transcription factor, Y-linked 2; heat shock transcription factor, Y-linked 1 | 1.59 | 1.55 | 1.03 | 0.0142 |
| TC22000825.hg.1 | NM_016272; ENST00000327492; BC028919; BC038957; OTTHUMT00000320699; OTTHUMT00000320700; uc003azz.1 | *TOB2* | transducer of ERBB2, 2 | 6.52 | 6.69 | -1.13 | 0.0143 |
| TC18000513.hg.1 | NM_001101654; NM_014593; ENST00000285106; ENST00000412036; AB209883; BC009188; BC014940; BC015733; OTTHUMT00000255927; uc002lep.4; uc002leq.4; uc002ler.4; uc010doy.3 | *CXXC1* | CXXC finger protein 1 | 5.82 | 5.91 | -1.07 | 0.0144 |
| TC01000293.hg.1 | NR_036057; uc021oid.1 | *MIR3115* | microRNA 3115 | 2.37 | 2.24 | 1.1 | 0.0145 |
| TC10000749.hg.1 | NM_004741; ENST00000370007; ENST00000405356; ENST00000488254; AK056755; AY820769; BC001883; BC006769; D21262; OTTHUMT00000050012; OTTHUMT00000050013; OTTHUMT00000050014; OTTHUMT00000050015; OTTHUMT00000050017; uc001kuo.2; uc001kup.2; uc001kuq.2; uc001kur.2; uc009xxb.1 | *NOLC1* | nucleolar and coiled-body phosphoprotein 1 | 5.44 | 5.55 | -1.07 | 0.0145 |
| TC0X001112.hg.1 | NM_002565; ENST00000374519; BC095503; BC096067; OTTHUMT00000057058; uc004dxz.1 | *P2RY4* | pyrimidinergic receptor P2Y, G-protein coupled, 4 | 4.46 | 4.39 | 1.05 | 0.0145 |
| TC01004961.hg.1 | BC038769 | *LOC101929441* | uncharacterized LOC101929441 | 3.03 | 3.23 | -1.14 | 0.0146 |
| TC22001488.hg.1 | NM_001202502; NM_003634; ENST00000216121; ENST00000494966; BC002371; OTTHUMT00000322117; OTTHUMT00000322118; OTTHUMT00000322119; OTTHUMT00000322120; OTTHUMT00000322121; OTTHUMT00000322123; uc003afx.4; uc011akp.2 | *NIPSNAP1* | nipsnap homolog 1 (C. elegans) | 5.21 | 5.28 | -1.05 | 0.0146 |
| TC02004751.hg.1 | AK001864 | *STAT4* | signal transducer and activator of transcription 4 | 2.05 | 2.19 | -1.11 | 0.0147 |
| TC11000025.hg.1 | NM_001042463; NM_174940; ENST00000397510; ENST00000397512; ENST00000488769; ENST00000526170; AK302585; BC008671; OTTHUMT00000257104; OTTHUMT00000348225; OTTHUMT00000348226; OTTHUMT00000348227 | *TMEM80* | transmembrane protein 80 | 6.27 | 6.2 | 1.05 | 0.0147 |
| TC09001566.hg.1 | NM_006626; ENST00000373659; BC037282; OTTHUMT00000053962 | *ZBTB6* | zinc finger and BTB domain containing 6 | 4.77 | 4.61 | 1.12 | 0.0147 |
| TC0X000469.hg.1 | NM_014467; ENST00000373004; ENST00000481988; BC020733; OTTHUMT00000057486; OTTHUMT00000057487; uc004egb.3 | *SRPX2* | sushi-repeat containing protein, X-linked 2 | 3.36 | 3.46 | -1.07 | 0.0149 |
| TC6_ssto_hap7000022.hg.1 | NM_014596; NM_170783; BC010898; BC050608; OTTHUMT00000140718; OTTHUMT00000140719; OTTHUMT00000140720; OTTHUMT00000315222; OTTHUMT00000315223; OTTHUMT00000315224; uc011jel.2; uc011jem.2 | *ZNRD1* | zinc ribbon domain containing 1 | 4.73 | 4.65 | 1.06 | 0.0149 |
| TC22001177.hg.1 | AK092640 | *ADM2* | adrenomedullin 2 | 4.08 | 4.25 | -1.12 | 0.015 |
| TC11000623.hg.1 | NM_006779; ENST00000279249; ENST00000533419; BC022337; BC075834; uc001odl.3 | *CDC42EP2* | CDC42 effector protein (Rho GTPase binding) 2 | 7.39 | 7.26 | 1.09 | 0.015 |
| TC01005287.hg.1 | BC012991; ENST00000317122; NR_024279 | *FLJ37453* | uncharacterized LOC729614 | 5.25 | 5.14 | 1.08 | 0.015 |
| TC12000152.hg.1 | NM_016523; ENST00000354855; ENST00000537723; AF175207; AF267244; AF267245; BC096734; BC098166; BC098257; BC098354; uc001qwm.3; uc009zgw.3; uc009zgx.3; uc009zgy.3; uc009zgz.3; uc009zha.3; uc021qux.1 | *KLRF1* | killer cell lectin-like receptor subfamily F, member 1 | 5.35 | 5.98 | -1.55 | 0.015 |
| TC11002692.hg.1 | X66187 | *MS4A2* | membrane-spanning 4-domains, subfamily A, member 2 | 5.43 | 4.55 | 1.83 | 0.015 |
| TC17002014.hg.1 | NR_033795 | *OR1D4* | olfactory receptor, family 1, subfamily D, member 4 (gene/pseudogene) | 4.53 | 4.65 | -1.09 | 0.015 |
| TC02004178.hg.1 | AK095013 | *SLC8A1* | solute carrier family 8 (sodium/calcium exchanger), member 1 | 3.87 | 3.56 | 1.24 | 0.015 |
| TC01002727.hg.1 | NM_033407; ENST00000251157; ENST00000340370; ENST00000404627; ENST00000489185; AB051558; AK292640; AK299737; BC041813; DQ118679; DQ118680; DQ341187; OTTHUMT00000036806; OTTHUMT00000036807; OTTHUMT00000036808; OTTHUMT00000036809; OTTHUMT00000036810; OTTHUMT00000036811; OTTHUMT00000036812; uc001dam.3; uc001dar.1; uc010oov.1 | *DOCK7* | dedicator of cytokinesis 7 | 3.35 | 3.49 | -1.1 | 0.0151 |
| TC05002377.hg.1 | AK095157 | *LOC105378967* | uncharacterized LOC105378967 | 3.39 | 3.24 | 1.11 | 0.0151 |
| TC0Y000335.hg.1 | uc004fvy.1 | *TTTY3* | testis-specific transcript, Y-linked 3 (non-protein coding) | 1.83 | 1.95 | -1.09 | 0.0151 |
| TC06000341.hg.1 | NM_001025091; NM_001090; ENST00000326195; ENST00000376545; AK307551; BC034488; BC112923; OTTHUMT00000076136; OTTHUMT00000076137; OTTHUMT00000076138; OTTHUMT00000076140; OTTHUMT00000256168; OTTHUMT00000256169; uc003nqk.2; uc003nql.3; uc003nqm.3 | *ABCF1* | ATP binding cassette subfamily F member 1 | 6.53 | 6.65 | -1.09 | 0.0152 |
| TC22001426.hg.1 | NM_014508; ENST00000361441; BC011739; OTTHUMT00000321241; OTTHUMT00000321242; uc003awr.3 | *APOBEC3C* | apolipoprotein B mRNA editing enzyme, catalytic polypeptide-like 3C | 7.74 | 7.97 | -1.17 | 0.0152 |
| TC17002658.hg.1 | NR_036474 | *GPATCH8* | G-patch domain containing 8 | 7.34 | 7.45 | -1.08 | 0.0152 |
| TC01003193.hg.1 | NM_003528; ENST00000369155; BC005827; BC069193; BC096121; BC098112; BC107084; OTTHUMT00000033455; uc001etc.3 | *HIST2H2BE* | histone cluster 2, H2be | 8.25 | 8.04 | 1.16 | 0.0152 |
| TC12000114.hg.1 | NM_024865; ENST00000229307; ENST00000526286; uc009zfy.1 | *NANOG* | Nanog homeobox | 4.18 | 4.33 | -1.11 | 0.0152 |
| TC17001715.hg.1 | NR_027025; uc010wnj.1 | *MTVR2* | mouse mammary tumor virus receptor homolog 2 | 5.37 | 5.14 | 1.18 | 0.0153 |
| TC09001366.hg.1 | NM_000264; NM_001083602; NM_001083603; NM_001083604; NM_001083605; NM_001083606; NM_001083607; ENST00000331920; ENST00000375274; ENST00000418258; ENST00000421141; ENST00000429896; ENST00000430669; ENST00000437951; ENST00000468211; ENST00000548379; AB212827; AB233422; AB233423; AB233424; AB239329; AK124593; BC043542; OTTHUMT00000053229; OTTHUMT00000053230; OTTHUMT00000053231; OTTHUMT00000053232; OTTHUMT00000053233; uc004avk.4; uc004avl.4; uc004avm.4; uc004avo.2; uc010mrn.3; uc010mro.3; uc010mrp.3; uc010mrq.3; uc010mrr.3; uc010mrs.1; uc010mrt.1; uc010mru.1; uc010mrv.1; uc010mrw.1 | *PTCH1* | patched 1 | 5.63 | 5.84 | -1.16 | 0.0153 |
| TC6_mann_hap4000024.hg.1 | NM_014596; NM_170783; BC010898; BC050608; OTTHUMT00000311165; OTTHUMT00000311166; OTTHUMT00000311167; OTTHUMT00000311168; OTTHUMT00000311169; OTTHUMT00000311170; uc011grj.2; uc011grk.2 | *ZNRD1* | zinc ribbon domain containing 1 | 4.67 | 4.58 | 1.06 | 0.0153 |
| TC03000676.hg.1 | NR_026954; uc011bkp.2 | *LOC90246* | uncharacterized LOC90246 | 3.58 | 3.4 | 1.13 | 0.0154 |
| TC07001296.hg.1 | AK096766 | *TARP* | TCR gamma alternate reading frame protein | 4.93 | 5.9 | -1.96 | 0.0155 |
| TC06003033.hg.1 | AK023584 | *RNF217* | ring finger protein 217 | 2.1 | 1.88 | 1.17 | 0.0156 |
| TC16000435.hg.1 | NM_153261; ENST00000389134; ENST00000427478; ENST00000458059; BC022550; BC036683; FJ516381; uc002eft.3; uc002efu.3; uc002efv.3 | *CNEP1R1* | CTD nuclear envelope phosphatase 1 regulatory subunit 1 | 6.19 | 5.98 | 1.15 | 0.0158 |
| TC19001180.hg.1 | AF161365 | *HSPC102* | uncharacterized LOC105372274 | 8.29 | 7.99 | 1.23 | 0.0158 |
| TC09002423.hg.1 | uc003zou.1 | *IFNA22P* | interferon, alpha 22, pseudogene | 1.37 | 1.54 | -1.12 | 0.0159 |
| TC20001388.hg.1 | BC041916 | *C20orf194* | chromosome 20 open reading frame 194 | 3.8 | 3.95 | -1.11 | 0.016 |
| TC01003506.hg.1 | NR_036064; uc021per.1 | *MIR3119-1* | microRNA 3119-1 | 1.08 | 0.97 | 1.08 | 0.016 |
| TC12000160.hg.1 | NM_001114396; NM_002262; NM_007334; ENST00000336164; ENST00000350274; ENST00000381908; ENST00000538997; ENST00000543420; ENST00000543777; ENST00000544747; AF498040; AJ000001; AY327501; BC028009; uc001qxw.4; uc001qxx.4; uc001qxy.4; uc001qxz.4; uc009zhh.3; uc009zhi.3 | *KLRD1* | killer cell lectin-like receptor subfamily D, member 1 | 6.1 | 6.55 | -1.36 | 0.0161 |
| TC0X002094.hg.1 | AL080202 | *LINC01278* | long intergenic non-protein coding RNA 1278 | 5.13 | 5.29 | -1.12 | 0.0162 |
| TC03001258.hg.1 | ENST00000432163; BC037254 | *LOC100996624* | uncharacterized LOC100996624 | 2.66 | 2.51 | 1.11 | 0.0162 |
| TC08001630.hg.1 | FM177980 | *POU5F1B* | POU class 5 homeobox 1B | 5.23 | 5.09 | 1.1 | 0.0162 |
| TC13000584.hg.1 | NM_025138; NM_170719; ENST00000352251; ENST00000484434; BC064348; OTTHUMT00000044605; OTTHUMT00000044606; OTTHUMT00000044607; OTTHUMT00000044608; OTTHUMT00000044609; OTTHUMT00000044610; OTTHUMT00000044611 | *PROSER1* | proline and serine rich 1 | 5.96 | 6.06 | -1.07 | 0.0162 |
| TC04001431.hg.1 | NM_003340; NM_181886; NM_181887; NM_181888; NM_181889; NM_181890; NM_181891; NM_181892; NM_181893; ENST00000321805; ENST00000338145; ENST00000343106; ENST00000349311; ENST00000350435; ENST00000357194; ENST00000394801; ENST00000394803; ENST00000394804; ENST00000453744; ENST00000502404; ENST00000504211; ENST00000505207; ENST00000507845; ENST00000513098; BC003395; BC037894; BC066917; OTTHUMT00000253791; OTTHUMT00000253792; OTTHUMT00000253793; OTTHUMT00000253794; OTTHUMT00000253795; OTTHUMT00000253796; OTTHUMT00000363392; OTTHUMT00000363393; OTTHUMT00000363394; OTTHUMT00000363395; OTTHUMT00000363396; OTTHUMT00000363397; OTTHUMT00000363398; OTTHUMT00000363399; OTTHUMT00000363400; OTTHUMT00000363401; OTTHUMT00000363402; OTTHUMT00000363403; OTTHUMT00000363404; OTTHUMT00000363405; OTTHUMT00000363406; OTTHUMT00000363407; OTTHUMT00000363408; OTTHUMT00000363409; OTTHUMT00000363410; OTTHUMT00000363555; OTTHUMT00000363556; OTTHUMT00000363557; OTTHUMT00000363558; OTTHUMT00000363559; OTTHUMT00000363560; uc003hwi.3; uc003hwk.3; uc003hwl.3; uc003hwo.3; uc003hwp.3; uc003hwq.3; uc003hwr.3; uc011cet.2; uc011ceu.2 | *UBE2D3* | ubiquitin conjugating enzyme E2D 3 | 7.32 | 7.19 | 1.1 | 0.0162 |
| TC07001889.hg.1 | NM_205855; ENST00000338588; ENST00000415751; AK297754; BC137311; BC137312; OTTHUMT00000340554; OTTHUMT00000340555; OTTHUMT00000340556; uc003vtd.3; uc010lmt.3; uc010lmu.2 | *FAM180A* | family with sequence similarity 180, member A | 2.6 | 2.67 | -1.05 | 0.0163 |
| TC6_mann_hap4000119.hg.1 | NM_014641; AK302434; BC110645; BC152556; EF177821; EF177822; OTTHUMT00000311028; OTTHUMT00000311029; OTTHUMT00000311030; OTTHUMT00000311031; OTTHUMT00000311032; OTTHUMT00000311033; OTTHUMT00000311034; OTTHUMT00000311035; uc011guc.2; uc011gud.2; uc011gue.1; uc011guf.1; uc011gug.1 | *MDC1* | mediator of DNA-damage checkpoint 1 | 5.45 | 5.56 | -1.08 | 0.0163 |
| TC6_cox_hap2000039.hg.1 | NM_001025091; NM_001090; AK307551; BC034488; BC112923; OTTHUMT00000076763; OTTHUMT00000076764; OTTHUMT00000076765; OTTHUMT00000076766; OTTHUMT00000256507; OTTHUMT00000314657; OTTHUMT00000314658; uc011eyk.1; uc011eyl.2; uc011eym.2 | *ABCF1* | ATP binding cassette subfamily F member 1 | 6.4 | 6.51 | -1.08 | 0.0164 |
| TC6_dbb_hap3000032.hg.1 | NM_001025091; NM_001090; AK307551; BC034488; BC112923; OTTHUMT00000310219; OTTHUMT00000310220; OTTHUMT00000310221; OTTHUMT00000310222; OTTHUMT00000310223; OTTHUMT00000310224; OTTHUMT00000310225; uc011fwx.1; uc011fwy.2; uc011fwz.2 | *ABCF1* | ATP binding cassette subfamily F member 1 | 6.4 | 6.51 | -1.08 | 0.0164 |
| TC6_mann_hap4000034.hg.1 | NM_001025091; NM_001090; AK307551; BC034488; BC112923; OTTHUMT00000311350; OTTHUMT00000311351; OTTHUMT00000311352; OTTHUMT00000311353; OTTHUMT00000311354; OTTHUMT00000311355; OTTHUMT00000311356; uc011gta.1; uc011gtb.2; uc011gtc.2 | *ABCF1* | ATP binding cassette subfamily F member 1 | 6.4 | 6.51 | -1.08 | 0.0164 |
| TC6_mcf_hap5000026.hg.1 | NM_001025091; NM_001090; AK307551; BC034488; BC112923; OTTHUMT00000312260; OTTHUMT00000312261; OTTHUMT00000312262; OTTHUMT00000312263; OTTHUMT00000312264; OTTHUMT00000312265; OTTHUMT00000312266; uc011hlu.1; uc011hlv.2; uc011hlw.2 | *ABCF1* | ATP binding cassette subfamily F member 1 | 6.4 | 6.51 | -1.08 | 0.0164 |
| TC6_qbl_hap6000032.hg.1 | NM_001025091; NM_001090; AK307551; BC034488; BC112923; OTTHUMT00000035475; OTTHUMT00000035476; OTTHUMT00000035477; OTTHUMT00000035478; OTTHUMT00000256160; OTTHUMT00000256161; OTTHUMT00000256162; uc011iiz.1; uc011ija.2; uc011ijb.2 | *ABCF1* | ATP binding cassette subfamily F member 1 | 6.4 | 6.51 | -1.08 | 0.0164 |
| TC6_ssto_hap7000032.hg.1 | NM_001025091; NM_001090; AK307551; BC034488; BC112923; OTTHUMT00000256625; OTTHUMT00000256626; OTTHUMT00000256627; OTTHUMT00000256628; OTTHUMT00000256629; OTTHUMT00000315282; OTTHUMT00000315283; uc011jgd.1; uc011jge.2; uc011jgf.2 | *ABCF1* | ATP binding cassette subfamily F member 1 | 6.4 | 6.51 | -1.08 | 0.0164 |
| TC14001303.hg.1 | NM_005589; ENST00000350259; ENST00000553458; ENST00000555126; ENST00000556852; AK294243; AK311478; BC004909; BC032371; uc001xpo.3; uc010asa.3; uc010tuq.2 | *ALDH6A1* | aldehyde dehydrogenase 6 family, member A1 | 4.54 | 4.64 | -1.07 | 0.0165 |
| TC13000372.hg.1 | AK096424; uc001vpx.1 | *METTL21EP* | methyltransferase like 21E, pseudogene | 3.4 | 3.26 | 1.1 | 0.0165 |
| TC18000266.hg.1 | NM_001136180; ENST00000451882; BC157848; uc002lno.4 | *HSBP1L1* | heat shock factor binding protein 1-like 1 | 5.2 | 5.08 | 1.09 | 0.0166 |
| TC02002557.hg.1 | NR_031648; uc021vss.1 | *MIR1246* | microRNA 1246 | 2.45 | 2.31 | 1.1 | 0.0166 |
| TC10001699.hg.1 | NM_003750; NR_002917; ENST00000369144; ENST00000384737; ENST00000462527; ENST00000541549; AK302575; AK309542; BC114429; OTTHUMT00000050634; OTTHUMT00000050635; uc001ldu.3; uc001ldv.3; uc009xzg.1; uc010qsu.2 | *EIF3A; SNORA19* | eukaryotic translation initiation factor 3, subunit A; small nucleolar RNA, H/ACA box 19 | 7.63 | 7.69 | -1.04 | 0.0167 |
| TC04001393.hg.1 | NM_014485; ENST00000295256; ENST00000514774; BC020734; OTTHUMT00000253587; OTTHUMT00000362982; OTTHUMT00000362983; uc003hte.1 | *HPGDS* | hematopoietic prostaglandin D synthase | 2.41 | 2.23 | 1.13 | 0.0168 |
| TC01000922.hg.1 | NR_033990; ENST00000418362; uc021oqw.1 | *LOC100129138* | THAP domain containing, apoptosis associated protein 3 pseudogene | 5.58 | 5.52 | 1.04 | 0.0168 |
| TC16000523.hg.1 | NM_020786; ENST00000311765; BC028030; OTTHUMT00000268831; uc002eqk.2 | *PDP2* | pyruvate dehyrogenase phosphatase catalytic subunit 2 | 3.95 | 4.02 | -1.05 | 0.0168 |
| TC11002223.hg.1 | NM_178127; ENST00000334289; BC049170; uc001pgl.3 | *ANGPTL5* | angiopoietin like 5 | 1.39 | 1.3 | 1.06 | 0.0169 |
| TC07003341.hg.1 | NM_001662; ENST00000000233; ENST00000467281; AK307297; BC003043; BC033104; OTTHUMT00000059567; OTTHUMT00000141971; OTTHUMT00000141972; OTTHUMT00000141973; OTTHUMT00000308979; OTTHUMT00000317141; uc003vmb.2; uc010llb.2 | *ARF5* | ADP-ribosylation factor 5 | 6.2 | 6.16 | 1.03 | 0.0169 |
| TC08001164.hg.1 | NM_001135731; NM_024645; ENST00000297737; ENST00000315769; ENST00000523823; BC019598; OTTHUMT00000376949; OTTHUMT00000376950; OTTHUMT00000376951; OTTHUMT00000376952; OTTHUMT00000376953; OTTHUMT00000376954; OTTHUMT00000376955; OTTHUMT00000376956; OTTHUMT00000376957; uc003xnr.3; uc003xns.3 | *ZMAT4* | zinc finger, matrin-type 4 | 2.48 | 2.57 | -1.07 | 0.0169 |
| TC02000463.hg.1 | NM_013247; NM_145074; ENST00000258080; ENST00000352222; ENST00000467961; AF141306; AK225932; AK310163; BC000096; OTTHUMT00000252219; OTTHUMT00000252220; OTTHUMT00000328589; OTTHUMT00000328590; OTTHUMT00000328591; OTTHUMT00000328592; OTTHUMT00000328593; OTTHUMT00000328594; OTTHUMT00000328595; OTTHUMT00000328596; uc002smi.1; uc002smj.1; uc002smk.1; uc002sml.1; uc010ffl.3 | *HTRA2* | HtrA serine peptidase 2 | 5.56 | 5.5 | 1.04 | 0.017 |
| TC11003397.hg.1 | BC012364 | *LOC105369553* | uncharacterized LOC105369553 | 3.53 | 3.75 | -1.17 | 0.017 |
| TC04001281.hg.1 | NM_002619; ENST00000296029; BC093965; BC112093; OTTHUMT00000252282 | *PF4* | platelet factor 4 | 8.2 | 7.82 | 1.3 | 0.017 |
| TC16000053.hg.1 | NM_174903; ENST00000321392; BC113014; uc002cnt.1 | *RNF151* | ring finger protein 151 | 4.74 | 4.85 | -1.08 | 0.0171 |
| TC03000816.hg.1 | NM_016275; ENST00000471696; ENST00000477889; ENST00000480740; ENST00000485923; BC006012; BC008411; BC009556; BC009611; BC026350; BC036738; BC071699; uc021xfp.1 | *SELT* | selenoprotein T | 6.39 | 6.24 | 1.11 | 0.0171 |
| TC06000330.hg.1 | NM_014596; NM_170783; ENST00000332435; ENST00000359374; ENST00000376782; ENST00000376785; ENST00000463141; BC010898; BC050608; OTTHUMT00000076271; OTTHUMT00000076272; OTTHUMT00000076273; OTTHUMT00000076274; OTTHUMT00000253192; OTTHUMT00000253193; uc003noz.3; uc003npa.3 | *ZNRD1* | zinc ribbon domain containing 1 | 4.63 | 4.56 | 1.05 | 0.0171 |
| TC0X000768.hg.1 | NM_000117; ENST00000369835; ENST00000369842; ENST00000492448; BC000738; OTTHUMT00000080921; OTTHUMT00000080923; OTTHUMT00000080924; OTTHUMT00000080925; OTTHUMT00000081633; OTTHUMT00000127770; OTTHUMT00000127771; OTTHUMT00000127772; OTTHUMT00000130306; uc004fkl.3 | *EMD* | emerin | 6.04 | 6.13 | -1.06 | 0.0172 |
| TC04002928.hg.1 | NM_001145191; ENST00000422728; ENST00000504137; BC017971; OTTHUMT00000360030; OTTHUMT00000360031; OTTHUMT00000360099; OTTHUMT00000360100; OTTHUMT00000360101; OTTHUMT00000360102; OTTHUMT00000360103; OTTHUMT00000360104; OTTHUMT00000360105; OTTHUMT00000360106; OTTHUMT00000360107; OTTHUMT00000360108; OTTHUMT00000360109; OTTHUMT00000360110; OTTHUMT00000360111; OTTHUMT00000360112; OTTHUMT00000360113; OTTHUMT00000360114; OTTHUMT00000360115; OTTHUMT00000360116; OTTHUMT00000360117; uc003gof.4 | *FAM200B* | family with sequence similarity 200, member B | 4.82 | 4.74 | 1.06 | 0.0172 |
| TC02001686.hg.1 | NR_002201; uc002rkn.1 | *FTH1P3* | ferritin, heavy polypeptide 1 pseudogene 3 | 7.97 | 7.82 | 1.11 | 0.0172 |
| TC0X000767.hg.1 | NM_001145933; NM_001145934; NM_012253; ENST00000369912; ENST00000369915; ENST00000482044; AK302261; BC025382; OTTHUMT00000058922; OTTHUMT00000058923; OTTHUMT00000058924; OTTHUMT00000132714; OTTHUMT00000286451; OTTHUMT00000286452; OTTHUMT00000286453; OTTHUMT00000316554; uc004fkg.3; uc004fkh.3; uc011mzl.2; uc011mzm.2 | *TKTL1* | transketolase-like 1 | 4.37 | 4.74 | -1.29 | 0.0172 |
| TC01004298.hg.1 | BC103825 | *TXLNA* | taxilin alpha | 6.04 | 6.2 | -1.12 | 0.0172 |
| TC13001642.hg.1 | AF339791 | *DZIP1* | DAZ interacting zinc finger protein 1 | 1.24 | 1.09 | 1.11 | 0.0173 |
| TC06001507.hg.1 | NM_014641; ENST00000376406; ENST00000494654; AK302434; BC110645; BC152556; EF177821; EF177822; OTTHUMT00000076103; OTTHUMT00000257581; OTTHUMT00000257582; OTTHUMT00000257583; OTTHUMT00000257584; OTTHUMT00000257585; OTTHUMT00000257586; OTTHUMT00000257587; uc003nrf.4; uc003nrg.4; uc003nrh.1; uc003nri.2; uc011dmp.1 | *MDC1* | mediator of DNA-damage checkpoint 1 | 5.41 | 5.53 | -1.08 | 0.0173 |
| TC12000130.hg.1 | NM_080387; ENST00000299665; BC032313; uc001qun.3 | *CLEC4D* | C-type lectin domain family 4, member D | 5.35 | 4.77 | 1.5 | 0.0174 |
| TC10000483.hg.1 | NM_012330; ENST00000287239; ENST00000372711; ENST00000372714; ENST00000372724; ENST00000372725; ENST00000490365; AF113514; AF119230; AK074816; BC150270; BC150618; OTTHUMT00000048769; OTTHUMT00000048770; OTTHUMT00000048771; OTTHUMT00000048772; OTTHUMT00000048773; OTTHUMT00000048774 | *KAT6B* | K(lysine) acetyltransferase 6B | 6.14 | 6.25 | -1.08 | 0.0174 |
| TC09001497.hg.1 | NR_024376; ENST00000439875 | *FAM225B* | family with sequence similarity 225, member B (non-protein coding) | 4.66 | 4.74 | -1.06 | 0.0175 |
| TC09000898.hg.1 | ENST00000355513 | *C9orf38* | chromosome 9 open reading frame 38 | 2.07 | 2.2 | -1.09 | 0.0176 |
| TC02000669.hg.1 | NM_144978; ENST00000295124; ENST00000412964; ENST00000470608; AK125199; BC038421; BC050579; OTTHUMT00000253593; OTTHUMT00000331203; OTTHUMT00000331204; OTTHUMT00000331205; OTTHUMT00000331206; OTTHUMT00000331207; uc002ten.1; uc002teo.1; uc002tep.1; uc010fjm.1 | *CCDC138* | coiled-coil domain containing 138 | 2.42 | 2.35 | 1.05 | 0.0176 |
| TC04001974.hg.1 | AK131492 | *DTHD1* | death domain containing 1 | 4.99 | 5.32 | -1.25 | 0.0176 |
| TC01005924.hg.1 | AF085892 | *LOC101928565* | uncharacterized LOC101928565 | 2.81 | 2.73 | 1.05 | 0.0176 |
| TC02002611.hg.1 | NR_039947; uc021vtw.1 | *MIR1245B* | microRNA 1245b | 0.98 | 0.91 | 1.05 | 0.0176 |
| TC06001669.hg.1 | NM_001193476; NM_052961; NM_138718; ENST00000355574; ENST00000394602; ENST00000490799; AK055314; AK057276; BC025408; OTTHUMT00000040325; OTTHUMT00000040326; OTTHUMT00000040327; OTTHUMT00000356007; OTTHUMT00000356008; OTTHUMT00000356009; OTTHUMT00000356010; OTTHUMT00000356011; uc003olk.3; uc003oll.3; uc003olm.3; uc003oln.3; uc010jwa.3 | *SLC26A8* | solute carrier family 26 (anion exchanger), member 8 | 4.64 | 4.37 | 1.2 | 0.0178 |
| TC22000566.hg.1 | NM_021916; ENST00000341976; BC040161; OTTHUMT00000319881; uc002zxs.3 | *ZNF70* | zinc finger protein 70 | 5.2 | 5.3 | -1.08 | 0.0178 |
| TC11002247.hg.1 | NM_001136109; NM_001136110; NM_001136112; NM_004347; NR_024239; NR_036562; ENST00000260315; ENST00000393141; ENST00000418434; ENST00000444749; ENST00000526056; ENST00000531367; BC074994; BC113406; OTTHUMT00000109397; OTTHUMT00000344191; OTTHUMT00000344192; OTTHUMT00000344193; OTTHUMT00000344537; uc009yxh.2; uc010ruz.1; uc010rva.1; uc010rvb.1; uc010rvc.1; uc010rvd.1 | *CASP5* | caspase 5 | 4.87 | 4.43 | 1.35 | 0.0179 |
| TC01002616.hg.1 | NM_001114172; NM_003629; ENST00000262741; ENST00000372006; ENST00000420542; ENST00000423209; ENST00000488808; ENST00000540385; AF028785; AK302049; AK304713; BC021622; OTTHUMT00000022166; OTTHUMT00000022167; OTTHUMT00000022168; OTTHUMT00000022169; OTTHUMT00000022170; OTTHUMT00000022171; uc001cpb.4; uc001cpc.4; uc009vyb.3; uc009vyc.3; uc010olv.2; uc010olw.2 | *PIK3R3* | phosphoinositide-3-kinase, regulatory subunit 3 (gamma) | 3.71 | 3.77 | -1.04 | 0.0179 |
| TC03001289.hg.1 | ENST00000449586 | *ITGA9-AS1* | ITGA9 antisense RNA 1 | 2.77 | 2.64 | 1.1 | 0.018 |
| TC6_mcf_hap5000120.hg.1 | NM_014641; AK302434; BC110645; BC152556; EF177821; EF177822; OTTHUMT00000311927; OTTHUMT00000311928; OTTHUMT00000311929; OTTHUMT00000311930; OTTHUMT00000311931; OTTHUMT00000311932; OTTHUMT00000311933; OTTHUMT00000311934; uc011hmw.2; uc011hmx.2; uc011hmy.1; uc011hmz.1; uc011hna.1 | *MDC1* | mediator of DNA-damage checkpoint 1 | 5.5 | 5.6 | -1.08 | 0.018 |
| TC6_ssto_hap7000121.hg.1 | NM_014641; AK302434; BC110645; BC152556; EF177821; EF177822; OTTHUMT00000258031; OTTHUMT00000315298; OTTHUMT00000315299; OTTHUMT00000315300; OTTHUMT00000315301; OTTHUMT00000315302; OTTHUMT00000315303; OTTHUMT00000315304; uc011jhb.2; uc011jhc.2; uc011jhd.1; uc011jhe.1; uc011jhf.1 | *MDC1* | mediator of DNA-damage checkpoint 1 | 5.5 | 5.6 | -1.08 | 0.018 |
| TC6_qbl_hap6000131.hg.1 | NM_014641; AK302434; BC110645; BC152556; EF177821; EF177822; OTTHUMT00000018852; OTTHUMT00000018853; OTTHUMT00000018854; OTTHUMT00000018855; OTTHUMT00000018856; OTTHUMT00000018857; OTTHUMT00000035378; uc011ikb.2; uc011ikc.2; uc011ikd.1; uc011ike.1; uc011ikf.1 | *MDC1* | mediator of DNA-damage checkpoint 1 | 5.54 | 5.64 | -1.08 | 0.018 |
| TC02000197.hg.1 | NM_001127399; NM_001127400; NM_001127401; NM_016061; ENST00000261353; ENST00000379519; ENST00000379520; ENST00000402003; ENST00000402708; ENST00000495673; AK307099; BC000836; BC047237; OTTHUMT00000215127; OTTHUMT00000215128; OTTHUMT00000215129; OTTHUMT00000325352; OTTHUMT00000325354; OTTHUMT00000325356; OTTHUMT00000325357; OTTHUMT00000325358; OTTHUMT00000325359; OTTHUMT00000325391; uc002rmz.4; uc002rna.4; uc002rnb.4; uc002rnc.4; uc002rnd.3; uc010ezn.3 | *YPEL5* | yippee like 5 | 7.16 | 7.05 | 1.08 | 0.0181 |
| TC07001260.hg.1 | NR_037514; uc022abl.1 | *MIR550B2* | microRNA 550b-2 | 2.58 | 2.74 | -1.11 | 0.0182 |
| TC16000840.hg.1 | NM_016256; ENST00000312251; ENST00000381955; AF052111; AK302916; BC012194; OTTHUMT00000207003; uc002cyg.3; uc002cyh.3; uc010buc.3; uc010uxx.2 | *NAGPA* | N-acetylglucosamine-1-phosphodiester alpha-N-acetylglucosaminidase | 5.76 | 5.84 | -1.05 | 0.0182 |
| TC07001209.hg.1 | NM_022150; ENST00000222674; OTTHUMT00000250315; uc003sxo.3 | *NPVF* | neuropeptide VF precursor | 1.32 | 1.24 | 1.06 | 0.0182 |
| TC03003353.hg.1 | NM_015106; ENST00000409535; AK023824; AK122683; AK304881; BC024298; OTTHUMT00000328672; OTTHUMT00000328700; OTTHUMT00000328701; OTTHUMT00000328702; OTTHUMT00000346540; uc003dbh.3; uc003dbj.3; uc011bdt.2; uc011bdu.2 | *RAD54L2* | RAD54-like 2 (S. cerevisiae) | 5.58 | 5.69 | -1.07 | 0.0182 |
| TC02001989.hg.1 | ENST00000413452; ENST00000439192; ENST00000453103 | *DGUOK-AS1* | DGUOK antisense RNA 1 | 2.49 | 2.38 | 1.08 | 0.0183 |
| TC06003250.hg.1 | AK097546 | *LOC285766* | uncharacterized LOC285766 | 2.85 | 2.79 | 1.04 | 0.0183 |
| TC21000226.hg.1 | NM_030891; ENST00000291592; BC119648; BC119649; OTTHUMT00000098095; uc002zfa.3 | *LRRC3* | leucine rich repeat containing 3 | 4.96 | 5.08 | -1.08 | 0.0183 |
| TC6_cox_hap2000141.hg.1 | NM_014641; AK302434; BC110645; BC152556; EF177821; EF177822; OTTHUMT00000076730; OTTHUMT00000314762; OTTHUMT00000314763; OTTHUMT00000314764; OTTHUMT00000314765; OTTHUMT00000314766; OTTHUMT00000314767; OTTHUMT00000314768; uc011ezm.2; uc011ezn.2; uc011ezo.1; uc011ezp.1; uc011ezq.1 | *MDC1* | mediator of DNA-damage checkpoint 1 | 5.42 | 5.53 | -1.08 | 0.0183 |
| TC12000399.hg.1 | NM_014033; ENST00000332160; ENST00000548553; AK293356; BC004492; BC008180; uc001rxb.3; uc010smv.1 | *METTL7A* | methyltransferase like 7A | 6.24 | 6.37 | -1.09 | 0.0183 |
| TC16001045.hg.1 | NR_039744; uc021tgr.1 | *MIR4519* | microRNA 4519 | 5.29 | 5.44 | -1.11 | 0.0183 |
| TC6_mcf_hap5000018.hg.1 | NM_014596; NM_170783; BC010898; BC050608; OTTHUMT00000312053; OTTHUMT00000312054; OTTHUMT00000312055; OTTHUMT00000312056; OTTHUMT00000312057; OTTHUMT00000312058; uc011hkp.2; uc011hkq.2 | *ZNRD1* | zinc ribbon domain containing 1 | 4.7 | 4.61 | 1.06 | 0.0183 |
| TC13000382.hg.1 | NM_032859; ENST00000375898; BC022566; BC070226; OTTHUMT00000045743; uc001vqq.3 | *ABHD13* | abhydrolase domain containing 13 | 6.79 | 6.7 | 1.06 | 0.0184 |
| TC10000044.hg.1 | NM_001818; ENST00000263126; ENST00000380448; ENST00000469875; BC020744; OTTHUMT00000046543; OTTHUMT00000046544; uc001ihw.2 | *AKR1C4* | aldo-keto reductase family 1, member C4 | 2.1 | 2.2 | -1.07 | 0.0184 |
| TC14001935.hg.1 | BX647964; NR_023921; NR_023922; NR_023923 | *DHRS4-AS1* | DHRS4 antisense RNA 1 | 5.37 | 5.44 | -1.04 | 0.0184 |
| TC6_apd_hap1000077.hg.1 | NM_014641; AK302434; BC110645; BC152556; EF177821; EF177822; OTTHUMT00000309234; OTTHUMT00000309235; OTTHUMT00000309236; OTTHUMT00000309237; OTTHUMT00000309238; OTTHUMT00000309239; OTTHUMT00000309240; OTTHUMT00000309241; uc011elr.2; uc011els.2; uc011elt.1; uc011elu.1; uc011elv.1 | *MDC1* | mediator of DNA-damage checkpoint 1 | 5.49 | 5.6 | -1.08 | 0.0184 |
| TC6_dbb_hap3000130.hg.1 | NM_014641; AK302434; BC110645; BC152556; EF177821; EF177822; OTTHUMT00000309891; OTTHUMT00000309892; OTTHUMT00000309893; OTTHUMT00000309894; OTTHUMT00000309895; OTTHUMT00000309896; OTTHUMT00000309897; OTTHUMT00000309898; uc011fxz.2; uc011fya.2; uc011fyb.1; uc011fyc.1; uc011fyd.1 | *MDC1* | mediator of DNA-damage checkpoint 1 | 5.49 | 5.6 | -1.08 | 0.0184 |
| TC07002775.hg.1 | BC040728 | *RNF216* | ring finger protein 216 | 5.72 | 5.5 | 1.17 | 0.0184 |
| TC09000441.hg.1 | NM_145006; ENST00000375469; ENST00000375472; ENST00000471462; AY358190; BC014601; BC041834; OTTHUMT00000053120; OTTHUMT00000053121; OTTHUMT00000053122; uc004atb.3; uc004atc.3 | *SUSD3* | sushi domain containing 3 | 6.32 | 6.25 | 1.05 | 0.0184 |
| TC17000892.hg.1 | NM_001142640; NM_018996; ENST00000301624; ENST00000335749; AK098609; BC039479; OTTHUMT00000255891; uc002juc.2; uc002jud.2; uc002jue.2; uc002juf.2 | *TNRC6C* | trinucleotide repeat containing 6C | 6.75 | 6.82 | -1.05 | 0.0184 |
| TC17000444.hg.1 | NM_012138; BC000591; uc002hni.3 | *AATF* | apoptosis antagonizing transcription factor | 7.63 | 7.79 | -1.12 | 0.0185 |
| TC03003023.hg.1 | BC015772 | *LOC100288721* | uncharacterized LOC100288721 | 1.74 | 1.95 | -1.15 | 0.0185 |
| TC01001013.hg.1 | ENST00000438885; BC043254 | *LOC101928995* | uncharacterized LOC101928995 | 1.66 | 1.78 | -1.08 | 0.0185 |
| TC06002280.hg.1 | NR_028092; NR_028093; ENST00000335388; uc003qtj.2; uc011efy.2 | *LPAL2* | lipoprotein, Lp(a)-like 2, pseudogene | 4.75 | 4.93 | -1.14 | 0.0186 |
| TC22001256.hg.1 | NR_038911 | *MIF-AS1* | MIF antisense RNA 1 | 4.33 | 4.58 | -1.19 | 0.0186 |
| TC03001429.hg.1 | NM_001206957; NM_007182; NM_170712; NM_170713; NM_170714; ENST00000327761; ENST00000357043; ENST00000359365; ENST00000395126; ENST00000488024; AF102772; AF291719; AK300994; BC110412; BC117153; OTTHUMT00000314303; OTTHUMT00000314304; OTTHUMT00000314305; OTTHUMT00000314306; OTTHUMT00000314307; OTTHUMT00000314309; OTTHUMT00000314311; OTTHUMT00000346387; OTTHUMT00000346388; uc003daa.1; uc003dab.1; uc003dac.2; uc003dad.1; uc003dae.1; uc003daf.1; uc010hlk.1; uc011bdq.1 | *RASSF1* | Ras association (RalGDS/AF-6) domain family member 1 | 5.83 | 5.91 | -1.06 | 0.0186 |
| TC19000442.hg.1 | NM_000175; NM_001184722; ENST00000356487; ENST00000415930; AB209575; AK025421; AK129884; AK293446; BC004982; uc002nvf.3; uc002nvg.2; uc002nvh.1; uc002nvi.2; uc010xrv.2; uc010xrw.2 | *GPI* | glucose-6-phosphate isomerase | 6.74 | 6.93 | -1.14 | 0.0187 |
| TC21001063.hg.1 | NM_001001503; NM_021075; ENST00000340344; ENST00000354250; ENST00000460259; BC021217; OTTHUMT00000195446; OTTHUMT00000195447; OTTHUMT00000195448; OTTHUMT00000195449; uc002zcm.3; uc002zcn.3 | *NDUFV3* | NADH dehydrogenase (ubiquinone) flavoprotein 3, 10kDa | 6.11 | 6.19 | -1.06 | 0.0187 |
| TC17001092.hg.1 | NM_004860; ENST00000250113; BC020090; BC051907; BC067272; uc002gia.2 | *FXR2* | fragile X mental retardation, autosomal homolog 2 | 5.83 | 5.86 | -1.02 | 0.0188 |
| TC13001580.hg.1 | BC034981 | *LINC00561* | long intergenic non-protein coding RNA 561 | 1.82 | 1.62 | 1.15 | 0.0188 |
| TC09001039.hg.1 | NM_148178; NM_148179; ENST00000297613; ENST00000378959; BC032136; OTTHUMT00000001129; OTTHUMT00000001130; uc003zuu.3; uc003zuv.3 | *RPP25L* | ribonuclease P/MRP 25kDa subunit-like | 5.76 | 5.9 | -1.1 | 0.019 |
| TC03000070.hg.1 | NM_001145392; NM_001145393; NM_001145394; NM_001145395; NM_025265; ENST00000284995; ENST00000402228; ENST00000415684; ENST00000444864; ENST00000454502; ENST00000475595; AK295508; BC004178; BC019582; OTTHUMT00000251981; OTTHUMT00000339517; OTTHUMT00000339518; OTTHUMT00000339519; OTTHUMT00000339520; OTTHUMT00000339521; OTTHUMT00000339522; OTTHUMT00000339523; OTTHUMT00000339524; OTTHUMT00000339525; uc003bwz.3; uc003bxa.3; uc003bxb.3; uc003bxc.3; uc011auq.1; uc011aur.1 | *TSEN2* | TSEN2 tRNA splicing endonuclease subunit | 4.42 | 4.34 | 1.06 | 0.0191 |
| TC12001445.hg.1 | NM_024095; ENST00000317697; ENST00000535055; ENST00000535988; ENST00000536071; ENST00000536549; ENST00000536953; ENST00000537754; ENST00000539528; ENST00000540782; AK297013; BC001321; uc001rrh.3; uc010slr.2 | *ASB8* | ankyrin repeat and SOCS box containing 8 | 7.09 | 7.23 | -1.1 | 0.0192 |
| TC01005427.hg.1 | BC080552 | *HIVEP3* | human immunodeficiency virus type I enhancer binding protein 3 | 5.08 | 5.24 | -1.12 | 0.0192 |
| TC01003709.hg.1 | NM_021633; ENST00000367259; ENST00000367261; AF306687; AF306689; AK024412; AK301791; BC003183; OTTHUMT00000099151; OTTHUMT00000099152; OTTHUMT00000099153; uc001gym.1; uc001gyn.1; uc001gyo.1; uc009xah.1; uc010pqc.1 | *KLHL12* | kelch-like family member 12 | 5.52 | 5.44 | 1.05 | 0.0192 |
| TC14002002.hg.1 | AK127576 | *LOC400212* | uncharacterized LOC400212 | 2.6 | 2.5 | 1.08 | 0.0192 |
| TC10002566.hg.1 | OTTHUMT00000047660 | *SLC9B1P3* | solute carrier family 9, subfamily B (NHA1, cation proton antiporter 1), member 1 pseudogene 3 | 2.01 | 1.95 | 1.04 | 0.0192 |
| TC20000538.hg.1 | NM_080571; NM_153269; ENST00000360321; ENST00000382369; ENST00000400269; AK302223; AK304526; BC134417; BC137450; BC144648; BC144649; OTTHUMT00000077439; OTTHUMT00000077440; uc002wde.2; uc010zpi.2; uc010zpj.1; uc010zpk.2; uc021vzl.1 | *C20orf96* | chromosome 20 open reading frame 96 | 3.51 | 3.64 | -1.09 | 0.0193 |
| TC21000592.hg.1 | NR_037585 | *LINC01549* | long intergenic non-protein coding RNA 1549 | 1.64 | 1.56 | 1.06 | 0.0193 |
| TC17002864.hg.1 | NM_003809; NR_037146; ENST00000293825; ENST00000462811; BC019047; BC071837; BC104420; OTTHUMT00000226951; OTTHUMT00000268803; OTTHUMT00000268804; OTTHUMT00000268805; uc002ghg.3; uc002ghh.3 | *TNFSF12* | tumor necrosis factor (ligand) superfamily, member 12 | 6.7 | 6.78 | -1.06 | 0.0193 |
| TC01001857.hg.1 | NM_002107; NM_005324; NR_002315; ENST00000366813; ENST00000366814; ENST00000366815; ENST00000366816; AK293541; BC029405; BC038989; BC081560; BC095447; M11353; OTTHUMT00000091323; OTTHUMT00000091324; OTTHUMT00000091325; OTTHUMT00000091326; uc001hpw.3; uc010pvl.2; uc021pjv.1 | *H3F3A; H3F3B; H3F3AP4* | H3 histone, family 3A; H3 histone, family 3B (H3.3B); H3 histone, family 3A, pseudogene 4 | 8.76 | 8.67 | 1.06 | 0.0195 |
| TC02004639.hg.1 | M31164 | *TNFAIP6* | tumor necrosis factor, alpha-induced protein 6 | 2.42 | 1.9 | 1.43 | 0.0195 |
| TC19001620.hg.1 | NM_001166049; NM_001983; NM_202001; ENST00000013807; ENST00000300853; ENST00000340192; ENST00000423698; BC008930; BC052813; uc002pbs.2; uc002pbt.2; uc002pbv.3 | *ERCC1* | excision repair cross-complementation group 1 | 5.8 | 5.87 | -1.05 | 0.0196 |
| TC10000240.hg.1 | NM_145012; NM_181698; ENST00000265375; ENST00000339497; ENST00000374704; ENST00000374706; ENST00000492478; AF465728; AK303283; BC050310; BC094815; BC104773; BC104801; BC143450; BC143455; OTTHUMT00000047567; OTTHUMT00000047568; OTTHUMT00000047569; OTTHUMT00000047570; OTTHUMT00000047571; OTTHUMT00000047572; OTTHUMT00000047573; OTTHUMT00000047574; uc001iyu.4; uc001iyv.4; uc001iyw.4; uc001iyx.4; uc009xmb.3; uc010qet.2 | *CCNY* | cyclin Y | 6.42 | 6.33 | 1.06 | 0.0197 |
| TC0X001697.hg.1 | AJ508600 | *TBC1D25* | TBC1 domain family, member 25 | 4.25 | 4.29 | -1.03 | 0.0198 |
| TC06001035.hg.1 | NM_016217; ENST00000367658; BC070068; OTTHUMT00000042456; uc003qin.3 | *HECA* | hdc homolog, cell cycle regulator | 7.81 | 7.69 | 1.09 | 0.0199 |
| TC01002044.hg.1 | NR_036070; uc021pmd.1 | *MIR3124* | microRNA 3124 | 2.44 | 2.33 | 1.08 | 0.0199 |
| TC02000322.hg.1 | NR_027258; ENST00000295112; uc010yoy.2 | *PRORSD1P* | prolyl-tRNA synthetase associated domain containing 1, pseudogene | 4.37 | 4.3 | 1.04 | 0.0199 |
| TC09000179.hg.1 | NM_001040410; NM_001040411; NM_001040412; NM_203299; ENST00000312292; ENST00000354479; ENST00000421362; BC045643; OTTHUMT00000052283; uc003zvu.3; uc003zvv.3; uc003zvw.3; uc003zvx.3 | *C9orf131* | chromosome 9 open reading frame 131 | 4.42 | 4.53 | -1.08 | 0.02 |
| TC05001910.hg.1 | NM_014790; ENST00000265272; ENST00000333010; ENST00000507386; AB011127; AK299724; BC017354; EF512550; OTTHUMT00000251941; OTTHUMT00000251942; OTTHUMT00000373321; OTTHUMT00000373322; OTTHUMT00000373323 | *JAKMIP2* | janus kinase and microtubule interacting protein 2 | 4.17 | 4.54 | -1.29 | 0.02 |
| TC11001902.hg.1 | NM_138689; ENST00000309318; ENST00000392210; ENST00000542235; BC014522; BC094817; uc001nza.3 | *PPP1R14B* | protein phosphatase 1, regulatory (inhibitor) subunit 14B | 6.04 | 5.99 | 1.04 | 0.02 |
| TC06001799.hg.1 | NM_002388; ENST00000229854; ENST00000419835; ENST00000476448; AK300754; BC001626; BC003509; OTTHUMT00000040897; OTTHUMT00000040899; OTTHUMT00000040900 | *MCM3* | minichromosome maintenance complex component 3 | 5.59 | 5.71 | -1.08 | 0.0201 |
| TC05001946.hg.1 | NM_001252385; NM_001252386; NM_001252390; NM_001252391; NM_001252392; NM_001252393; NM_006058; ENST00000315050; ENST00000389378; ENST00000518977; ENST00000520931; ENST00000521423; ENST00000521591; ENST00000522226; ENST00000523200; ENST00000523338; ENST00000524280; AB177543; AB177544; AB252974; AB252978; AB252979; AB252980; AB252981; BC012133; BC014008; OTTHUMT00000374909; OTTHUMT00000374910; OTTHUMT00000374911; OTTHUMT00000374912; OTTHUMT00000374913; OTTHUMT00000374914; OTTHUMT00000374915; OTTHUMT00000374916; OTTHUMT00000374917; OTTHUMT00000377088; OTTHUMT00000377603; OTTHUMT00000377604; OTTHUMT00000377605; OTTHUMT00000377606; OTTHUMT00000377607; OTTHUMT00000377608; OTTHUMT00000377609; OTTHUMT00000377610; uc003ltj.3; uc003ltk.3; uc010jhl.3; uc010jho.2; uc010jhp.2; uc010jhq.2; uc010jhs.2; uc021ygb.1 | *TNIP1* | TNFAIP3 interacting protein 1 | 7.31 | 7.21 | 1.07 | 0.0201 |
| TC04001265.hg.1 | NM_021139; ENST00000305107; ENST00000506580; ENST00000512583; AK300084; AY529122; BC026264; OTTHUMT00000365526; OTTHUMT00000365527; OTTHUMT00000365528; OTTHUMT00000365529; OTTHUMT00000365530; OTTHUMT00000365531; uc003hek.4; uc003hel.4; uc011cap.2 | *UGT2B4* | UDP glucuronosyltransferase 2 family, polypeptide B4 | 1.61 | 1.52 | 1.06 | 0.0202 |
| TC07001765.hg.1 | NM_152556; ENST00000297145; ENST00000485446; AK299901; BC114615; OTTHUMT00000338923; OTTHUMT00000338924; OTTHUMT00000338925; uc003vgo.1; uc011kms.1 | *C7orf60* | chromosome 7 open reading frame 60 | 5.25 | 5.19 | 1.04 | 0.0203 |
| TC17000515.hg.1 | NM_000805; ENST00000329402; BC069724; BC069762; OTTHUMT00000257409; uc002hxl.3 | *GAST* | gastrin | 6.64 | 6.76 | -1.09 | 0.0203 |
| TC22000907.hg.1 | NM_015166; NM_139202; ENST00000311597; ENST00000395876; ENST00000483836; AK294048; AK295106; AK297341; AK299841; BC028425; BC070042; OTTHUMT00000316979; OTTHUMT00000316980; OTTHUMT00000316981; OTTHUMT00000317005; OTTHUMT00000317006; uc003bjg.1; uc003bjh.1; uc011arl.1; uc011arm.1; uc011arn.1; uc011aro.1 | *MLC1* | megalencephalic leukoencephalopathy with subcortical cysts 1 | 5.14 | 5.26 | -1.08 | 0.0203 |
| TC17000822.hg.1 | NM_018714; ENST00000299886; ENST00000438720; AB037802; BC021985; BC047465; uc002jjf.1; uc002jjg.3; uc002jjh.3 | *COG1* | component of oligomeric golgi complex 1 | 6.58 | 6.67 | -1.07 | 0.0204 |
| TC01002529.hg.1 | NM_006802; ENST00000373019; ENST00000489537; AK301424; BC002395; BC011523; OTTHUMT00000012976; OTTHUMT00000012977; OTTHUMT00000012978; OTTHUMT00000012979; OTTHUMT00000012980; OTTHUMT00000012981; OTTHUMT00000012982; OTTHUMT00000012983; uc001cci.3; uc010oik.2 | *SF3A3* | splicing factor 3a subunit 3 | 5.6 | 5.76 | -1.12 | 0.0204 |
| TC05001914.hg.1 | ENST00000501695; AK054753 | *LOC102546294* | uncharacterized LOC102546294 | 3.52 | 3.38 | 1.1 | 0.0205 |
| TC14000940.hg.1 | NM_001039619; NM_006109; ENST00000216350; ENST00000324366; ENST00000397440; ENST00000397441; ENST00000553641; ENST00000553897; ENST00000556426; AK300863; AK301812; AK302240; BC005820; BC025979; OTTHUMT00000071674; OTTHUMT00000071676; OTTHUMT00000071678; OTTHUMT00000071679; uc001whl.1; uc001whm.1; uc001whn.1; uc010tnf.1; uc010tng.1; uc010tnh.1 | *PRMT5* | protein arginine methyltransferase 5 | 5.63 | 5.5 | 1.09 | 0.0207 |
| TC17000543.hg.1 | NM_173079; ENST00000361677; BC039247; uc002ici.1 | *RUNDC1* | RUN domain containing 1 | 6.05 | 5.94 | 1.08 | 0.0207 |
| TC18000142.hg.1 | NM_001143826; NM_001143827; NM_001256420; NM_014268; NR_046177; ENST00000300249; ENST00000413393; ENST00000436190; ENST00000538170; AB016823; AK296251; BC007318; OTTHUMT00000255753; uc002kyf.2 | *MAPRE2* | microtubule-associated protein, RP/EB family, member 2 | 6.95 | 7.07 | -1.08 | 0.0208 |
| TC10002264.hg.1 | AL137373 | *TAF5* | TAF5 RNA polymerase II, TATA box binding protein (TBP)-associated factor, 100kDa | 2.8 | 2.54 | 1.19 | 0.0208 |
| TC02000222.hg.1 | ENST00000366209; ENST00000442026 | *LINC01317* | long intergenic non-protein coding RNA 1317 | 2.42 | 2.35 | 1.05 | 0.0209 |
| TC19000257.hg.1 | NM_002741; NM_213560; ENST00000242783; ENST00000342216; BC040061; BC094766; OTTHUMT00000095510; OTTHUMT00000095511; uc002myp.3; uc002myq.3 | *PKN1* | protein kinase N1 | 6.59 | 6.65 | -1.04 | 0.0209 |
| TC08001408.hg.1 | NM_018710; ENST00000285419; BC033892; OTTHUMT00000376778; OTTHUMT00000376779; OTTHUMT00000376780; OTTHUMT00000376781; OTTHUMT00000376782; uc003yes.3 | *TMEM55A* | transmembrane protein 55A | 6.74 | 6.42 | 1.25 | 0.0209 |
| TC07002238.hg.1 | AY462278; AY495951; AY495952 | *ADCYAP1R1* | adenylate cyclase activating polypeptide 1 (pituitary) receptor type I | 4.4 | 4.48 | -1.06 | 0.021 |
| TC14000371.hg.1 | NM_006255; ENST00000332981; ENST00000555082; ENST00000556245; AK296158; AK300864; BC037268; OTTHUMT00000276974; uc001xfn.3; uc010tsa.2; uc010tsb.2 | *PRKCH* | protein kinase C, eta | 7.65 | 7.85 | -1.14 | 0.021 |
| TC03001941.hg.1 | NM_001167911; NM_001167912; NM_001167915; NM_001167916; NM_001167917; NM_024621; ENST00000362010; ENST00000392832; ENST00000392833; ENST00000468233; ENST00000469007; ENST00000494677; ENST00000537559; BC101660; BC111017; BC113555; OTTHUMT00000351845; OTTHUMT00000351846; OTTHUMT00000351847; OTTHUMT00000351848; OTTHUMT00000351849; OTTHUMT00000351850; OTTHUMT00000351851; OTTHUMT00000351852; OTTHUMT00000351853; OTTHUMT00000351854; OTTHUMT00000351855; OTTHUMT00000352009; OTTHUMT00000352010; OTTHUMT00000352011; OTTHUMT00000352012; OTTHUMT00000352013; OTTHUMT00000352014; OTTHUMT00000352015; uc003fbj.2; uc003fbk.2; uc003fbm.3; uc003fbn.3; uc010hvu.2; uc021xgk.1 | *VEPH1* | ventricular zone expressed PH domain containing 1 | 2.75 | 2.64 | 1.08 | 0.021 |
| TC04001226.hg.1 | NM_001253835; NM_001553; ENST00000295666; ENST00000512512; BC017201; BC066339; OTTHUMT00000250693; OTTHUMT00000362249; OTTHUMT00000362250; uc003hcn.3; uc011cag.2 | *IGFBP7* | insulin like growth factor binding protein 7 | 5.74 | 6.05 | -1.23 | 0.0211 |
| TC19002170.hg.1 | BC035704 | *PPP1R37* | protein phosphatase 1, regulatory subunit 37 | 4.73 | 4.81 | -1.06 | 0.0211 |
| TC12000095.hg.1 | NM_001098536; NM_003481; ENST00000229268; ENST00000389231; ENST00000541969; BC004889; BC005139; uc001qrh.4; uc001qri.4 | *USP5* | ubiquitin specific peptidase 5 (isopeptidase T) | 5.74 | 5.83 | -1.07 | 0.0211 |
| TC20000356.hg.1 | NM_052951; ENST00000372622; BC024290; OTTHUMT00000079499; OTTHUMT00000079500; OTTHUMT00000079501; OTTHUMT00000079502; OTTHUMT00000079503; OTTHUMT00000079504; uc002xpk.3 | *DNTTIP1* | deoxynucleotidyltransferase, terminal, interacting protein 1 | 6.91 | 6.87 | 1.03 | 0.0212 |
| TC17000613.hg.1 | NM_013351; ENST00000177694; BC039739; uc002ilv.1 | *TBX21* | T-box 21 | 6.57 | 6.84 | -1.2 | 0.0212 |
| TC04001380.hg.1 | NM_001015045; NM_014883; ENST00000264344; ENST00000395002; ENST00000502459; ENST00000503556; ENST00000508369; ENST00000509094; ENST00000511976; ENST00000513837; ENST00000515600; AK296932; AK298262; BC053569; BC058029; BC086875; OTTHUMT00000363350; OTTHUMT00000363351; OTTHUMT00000363352; OTTHUMT00000363353; OTTHUMT00000363354; OTTHUMT00000363355; OTTHUMT00000363356; OTTHUMT00000363358; OTTHUMT00000363359; OTTHUMT00000363360; OTTHUMT00000363361; OTTHUMT00000363362; OTTHUMT00000363363; OTTHUMT00000363364; OTTHUMT00000363365; OTTHUMT00000363366; OTTHUMT00000363367; OTTHUMT00000363368; OTTHUMT00000363369; OTTHUMT00000363371; OTTHUMT00000363372; OTTHUMT00000363373 | *FAM13A* | family with sequence similarity 13, member A | 4.58 | 4.43 | 1.11 | 0.0213 |
| TC11000892.hg.1 | NR_038146 | *TRIM51EP* | tripartite motif-containing 51E, pseudogene | 4.74 | 4.18 | 1.47 | 0.0213 |
| TC03003362.hg.1 | NM_001134438; NM_001134439; NM_145753; ENST00000393925; ENST00000412622; ENST00000431670; ENST00000470699; ENST00000477695; ENST00000478922; ENST00000481953; ENST00000495180; AF506820; AK125555; AL832205; BC038806; BC142678; BX647615; OTTHUMT00000354337; OTTHUMT00000354339; OTTHUMT00000354340; OTTHUMT00000354341; OTTHUMT00000354342; OTTHUMT00000354344; OTTHUMT00000354345; OTTHUMT00000354346; OTTHUMT00000354347; OTTHUMT00000354348; OTTHUMT00000354349; OTTHUMT00000354350; OTTHUMT00000354351; uc003dyd.3; uc003dye.4; uc003dyf.4; uc003dyg.3; uc003dyh.3; uc003dyi.3; uc003dyj.3; uc010hqa.3 | *PHLDB2* | pleckstrin homology-like domain, family B, member 2 | 3.32 | 3.44 | -1.09 | 0.0214 |
| TC05000149.hg.1 | NM_016568; ENST00000330120; BC095526; BC113438; OTTHUMT00000207369; uc003jic.2 | *RXFP3* | relaxin/insulin-like family peptide receptor 3 | 4.55 | 4.63 | -1.05 | 0.0214 |
| TC04001499.hg.1 | NM_001001701; NM_001170330; ENST00000399075; ENST00000504110; BC017399; OTTHUMT00000364576; OTTHUMT00000364577; uc003icv.4; uc021xrf.1 | *C4orf3* | chromosome 4 open reading frame 3 | 5.37 | 5.29 | 1.05 | 0.0215 |
| TC18000550.hg.1 | NM_005912; ENST00000299766; BC069172; BC101802; OTTHUMT00000256139; uc002lie.1 | *MC4R* | melanocortin 4 receptor | 1.93 | 2.01 | -1.05 | 0.0215 |
| TC01005631.hg.1 | NR_036634 | *TGFBR3* | transforming growth factor beta receptor III | 6.6 | 7.02 | -1.34 | 0.0215 |
| TC14000342.hg.1 | NM_001011713; ENST00000554703; ENST00000555166; ENST00000556492; AK296372; AK307348; BC118589; BC122557; uc001xcx.4; uc010aow.3; uc010trk.2 | *NAA30* | N(alpha)-acetyltransferase 30, NatC catalytic subunit | 5.57 | 5.63 | -1.04 | 0.0216 |
| TC15002531.hg.1 | NR_026891 | *FLJ10038* | uncharacterized protein FLJ10038 | 5.9 | 5.63 | 1.2 | 0.0217 |
| TC20001730.hg.1 | NM_001199534; NM_018840; NM_199483; NR_026562; ENST00000342422; ENST00000344795; ENST00000373852; BC001871; BC004446; OTTHUMT00000079006; OTTHUMT00000079007; OTTHUMT00000079008; OTTHUMT00000079009; OTTHUMT00000079010; OTTHUMT00000079011; OTTHUMT00000079012; uc002xfq.3; uc002xfr.3; uc002xfs.3; uc002xft.3 | *C20orf24* | chromosome 20 open reading frame 24 | 6.06 | 5.94 | 1.09 | 0.0218 |
| TC09002865.hg.1 | CR542066 | *CLIC3* | chloride intracellular channel 3 | 6.42 | 6.76 | -1.27 | 0.0218 |
| TC21000047.hg.1 | NR_037585; NR_037586; ENST00000440664; BC144391 | *LINC01549* | long intergenic non-protein coding RNA 1549 | 2.96 | 2.89 | 1.05 | 0.0218 |
| TC16001947.hg.1 | AK125886 | *HYDIN* | HYDIN, axonemal central pair apparatus protein | 2.48 | 2.32 | 1.12 | 0.0219 |
| TC03002464.hg.1 | AK027862 | *PHLDB2* | pleckstrin homology-like domain, family B, member 2 | 3.17 | 3.38 | -1.15 | 0.0219 |
| TC15000609.hg.1 | NM_001206836; NM_004663; ENST00000261890; BC013348; OTTHUMT00000256864; uc002apk.3; uc010ujk.2 | *RAB11A* | RAB11A, member RAS oncogene family | 7.32 | 7.23 | 1.06 | 0.0219 |
| TC09000037.hg.1 | NM_014143; ENST00000381573; ENST00000381577; ENST00000498261; AK300470; AY714881; BC069381; BC074984; BC113734; DQ286582; OTTHUMT00000051631; OTTHUMT00000051632; OTTHUMT00000051633; OTTHUMT00000207346; uc003zje.3; uc011lmb.2 | *CD274* | CD274 molecule | 4.77 | 4.57 | 1.14 | 0.022 |
| TC08002431.hg.1 | AK057998 | *LOC100288748* | uncharacterized LOC100288748 | 2.02 | 2.14 | -1.08 | 0.022 |
| TC03001976.hg.1 | NM_007217; NM_145859; NM_145860; ENST00000392750; ENST00000461494; ENST00000470131; ENST00000471885; ENST00000473645; ENST00000487678; ENST00000487947; ENST00000492396; ENST00000497056; BC002506; BC016353; OTTHUMT00000350966; OTTHUMT00000350967; OTTHUMT00000350968; OTTHUMT00000350969; OTTHUMT00000350970; OTTHUMT00000350972; OTTHUMT00000350973; OTTHUMT00000350974; OTTHUMT00000351061; OTTHUMT00000351062; OTTHUMT00000351063; OTTHUMT00000351064; OTTHUMT00000351065; OTTHUMT00000351066; OTTHUMT00000351067; OTTHUMT00000351068; OTTHUMT00000351069; OTTHUMT00000351070; uc003fex.3; uc003fey.3; uc003fez.3 | *PDCD10* | programmed cell death 10 | 5.9 | 5.81 | 1.06 | 0.022 |
| TC06001790.hg.1 | NM_001037499; ENST00000322066; OTTHUMT00000359665; uc011dwp.2 | *DEFB114* | defensin, beta 114 | 1.15 | 1.24 | -1.06 | 0.0221 |
| TC04000593.hg.1 | NR_031737; uc021xqy.1 | *MIR1973* | microRNA 1973 | 3.91 | 4.12 | -1.15 | 0.0222 |
| TC06002265.hg.1 | NM_001111077; NM_003379; ENST00000337147; ENST00000367075; ENST00000392177; ENST00000476189; AK299456; AK316031; BC013903; BC068458; OTTHUMT00000042878; OTTHUMT00000042879; OTTHUMT00000042880; uc003qrt.4; uc003qru.4; uc011efr.2; uc011efs.2 | *EZR* | ezrin | 7.89 | 8.07 | -1.13 | 0.0223 |
| TC14002194.hg.1 | NM_174913; ENST00000267425; ENST00000396802; AK123717; BC025332; OTTHUMT00000073186; OTTHUMT00000073187; uc001wol.1; uc001wom.1 | *NOP9* | NOP9 nucleolar protein | 4.78 | 4.92 | -1.1 | 0.0223 |
| TC09000193.hg.1 | NM_001080496; ENST00000378078; BC001725; OTTHUMT00000052351; OTTHUMT00000052352; uc011lpf.2 | *RGP1* | RGP1 homolog, RAB6A GEF complex partner 1 | 6.12 | 6.07 | 1.03 | 0.0223 |
| TC01003479.hg.1 | NM_000734; NM_198053; ENST00000362089; ENST00000392122; ENST00000483825; AK128376; BC025703; OTTHUMT00000083706; OTTHUMT00000083707; OTTHUMT00000083708; OTTHUMT00000083709; OTTHUMT00000083903; OTTHUMT00000083904; OTTHUMT00000083905; uc001gei.4; uc001gej.4; uc001gek.2 | *CD247* | CD247 molecule | 6.61 | 6.9 | -1.22 | 0.0224 |
| TC11001231.hg.1 | NM_001135053; NM_001135054; NM_021805; ENST00000332725; ENST00000397632; ENST00000431843; ENST00000529486; ENST00000531205; AY358342; BC003591; BC025953; BC106007; uc001lpd.2; uc001lpe.1; uc001lpf.2; uc001lpg.3 | *SIGIRR* | single immunoglobulin and toll-interleukin 1 receptor (TIR) domain | 6.57 | 6.65 | -1.05 | 0.0224 |
| TC05000453.hg.1 | NM_032290; ENST00000265140; ENST00000493934; AK297452; BC063674; OTTHUMT00000241610; OTTHUMT00000330189; OTTHUMT00000330190; OTTHUMT00000330191; OTTHUMT00000330192; OTTHUMT00000369978; OTTHUMT00000369979; OTTHUMT00000369980 | *SLF1* | SMC5-SMC6 complex localization factor 1 | 4.12 | 3.99 | 1.09 | 0.0224 |
| TC07000870.hg.1 | NM_001144920; NM_001144923; NM_024926; ENST00000343187; ENST00000430935; ENST00000464848; ENST00000474035; ENST00000478836; ENST00000481482; ENST00000495038; AK293444; AK295092; AK300807; AL137393; BC013912; BC034466; BC126331; BC130339; OTTHUMT00000348919; OTTHUMT00000348920; OTTHUMT00000348921; OTTHUMT00000348922; OTTHUMT00000348923; OTTHUMT00000348924; OTTHUMT00000349112; OTTHUMT00000349113; OTTHUMT00000349114; uc003vuq.2; uc003vur.4; uc003vus.2; uc003vut.2; uc011kqm.1; uc011kqn.1; uc011kqo.1; uc011kqp.1; uc011kqq.1 | *TTC26* | tetratricopeptide repeat domain 26 | 2.9 | 2.81 | 1.06 | 0.0224 |
| TC15001591.hg.1 | NM_001031733; NM_033429; ENST00000395463; ENST00000395465; ENST00000448060; ENST00000467889; ENST00000478113; ENST00000540479; BC009516; BX640702; BX640817; OTTHUMT00000257067; OTTHUMT00000313445; OTTHUMT00000313446; OTTHUMT00000313447; OTTHUMT00000313448; OTTHUMT00000313449; uc002arb.3; uc002arc.3; uc002ard.3; uc002are.3; uc010bhz.3 | *CALML4* | calmodulin-like 4 | 5.56 | 5.5 | 1.05 | 0.0225 |
| TC02000969.hg.1 | ENST00000439050; AK027541 | *LOC100996579* | uncharacterized LOC100996579 | 3.75 | 3.54 | 1.16 | 0.0225 |
| TC16001156.hg.1 | NR_002980 | *SNORA50A* | small nucleolar RNA, H/ACA box 50A | 7.7 | 7.34 | 1.28 | 0.0225 |
| TC07001935.hg.1 | NM_176817; ENST00000547270; BC104933; BC104937; OTTHUMT00000350810; uc003vwx.1 | *TAS2R38* | taste receptor, type 2, member 38 | 3.25 | 3.45 | -1.15 | 0.0225 |
| TC12001273.hg.1 | NM_016312; ENST00000261167; BC001621; BC023532; uc001rci.3 | *WBP11* | WW domain binding protein 11 | 7.38 | 7.47 | -1.07 | 0.0225 |
| TC08001505.hg.1 | NR_034092; AK125733 | *BAALC-AS1* | BAALC antisense RNA 1 | 3.08 | 3.12 | -1.03 | 0.0226 |
| TC13000696.hg.1 | ENST00000423442; BC020935; BC026300 | *LINC00458* | long intergenic non-protein coding RNA 458 | 3.08 | 2.99 | 1.07 | 0.0226 |
| TC17001609.hg.1 | NR_024559; uc010wjz.2 | *MAPT-AS1* | MAPT antisense RNA 1 | 5.81 | 5.97 | -1.12 | 0.0226 |
| TC18000282.hg.1 | NM_022840; ENST00000319888; AK022584; BC111020; BC136766; BC136767; OTTHUMT00000254326; uc002klh.4; uc010dkj.3 | *METTL4* | methyltransferase like 4 | 5.36 | 5.23 | 1.09 | 0.0226 |
| TC03001624.hg.1 | NM_170662; ENST00000264122; ENST00000403724; ENST00000405772; ENST00000407712; AK123147; AK302533; AK302892; BC032851; OTTHUMT00000319417; OTTHUMT00000319418; OTTHUMT00000319419; OTTHUMT00000319716; OTTHUMT00000319717; OTTHUMT00000319718; OTTHUMT00000319719; OTTHUMT00000319720; OTTHUMT00000319721; U26711; U26712; uc003dwa.3; uc003dwc.3; uc003dwd.2; uc003dwe.2; uc011bhi.2; uc011bhj.1 | *CBLB* | Cbl proto-oncogene B, E3 ubiquitin protein ligase | 6.92 | 7.12 | -1.14 | 0.0227 |
| TC05002656.hg.1 | BC023982 | *CYSTM1* | cysteine-rich transmembrane module containing 1 | 7.23 | 7 | 1.17 | 0.0227 |
| TC11001860.hg.1 | NM_002032; ENST00000273550; ENST00000526640; ENST00000529191; ENST00000529631; ENST00000532601; BC000857; BC001399; BC011359; BC013724; BC015156; BC016009; BC016857; BC063514; BC066341; BC066961; BC073750; uc001nsu.3 | *FTH1* | ferritin, heavy polypeptide 1 | 10.83 | 10.66 | 1.13 | 0.0227 |
| TC01000077.hg.1 | NR_027088; ENST00000423197; uc001alj.2 | *LOC284661* | uncharacterized LOC284661 | 3.42 | 3.34 | 1.05 | 0.0228 |
| TC14001592.hg.1 | AK093277 | *PRMT5-AS1* | PRMT5 antisense RNA 1 | 1.96 | 1.82 | 1.1 | 0.0228 |
| TC07000067.hg.1 | NM_001134387; NM_001134388; NM_001134389; NM_018106; ENST00000335965; ENST00000396706; ENST00000396707; ENST00000396709; ENST00000396713; ENST00000405731; ENST00000496017; BC001239; OTTHUMT00000059900; OTTHUMT00000207476; OTTHUMT00000207477; OTTHUMT00000207478; OTTHUMT00000324146; OTTHUMT00000324147; OTTHUMT00000324148; OTTHUMT00000324149; OTTHUMT00000324150; OTTHUMT00000324151; uc003sqh.3; uc003sqi.3; uc003sqj.3; uc003sql.3 | *ZDHHC4* | zinc finger, DHHC-type containing 4 | 5.35 | 5.38 | -1.02 | 0.0228 |
| TC09001683.hg.1 | NM_020469; ENST00000453660; AK302203; BC069595; BC069605; J05175; OTTHUMT00000054907; uc004cda.1; uc010naf.1; uc010nag.1; uc011mcz.1 | *ABO* | ABO blood group (transferase A, alpha 1-3-N-acetylgalactosaminyltransferase; transferase B, alpha 1-3-galactosyltransferase) | 4.35 | 4.53 | -1.14 | 0.023 |
| TC06001556.hg.1 | ENST00000415626 | *C2-AS1* | C2 antisense RNA 1 | 3.28 | 3.4 | -1.08 | 0.0231 |
| TC19001167.hg.1 | NM_001166215; NM_030760; ENST00000333430; ENST00000439028; BC034703; BC067781; uc002mot.2; uc002mou.2 | *S1PR5* | sphingosine-1-phosphate receptor 5 | 6.39 | 6.59 | -1.15 | 0.0231 |
| TC01002102.hg.1 | NM_014188; ENST00000291386; ENST00000359060; ENST00000378725; ENST00000378726; AK291324; AK297505; BC008070; OTTHUMT00000001366; OTTHUMT00000001367; OTTHUMT00000001368; OTTHUMT00000100389; uc001agd.3; uc001age.1; uc009vkg.1 | *SSU72* | SSU72 homolog, RNA polymerase II CTD phosphatase | 6.67 | 6.63 | 1.03 | 0.0231 |
| TC15002198.hg.1 | AB073658 | *TMOD2* | tropomodulin 2 (neuronal) | 5.28 | 4.9 | 1.3 | 0.0231 |
| TC10000896.hg.1 | NM_001609; ENST00000358776; ENST00000368869; AK298638; BC013756; OTTHUMT00000050843; OTTHUMT00000050844; uc001lhb.3; uc010qub.2 | *ACADSB* | acyl-CoA dehydrogenase, short/branched chain | 4.87 | 4.94 | -1.04 | 0.0232 |
| TC16001332.hg.1 | NM_015144; ENST00000268616; AB030243; AL117532; BC101478; OTTHUMT00000269107; uc002fjz.1; uc002fka.1; uc002fkb.3 | *ZCCHC14* | zinc finger, CCHC domain containing 14 | 5.22 | 5.32 | -1.07 | 0.0232 |
| TC13000799.hg.1 | NM_001105515; NM_005845; ENST00000376887; ENST00000467685; ENST00000536256; AF541977; AK296247; AK309460; AY133678; AY133679; AY133680; BC041560; OTTHUMT00000045478; OTTHUMT00000045479; OTTHUMT00000045480; OTTHUMT00000045481; OTTHUMT00000045482; uc001vmd.4; uc001vme.2; uc001vmf.2; uc010afj.3; uc010afk.3; uc010afl.1; uc010afm.1; uc010tih.1 | *ABCC4* | ATP binding cassette subfamily C member 4 | 5.52 | 5.25 | 1.21 | 0.0233 |
| TC09000597.hg.1 | NM_001099679; NM_012210; ENST00000373983; ENST00000450136; BC003154; OTTHUMT00000055466; OTTHUMT00000055467; uc004bjw.2; uc004bjx.2 | *TRIM32* | tripartite motif containing 32 | 4.78 | 4.87 | -1.07 | 0.0233 |
| TC07002674.hg.1 | BC006010 | *GIMAP7* | GTPase, IMAP family member 7 | 7.83 | 8.02 | -1.14 | 0.0234 |
| TC10000903.hg.1 | NM_153442; ENST00000284674; BC112011; BC113462; OTTHUMT00000050850; uc001lhh.3 | *GPR26* | G protein-coupled receptor 26 | 4.21 | 4.29 | -1.05 | 0.0234 |
| TC07000840.hg.1 | NM_018295; ENST00000275767; ENST00000466307; BC020942; OTTHUMT00000340017; OTTHUMT00000340018; uc003vsi.3 | *TMEM140* | transmembrane protein 140 | 8.55 | 8.32 | 1.17 | 0.0234 |
| TC0Y000301.hg.1 | AF527832 | *TTTY20* | testis-specific transcript, Y-linked 20 (non-protein coding) | 1.93 | 1.75 | 1.13 | 0.0234 |
| TC07001915.hg.1 | NM_001113239; NM_022740; ENST00000342645; ENST00000406875; ENST00000428878; OTTHUMT00000349430; OTTHUMT00000349431; uc003vvd.4; uc003vvf.4 | *HIPK2* | homeodomain interacting protein kinase 2 | 7.8 | 8.04 | -1.18 | 0.0235 |
| TC17001723.hg.1 | AK095112; AK126318 | *SRSF1* | serine/arginine-rich splicing factor 1 | 4.94 | 4.78 | 1.12 | 0.0235 |
| TC06001725.hg.1 | NM_018561; ENST00000373006; ENST00000373009; ENST00000373010; ENST00000394253; BC014176; OTTHUMT00000040536; OTTHUMT00000040537; OTTHUMT00000315992; OTTHUMT00000315993; OTTHUMT00000316513; uc003ori.3 | *USP49* | ubiquitin specific peptidase 49 | 3.78 | 3.72 | 1.04 | 0.0235 |
| TC06002364.hg.1 | AY566846; AY566847 | *MOG* | myelin oligodendrocyte glycoprotein | 1.15 | 1.08 | 1.05 | 0.0236 |
| TC07002496.hg.1 | AF016693 | *MUC17* | mucin 17, cell surface associated | 2.13 | 2.26 | -1.09 | 0.0237 |
| TC22000222.hg.1 | ENST00000504335 | *SMTN* | smoothelin | 2.54 | 2.69 | -1.11 | 0.0237 |
| TC06003823.hg.1 | AJ420569 | *TSPYL4* | TSPY-like 4 | 3.62 | 3.49 | 1.09 | 0.0237 |
| TC04001346.hg.1 | NM_015697; ENST00000311461; ENST00000311469; ENST00000514935; AF091086; AK300972; BC008804; BC020728; OTTHUMT00000363027; OTTHUMT00000363975; OTTHUMT00000363976; OTTHUMT00000363977; OTTHUMT00000367682; uc003hof.3; uc003hog.3; uc011ccp.2 | *COQ2* | coenzyme Q2 4-hydroxybenzoate polyprenyltransferase | 5.83 | 5.7 | 1.09 | 0.0238 |
| TC0X001539.hg.1 | NM_001327; NM_139250; ENST00000328435; ENST00000359887; BC130362; OTTHUMT00000061150; OTTHUMT00000061151; uc004fmf.1 | *CTAG1B; CTAG1A* | cancer/testis antigen 1B; cancer/testis antigen 1A | 4.99 | 5.05 | -1.05 | 0.0238 |
| TC05001601.hg.1 | NM_001145678; NM_173665; ENST00000329378; ENST00000427991; ENST00000513200; AK130941; BC035515; OTTHUMT00000254102; OTTHUMT00000370877; OTTHUMT00000371180; uc003kkn.3; uc003kkp.2; uc011cuk.2 | *KIAA0825* | KIAA0825 | 5.41 | 5.08 | 1.26 | 0.0238 |
| TC06000614.hg.1 | NM_001078174; NM_001078175; NM_001078176; NM_001078177; NM_004955; ENST00000371708; ENST00000371713; ENST00000371724; ENST00000371731; ENST00000371740; ENST00000371755; ENST00000393841; ENST00000393844; ENST00000427851; ENST00000472176; AB490708; AB490709; AK293558; AK304285; BC001382; BC008954; OTTHUMT00000040715; OTTHUMT00000040716; OTTHUMT00000040717; OTTHUMT00000040718; OTTHUMT00000040719; OTTHUMT00000040720; OTTHUMT00000040721; OTTHUMT00000040722; uc003owu.1; uc003owv.1; uc003owz.1; uc011dvp.1; uc021yzw.1; uc021yzx.1 | *SLC29A1* | solute carrier family 29 (equilibrative nucleoside transporter), member 1 | 5.81 | 5.91 | -1.07 | 0.0238 |
| TC18000128.hg.1 | NM_001943; ENST00000261590; BC099655; uc002kwu.4 | *DSG2* | desmoglein 2 | 1.87 | 1.82 | 1.04 | 0.0239 |
| TC02000581.hg.1 | NM_001008949; NM_001163523; NM_001163524; NM_178495; ENST00000361124; ENST00000439118; ENST00000536814; BC034503; BC073153; OTTHUMT00000338895; OTTHUMT00000338896; OTTHUMT00000338897; OTTHUMT00000338898; uc002svx.3; uc002svy.3; uc010yuk.2; uc010yul.2 | *ITPRIPL1* | inositol 1,4,5-trisphosphate receptor interacting protein-like 1 | 5.33 | 5.46 | -1.1 | 0.0239 |
| TC21000301.hg.1 | NR_038870; NR_038871; ENST00000430815; uc002ykm.3; uc002ykn.3 | *C21orf91-OT1* | C21orf91 overlapping transcript 1 | 3.45 | 3.35 | 1.07 | 0.024 |
| TC0X001523.hg.1 | NR_036175; uc022cib.1 | *MIR3202-2* | microRNA 3202-2 | 1.12 | 1 | 1.09 | 0.024 |
| TC06001800.hg.1 | NM_012288; ENST00000182527; AY927606; BC028121; OTTHUMT00000040910; uc003paq.3; uc003par.1 | *TRAM2* | translocation associated membrane protein 2 | 5.83 | 5.98 | -1.11 | 0.024 |
| TC05001106.hg.1 | NR_039662; uc021xwd.1 | *MIR4457* | microRNA 4457 | 1.7 | 1.9 | -1.15 | 0.0242 |
| TC05003271.hg.1 | BC042887 | *PCDHA6* | protocadherin alpha 6 | 4.11 | 4.24 | -1.09 | 0.0242 |
| TC0X001065.hg.1 | NM_001037811; NM_004493; ENST00000168216; ENST00000375298; ENST00000375304; ENST00000495986; BC000372; BC008708; OTTHUMT00000056750; OTTHUMT00000056751; OTTHUMT00000056752; OTTHUMT00000056753; OTTHUMT00000056754; uc004dsl.1; uc004dsm.1 | *HSD17B10* | hydroxysteroid (17-beta) dehydrogenase 10 | 6.53 | 6.6 | -1.04 | 0.0243 |
| TC12000704.hg.1 | ENST00000548172; ENST00000549313; ENST00000549470 | *LOC105369893* | uncharacterized LOC105369893 | 2.95 | 2.9 | 1.04 | 0.0243 |
| TC14001337.hg.1 | BC038792 | *LOC105370580* | uncharacterized LOC105370580 | 1.34 | 1.21 | 1.1 | 0.0243 |
| TC08000713.hg.1 | ENST00000362808 | *RNY4P5* | RNA, Ro-associated Y4 pseudogene 5 | 4.59 | 4.36 | 1.18 | 0.0243 |
| TC03001990.hg.1 | BC131773 | *PHC3* | polyhomeotic homolog 3 (Drosophila) | 5.36 | 5.22 | 1.1 | 0.0244 |
| TC17001361.hg.1 | NM_207454; BC133004; BC142651; OTTHUMT00000346435; uc002hie.1 | *C17orf102* | chromosome 17 open reading frame 102 | 2.75 | 2.65 | 1.07 | 0.0245 |
| TC11000133.hg.1 | NM_001014794; NM_001014795; NM_004517; ENST00000299421; ENST00000396751; ENST00000420936; ENST00000526711; ENST00000528995; ENST00000537806; AK293474; AK296628; BC001554; OTTHUMT00000257264; uc001mee.3; uc001mef.3; uc001meh.3; uc010rap.2; uc010raq.2 | *ILK* | integrin linked kinase | 7.86 | 7.77 | 1.06 | 0.0245 |
| TC03001650.hg.1 | NM_022488; ENST00000283290; ENST00000402314; ENST00000495756; AK308919; BC002830; BC024221; OTTHUMT00000354147; OTTHUMT00000354148; OTTHUMT00000354149; OTTHUMT00000354150; OTTHUMT00000354151; OTTHUMT00000354152; OTTHUMT00000354153; OTTHUMT00000354154; OTTHUMT00000354155; uc003dzc.3; uc003dzd.3; uc010hqe.3 | *ATG3* | autophagy related 3 | 6.64 | 6.56 | 1.06 | 0.0246 |
| TC21000096.hg.1 | NR_027072; ENST00000420364; BC065252; OTTHUMT00000171969; OTTHUMT00000171970; OTTHUMT00000171971; uc002ynh.3; uc002yni.3 | *LINC00189* | long intergenic non-protein coding RNA 189 | 5.12 | 4.45 | 1.58 | 0.0246 |
| TC13000660.hg.1 | NM_001079670; NM_030925; ENST00000347776; ENST00000355854; ENST00000409130; ENST00000409308; ENST00000410043; ENST00000476943; AY288977; BC010993; OTTHUMT00000044908; OTTHUMT00000044909; OTTHUMT00000044910; OTTHUMT00000044911; OTTHUMT00000335456; OTTHUMT00000335457; OTTHUMT00000335458; OTTHUMT00000335459; OTTHUMT00000335460; OTTHUMT00000335461; uc001vcw.3; uc001vcx.3; uc010adf.3 | *CAB39L* | calcium binding protein 39-like | 3.08 | 3.01 | 1.06 | 0.0247 |
| TC10000452.hg.1 | NM_138357; ENST00000357157; ENST00000373053; ENST00000483185; ENST00000536019; AK128016; AK301475; AK310455; AK311133; BC010682; BC034235; OTTHUMT00000048593; OTTHUMT00000048594; OTTHUMT00000048595; uc001jtc.3; uc001jtd.3; uc009xqp.1; uc009xqq.1; uc009xqr.3 | *MCU* | mitochondrial calcium uniporter | 5.8 | 5.63 | 1.12 | 0.0247 |
| TC17000979.hg.1 | NM_003693; NM_145350; NM_145352; NR_028075; NR_028076; ENST00000263071; ENST00000348987; ENST00000434376; BC039735; OTTHUMT00000207081 | *SCARF1* | scavenger receptor class F, member 1 | 6.12 | 6 | 1.09 | 0.0247 |
| TC01001982.hg.1 | NR_039824; uc021plt.1 | *MIR4677* | microRNA 4677 | 5.29 | 5.05 | 1.18 | 0.0248 |
| TC05000307.hg.1 | AK311046 | *NAIP* | NLR family, apoptosis inhibitory protein | 8.65 | 8.11 | 1.46 | 0.0248 |
| TC09000545.hg.1 | NM_001244713; NM_001244724; NM_002874; ENST00000358015; ENST00000416373; AK293532; BC020973; OTTHUMT00000053548; OTTHUMT00000053549; OTTHUMT00000053550; OTTHUMT00000053551; uc004bde.3; uc011lwa.2; uc011lwb.2; uc022blj.1 | *RAD23B* | RAD23 homolog B, nucleotide excision repair protein | 7.11 | 7.06 | 1.03 | 0.0248 |
| TC02001430.hg.1 | NM_001037131; NM_001244888; NM_014914; ENST00000304032; ENST00000336665; ENST00000409457; ENST00000409538; ENST00000428334; BC140856; BC167153; BC167157; OTTHUMT00000257076; OTTHUMT00000257077; OTTHUMT00000329672; OTTHUMT00000329673; OTTHUMT00000329674; OTTHUMT00000329675; OTTHUMT00000329676; OTTHUMT00000329677; OTTHUMT00000329678; OTTHUMT00000329679; uc002vvs.3; uc002vvt.3; uc021vyp.1 | *AGAP1* | ArfGAP with GTPase domain, ankyrin repeat and PH domain 1 | 5.21 | 5.36 | -1.11 | 0.0249 |
| TC04002813.hg.1 | AK021429 | *SH3RF1* | SH3 domain containing ring finger 1 | 4.38 | 4.51 | -1.09 | 0.0249 |
| TC01005834.hg.1 | M21302 | *SPRR2D* | small proline-rich protein 2D | 2.62 | 2.75 | -1.1 | 0.0249 |
| TC17001296.hg.1 | NM_000638; NM_001080837; ENST00000226218; ENST00000438614; ENST00000536498; BC005046; OTTHUMT00000255680; uc002hbc.3; uc010wai.1 | *VTN; SEBOX* | vitronectin; SEBOX homeobox | 4.84 | 4.87 | -1.02 | 0.0249 |
| TC07000893.hg.1 | NM_016943; ENST00000247879; BC069337; BC095523; OTTHUMT00000349288; uc003vwp.1 | *TAS2R3* | taste receptor, type 2, member 3 | 4.45 | 4.12 | 1.26 | 0.025 |
| TC03000770.hg.1 | NM_001251845; NM_003304; ENST00000273482; ENST00000476941; BC112338; BC113953; GQ293239; OTTHUMT00000354476; OTTHUMT00000354520; OTTHUMT00000354522; OTTHUMT00000354523; uc003evb.3; uc003evc.3; uc011bni.1 | *TRPC1* | transient receptor potential cation channel, subfamily C, member 1 | 2.5 | 2.45 | 1.04 | 0.025 |
| TC22000327.hg.1 | NM_001204827; NM_014248; NM_022098; ENST00000216225; ENST00000357137; ENST00000482652; AK301635; AK310502; AL834310; BC001208; BC001466; BC001681; BC045166; OTTHUMT00000322149; OTTHUMT00000322150; OTTHUMT00000322151; OTTHUMT00000322201; OTTHUMT00000322202; OTTHUMT00000322204; OTTHUMT00000322205; OTTHUMT00000322206; uc003azf.2; uc003azg.2; uc003azh.3; uc003azi.3; uc003azk.3; uc010gyh.1; uc011aox.2 | *XPNPEP3; RBX1* | X-prolyl aminopeptidase 3, mitochondrial; ring-box 1, E3 ubiquitin protein ligase | 5.18 | 5.11 | 1.05 | 0.025 |
| TC19001166.hg.1 | NM_012289; NM_203500; ENST00000171111; ENST00000393623; BC002417; BC002930; BC014118; BC015945; uc002mop.1; uc002moq.1; uc002mor.1 | *KEAP1* | kelch-like ECH-associated protein 1 | 5.95 | 6 | -1.04 | 0.0251 |
| TC09002629.hg.1 | NR_040117 | *LOC392364* | nuclear pore associated protein 1 pseudogene | 3.97 | 4.14 | -1.13 | 0.0251 |
| TC03002064.hg.1 | NM_001023587; NM_005688; ENST00000265586; ENST00000334444; ENST00000382494; ENST00000392579; ENST00000427120; ENST00000446941; ENST00000492216; AY196484; AY754874; AY754876; BC051358; BC140771; BC142670; BC144495; OTTHUMT00000346350; OTTHUMT00000346351; OTTHUMT00000346352; OTTHUMT00000346353; OTTHUMT00000346354; OTTHUMT00000346355; OTTHUMT00000346356; OTTHUMT00000346358; OTTHUMT00000346359; OTTHUMT00000346360; OTTHUMT00000346361; OTTHUMT00000346362; uc003fmg.3; uc003fmh.3; uc003fmi.3; uc010hxl.3; uc010hxm.3; uc010hxn.3; uc010hxo.3; uc011bqt.2 | *ABCC5* | ATP binding cassette subfamily C member 5 | 5.7 | 5.51 | 1.14 | 0.0252 |
| TC01003618.hg.1 | BC071857 | *ARPC5* | actin related protein 2/3 complex subunit 5 | 6.55 | 6.39 | 1.12 | 0.0253 |
| TC01000460.hg.1 | OTTHUMT00000012733 | *RP5-983H21.3* |  | 3.36 | 3.44 | -1.05 | 0.0253 |
| TC11002277.hg.1 | CR936778; NR_034154 | *COLCA1* | colorectal cancer associated 1 | 3.7 | 3.79 | -1.06 | 0.0254 |
| TC01000721.hg.1 | NR_002771; OTTHUMT00000024916; uc001dbg.1 | *DLEU2L* | deleted in lymphocytic leukemia 2-like | 3.41 | 3.26 | 1.11 | 0.0255 |
| TC21000951.hg.1 | NR_040084 | *LOC100133286* | uncharacterized LOC100133286 | 3.91 | 3.83 | 1.06 | 0.0255 |
| TC22001424.hg.1 | NM_001193289; NM_145699; ENST00000249116; ENST00000402255; ENST00000495988; BC126416; BC144146; OTTHUMT00000320915; OTTHUMT00000321237; OTTHUMT00000321238; uc003awn.2; uc011aob.1 | *APOBEC3A_B; APOBEC3A* | APOBEC3A and APOBEC3B deletion hybrid; apolipoprotein B mRNA editing enzyme, catalytic polypeptide-like 3A | 8.5 | 8.26 | 1.18 | 0.0256 |
| TC19001563.hg.1 | NM_001256213; NM_001256214; NM_152296; ENST00000302102; ENST00000543770; ENST00000545399; AK295833; AK296557; AK316069; BC009282; BC013763; OTTHUMT00000268107; OTTHUMT00000268108; OTTHUMT00000268109; OTTHUMT00000268111; OTTHUMT00000268112; OTTHUMT00000268113; OTTHUMT00000268114; OTTHUMT00000268115; uc010xwf.2; uc010xwg.2 | *ATP1A3* | ATPase, Na+/K+ transporting, alpha 3 polypeptide | 5.45 | 5.53 | -1.05 | 0.0256 |
| TC04000643.hg.1 | NM_014278; ENST00000296464; ENST00000505726; ENST00000508776; ENST00000515262; AK302115; AK307464; BC040560; OTTHUMT00000257096; OTTHUMT00000364141; OTTHUMT00000364142; OTTHUMT00000364143; OTTHUMT00000364144; uc003ifm.3; uc010iny.1; uc011cgr.2 | *HSPA4L* | heat shock 70kDa protein 4-like | 1.56 | 1.51 | 1.03 | 0.0256 |
| TC16000682.hg.1 | NR_024399; NR_024402; BX640980; uc002flh.2; uc002fli.4; uc010chy.3 | *SNAI3-AS1* | SNAI3 antisense RNA 1 | 5.08 | 5.17 | -1.06 | 0.0256 |
| TC0X000407.hg.1 | ENST00000373488 | *BMP2KL* | BMP2 inducible kinase-like | 2.72 | 2.59 | 1.09 | 0.0257 |
| TC09000139.hg.1 | NM_001195536; NM_016410; ENST00000223500; ENST00000419016; ENST00000487080; BC006974; BC007457; BC016698; BC021168; OTTHUMT00000052040; OTTHUMT00000052041; uc003zsm.4; uc011lnv.2 | *CHMP5* | charged multivesicular body protein 5 | 5.53 | 5.42 | 1.08 | 0.0258 |
| TC09000640.hg.1 | NR_038975; AB074162; OTTHUMT00000054033; uc022bni.1 | *MIR181A2HG* | MIR181A2 host gene | 3.84 | 3.99 | -1.11 | 0.0258 |
| TC06002906.hg.1 | AK124105 | *RIPPLY2* | ripply transcriptional repressor 2 | 1.63 | 1.52 | 1.07 | 0.0258 |
| TC11002677.hg.1 | M13203 | *SERPING1* | serpin peptidase inhibitor, clade G (C1 inhibitor), member 1 | 6.34 | 5.8 | 1.45 | 0.0258 |
| TC04000517.hg.1 | NM_014395; ENST00000296414; ENST00000512369; AF186022; AK301355; BC012924; OTTHUMT00000363214; OTTHUMT00000363215; OTTHUMT00000363216; OTTHUMT00000363217; uc003hvf.4; uc010ilh.3; uc011cek.2 | *DAPP1* | dual adaptor of phosphotyrosine and 3-phosphoinositides | 6.89 | 6.74 | 1.11 | 0.0259 |
| TC12001359.hg.1 | ENST00000542490; BC029816; BC039117; uc010sjy.1 | *OVOS; OVOS2* | ovostatin; ovostatin 2 | 2.14 | 2.03 | 1.08 | 0.0259 |
| TC07000161.hg.1 | NM_001199835; NM_001199837; NM_001199838; NM_013322; NR_037670; ENST00000338523; ENST00000396376; ENST00000409367; ENST00000409838; ENST00000446848; ENST00000462993; BC031050; BC034992; OTTHUMT00000214119; OTTHUMT00000214120; OTTHUMT00000327997; OTTHUMT00000327998; OTTHUMT00000327999; OTTHUMT00000328000; OTTHUMT00000328001; uc003sxx.3; uc010kuu.3; uc010kuv.3; uc010kuw.3; uc011jzg.2 | *SNX10* | sorting nexin 10 | 6.34 | 6.19 | 1.11 | 0.0259 |
| TC03001959.hg.1 | NM_001040100; ENST00000359175; ENST00000497137; BC065208; BC107756; BC126236; OTTHUMT00000353181; OTTHUMT00000353182; OTTHUMT00000353183; uc003fee.3 | *SPTSSB* | serine palmitoyltransferase, small subunit B | 3.66 | 3.75 | -1.07 | 0.0259 |
| TC09001609.hg.1 | NM_000118; NM_001114753; ENST00000344849; ENST00000373203; ENST00000480266; AK301171; BC014271; OTTHUMT00000054312; OTTHUMT00000054313; OTTHUMT00000054314; OTTHUMT00000054316 | *ENG* | endoglin | 6.59 | 6.75 | -1.12 | 0.026 |
| TC18000519.hg.1 | BC034434 | *LOC101928167* | uncharacterized LOC101928167 | 1.93 | 1.76 | 1.13 | 0.026 |
| TC02001556.hg.1 | NM_138799; ENST00000305997; ENST00000486484; AK295907; BC146871; OTTHUMT00000206735; OTTHUMT00000353388; OTTHUMT00000353389; OTTHUMT00000353390; OTTHUMT00000353391; OTTHUMT00000353392; OTTHUMT00000353393; OTTHUMT00000353394; OTTHUMT00000353395; OTTHUMT00000353396; uc002qzg.1; uc010yix.1 | *MBOAT2* | membrane bound O-acyltransferase domain containing 2 | 5.72 | 5.44 | 1.21 | 0.026 |
| TC16000313.hg.1 | NM_032815; NR_039742; ENST00000320805; AK027545; AK304172; AK304606; BC068007; BC101741; OTTHUMT00000214999; uc002drt.3; uc002dru.3; uc002drv.3; uc010vdg.1; uc010vdh.2; uc021tfu.1 | *NFATC2IP; MIR4517* | nuclear factor of activated T-cells, cytoplasmic, calcineurin-dependent 2 interacting protein; microRNA 4517 | 5.6 | 5.67 | -1.05 | 0.026 |
| TC09000741.hg.1 | NM_005157; NM_007313; ENST00000318560; BC117451; OTTHUMT00000054684; OTTHUMT00000054685; OTTHUMT00000054686; uc004bzv.3; uc004bzw.3 | *ABL1* | ABL proto-oncogene 1, non-receptor tyrosine kinase | 6.22 | 6.29 | -1.05 | 0.0261 |
| TC11000009.hg.1 | NM_025092; ENST00000409479; ENST00000409548; ENST00000409655; AL832932; BC048336; BC066641; BC109257; OTTHUMT00000330161; OTTHUMT00000330162; OTTHUMT00000330163; OTTHUMT00000330164; OTTHUMT00000330165; uc001lor.4; uc001lou.4; uc001lov.4; uc010qvu.2 | *ATHL1* | ATH1, acid trehalase-like 1 (yeast) | 6.68 | 6.54 | 1.11 | 0.0261 |
| TC12000949.hg.1 | NM_014730; ENST00000228506; ENST00000412616; ENST00000535413; BC000371; BC016297; uc001tyy.1 | *MLEC* | malectin | 6.64 | 6.74 | -1.07 | 0.0261 |
| TC13000632.hg.1 | NR_002967; ENST00000362607; uc001vaa.1 | *SNORA31* | small nucleolar RNA, H/ACA box 31 | 5.1 | 4.89 | 1.16 | 0.0261 |
| TC11002045.hg.1 | ENST00000508969; BC073927; uc001oqx.1 | *ALG1L9P* | asparagine-linked glycosylation 1-like 9, pseudogene | 4.96 | 5.02 | -1.04 | 0.0262 |
| TC02001747.hg.1 | AK057187 | *LINC00211* | long intergenic non-protein coding RNA 211 | 4.21 | 4.08 | 1.1 | 0.0262 |
| TC01006361.hg.1 | NM_004120; ENST00000370466; ENST00000463660; BC022272; BC073163; OTTHUMT00000029406; OTTHUMT00000029407; OTTHUMT00000029408; uc001dmz.1 | *GBP2* | guanylate binding protein 2, interferon-inducible | 8.45 | 8.17 | 1.21 | 0.0263 |
| TC08001700.hg.1 | NM_001077527; NM_003724; BC043351; BC071862; OTTHUMT00000362912; OTTHUMT00000362914; OTTHUMT00000362918; OTTHUMT00000362919; OTTHUMT00000362920; uc003ywo.3; uc003ywp.3; uc022bcb.1 | *JRK* | Jrk helix-turn-helix protein | 5.35 | 5.4 | -1.03 | 0.0263 |
| TC01004748.hg.1 | AF086289 | *LCE2B* | late cornified envelope 2B | 5.16 | 5.24 | -1.06 | 0.0263 |
| TC01002219.hg.1 | ENST00000436041 | *PRAMEF27* | PRAME family member 27 | 3.13 | 3.01 | 1.09 | 0.0263 |
| TC06002887.hg.1 | BC036479 | *LOC105379704* | CASP-like protein 4A1 | 3.29 | 3.39 | -1.07 | 0.0266 |
| TC09000642.hg.1 | NR_029782; ENST00000385004; uc004bou.3 | *MIR181B2* | microRNA 181b-2 | 3.58 | 3.92 | -1.26 | 0.0266 |
| TC01000561.hg.1 | NM_018150; ENST00000355387; ENST00000361799; ENST00000372247; ENST00000480686; AK001459; AK225180; AK293411; AK296038; AK297228; BC000279; BC034221; BC098266; BC098300; OTTHUMT00000020683; OTTHUMT00000021572; OTTHUMT00000021573; OTTHUMT00000021574; OTTHUMT00000021575; OTTHUMT00000021576; OTTHUMT00000021577; OTTHUMT00000021578; OTTHUMT00000021579; OTTHUMT00000021580; OTTHUMT00000021581; OTTHUMT00000021582; OTTHUMT00000021583; OTTHUMT00000021584; OTTHUMT00000021585; OTTHUMT00000021586; OTTHUMT00000021587; uc001clv.1; uc001clw.1; uc001clx.2; uc001cma.1; uc010okx.1; uc010oky.1; uc010okz.2; uc021omt.1 | *RNF220* | ring finger protein 220 | 5.78 | 5.86 | -1.06 | 0.0266 |
| TC06002238.hg.1 | NM_033071; NM_182961; ENST00000341594; ENST00000347037; ENST00000354674; ENST00000367248; ENST00000367253; ENST00000367255; ENST00000413186; ENST00000423061; ENST00000448038; ENST00000466159; ENST00000495090; ENST00000539504; AB033088; AB051543; AK304825; AK308717; AK310977; AK316101; AL713682; AY061755; BC028616; BC039121; BX537517; BX647837; CR933676; FM162565; OTTHUMT00000042729; OTTHUMT00000042730; OTTHUMT00000042731; OTTHUMT00000042732; OTTHUMT00000042733; OTTHUMT00000042734; OTTHUMT00000042735; OTTHUMT00000042736; OTTHUMT00000042738; OTTHUMT00000042745; OTTHUMT00000042746; OTTHUMT00000042747; OTTHUMT00000334755; OTTHUMT00000334756; OTTHUMT00000334757; OTTHUMT00000334758; OTTHUMT00000334759; OTTHUMT00000334760; OTTHUMT00000334761; OTTHUMT00000334762; OTTHUMT00000334763; OTTHUMT00000334764; OTTHUMT00000334765; OTTHUMT00000334766; OTTHUMT00000334767; OTTHUMT00000334768; uc003qoq.4; uc003qor.4; uc003qos.4; uc003qot.4; uc003qou.4; uc003qov.3; uc003qow.3; uc003qox.1; uc003qoy.2; uc003qoz.2; uc003qpa.1; uc010kiy.1; uc010kja.2; uc010kjb.1; uc011eez.2 | *SYNE1* | spectrin repeat containing, nuclear envelope 1 | 6.24 | 6.52 | -1.22 | 0.0266 |
| TC01005268.hg.1 | DQ426869 | *DHRS3* | dehydrogenase/reductase (SDR family) member 3 | 6.92 | 7.09 | -1.13 | 0.0267 |
| TC11000735.hg.1 | NM_001012503; ENST00000398536; BC136967; OTTHUMT00000127953; uc001oqq.1 | *KRTAP5-7* | keratin associated protein 5-7 | 4.42 | 4.49 | -1.06 | 0.0267 |
| TC19000506.hg.1 | BC024306 | *LOC728485* | uncharacterized LOC728485 | 4.56 | 4.72 | -1.12 | 0.0268 |
| TC12001533.hg.1 | NM_175078; ENST00000341809; BC118598; BC122558; uc001saw.3; uc009zmi.3 | *KRT77* | keratin 77, type II | 4.57 | 4.7 | -1.1 | 0.0269 |
| TC22001370.hg.1 | NR_027779 | *TTLL1* | tubulin tyrosine ligase-like family member 1 | 4.57 | 4.65 | -1.06 | 0.0269 |
| TC10000872.hg.1 | NR_039830; uc021pzs.1 | *MIR4682* | microRNA 4682 | 5.66 | 5.83 | -1.13 | 0.027 |
| TC17001110.hg.1 | NR_026951; ENST00000315707; OTTHUMT00000256341; uc002gkp.4 | *LINC00324* | long intergenic non-protein coding RNA 324 | 5.38 | 5.29 | 1.06 | 0.0271 |
| TC17001308.hg.1 | NR_029970; ENST00000385059; uc021ttv.1 | *MIR451A; MIR451B* | microRNA 451a; microRNA 451b | 1.31 | 1.2 | 1.08 | 0.0271 |
| TC01002342.hg.1 | NM_003196; ENST00000374601; ENST00000450454; ENST00000476978; AK300707; AK310039; BC041613; OTTHUMT00000008911; OTTHUMT00000008912; OTTHUMT00000008913; OTTHUMT00000008914; OTTHUMT00000008915; uc009vqm.2; uc010ody.1; uc021oig.1; uc021oih.1 | *TCEA3* | transcription elongation factor A (SII), 3 | 5.16 | 5.07 | 1.06 | 0.0271 |
| TC07000384.hg.1 | NM_001013746; NM_016220; ENST00000344930; ENST00000395391; ENST00000423627; BC047243; OTTHUMT00000251593; OTTHUMT00000344740; OTTHUMT00000344741; uc003ttd.3; uc003tte.3 | *ZNF107* | zinc finger protein 107 | 6.81 | 6.55 | 1.2 | 0.0271 |
| TC19001488.hg.1 | NM_001172677; NM_032689; ENST00000355202; ENST00000395835; BC014850; uc002ohb.2; uc002ohc.2 | *ZNF607* | zinc finger protein 607 | 3.83 | 3.94 | -1.08 | 0.0271 |
| TC11001941.hg.1 | NM_138368; ENST00000532090; BC109109 | *AP5B1* | adaptor-related protein complex 5, beta 1 subunit | 6.71 | 6.53 | 1.14 | 0.0272 |
| TC19000332.hg.1 | NM_024050; ENST00000359866; BC000615; uc002ngd.3 | *DDA1* | DET1 and DDB1 associated 1 | 7.12 | 7.17 | -1.04 | 0.0272 |
| TC11001009.hg.1 | NM_000317; ENST00000280362; ENST00000524931; ENST00000525803; AK310768; BC009686; BC018029; uc001pnj.4; uc009yyo.3 | *PTS* | 6-pyruvoyltetrahydropterin synthase | 5.07 | 4.99 | 1.06 | 0.0272 |
| TC04001399.hg.1 | NM_174952; ENST00000295268; ENST00000506482; BC036870; OTTHUMT00000253642; OTTHUMT00000364068; OTTHUMT00000381036; uc003htt.2 | *STPG2* | sperm-tail PG-rich repeat containing 2 | 1.49 | 1.46 | 1.02 | 0.0272 |
| TC09000357.hg.1 | NM_001098802; NM_032171; ENST00000277082; ENST00000376597; ENST00000376598; ENST00000415759; ENST00000424347; ENST00000487108; AK022705; AK303457; BC091515; BC128058; OTTHUMT00000052766; OTTHUMT00000052767; OTTHUMT00000052768; OTTHUMT00000052769; OTTHUMT00000052770; OTTHUMT00000052771; OTTHUMT00000052772; OTTHUMT00000052773; uc004akx.2; uc004aky.4; uc004akz.1; uc010mpp.3; uc011lsp.1 | *CEP78* | centrosomal protein 78kDa | 4.95 | 5.09 | -1.1 | 0.0273 |
| TC01000314.hg.1 | NM_013943; ENST00000374379; ENST00000497755; AK095959; BC012444; OTTHUMT00000009332; OTTHUMT00000009333; OTTHUMT00000009334; OTTHUMT00000009335; uc001bjo.2; uc001bjp.1; Z24749 | *CLIC4* | chloride intracellular channel 4 | 5.6 | 5.37 | 1.18 | 0.0273 |
| TC17001933.hg.1 | NM_014740; ENST00000269349; BC003662; BC004386; BC011151; uc002jxs.3; uc010wuc.2; X79538 | *EIF4A3* | eukaryotic translation initiation factor 4A3 | 6.38 | 6.44 | -1.04 | 0.0273 |
| TC01002011.hg.1 | AK130400 | *GCSAML* | germinal center-associated, signaling and motility-like | 3.91 | 3.78 | 1.1 | 0.0273 |
| TC01002395.hg.1 | NM_018066; ENST00000374133; ENST00000374135; ENST00000477418; BC007815; BC008634; OTTHUMT00000012175; OTTHUMT00000012176; OTTHUMT00000012177; OTTHUMT00000012178; OTTHUMT00000012179; uc001bnd.1 | *GPN2* | GPN-loop GTPase 2 | 5.65 | 5.6 | 1.04 | 0.0273 |
| TC17001455.hg.1 | NM_012481; NM_183228; NM_183229; NM_183230; NM_183231; NM_183232; ENST00000293068; ENST00000346243; ENST00000346872; ENST00000350532; ENST00000351680; ENST00000377944; ENST00000377945; ENST00000377952; ENST00000377958; ENST00000394189; ENST00000467757; ENST00000535189; AK301250; AY377973; AY377975; AY377976; AY377977; AY377978; AY377979; AY377980; AY377981; AY377982; BC032707; OTTHUMT00000257004; OTTHUMT00000257005; OTTHUMT00000257006; OTTHUMT00000257007; OTTHUMT00000257008; OTTHUMT00000257009; OTTHUMT00000257010; OTTHUMT00000257011; OTTHUMT00000257012; OTTHUMT00000257013; OTTHUMT00000257014; OTTHUMT00000257015; OTTHUMT00000257016; OTTHUMT00000257017 | *IKZF3* | IKAROS family zinc finger 3 | 7.61 | 7.83 | -1.16 | 0.0273 |
| TC01001938.hg.1 | NR_033927; ENST00000429269; uc021pkr.1 | *LINC00184* | long intergenic non-protein coding RNA 184 | 2.52 | 2.74 | -1.16 | 0.0273 |
| TC07001562.hg.1 | NM_000601; NM_001010931; NM_001010932; NM_001010933; NM_001010934; ENST00000222390; ENST00000354224; ENST00000423064; ENST00000444829; ENST00000453018; ENST00000453411; ENST00000457544; BC130284; FJ830862; OTTHUMT00000253315; OTTHUMT00000337133; OTTHUMT00000337134; OTTHUMT00000337135; OTTHUMT00000337136; OTTHUMT00000337169; OTTHUMT00000337170; OTTHUMT00000337171; OTTHUMT00000337172; uc003uhl.3; uc003uhm.3; uc003uhn.1; uc003uho.1; uc003uhp.3; uc022agw.1 | *HGF* | hepatocyte growth factor (hepapoietin A; scatter factor) | 4 | 3.81 | 1.14 | 0.0274 |
| TC12001254.hg.1 | NR_024061; ENST00000381800; uc001rak.3 | *LOH12CR2* | loss of heterozygosity, 12, chromosomal region 2 (non-protein coding) | 2.88 | 3.02 | -1.1 | 0.0274 |
| TC07000584.hg.1 | NM_001159491; NM_015379; ENST00000297290; ENST00000473967; ENST00000539286; BC018737; BC062370; OTTHUMT00000334674; OTTHUMT00000334675; OTTHUMT00000334676; uc003upi.2; uc011kip.1 | *BRI3* | brain protein I3 | 6.7 | 6.61 | 1.06 | 0.0275 |
| TC11000499.hg.1 | NM_001178040; NM_004177; ENST00000300150; ENST00000337979; ENST00000529177; ENST00000530221; AJ002077; AK293494; BC007405; uc001nog.3; uc009ymt.1; uc010rkx.2; uc010rky.2 | *STX3* | syntaxin 3 | 7.97 | 7.76 | 1.16 | 0.0275 |
| TC07001956.hg.1 | OTTHUMT00000351233 | *TRBV9* | T cell receptor beta variable 9 | 5.69 | 5.91 | -1.16 | 0.0275 |
| TC12000637.hg.1 | NM_001136262; ENST00000519948; BC009111; OTTHUMT00000376473; uc001sxd.4 | *ATXN7L3B* | ataxin 7-like 3B | 6.23 | 6.3 | -1.05 | 0.0276 |
| TC09001542.hg.1 | NM_001735; ENST00000223642; ENST00000466280; AK310774; AK310780; BC113738; BC113740; OTTHUMT00000053844; OTTHUMT00000053845; OTTHUMT00000053846; OTTHUMT00000053847; OTTHUMT00000053848; uc004bkv.3; uc010mvm.1; uc010mvn.1 | *C5* | complement component 5 | 2.89 | 2.8 | 1.06 | 0.0276 |
| TC03003289.hg.1 | AF058804 | *MUC4* | mucin 4, cell surface associated | 4.98 | 5.21 | -1.17 | 0.0276 |
| TC20001202.hg.1 | D82057 | *MYL9* | myosin light chain 9 | 8 | 7.66 | 1.27 | 0.0276 |
| TC09002585.hg.1 | AL713639 | *APBA1* | amyloid beta (A4) precursor protein-binding, family A, member 1 | 2.47 | 2.58 | -1.08 | 0.0277 |
| TC01005636.hg.1 | uc001dqd.1 | *MIG7* | mig-7 | 2.83 | 2.92 | -1.07 | 0.0277 |
| TC05000231.hg.1 | NM_006144; ENST00000274306; BC015739; OTTHUMT00000214100; uc003jpm.3 | *GZMA* | granzyme A | 5.88 | 6.33 | -1.37 | 0.0278 |
| TC20000046.hg.1 | BC008667 | *PANK2* | pantothenate kinase 2 | 5.28 | 5.05 | 1.17 | 0.0278 |
| TC03001689.hg.1 | NM_001168271; NM_153002; ENST00000315843; ENST00000461057; ENST00000464295; BC113701; BC143606; OTTHUMT00000355138; OTTHUMT00000355139; OTTHUMT00000355140; OTTHUMT00000355141; uc011bjf.2; uc011bjg.2 | *GPR156* | G protein-coupled receptor 156 | 3.1 | 3.2 | -1.08 | 0.0279 |
| TC21000469.hg.1 | NM_182832; OTTHUMT00000195066; uc002yyz.3 | *PLAC4* | placenta specific 4 | 3.05 | 3.1 | -1.04 | 0.0279 |
| TC16001775.hg.1 | NR_024050 | *BCAR4* | breast cancer anti-estrogen resistance 4 (non-protein coding) | 2.31 | 2.25 | 1.04 | 0.028 |
| TC01000730.hg.1 | NM_001003679; NM_001003680; NM_001198681; NM_001198683; NM_001198687; NM_001198688; NM_001198689; NM_002303; NM_017526; ENST00000344610; ENST00000349533; ENST00000371058; ENST00000371059; ENST00000371060; ENST00000371065; ENST00000406510; ENST00000462765; ENST00000484243; BC056250; BC131779; OTTHUMT00000025132; OTTHUMT00000025133; OTTHUMT00000025263; OTTHUMT00000025264; OTTHUMT00000025265; OTTHUMT00000025275; OTTHUMT00000025276; OTTHUMT00000025277; OTTHUMT00000025278; OTTHUMT00000025279; OTTHUMT00000025280; uc001dcf.3; uc001dcg.3; uc001dch.3; uc001dci.3; uc001dcj.3; uc001dck.3; uc009wao.3; uc009wap.3; uc009waq.3; uc021ool.1 | *LEPR; LEPROT* | leptin receptor; leptin receptor overlapping transcript | 4.6 | 4.53 | 1.05 | 0.028 |
| TC0X000205.hg.1 | ENST00000440955; AK056105 | *LOC101927501* | uncharacterized LOC101927501 | 1.89 | 1.8 | 1.06 | 0.028 |
| TC15000950.hg.1 | NR_039864; uc021sxh.1 | *MIR4714* | microRNA 4714 | 1.66 | 1.84 | -1.14 | 0.028 |
| TC20001070.hg.1 | BC008667 | *PANK2* | pantothenate kinase 2 | 5.97 | 5.67 | 1.23 | 0.028 |
| TC11001311.hg.1 | NM_018073; ENST00000300747; AK301120; AK310888; BC075058; BC109063; uc001lzf.2; uc009yek.2; uc010qyj.2 | *TRIM68* | tripartite motif containing 68 | 5.39 | 5.49 | -1.07 | 0.028 |
| TC0X001273.hg.1 | NM_001025580; NM_001171689; NM_015365; ENST00000262844; ENST00000372057; ENST00000372059; ENST00000496695; BC060813; OTTHUMT00000057907; OTTHUMT00000057908; OTTHUMT00000057909; OTTHUMT00000057910; OTTHUMT00000355125; uc004eoo.3; uc004eop.3; uc004eoq.3 | *AMMECR1* | Alport syndrome, mental retardation, midface hypoplasia and elliptocytosis chromosomal region gene 1 | 5.13 | 5.1 | 1.02 | 0.0281 |
| TC11001543.hg.1 | NM_001142315; NM_001142316; NM_005574; ENST00000257818; ENST00000395833; ENST00000493667; AF257211; BC034041; BC035607; BC042426; BC073973; OTTHUMT00000347776; OTTHUMT00000347777; OTTHUMT00000347778; OTTHUMT00000347779; OTTHUMT00000347780; OTTHUMT00000347781; uc001mvc.3; uc001mvd.3; uc001mve.3; uc010rel.2; uc010rem.2 | *LMO2* | LIM domain only 2 (rhombotin-like 1) | 5.99 | 5.93 | 1.04 | 0.0281 |
| TC11000413.hg.1 | NM_001005470; ENST00000309562; uc010rhs.2 | *OR4B1* | olfactory receptor, family 4, subfamily B, member 1 | 2.03 | 1.84 | 1.14 | 0.0282 |
| TC02001161.hg.1 | NM_001160033; NM_001160046; NM_152524; ENST00000357799; ENST00000409203; ENST00000469840; BC048349; BC092412; OTTHUMT00000335834; OTTHUMT00000335835; OTTHUMT00000335836; OTTHUMT00000335837; OTTHUMT00000335838; OTTHUMT00000335839; uc002uvv.4; uc002uvw.2; uc010zhd.1; uc010zhe.1 | *SGOL2* | shugoshin-like 2 (S. pombe) | 2.19 | 2.15 | 1.03 | 0.0282 |
| TC07000839.hg.1 | NM_178563; ENST00000435976; ENST00000436302; ENST00000494702; BC030651; OTTHUMT00000339931; OTTHUMT00000339932; OTTHUMT00000339933; OTTHUMT00000339934; OTTHUMT00000340213; OTTHUMT00000376655; OTTHUMT00000376656; uc011kpw.2 | *AGBL3* | ATP/GTP binding protein-like 3 | 2.27 | 2.24 | 1.02 | 0.0283 |
| TC06000115.hg.1 | NM_016255; ENST00000259963; BC047881; OTTHUMT00000039950; uc003ncc.3 | *FAM8A1* | family with sequence similarity 8, member A1 | 7.29 | 7.14 | 1.11 | 0.0283 |
| TC02000860.hg.1 | ENST00000431979 | *LINC01087* | long intergenic non-protein coding RNA 1087 | 1.24 | 1.15 | 1.06 | 0.0285 |
| TC11000436.hg.1 | NM_001001967; ENST00000361760; uc010ril.2 | *OR5D13* | olfactory receptor, family 5, subfamily D, member 13 (gene/pseudogene) | 1.78 | 1.7 | 1.05 | 0.0285 |
| TC05000753.hg.1 | NM_018930; ENST00000239446; BC031837; OTTHUMT00000251821; uc003lix.3 | *PCDHB10* | protocadherin beta 10 | 2.62 | 2.46 | 1.12 | 0.0285 |
| TC13000831.hg.1 | OTTHUMT00000045601 | *RP11-214F16.3* |  | 3.81 | 3.69 | 1.09 | 0.0285 |
| TC21000747.hg.1 | AL355711 | *ABCG1* | ATP binding cassette subfamily G member 1 | 6.03 | 5.64 | 1.31 | 0.0286 |
| TC05002534.hg.1 | AK093521 | *LOC100289230* | uncharacterized LOC100289230 | 4.62 | 4.52 | 1.07 | 0.0286 |
| TC17001553.hg.1 | NM_007294; NM_007297; NM_007298; NM_007299; NM_007300; NR_027676; ENST00000352993; ENST00000354071; ENST00000357654; ENST00000412061; ENST00000468300; ENST00000471181; ENST00000491747; ENST00000493795; AK293762; AK307553; AK308084; AK316200; AY354539; BC038947; BC046142; BC085615; BC106746; BC114511; BC114562; BC115037; DQ333386; DQ333387; DQ363751; OTTHUMT00000348798; OTTHUMT00000348799; OTTHUMT00000348800; OTTHUMT00000348801; OTTHUMT00000348802; OTTHUMT00000348803; OTTHUMT00000348804; OTTHUMT00000348805; OTTHUMT00000348806; OTTHUMT00000348807; OTTHUMT00000348808; OTTHUMT00000348809; OTTHUMT00000348810; OTTHUMT00000348811; OTTHUMT00000348812; OTTHUMT00000348813; OTTHUMT00000348814; OTTHUMT00000348815; OTTHUMT00000348816; OTTHUMT00000348817; OTTHUMT00000348818; OTTHUMT00000348819; uc002icp.4; uc002icq.3; uc002ict.3; uc002icu.3; uc002idc.1; uc002idd.3; uc002ide.1; uc010cyx.3; uc010cyy.1; uc010cyz.2; uc010cza.2; uc010whl.2; uc010whm.2; uc010whn.2; uc010whq.1; uc010whr.1; uc010whs.1; uc010wht.1 | *BRCA1* | breast cancer 1, early onset | 4.17 | 4.15 | 1.01 | 0.0287 |
| TC01001046.hg.1 | NR_027337; OTTHUMT00000099688; uc001eio.2 | *HIST2H2BA* | histone cluster 2, H2ba (pseudogene) | 5.86 | 5.68 | 1.13 | 0.0288 |
| TC17002436.hg.1 | NR_028075; NR_028076 | *SCARF1* | scavenger receptor class F, member 1 | 6.14 | 6.03 | 1.08 | 0.0288 |
| TC07001405.hg.1 | NM_207366; ENST00000388975; ENST00000477628; OTTHUMT00000251489; OTTHUMT00000343102; uc003tqz.2 | *Sep-14* | septin 14 | 2.07 | 2.01 | 1.04 | 0.0288 |
| TC11003507.hg.1 | NM_001007232; ENST00000375707; uc001pir.1 | *CARD17* | caspase recruitment domain family, member 17 | 4.84 | 4.55 | 1.22 | 0.0289 |
| TC0X000417.hg.1 | NM_016500; ENST00000373357; ENST00000373358; AY927616; BC001220; BC051894; OTTHUMT00000057294; OTTHUMT00000057295; uc004eck.1; uc004ecl.1 | *PBDC1* | polysaccharide biosynthesis domain containing 1 | 5.38 | 5.45 | -1.04 | 0.0289 |
| TC21000025.hg.1 | NM_144770; ENST00000400577; ENST00000468643; AF519623; AY077695; BC030196; OTTHUMT00000157818; OTTHUMT00000157819; OTTHUMT00000157820; OTTHUMT00000157821; OTTHUMT00000157822; OTTHUMT00000157823; uc002yjn.4; uc002yjo.4; uc002yjp.4 | *RBM11* | RNA binding motif protein 11 | 1.92 | 1.84 | 1.06 | 0.0289 |
| TC17001094.hg.1 | NM_000546; NM_001126112; NM_001126113; NM_001126114; NM_001126115; NM_001126116; NM_001126117; NM_001126118; ENST00000269305; ENST00000359597; ENST00000413465; ENST00000420246; ENST00000445888; ENST00000455263; AK303277; AM076971; AM076972; BC003596; DQ485152; DQ648883; DQ648884; DQ648885; DQ648887; OTTHUMT00000367397; OTTHUMT00000367398; OTTHUMT00000367399; OTTHUMT00000367400; OTTHUMT00000367401; OTTHUMT00000367402; OTTHUMT00000367403; OTTHUMT00000367404; OTTHUMT00000367405; OTTHUMT00000367409; OTTHUMT00000367410; OTTHUMT00000367411; uc002gig.1; uc002gih.3; uc010cne.1; uc010cnj.1 | *TP53* | tumor protein p53 | 6.91 | 7.02 | -1.08 | 0.0289 |
| TC09000952.hg.1 | NM_002171; ENST00000357374; BC069409; BC103972; OTTHUMT00000051887; uc003zoq.1 | *IFNA10* | interferon, alpha 10 | 1.86 | 1.75 | 1.08 | 0.029 |
| TC04001302.hg.1 | NM_006239; ENST00000286719; ENST00000510607; AF023457; BX648754; OTTHUMT00000362929; OTTHUMT00000362930; OTTHUMT00000362931; OTTHUMT00000362932; OTTHUMT00000362933; OTTHUMT00000362934; uc003hix.3; uc003hiy.3; uc003hiz.1 | *PPEF2* | protein phosphatase, EF-hand calcium binding domain 2 | 2.55 | 2.52 | 1.02 | 0.029 |
| TC09001374.hg.1 | NM_007001; ENST00000253270; ENST00000375257; ENST00000375259; ENST00000482643; AK307890; BC100278; BC113579; OTTHUMT00000053261; OTTHUMT00000053262; OTTHUMT00000053263; OTTHUMT00000053264; uc004awc.3; uc010msd.3; uc010msf.3 | *SLC35D2* | solute carrier family 35 (UDP-GlcNAc/UDP-glucose transporter), member D2 | 4.67 | 4.58 | 1.06 | 0.029 |
| TC02002510.hg.1 | NM_024622; ENST00000453153; ENST00000453929; ENST00000495505; AK055892; AK130797; BC017950; BC032687; OTTHUMT00000337788; OTTHUMT00000337789; OTTHUMT00000337790; OTTHUMT00000337791; OTTHUMT00000337792; OTTHUMT00000337793; OTTHUMT00000337794; OTTHUMT00000337795; OTTHUMT00000337796; OTTHUMT00000337797; uc002uev.4; uc002uew.4 | *FASTKD1* | FAST kinase domains 1 | 4.09 | 3.97 | 1.08 | 0.0291 |
| TC11003505.hg.1 | NM_001223; NM_033292; NM_033293; NM_033294; NM_033295; ENST00000353247; ENST00000436863; ENST00000446369; ENST00000525825; ENST00000526568; ENST00000527979; ENST00000528974; ENST00000531166; ENST00000533400; ENST00000534497; AK290114; AK290122; AK296646; AK301037; AK310059; BC062327; M87507; OTTHUMT00000109396; uc021qpt.1 | *CASP1* | caspase 1 | 7.07 | 6.82 | 1.18 | 0.0292 |
| TC0X000082.hg.1 | NM_001080975; NM_004726; ENST00000303843; ENST00000357277; ENST00000469714; AK298913; OTTHUMT00000055924; OTTHUMT00000055925; OTTHUMT00000055926; OTTHUMT00000055927; OTTHUMT00000316778; uc004cxv.1; uc004cxw.1; uc011miw.1 | *REPS2* | RALBP1 associated Eps domain containing 2 | 7.42 | 7.09 | 1.26 | 0.0292 |
| TC04002558.hg.1 | BC034324 | *LOC101928760* | uncharacterized LOC101928760 | 3.33 | 3.15 | 1.13 | 0.0293 |
| TC16000631.hg.1 | NM_016373; NM_130791; NM_130844; ENST00000355860; ENST00000402655; ENST00000406884; ENST00000408984; ENST00000539474; AF395124; AK298322; BC003184; uc002ffi.2; uc002ffj.2; uc002ffk.3; uc002ffl.3; uc010che.3; uc010vnk.2 | *WWOX* | WW domain containing oxidoreductase | 5.27 | 5.32 | -1.04 | 0.0293 |
| TC03001998.hg.1 | NM_001161560; NM_001161561; NM_001161562; NM_001161563; NM_001161564; NM_001161565; NM_001161566; NM_015028; NR_027767; ENST00000284483; ENST00000341852; ENST00000357327; ENST00000436636; ENST00000460047; ENST00000464785; ENST00000465393; ENST00000470834; ENST00000475336; ENST00000488470; AK026470; BC055427; BC150256; OTTHUMT00000352973; OTTHUMT00000352974; OTTHUMT00000352975; OTTHUMT00000352976; OTTHUMT00000352977; OTTHUMT00000352978; OTTHUMT00000352979; OTTHUMT00000352980; OTTHUMT00000352981; OTTHUMT00000352982; OTTHUMT00000352983; OTTHUMT00000352984; OTTHUMT00000353029; OTTHUMT00000353030; OTTHUMT00000353031; uc003fhg.2; uc003fhh.2; uc003fhi.2; uc003fhj.2; uc003fhk.2; uc003fhl.2; uc003fhm.2; uc003fhn.2; uc003fho.2; uc003fhp.3; uc003fhq.3 | *TNIK* | TRAF2 and NCK interacting kinase | 6.24 | 6.37 | -1.09 | 0.0294 |
| TC21000125.hg.1 | NM_138983; ENST00000382348; ENST00000498799; BC026989; BC033290; OTTHUMT00000139730; OTTHUMT00000139731; OTTHUMT00000139732; uc002yqz.3 | *OLIG1* | oligodendrocyte transcription factor 1 | 5.44 | 5.52 | -1.06 | 0.0295 |
| TC12000555.hg.1 | NM_001252078; NM_001252079; NM_006313; ENST00000280377; ENST00000312635; ENST00000353364; ENST00000550632; AK295947; AK297759; BC125123; uc001sra.3; uc001srb.2; uc001src.2; uc010ssj.2; uc010ssk.2 | *USP15* | ubiquitin specific peptidase 15 | 8.55 | 8.27 | 1.21 | 0.0295 |
| TC03002231.hg.1 | AK092130 | *LOC285378* | uncharacterized LOC285378 | 4.51 | 4.38 | 1.09 | 0.0297 |
| TC02004312.hg.1 | AK123766 | *LOC644838* | uncharacterized LOC644838 | 2.47 | 2.41 | 1.05 | 0.0297 |
| TC01003662.hg.1 | NM_001206846; NM_018136; ENST00000294732; ENST00000367408; ENST00000367409; AY971957; OTTHUMT00000088256; OTTHUMT00000088257; uc001gtu.3; uc001gtv.3; uc001gtw.4 | *ASPM* | abnormal spindle microtubule assembly | 2.84 | 2.49 | 1.27 | 0.0298 |
| TC12000987.hg.1 | NR_037470; uc021rfx.1 | *MIR3908* | microRNA 3908 | 1.14 | 0.99 | 1.11 | 0.0298 |
| TC06001918.hg.1 | ENST00000455071; AK024998 | *LOC101928820* | uncharacterized LOC101928820 | 2.49 | 2.37 | 1.08 | 0.0299 |
| TC10001535.hg.1 | NM_013451; NM_133337; ENST00000358334; ENST00000359263; ENST00000371488; ENST00000371489; AL096713; BC033616; BC040110; BC052617; OTTHUMT00000049419; OTTHUMT00000049420; OTTHUMT00000049421; OTTHUMT00000049422; OTTHUMT00000049423; OTTHUMT00000049424; OTTHUMT00000049425; OTTHUMT00000049426; OTTHUMT00000049427; uc001kin.3; uc001kio.3; uc001kip.4; uc009xue.3; uc009xuf.2 | *MYOF* | myoferlin | 4.73 | 5.1 | -1.3 | 0.0299 |
| TC19002594.hg.1 | AF113692 | *ZNF460* | zinc finger protein 460 | 2.63 | 2.82 | -1.14 | 0.0299 |
| TC22000387.hg.1 | NR_027240; uc011aqu.1 | *LOC730668* | dynein heavy chain -like pseudogene | 3.86 | 3.96 | -1.08 | 0.03 |
| TC14002324.hg.1 | NM_022571; BC032831; OTTHUMT00000276940; uc001xed.2; uc010apj.3 | *GPR135* | G protein-coupled receptor 135 | 4.61 | 4.54 | 1.05 | 0.0301 |
| TC01005172.hg.1 | X64978 | *OR2L2* | olfactory receptor, family 2, subfamily L, member 2 | 3.6 | 3.18 | 1.34 | 0.0301 |
| TC09002275.hg.1 | NR_024425 | *PTGES2-AS1* | PTGES2 antisense RNA 1 (head to head) | 5.83 | 5.98 | -1.11 | 0.0301 |
| TC19000133.hg.1 | NM_001193374; NM_020415; ENST00000221515; ENST00000381324; AB111910; BC069302; BC101554; BC101560; uc002mhf.1; uc002mhg.1; uc010dvm.1 | *RETN* | resistin | 5.57 | 5.66 | -1.06 | 0.0301 |
| TC06001077.hg.1 | NM_005715; ENST00000367463; ENST00000466695; BC093668; BC093694; OTTHUMT00000043363; OTTHUMT00000043364; OTTHUMT00000043365; uc003qmg.3 | *UST* | uronyl-2-sulfotransferase | 4.42 | 4.54 | -1.09 | 0.0301 |
| TC21000436.hg.1 | NM_000411; NM_001242784; NM_001242785; ENST00000336648; ENST00000399120; ENST00000482273; AK307940; BC060787; OTTHUMT00000194686; OTTHUMT00000194687; OTTHUMT00000194688; OTTHUMT00000194689; OTTHUMT00000194690; OTTHUMT00000194691; uc002yvs.3; uc010gnb.3; uc010gnc.2; uc021wjb.1 | *HLCS* | holocarboxylase synthetase (biotin-(proprionyl-CoA-carboxylase (ATP-hydrolysing)) ligase) | 4.9 | 4.99 | -1.06 | 0.0302 |
| TC0X000643.hg.1 | ENST00000441841 | *LOC340581* | uncharacterized LOC340581 | 2.51 | 2.39 | 1.09 | 0.0303 |
| TC04000145.hg.1 | NM_001775; ENST00000226279; AK297592; BC007964; OTTHUMT00000250322; OTTHUMT00000359588; OTTHUMT00000359589; OTTHUMT00000359590; OTTHUMT00000359591; uc003gol.1; uc021xmk.1 | *CD38* | CD38 molecule | 6.06 | 6.39 | -1.26 | 0.0304 |
| TC02000978.hg.1 | NM_012198; ENST00000233612; ENST00000429691; ENST00000437150; ENST00000473240; BC005214; OTTHUMT00000255080; OTTHUMT00000332986; OTTHUMT00000332989; OTTHUMT00000332990; OTTHUMT00000332991; OTTHUMT00000332993; OTTHUMT00000332994; OTTHUMT00000332995; OTTHUMT00000332996; OTTHUMT00000332997; uc002ucg.3 | *GCA* | grancalcin, EF-hand calcium binding protein | 7.63 | 7.39 | 1.19 | 0.0304 |
| TC06002427.hg.1 | NR_040662 | *HCP5* | HLA complex P5 (non-protein coding) | 1.82 | 1.72 | 1.07 | 0.0304 |
| TC05000982.hg.1 | NM_022304; ENST00000231683; uc003mdd.2 | *HRH2* | histamine receptor H2 | 4.82 | 4.6 | 1.16 | 0.0304 |
| TC05003405.hg.1 | NM_006930; NM_170679; ENST00000353411; ENST00000517625; ENST00000521216; ENST00000522552; ENST00000522855; BC009839; BC020798; BC025673; BC065730; OTTHUMT00000251162; OTTHUMT00000251163; OTTHUMT00000381580; OTTHUMT00000381581; OTTHUMT00000381582; OTTHUMT00000381583; OTTHUMT00000381584; OTTHUMT00000381585; OTTHUMT00000381586; OTTHUMT00000381587; OTTHUMT00000381588; OTTHUMT00000381589; OTTHUMT00000381590; uc003kzc.4; uc003kzd.4 | *SKP1* | S-phase kinase-associated protein 1 | 5.82 | 5.63 | 1.14 | 0.0304 |
| TC17001982.hg.1 | NM_001033046; NM_001100407; NM_001100408; NM_001193653; NM_001193654; NM_001193655; NM_001193657; NR_036514; NR_036516; NR_036517; NR_036518; NR_036519; ENST00000306645; ENST00000336995; ENST00000342572; ENST00000434650; ENST00000437807; ENST00000536759; AF269290; AK090484; AK309201; BC003595; uc002kex.3; uc002key.3; uc002kfa.3; uc002kfb.4; uc002kfc.4; uc002kfd.4; uc002kfe.4; uc010dir.3; uc010dis.2; uc021ufq.1; uc021ufr.1; uc021ufs.1; uc021uft.1; uc021ufu.1; uc021ufv.1 | *C17orf62* | chromosome 17 open reading frame 62 | 7.71 | 7.59 | 1.08 | 0.0305 |
| TC19002141.hg.1 | AK123333 | *LINC01480* | long intergenic non-protein coding RNA 1480 | 3.43 | 3.29 | 1.1 | 0.0305 |
| TC02001769.hg.1 | NM_001112800; NM_001112801; NM_001112802; NM_001252624; NM_021097; ENST00000332839; ENST00000402441; ENST00000403092; ENST00000405269; ENST00000405901; ENST00000406391; ENST00000406785; ENST00000408028; BX648299; OTTHUMT00000325968; OTTHUMT00000325969; OTTHUMT00000326025; OTTHUMT00000326026; OTTHUMT00000326028; OTTHUMT00000326029; OTTHUMT00000326065; OTTHUMT00000326066; OTTHUMT00000326067; uc002rrx.3; uc002rry.3; uc002rrz.3; uc002rsa.3; uc002rsb.2; uc002rsc.1; uc002rsd.4; uc010fan.1; Y12878; Y13032 | *SLC8A1* | solute carrier family 8 (sodium/calcium exchanger), member 1 | 5.37 | 5.28 | 1.07 | 0.0305 |
| TC01002211.hg.1 | NM_004753; ENST00000482265; AK225057; AK310202; AY358093; BC002730; DQ426870; OTTHUMT00000005318; OTTHUMT00000005319; OTTHUMT00000005320; OTTHUMT00000005321; uc001aub.3; uc001auc.3; uc001aud.4; uc001aue.1; uc009vnm.3 | *DHRS3* | dehydrogenase/reductase (SDR family) member 3 | 5.77 | 5.9 | -1.1 | 0.0306 |
| TC08000524.hg.1 | ENST00000522365; BC038578 | *LINC01419* | long intergenic non-protein coding RNA 1419 | 3.96 | 3.87 | 1.06 | 0.0307 |
| TC01000966.hg.1 | NM_032414; ENST00000271331; BC025399; OTTHUMT00000031969; uc001dzs.3 | *PROK1* | prokineticin 1 | 4.96 | 5.01 | -1.03 | 0.0307 |
| TC19000534.hg.1 | NM_001243116; NM_002503; NR_040515; ENST00000313582; ENST00000392079; ENST00000509705; AK290569; BC015528; uc002ojw.3; uc002ojx.3; uc002ojy.3; uc010egk.2 | *NFKBIB* | nuclear factor of kappa light polypeptide gene enhancer in B-cells inhibitor, beta | 5.78 | 5.84 | -1.04 | 0.0308 |
| TC12000855.hg.1 | NM_001143779; NM_014055; NM_031473; ENST00000242591; ENST00000361948; ENST00000549009; ENST00000550748; ENST00000552912; BC004536; BC108257; uc001tqg.3; uc001tqh.3; uc001tqi.3 | *IFT81* | intraflagellar transport 81 | 2.41 | 2.39 | 1.02 | 0.0309 |
| TC19000228.hg.1 | NM_002229; ENST00000302754; BC004250; BC009465; BC009466; uc002mvc.3 | *JUNB* | jun B proto-oncogene | 7.53 | 7.36 | 1.13 | 0.0309 |
| TC09002414.hg.1 | DQ884942; DQ884943 | *BNC2* | basonuclin 2 | 2.13 | 2.31 | -1.14 | 0.031 |
| TC06001246.hg.1 | NM_000129; ENST00000264870; AK304335; BC027963; OTTHUMT00000039756; OTTHUMT00000039757; OTTHUMT00000317456; OTTHUMT00000317457; OTTHUMT00000317458; OTTHUMT00000317459; uc003mwv.3; uc011dib.2 | *F13A1* | coagulation factor XIII, A1 polypeptide | 8.62 | 8.36 | 1.2 | 0.0311 |
| TC09000330.hg.1 | NM_001242505; NM_001242506; NM_001242507; NM_004293; ENST00000238018; ENST00000358399; ENST00000376986; ENST00000477618; ENST00000545168; AF019638; AK097540; BC012859; BC053584; OTTHUMT00000052633; OTTHUMT00000052634; OTTHUMT00000052635; OTTHUMT00000052636; OTTHUMT00000052637; OTTHUMT00000052638; OTTHUMT00000052639; uc004aiq.3; uc004air.3; uc004ais.3; uc004ait.1; uc010mow.2; uc011lse.2; uc011lsf.2 | *GDA* | guanine deaminase | 2.56 | 2.55 | 1.01 | 0.0311 |
| TC01000741.hg.1 | NM_001559; ENST00000262345; ENST00000371000; ENST00000465396; ENST00000541374; ENST00000544434; AK294494; AK316525; BC104772; BC143248; BC143249; BC143250; BC143252; OTTHUMT00000025202; OTTHUMT00000095782; OTTHUMT00000095783; uc001ddu.3; uc010oqi.2; uc010oqj.2 | *IL12RB2* | interleukin 12 receptor, beta 2 | 4.37 | 4.62 | -1.19 | 0.0311 |
| TC08000440.hg.1 | NR_039979; ENST00000517689; ENST00000518035 | *LINC00967* | long intergenic non-protein coding RNA 967 | 3.25 | 3.2 | 1.04 | 0.0311 |
| TC0X000013.hg.1 | NR_033380; NR_033381; ENST00000414513; uc004cqi.2; uc004cqj.2 | *CD99P1* | CD99 molecule pseudogene 1 | 5.73 | 5.66 | 1.05 | 0.0312 |
| TC10000427.hg.1 | ENST00000437376 | *CEP57L1P1* | centrosomal protein 57kDa-like 1 pseudogene 1 | 2.61 | 2.36 | 1.19 | 0.0312 |
| TC03003225.hg.1 | NR_028135 | *KCNMB3* | potassium channel subfamily M regulatory beta subunit 3 | 4.28 | 4.42 | -1.1 | 0.0312 |
| TC22001133.hg.1 | BC039353 | *LOC100506679* | uncharacterized LOC100506679 | 3.29 | 3.4 | -1.08 | 0.0312 |
| TC01004095.hg.1 | NM_030645; ENST00000366472; ENST00000475978; AK125837; AK299713; BC017254; OTTHUMT00000097140; OTTHUMT00000097141; OTTHUMT00000097142; OTTHUMT00000097143; uc001iev.1; uc001iew.1; uc010pzp.1 | *SH3BP5L* | SH3-binding domain protein 5-like | 5.65 | 5.57 | 1.05 | 0.0312 |
| TC11002410.hg.1 | NM_001199922; NM_170601; ENST00000263593; ENST00000525730; ENST00000545756; BC068450; uc001qan.3; uc021qru.1 | *SIAE* | sialic acid acetylesterase | 5.03 | 4.94 | 1.07 | 0.0312 |
| TC07001003.hg.1 | NM_153236; ENST00000313543; BC027613; OTTHUMT00000349277; uc003whk.3 | *GIMAP7* | GTPase, IMAP family member 7 | 7.84 | 7.95 | -1.08 | 0.0313 |
| TC0X000366.hg.1 | ENST00000364816 | *RNA5SP506* | RNA, 5S ribosomal pseudogene 506 | 7.59 | 7.4 | 1.14 | 0.0313 |
| TC04001205.hg.1 | ENST00000508484; BC044946 | *LOC339978* | uncharacterized LOC339978 | 2.96 | 2.77 | 1.14 | 0.0314 |
| TC22000776.hg.1 | ENST00000456099; AK097791 | *LOC101927051* | uncharacterized LOC101927051 | 5.06 | 5.14 | -1.06 | 0.0314 |
| TC12002005.hg.1 | NR_030351; ENST00000385232; uc021reh.1 | *MIR620* | microRNA 620 | 0.91 | 0.86 | 1.04 | 0.0314 |
| TC13001497.hg.1 | NR_023351 | *SPRYD7* | SPRY domain containing 7 | 3.89 | 4.06 | -1.12 | 0.0314 |
| TC0Y000141.hg.1 | NR_001546; ENST00000434487; OTTHUMT00000099510; uc004frm.3 | *TTTY20* | testis-specific transcript, Y-linked 20 (non-protein coding) | 2.05 | 1.92 | 1.09 | 0.0315 |
| TC09000928.hg.1 | NM_017637; ENST00000380666; ENST00000380667; ENST00000380672; ENST00000471301; ENST00000545497; AK299073; AK304745; BC037160; CR933649; DQ884933; DQ884934; DQ884935; DQ884936; DQ884937; DQ884938; DQ884939; DQ884940; DQ884941; DQ884942; DQ884943; DQ884944; DQ884945; DQ884948; OTTHUMT00000051781; OTTHUMT00000051782; OTTHUMT00000051783; OTTHUMT00000051786; OTTHUMT00000207395; OTTHUMT00000216901; OTTHUMT00000216902; OTTHUMT00000218575; uc003zmi.3; uc003zmj.3; uc003zml.3; uc003zmm.3; uc003zmn.1; uc003zmo.1; uc003zmp.1; uc003zmq.1; uc003zmr.1; uc003zms.1; uc003zmt.1; uc003zmu.1; uc010mij.1; uc010mik.1; uc010mim.1; uc011lmv.2; uc011lmw.2 | *BNC2* | basonuclin 2 | 3.26 | 3.34 | -1.05 | 0.0316 |
| TC07001159.hg.1 | NM_001101417; NM_001101426; ENST00000399310; ENST00000407010; ENST00000479493; OTTHUMT00000326252; OTTHUMT00000326298; OTTHUMT00000326299; uc010ktx.2; uc010kty.2 | *ISPD* | isoprenoid synthase domain containing | 2.45 | 2.35 | 1.07 | 0.0316 |
| TC15000284.hg.1 | NM_001159508; NM_002225; ENST00000479013; ENST00000487418; AF070531; BC017202; OTTHUMT00000252250; OTTHUMT00000352698; OTTHUMT00000352699; OTTHUMT00000352700; OTTHUMT00000352701; OTTHUMT00000352702; OTTHUMT00000352703; OTTHUMT00000352704; OTTHUMT00000352705; OTTHUMT00000352706; uc001zlq.2; uc001zlr.2; uc001zls.3 | *IVD* | isovaleryl-CoA dehydrogenase | 5.21 | 5.26 | -1.04 | 0.0316 |
| TC19_gl000209_random000004.hg.1 | NM_001242867; AF208687; uc002qup.1 | *KIR3DL2* | killer cell immunoglobulin-like receptor, three domains, long cytoplasmic tail, 2 | 4.59 | 5.16 | -1.49 | 0.0316 |
| TC22001340.hg.1 | AK097791 | *LOC101927051* | uncharacterized LOC101927051 | 5 | 5.11 | -1.08 | 0.0316 |
| TC14001135.hg.1 | ENST00000556834 | *LOC102723604* | uncharacterized LOC102723604 | 2.84 | 2.98 | -1.1 | 0.0316 |
| TC16000165.hg.1 | NM_003498; ENST00000329565; BC036100; BC036443; OTTHUMT00000207059; uc002dbf.3 | *SNN* | stannin | 7.74 | 7.55 | 1.14 | 0.0317 |
| TC10001140.hg.1 | NR_033805; BC043365; uc001iuc.3; uc021pon.1 | *WAC-AS1* | WAC antisense RNA 1 (head to head) | 5.75 | 5.66 | 1.06 | 0.0317 |
| TC07001842.hg.1 | NM_022143; ENST00000249363; BC111561; OTTHUMT00000349170; OTTHUMT00000349171; OTTHUMT00000349172; OTTHUMT00000349173; uc003vmk.3 | *LRRC4* | leucine rich repeat containing 4 | 6.39 | 6.21 | 1.13 | 0.0318 |
| TC12001752.hg.1 | NM_004537; NM_139207; ENST00000261182; ENST00000393263; ENST00000431879; ENST00000535020; ENST00000542344; ENST00000544816; ENST00000547773; ENST00000547993; ENST00000548044; ENST00000549596; ENST00000552342; AK122670; AK295147; AK297500; AK304787; BC002387; uc001sxw.2; uc001sxx.2; uc001sxz.2; uc010sty.1; uc010stz.1; uc010sua.1 | *NAP1L1* | nucleosome assembly protein 1-like 1 | 6.39 | 6.26 | 1.1 | 0.0318 |
| TC0Y000080.hg.1 | NR_001526; NR_002179; NR_002180; ENST00000416110; OTTHUMT00000102242; uc004fvi.3 | *TTTY17A; TTTY17C; TTTY17B* | testis-specific transcript, Y-linked 17A (non-protein coding); testis-specific transcript, Y-linked 17C (non-protein coding); testis-specific transcript, Y-linked 17B (non-protein coding) | 1.54 | 1.46 | 1.06 | 0.0318 |
| TC0Y000090.hg.1 | NR_002180; ENST00000441906; NR_001526; NR_002179; uc004fwg.3 | *TTTY17B; TTTY17C; TTTY17A* | testis-specific transcript, Y-linked 17B (non-protein coding); testis-specific transcript, Y-linked 17C (non-protein coding); testis-specific transcript, Y-linked 17A (non-protein coding) | 1.54 | 1.46 | 1.06 | 0.0318 |
| TC0Y000213.hg.1 | NR_002180; ENST00000421387; NR_001526; NR_002179; uc004fwt.3 | *TTTY17B; TTTY17C; TTTY17A* | testis-specific transcript, Y-linked 17B (non-protein coding); testis-specific transcript, Y-linked 17C (non-protein coding); testis-specific transcript, Y-linked 17A (non-protein coding) | 1.54 | 1.46 | 1.06 | 0.0318 |
| TC10002856.hg.1 | BC017894 | *CACUL1* | CDK2-associated, cullin domain 1 | 7.16 | 6.91 | 1.19 | 0.0319 |
| TC22000829.hg.1 | NM_015704; ENST00000263256; ENST00000463886; BC093956; BC171724; OTTHUMT00000104124; OTTHUMT00000321672; OTTHUMT00000321902; OTTHUMT00000321903; uc003bam.2; uc011apb.2 | *DESI1* | desumoylating isopeptidase 1 | 6.03 | 5.94 | 1.06 | 0.0319 |
| TC03000081.hg.1 | NR_036481; OTTHUMT00000359661; uc021wtj.1 | *FGD5P1* | FYVE, RhoGEF and PH domain containing 5 pseudogene 1 | 4.54 | 4.7 | -1.11 | 0.0319 |
| TC17000091.hg.1 | NM_198154; ENST00000330767; ENST00000389982; BC040900; BC107110; OTTHUMT00000256403; OTTHUMT00000256407; uc002ggf.1; uc002ggg.1; uc002ggh.1 | *TMEM95* | transmembrane protein 95 | 3.86 | 3.98 | -1.08 | 0.0319 |
| TC14001339.hg.1 | NM_174976; ENST00000319374; BC117676; uc010asp.3 | *ZDHHC22* | zinc finger, DHHC-type containing 22 | 4.21 | 4.27 | -1.04 | 0.0319 |
| TC16000493.hg.1 | NM_001145770; NM_001145771; NM_001145772; NM_001145773; NM_001145774; NM_005682; NM_201524; NM_201525; ENST00000388813; ENST00000456916; ENST00000540164; AK131550; AK297236; BC008770; CR936747 | *ADGRG1* | adhesion G protein-coupled receptor G1 | 6.84 | 7.19 | -1.27 | 0.032 |
| TC02002577.hg.1 | NM_178123; ENST00000428443; ENST00000486468; AL122046; BC047578; OTTHUMT00000335916; OTTHUMT00000335917; OTTHUMT00000335918; OTTHUMT00000335919; OTTHUMT00000335920; OTTHUMT00000335921; OTTHUMT00000335922; OTTHUMT00000335923; OTTHUMT00000335924; uc002unh.4; uc002uni.4 | *SESTD1* | SEC14 and spectrin domains 1 | 5.17 | 5.06 | 1.08 | 0.032 |
| TC19001776.hg.1 | NM_005601; ENST00000221978; BC015759; uc002pwj.3 | *NKG7* | natural killer cell granule protein 7 | 8.06 | 8.5 | -1.36 | 0.0321 |
| TC17001032.hg.1 | NM_000080; ENST00000293780; OTTHUMT00000207560; uc002fzk.1 | *CHRNE* | cholinergic receptor, nicotinic epsilon | 4.78 | 4.92 | -1.1 | 0.0322 |
| TC06002735.hg.1 | NR_044997 | *HCG25* | HLA complex group 25 (non-protein coding) | 5.12 | 5.23 | -1.08 | 0.0322 |
| TC02002787.hg.1 | NR_036081; uc021vxa.1 | *MIR3131* | microRNA 3131 | 5.03 | 4.88 | 1.11 | 0.0322 |
| TC03001450.hg.1 | NR_029660; uc011bee.2 | *MIRLET7G* | microRNA let-7g | 4.3 | 4.03 | 1.21 | 0.0322 |
| TC13000064.hg.1 | NM_001008564; NM_014089; ENST00000381718; ENST00000381736; ENST00000463407; ENST00000466694; BC001104; OTTHUMT00000044228; OTTHUMT00000044229; OTTHUMT00000044230; OTTHUMT00000044231; OTTHUMT00000044232; OTTHUMT00000044233; OTTHUMT00000354750; OTTHUMT00000354752; OTTHUMT00000354753; OTTHUMT00000354754; OTTHUMT00000354755; OTTHUMT00000354756; OTTHUMT00000354757; OTTHUMT00000354758 | *NUP58* | nucleoporin 58kDa | 6.39 | 6.29 | 1.07 | 0.0322 |
| TC19000158.hg.1 | NM_001079935; ENST00000456448; BC146920; uc002mlb.1 | *OR7E24* | olfactory receptor, family 7, subfamily E, member 24 | 2.18 | 2.3 | -1.09 | 0.0322 |
| TC09000989.hg.1 | AK172755 | *MOB3B* | MOB kinase activator 3B | 2.52 | 2.35 | 1.13 | 0.0323 |
| TC11000807.hg.1 | NR_000005; ENST00000384214; uc001owj.1 | *SNORD15A* | small nucleolar RNA, C/D box 15A | 5.48 | 4.95 | 1.45 | 0.0323 |
| TC21000345.hg.1 | NM_006988; ENST00000284984; BC036515; OTTHUMT00000171650; OTTHUMT00000171651; OTTHUMT00000171652; OTTHUMT00000171653; OTTHUMT00000381380; OTTHUMT00000381381; uc002ymf.3 | *ADAMTS1* | ADAM metallopeptidase with thrombospondin type 1 motif 1 | 4.05 | 4.19 | -1.11 | 0.0324 |
| TC02001466.hg.1 | NM_001033575; ENST00000343217; ENST00000405954; ENST00000497221; BC036198; OTTHUMT00000325814; OTTHUMT00000325815; OTTHUMT00000325816; OTTHUMT00000325817; uc002vzg.3; uc002vzh.3 | *DUSP28* | dual specificity phosphatase 28 | 4.92 | 4.86 | 1.05 | 0.0324 |
| TC05001604.hg.1 | NM_001002796; NM_024717; ENST00000312216; ENST00000429576; ENST00000505078; ENST00000505208; ENST00000514040; ENST00000515393; AK057694; AK058012; BC030005; OTTHUMT00000370280; OTTHUMT00000370281; OTTHUMT00000370282; OTTHUMT00000370283; OTTHUMT00000370284; OTTHUMT00000370285; OTTHUMT00000370286; OTTHUMT00000370287; OTTHUMT00000370288; OTTHUMT00000370289; OTTHUMT00000370413; OTTHUMT00000370414; OTTHUMT00000370415; OTTHUMT00000370416; OTTHUMT00000370417; OTTHUMT00000370418; OTTHUMT00000370419; OTTHUMT00000377208; uc003kkv.2; uc003kkw.2; uc003kkx.2; uc003kkz.2 | *MCTP1* | multiple C2 domains, transmembrane 1 | 5.68 | 5.49 | 1.14 | 0.0325 |
| TC16002039.hg.1 | NM_001014449; NM_018332; ENST00000302243; ENST00000417604; ENST00000443119; AK125701; AK299504; AK299539; AK310604; AK310804; BC005162; BC006544; BC137496; BC137497; CR749227; OTTHUMT00000268967; uc002eyv.3; uc010cfq.1; uc010cfr.3; uc010cfs.3; uc010vlz.2 | *DDX19B; DDX19A* | DEAD (Asp-Glu-Ala-Asp) box polypeptide 19B; DEAD (Asp-Glu-Ala-Asp) box polypeptide 19A | 5.21 | 5.27 | -1.04 | 0.0326 |
| TC0X002326.hg.1 | NM_004469; ENST00000297904; ENST00000488351; BC027948; OTTHUMT00000055859; OTTHUMT00000055860; uc004cwt.2 | *FIGF* | c-fos induced growth factor (vascular endothelial growth factor D) | 1.9 | 1.96 | -1.04 | 0.0326 |
| TC03002038.hg.1 | NM_021629; ENST00000232564; ENST00000465153; ENST00000468623; AK310009; BC000873; OTTHUMT00000258218; OTTHUMT00000348858; OTTHUMT00000348859; OTTHUMT00000348860; OTTHUMT00000348861; uc003fju.4; uc003fjv.4 | *GNB4* | guanine nucleotide binding protein (G protein), beta polypeptide 4 | 6.32 | 6.18 | 1.1 | 0.0326 |
| TC18000500.hg.1 | NM_001190821; NM_001190822; NM_001190823; NM_005904; ENST00000262158; ENST00000545051; BC074818; OTTHUMT00000255906; uc002ldf.3; uc002ldg.3; uc010xde.2; uc021ujr.1 | *SMAD7* | SMAD family member 7 | 4.96 | 5.03 | -1.05 | 0.0326 |
| TC07001297.hg.1 | OTTHUMT00000338399 | *TRGV8* | T cell receptor gamma variable 8 | 5.71 | 5.96 | -1.19 | 0.0327 |
| TC13000518.hg.1 | NM_001135919; NM_181785; ENST00000266943; ENST00000380814; ENST00000475385; AK124604; BC036662; BC060850; BC068556; OTTHUMT00000044324; OTTHUMT00000044325; OTTHUMT00000276111; uc001usg.3; uc001ush.3; uc001usj.3 | *SLC46A3* | solute carrier family 46, member 3 | 5.89 | 5.76 | 1.1 | 0.0328 |
| TC06001065.hg.1 | NM_006834; ENST00000367495; BC015061; OTTHUMT00000042579; uc003qln.1 | *RAB32* | RAB32, member RAS oncogene family | 5.21 | 5.11 | 1.08 | 0.0329 |
| TC10001166.hg.1 | NM_001024628; NM_001024629; NM_001244972; NM_001244973; NM_003873; NR_045259; ENST00000265371; ENST00000374816; ENST00000374821; ENST00000374822; ENST00000374823; ENST00000374867; ENST00000374875; ENST00000395995; ENST00000466932; AF280547; AY249243; BC007533; BC007737; BX648025; OTTHUMT00000051203; OTTHUMT00000051204; OTTHUMT00000051205; OTTHUMT00000051206; OTTHUMT00000051207; OTTHUMT00000051208; OTTHUMT00000051209; OTTHUMT00000051210; OTTHUMT00000051211; OTTHUMT00000051212; OTTHUMT00000051213; uc001iwv.4; uc001iww.4; uc001iwx.4; uc001iwy.4; uc001iwz.2; uc001ixa.2; uc001ixb.2; uc001ixc.1; uc009xlz.3 | *NRP1* | neuropilin 1 | 3.86 | 3.77 | 1.07 | 0.033 |
| TC01001372.hg.1 | NM_021181; ENST00000359331; ENST00000368042; ENST00000368043; ENST00000441662; ENST00000444090; ENST00000458104; ENST00000458602; ENST00000484221; AK298499; AK298548; AK301137; AK301432; AK301438; BC027867; OTTHUMT00000060464; OTTHUMT00000060465; OTTHUMT00000060466; OTTHUMT00000060467; OTTHUMT00000060468; OTTHUMT00000060470; uc001fwq.3; uc001fwr.3; uc001fws.3; uc010pjn.2; uc010pjo.2; uc010pjp.2; uc010pjq.2; uc010pjr.2 | *SLAMF7* | SLAM family member 7 | 5.94 | 6.25 | -1.24 | 0.0331 |
| TC01005428.hg.1 | M61743 | *HIVEP3* | human immunodeficiency virus type I enhancer binding protein 3 | 5.35 | 5.49 | -1.11 | 0.0332 |
| TC08002512.hg.1 | BC033393 | *LINC00964* | long intergenic non-protein coding RNA 964 | 1.71 | 1.58 | 1.09 | 0.0332 |
| TC01000157.hg.1 | NR_003022; NR_003025; ENST00000459326; uc001atz.1 | *SNORA59B; SNORA59A* | small nucleolar RNA, H/ACA box 59B; small nucleolar RNA, H/ACA box 59A | 6.2 | 5.68 | 1.44 | 0.0332 |
| TC17000242.hg.1 | NR_003022; NR_003025; ENST00000458926; uc002gvz.1 | *SNORA59B; SNORA59A* | small nucleolar RNA, H/ACA box 59B; small nucleolar RNA, H/ACA box 59A | 6.2 | 5.68 | 1.44 | 0.0332 |
| TC11000442.hg.1 | NM_032681; ENST00000244891; ENST00000449290; BC005014; OTTHUMT00000344190; uc010rip.2; uc010riq.2 | *TRIM51* | tripartite motif-containing 51 | 3.76 | 3.58 | 1.13 | 0.0332 |
| TC01002612.hg.1 | NM_001114938; NM_001190182; ENST00000421127; ENST00000445048; ENST00000464739; ENST00000528266; AK057646; BC029888; BC128177; BC128178; OTTHUMT00000021511; OTTHUMT00000021512; OTTHUMT00000021513; OTTHUMT00000021514; OTTHUMT00000090853; uc001com.4; uc001con.4; uc009vxz.3; uc010ols.2; uc010olt.2 | *CCDC17* | coiled-coil domain containing 17 | 5.26 | 5.22 | 1.03 | 0.0333 |
| TC01004146.hg.1 | NR_036637 | *FAM213B* | family with sequence similarity 213, member B | 5.65 | 5.75 | -1.07 | 0.0333 |
| TC15002464.hg.1 | AK094982 | *LOC283713* | uncharacterized LOC283713 | 2.49 | 2.67 | -1.13 | 0.0333 |
| TC22000316.hg.1 | NM_019008; ENST00000325301; ENST00000402881; ENST00000404569; ENST00000478342; AK290954; AL136768; AL834205; BC008327; OTTHUMT00000321325; OTTHUMT00000321326; OTTHUMT00000321327; OTTHUMT00000321328; OTTHUMT00000321329; OTTHUMT00000321331; OTTHUMT00000321332; OTTHUMT00000321333; OTTHUMT00000321334; OTTHUMT00000321335; OTTHUMT00000321336; OTTHUMT00000321337; OTTHUMT00000321338; OTTHUMT00000321339 | *MIEF1* | mitochondrial elongation factor 1 | 4.89 | 4.96 | -1.05 | 0.0333 |
| TC20000268.hg.1 | NM_006097; NM_181526; ENST00000279022; ENST00000346786; BC002648; OTTHUMT00000079015; OTTHUMT00000079016 | *MYL9* | myosin light chain 9 | 8.1 | 7.81 | 1.22 | 0.0333 |
| TC01002864.hg.1 | NM_001195683; NM_001195684; NM_003243; NR_036634; ENST00000212355; ENST00000370399; ENST00000465892; ENST00000468996; ENST00000525962; BC099914; BC126116; OTTHUMT00000027926; OTTHUMT00000027973; OTTHUMT00000027974; OTTHUMT00000027975; OTTHUMT00000027976; uc001doh.3; uc001doi.3; uc001doj.3; uc009wde.3; uc010osy.2 | *TGFBR3* | transforming growth factor beta receptor III | 6.04 | 6.32 | -1.22 | 0.0333 |
| TC01001651.hg.1 | NM_203459; ENST00000236925; ENST00000358823; ENST00000413307; BC125229; BC125230; OTTHUMT00000086956; OTTHUMT00000087034; OTTHUMT00000087035; OTTHUMT00000098716; OTTHUMT00000098717; uc001gvk.3; uc001gvl.3; uc001gvm.3 | *CAMSAP2* | calmodulin regulated spectrin-associated protein family, member 2 | 3.54 | 3.41 | 1.1 | 0.0334 |
| TC17002381.hg.1 | BC038218 | *LINC00868* | long intergenic non-protein coding RNA 868 | 4.8 | 4.93 | -1.09 | 0.0334 |
| TC17001429.hg.1 | ENST00000363245 | *RNA5SP440* | RNA, 5S ribosomal pseudogene 440 | 7.34 | 7.12 | 1.16 | 0.0334 |
| TC01006328.hg.1 | NM_024575; ENST00000368910; BC063014; OTTHUMT00000034069; uc001ewx.2 | *TNFAIP8L2* | tumor necrosis factor, alpha-induced protein 8-like 2 | 5.83 | 5.71 | 1.09 | 0.0334 |
| TC05003302.hg.1 | M25786 | *CSF1R* | colony stimulating factor 1 receptor | 6.23 | 6.38 | -1.11 | 0.0336 |
| TC19001744.hg.1 | NM_004977; ENST00000376959; ENST00000474951; ENST00000477616; AB208930; OTTHUMT00000104026; OTTHUMT00000314287; OTTHUMT00000314288; uc002prt.1; uc002pru.1 | *KCNC3* | potassium channel, voltage gated Shaw related subfamily C, member 3 | 5.37 | 5.45 | -1.06 | 0.0336 |
| TC03000880.hg.1 | NM_139245; ENST00000295839; ENST00000464260; ENST00000480117; ENST00000497343; ENST00000498165; AK055115; AK055323; BC090872; BC104885; BC104887; OTTHUMT00000353019; OTTHUMT00000353020; OTTHUMT00000353021; OTTHUMT00000353022; OTTHUMT00000353023; uc003fdr.3; uc003fds.3; uc003fdt.3; uc010hwf.3 | *PPM1L* | protein phosphatase, Mg2+/Mn2+ dependent, 1L | 4.4 | 4.57 | -1.12 | 0.0336 |
| TC0X000789.hg.1 | NR_039991; ENST00000433624; ENST00000452506; uc004fnl.3 | *TMLHE-AS1* | TMLHE antisense RNA 1 | 4.22 | 4.14 | 1.06 | 0.0336 |
| TC06000034.hg.1 | ENST00000356722 | *C6ORF50* |  | 1.51 | 1.37 | 1.1 | 0.0337 |
| TC01003191.hg.1 | NR_036461; uc021oxz.1 | *HIST2H2BC* | histone cluster 2, H2bc (pseudogene) | 7.35 | 7.11 | 1.19 | 0.0337 |
| TC12001612.hg.1 | NM_006601; ENST00000262033; ENST00000414274; ENST00000436399; ENST00000448157; ENST00000456859; ENST00000537473; AK098214; AK295208; AK298147; AK298160; BC003005; uc001slu.4; uc001slv.4; uc001slw.4; uc010sqs.2; uc010sqt.2 | *PTGES3* | prostaglandin E synthase 3 (cytosolic) | 7.17 | 7.09 | 1.06 | 0.0337 |
| TC05000777.hg.1 | NM_030571; ENST00000253814; ENST00000509436; AK075495; BC004317; OTTHUMT00000251859; OTTHUMT00000370657; OTTHUMT00000370658; OTTHUMT00000370659; uc003lmi.4; uc003lmj.1 | *NDFIP1* | Nedd4 family interacting protein 1 | 5.82 | 5.71 | 1.08 | 0.0338 |
| TC07000385.hg.1 | BC053669 | *ZNF107* | zinc finger protein 107 | 7.9 | 7.55 | 1.28 | 0.0338 |
| TC03001404.hg.1 | NM_022903; ENST00000321895; BC035516; OTTHUMT00000345980; uc003cwg.4 | *CCDC71* | coiled-coil domain containing 71 | 6.38 | 6.31 | 1.05 | 0.0339 |
| TC20000714.hg.1 | NM_001008693; ENST00000376971; BC137302; OTTHUMT00000078341; uc002wtl.3 | *CST9* | cystatin 9 (testatin) | 3.95 | 4.02 | -1.05 | 0.0339 |
| TC17001839.hg.1 | ENST00000446332 | *SOX9-AS1* | SOX9 antisense RNA 1 | 3.35 | 3.48 | -1.1 | 0.0339 |
| TC15001576.hg.1 | NM_017858; ENST00000261881; AK299233; BC000870; OTTHUMT00000256897; uc002apr.2; uc010ujo.1 | *TIPIN* | TIMELESS interacting protein | 2.93 | 3.04 | -1.08 | 0.0339 |
| TC01003194.hg.1 | NM_175065; ENST00000331128; BC132811; OTTHUMT00000033440; uc001ete.3 | *HIST2H2AB* | histone cluster 2, H2ab | 5.42 | 5.14 | 1.21 | 0.034 |
| TC01002741.hg.1 | NM_002227; ENST00000342505; ENST00000465376; AK314257; BC132729; BX647675; OTTHUMT00000025786; OTTHUMT00000025787; OTTHUMT00000025788; OTTHUMT00000025789; OTTHUMT00000025790; OTTHUMT00000025791; uc001dbu.1; uc009wal.1; uc009wam.1 | *JAK1* | Janus kinase 1 | 8.71 | 8.9 | -1.14 | 0.034 |
| TC11000513.hg.1 | NR_026946; ENST00000320202; ENST00000412599; ENST00000527161; ENST00000532591; BC029583; uc001npt.3; uc001npu.3 | *LINC00301* | long intergenic non-protein coding RNA 301 | 1.31 | 1.27 | 1.02 | 0.034 |
| TC6_ssto_hap7000094.hg.1 | NM_152735; BC014978; OTTHUMT00000276523; uc011jvm.2 | *ZBTB9* | zinc finger and BTB domain containing 9 | 4.74 | 4.89 | -1.11 | 0.034 |
| TC01003408.hg.1 | NM_001166663; NM_001166664; NM_016382; ENST00000322302; ENST00000368033; ENST00000368034; ENST00000481677; AJ245377; AK295950; BC028073; BC053985; OTTHUMT00000071468; OTTHUMT00000071469; OTTHUMT00000071470; OTTHUMT00000071471; OTTHUMT00000071472; uc001fxa.3; uc009wtp.3; uc009wtq.3; uc009wtr.3; uc010pjt.2 | *CD244* | CD244 molecule, natural killer cell receptor 2B4 | 5.54 | 5.71 | -1.12 | 0.0341 |
| TC18000314.hg.1 | NR_024419; uc010wzm.1 | *LOC100192426* | uncharacterized LOC100192426 | 5.25 | 5.34 | -1.07 | 0.0341 |
| TC11000467.hg.1 | NM_000062; NM_001032295; ENST00000278407; ENST00000340687; ENST00000378323; ENST00000378324; ENST00000403558; ENST00000531605; AK303809; AK303840; AY732485; BC011171; OTTHUMT00000317465; OTTHUMT00000317466; OTTHUMT00000317467; OTTHUMT00000317468; OTTHUMT00000317469; uc001nkp.1; uc001nkr.1; uc001nks.1; uc010rju.1; uc010rjv.1 | *SERPING1* | serpin peptidase inhibitor, clade G (C1 inhibitor), member 1 | 6.09 | 5.82 | 1.2 | 0.0341 |
| TC10001523.hg.1 | ENST00000432246; ENST00000432938 | *TNKS2-AS1* | TNKS2 antisense RNA 1 (head to head) | 2.67 | 2.74 | -1.05 | 0.0341 |
| TC07000889.hg.1 | NM_018238; ENST00000355413; ENST00000473247; ENST00000492693; ENST00000495028; AK299131; BC009775; BC022777; OTTHUMT00000348969; OTTHUMT00000348970; OTTHUMT00000348971; OTTHUMT00000348972; OTTHUMT00000348973; OTTHUMT00000348974; OTTHUMT00000348975; OTTHUMT00000348976; OTTHUMT00000348977; OTTHUMT00000348978; uc003vwh.2; uc003vwi.2; uc011krg.1 | *AGK* | acylglycerol kinase | 5.54 | 5.68 | -1.1 | 0.0342 |
| TC06001030.hg.1 | NM_014320; ENST00000367697; ENST00000448741; BC008205; BC010290; BC093037; OTTHUMT00000042426; OTTHUMT00000042427; OTTHUMT00000042428; OTTHUMT00000348263; uc003qhw.1 | *HEBP2* | heme binding protein 2 | 6.99 | 6.89 | 1.07 | 0.0342 |
| TC09000525.hg.1 | ENST00000425157 | *LINC01492* | long intergenic non-protein coding RNA 1492 | 2.2 | 2.01 | 1.14 | 0.0342 |
| TC04000013.hg.1 | NM_006315; ENST00000362003; ENST00000400151; ENST00000470161; ENST00000482726; ENST00000505655; AK057124; AK125801; AK225892; AK300290; BC050044; BC107061; BX648409; OTTHUMT00000239197; OTTHUMT00000318553; OTTHUMT00000318554; OTTHUMT00000318555; OTTHUMT00000318556; OTTHUMT00000318557; OTTHUMT00000318558; OTTHUMT00000318559; OTTHUMT00000318560; OTTHUMT00000318561; OTTHUMT00000318562; OTTHUMT00000318563; uc003gbd.1; uc003gbe.3; uc003gbh.3; uc010ibh.3; uc011bva.1 | *PCGF3* | polycomb group ring finger 3 | 6.23 | 6.09 | 1.1 | 0.0342 |
| TC08002577.hg.1 | NR_023392 | *ZNF252P* | zinc finger protein 252, pseudogene | 4.82 | 4.66 | 1.12 | 0.0344 |
| TC12003003.hg.1 | AF086029 | *LINC01619* | long intergenic non-protein coding RNA 1619 | 3.29 | 3.19 | 1.07 | 0.0345 |
| TC03001227.hg.1 | NM_001012409; NM_001012410; NM_001012411; NM_001012412; NM_001012413; NM_001199251; NM_001199252; NM_001199253; NM_001199254; NM_001199255; NM_001199256; NM_001199257; NM_138484; ENST00000263753; ENST00000306698; ENST00000412997; ENST00000417364; ENST00000419233; ENST00000421451; ENST00000425061; ENST00000437051; ENST00000442720; ENST00000443724; ENST00000452020; ENST00000460637; AB567656; AB567657; BC017867; OTTHUMT00000252878; OTTHUMT00000340012; OTTHUMT00000340492; OTTHUMT00000340493; OTTHUMT00000340494; OTTHUMT00000340495; OTTHUMT00000340496; OTTHUMT00000340498; OTTHUMT00000340499; OTTHUMT00000340501; OTTHUMT00000340504; OTTHUMT00000340541; OTTHUMT00000340542; uc003cbr.3; uc003cbs.3; uc003cbt.3; uc003cbu.3; uc003cbv.3; uc003cbw.3; uc003cbx.3; uc003cby.3; uc003cbz.3; uc003cca.3; uc003ccb.3; uc003ccc.3; uc010hfa.3; uc021wtx.1; uc021wty.1 | *SGOL1* | shugoshin-like 1 (S. pombe) | 3.25 | 3.1 | 1.11 | 0.0345 |
| TC14002326.hg.1 | NM_001164399; ENST00000281581; ENST00000537690; ENST00000556936; ENST00000556996; uc021rtw.1 | *CCDC175* | coiled-coil domain containing 175 | 1.51 | 1.5 | 1.01 | 0.0346 |
| TC09002285.hg.1 | DQ656057 | *NUP188* | nucleoporin 188kDa | 5.03 | 5.14 | -1.08 | 0.0346 |
| TC03000891.hg.1 | ENST00000498413 | *LINC01327* | long intergenic non-protein coding RNA 1327 | 1.53 | 1.46 | 1.05 | 0.0347 |
| TC17000402.hg.1 | NM_003487; NM_139215; AK307637; BC030591; BC046099; uc010ctw.1 | *TAF15* | TATA box binding protein associated factor 15 | 7.56 | 7.69 | -1.09 | 0.0347 |
| TC05002082.hg.1 | NR_046113 | *FLJ16171* | FLJ16171 protein | 2.22 | 2.17 | 1.03 | 0.0348 |
| TC06004073.hg.1 | NM_000247; NM_001177519; ENST00000449934; AK094237; BC016929; NR_036523; NR_036524; OTTHUMT00000076101; OTTHUMT00000258103; uc003ntk.1; uc003rxz.1 | *MICA* | MHC class I polypeptide-related sequence A | 5.43 | 5.33 | 1.07 | 0.0348 |
| TC11001870.hg.1 | NM_004739; ENST00000278823; ENST00000524902; ENST00000527204; AK301569; BC053650; uc001ntq.2; uc010rlx.1 | *MTA2* | metastasis associated 1 family member 2 | 6.89 | 7.02 | -1.1 | 0.0348 |
| TC04000988.hg.1 | NM_032927; ENST00000254742; ENST00000382753; AK295248; AK296007; BC007729; BC091482; CR933611; OTTHUMT00000246797; OTTHUMT00000246798; uc003ghq.1; uc003ghr.1; uc003ghs.3; uc011bvv.1; uc011bvw.1 | *TMEM128* | transmembrane protein 128 | 4.26 | 4.13 | 1.09 | 0.0348 |
| TC05003418.hg.1 | ENST00000433265; ENST00000507434; ENST00000509252; ENST00000514146; BC016291 | *TRIM52-AS1* | TRIM52 antisense RNA 1 (head to head) | 5.16 | 5.09 | 1.05 | 0.0348 |
| TC16000725.hg.1 | NM_003502; NM_181050; ENST00000262320; ENST00000354866; ENST00000481769; BC044648; OTTHUMT00000139441; OTTHUMT00000139442; OTTHUMT00000139444; OTTHUMT00000139445; OTTHUMT00000313678; uc002cgp.2; uc002cgq.2 | *AXIN1* | axin 1 | 6.5 | 6.52 | -1.02 | 0.0349 |
| TC0X001067.hg.1 | NM_031407; ENST00000218328; ENST00000262854; ENST00000342160; ENST00000474288; AB002310; BC072421; OTTHUMT00000056766; OTTHUMT00000056767; OTTHUMT00000056768; OTTHUMT00000056769; OTTHUMT00000056770; OTTHUMT00000056771; OTTHUMT00000056772; OTTHUMT00000056773; OTTHUMT00000056774; OTTHUMT00000056775; OTTHUMT00000056776; OTTHUMT00000056777; OTTHUMT00000056778; OTTHUMT00000056779; uc004dsq.1 | *HUWE1* | HECT, UBA and WWE domain containing 1, E3 ubiquitin protein ligase | 7.15 | 7.26 | -1.08 | 0.0349 |
| TC10002239.hg.1 | BC015656 | *LOXL4* | lysyl oxidase-like 4 | 4.17 | 4.07 | 1.08 | 0.0349 |
| TC01004068.hg.1 | NR_037480; uc021pma.1 | *MIR3916* | microRNA 3916 | 6.6 | 6.71 | -1.08 | 0.0349 |
| TC06002338.hg.1 | AK023627 | *TCTE3* | t-complex-associated-testis-expressed 3 | 3.34 | 3.25 | 1.07 | 0.0349 |
| TC05001273.hg.1 | OTTHUMT00000366970 | *CTD-2653M23.2* |  | 4.78 | 4.63 | 1.11 | 0.035 |
| TC05001943.hg.1 | NM_001135643; NM_001135644; NM_016221; ENST00000424236; ENST00000446090; ENST00000447998; ENST00000521093; AK299517; AK311693; BC026323; OTTHUMT00000252372; OTTHUMT00000374554; OTTHUMT00000374557; OTTHUMT00000374559; OTTHUMT00000374560; OTTHUMT00000374561; OTTHUMT00000374562; OTTHUMT00000374563; OTTHUMT00000374641; OTTHUMT00000374642; OTTHUMT00000374643; OTTHUMT00000374644; OTTHUMT00000374645; OTTHUMT00000374646; uc003lsu.3; uc003lsv.3; uc010jhi.3; uc010jhj.2; uc011dck.1 | *DCTN4* | dynactin 4 (p62) | 6.43 | 6.38 | 1.04 | 0.035 |
| TC02001550.hg.1 | NR_034135; AK127578; OTTHUMT00000231926; OTTHUMT00000276814; OTTHUMT00000323258; OTTHUMT00000323259; uc002qyy.1; uc010eww.2 | *LINC00299* | long intergenic non-protein coding RNA 299 | 4.51 | 4.73 | -1.16 | 0.035 |
| TC11000097.hg.1 | NM_001004751; ENST00000357605; BC140717; uc010qyk.2 | *OR51D1* | olfactory receptor, family 51, subfamily D, member 1 | 2.49 | 2.59 | -1.07 | 0.035 |
| TC07000004.hg.1 | NM_020223; ENST00000313766; ENST00000471328; AL390147; BC040074; OTTHUMT00000322476; OTTHUMT00000322477; OTTHUMT00000322478; uc003sip.3; uc011jvn.2 | *FAM20C* | family with sequence similarity 20, member C | 5.23 | 5.3 | -1.05 | 0.0351 |
| TC07000945.hg.1 | NM_012369; ENST00000392899; BC104975; OTTHUMT00000349581; uc003wds.1 | *OR2F1* | olfactory receptor, family 2, subfamily F, member 1 (gene/pseudogene) | 2.76 | 2.88 | -1.08 | 0.0351 |
| TC01002632.hg.1 | NR_026878; ENST00000445551; uc001crl.3 | *FOXD2-AS1* | FOXD2 antisense RNA 1 (head to head) | 5.31 | 5.48 | -1.12 | 0.0352 |
| TC01001595.hg.1 | NM_007212; ENST00000367509; ENST00000367510; BC012583; OTTHUMT00000085793; OTTHUMT00000085794; OTTHUMT00000085795; OTTHUMT00000085796; uc001grc.1 | *RNF2* | ring finger protein 2 | 5.28 | 5.23 | 1.03 | 0.0352 |
| TC05001182.hg.1 | NM_001034850; NM_019000; ENST00000306320; ENST00000399793; ENST00000509048; BC053326; BC073132; OTTHUMT00000366090; OTTHUMT00000366091; OTTHUMT00000366092; OTTHUMT00000366093; OTTHUMT00000366094; OTTHUMT00000366095; uc003jfr.3; uc003jfs.3 | *FAM134B* | family with sequence similarity 134, member B | 4.67 | 4.47 | 1.15 | 0.0353 |
| TC11002242.hg.1 | NM_025208; NM_033135; ENST00000302251; ENST00000393158; BC030645; uc001php.3; uc001phq.3 | *PDGFD* | platelet derived growth factor D | 4.36 | 4.46 | -1.07 | 0.0353 |
| TC07000245.hg.1 | NM_032016; ENST00000009041; ENST00000396013; ENST00000434197; BC003074; BC005959; uc003tfr.3 | *STARD3NL* | STARD3 N-terminal like | 4.99 | 4.93 | 1.04 | 0.0353 |
| TC06002281.hg.1 | NM_005577; ENST00000316300; ENST00000447678; OTTHUMT00000042957; uc003qtl.3 | *LPA* | lipoprotein, Lp(a) | 3.46 | 3.55 | -1.07 | 0.0354 |
| TC02003391.hg.1 | X57416 | *RPIA* | ribose 5-phosphate isomerase A | 2.85 | 3.01 | -1.12 | 0.0354 |
| TC03001490.hg.1 | NM_003865; ENST00000295934; ENST00000473921; BC069515; BC093979; OTTHUMT00000351430; OTTHUMT00000351431; uc003din.4 | *HESX1* | HESX homeobox 1 | 1.32 | 1.22 | 1.07 | 0.0355 |
| TC17001386.hg.1 | NM_001001417; BC071680; uc002hky.2 | *TBC1D3B; TBC1D3G* | TBC1 domain family, member 3B; TBC1 domain family, member 3G | 6.29 | 6.16 | 1.1 | 0.0355 |
| TC04001153.hg.1 | ENST00000508563 | *LOC105374436* | uncharacterized LOC105374436 | 1.96 | 1.76 | 1.14 | 0.0356 |
| TC0X001498.hg.1 | NR_030409; ENST00000390228; uc022chd.1 | *MIR767* | microRNA 767 | 2.05 | 2.3 | -1.19 | 0.0356 |
| TC19002265.hg.1 | X65231 | *ZNF135* | zinc finger protein 135 | 6.34 | 6.5 | -1.11 | 0.0356 |
| TC06001789.hg.1 | NM_001166478; ENST00000398721; uc003ozy.2 | *DEFB133* | defensin, beta 133 | 1.43 | 1.32 | 1.08 | 0.0357 |
| TC03001978.hg.1 | NM_014498; ENST00000309027; ENST00000470487; BC143232; BC143233; BC171748; OTTHUMT00000351278; OTTHUMT00000351279; OTTHUMT00000351280; uc003ffe.2; uc011bpe.1; uc011bpf.1; uc011bpg.1 | *GOLIM4* | golgi integral membrane protein 4 | 4.79 | 4.91 | -1.08 | 0.0357 |
| TC10001363.hg.1 | NM_022146; ENST00000277942; BC131580; OTTHUMT00000048504; uc021psj.1 | *NPFFR1* | neuropeptide FF receptor 1 | 4.5 | 4.4 | 1.07 | 0.0357 |
| TC07001811.hg.1 | NM_005763; ENST00000393376; ENST00000417368; ENST00000473553; AJ007714; AK294326; BC144246; BC144247; OTTHUMT00000347298; OTTHUMT00000347299; OTTHUMT00000347300; OTTHUMT00000347301; OTTHUMT00000347302; OTTHUMT00000347303; OTTHUMT00000347304; uc003vka.3; uc003vkb.3; uc011knu.2; uc011knv.2; uc011knw.2 | *AASS* | aminoadipate-semialdehyde synthase | 2.65 | 2.75 | -1.08 | 0.0358 |
| TC11001805.hg.1 | NM_207374; ENST00000395079; BC132848; uc001nmq.1 | *OR10W1* | olfactory receptor, family 10, subfamily W, member 1 | 2.21 | 2.14 | 1.05 | 0.0359 |
| TC17001582.hg.1 | NM_001002909; ENST00000335500; AB011125; BC136629; NR_036474; uc002igv.2; uc002igw.2 | *GPATCH8* | G-patch domain containing 8 | 6.99 | 7.06 | -1.05 | 0.036 |
| TC0X001098.hg.1 | NM_001170649; NM_001170650; NM_031206; ENST00000374804; ENST00000374807; ENST00000374811; ENST00000484069; AK125760; AK127207; BC014545; BC018610; BC019302; OTTHUMT00000056972; OTTHUMT00000056973; OTTHUMT00000056974; OTTHUMT00000056975; OTTHUMT00000056976; uc004dvy.1; uc004dvz.2; uc004dwa.2; uc004dwc.2; uc004dwd.2 | *LAS1L* | LAS1-like, ribosome biogenesis factor | 5.21 | 5.29 | -1.06 | 0.036 |
| TC07001391.hg.1 | NR_038371; ENST00000380970 | *LINC01446* | long intergenic non-protein coding RNA 1446 | 2.38 | 2.42 | -1.03 | 0.036 |
| TC18000465.hg.1 | NR_024391; AK090603; BC035184 | *MIR924HG* | MIR924 host gene | 1.66 | 1.58 | 1.05 | 0.036 |
| TC08001988.hg.1 | S76965 | *PKIA* | protein kinase (cAMP-dependent, catalytic) inhibitor alpha | 4.2 | 4.06 | 1.1 | 0.036 |
| TC15000311.hg.1 | NM_001080541; NM_001164273; ENST00000219905; ENST00000545763; AK301207; BC038449; BC136659; BX648098; uc001zog.1; uc001zoi.3; uc010ucy.2; uc010ucz.2; uc010uda.1 | *MGA* | MGA, MAX dimerization protein | 6.24 | 6.33 | -1.06 | 0.0361 |
| TC09000783.hg.1 | NR_039816; uc022bpa.1 | *MIR4669* | microRNA 4669 | 6.54 | 6.68 | -1.1 | 0.0361 |
| TC6_dbb_hap3000008.hg.1 | NM_013936; BC069123; BC101742; BC101746; OTTHUMT00000309666; uc011fsc.2 | *OR12D2* | olfactory receptor, family 12, subfamily D, member 2 (gene/pseudogene) | 1.26 | 1.16 | 1.07 | 0.0361 |
| TC6_ssto_hap7000010.hg.1 | NM_013936; BC069123; BC101742; BC101746; OTTHUMT00000251122; uc011jbw.2 | *OR12D2* | olfactory receptor, family 12, subfamily D, member 2 (gene/pseudogene) | 1.26 | 1.16 | 1.07 | 0.0361 |
| TC01000733.hg.1 | NM_001037339; NM_001037340; NM_001037341; NM_002600; ENST00000329654; ENST00000341517; ENST00000371045; ENST00000371048; ENST00000423207; ENST00000480109; BC101480; CR749667; EF595686; OTTHUMT00000025188; OTTHUMT00000025189; OTTHUMT00000025190; OTTHUMT00000025191; OTTHUMT00000025192; OTTHUMT00000025193; OTTHUMT00000025194; OTTHUMT00000127844; uc001dcn.3; uc001dco.3; uc001dcp.3; uc001dcq.3; uc009war.3; uc009was.3 | *PDE4B* | phosphodiesterase 4B, cAMP-specific | 5.13 | 5.01 | 1.08 | 0.0363 |
| TC09000782.hg.1 | NM_002957; ENST00000356384; ENST00000481739; AK090416; AK131081; AK131192; BC110998; OTTHUMT00000054948; OTTHUMT00000054949; OTTHUMT00000054950; uc004cfa.1; uc004cfb.2; uc004cfc.1; uc004cfd.1 | *RXRA* | retinoid X receptor alpha | 7.35 | 7.23 | 1.09 | 0.0363 |
| TC01003403.hg.1 | NM_001184714; NM_001184715; NM_001184716; NM_052931; ENST00000368055; ENST00000368057; ENST00000368059; AK300013; AK310085; BC113893; BC114495; OTTHUMT00000059010; OTTHUMT00000059011; OTTHUMT00000059012; uc001fwd.2; uc001fwe.2; uc009wtm.2; uc010pjh.2; uc010pji.2; uc010pjj.2 | *SLAMF6* | SLAM family member 6 | 7.57 | 7.78 | -1.16 | 0.0363 |
| TC0X001274.hg.1 | NR_004379; ENST00000386148; uc010npr.1 | *SNORD96B* | small nucleolar RNA, C/D box 96B | 3.59 | 3.4 | 1.14 | 0.0363 |
| TC07002205.hg.1 | NR_037670 | *SNX10* | sorting nexin 10 | 7.07 | 6.89 | 1.13 | 0.0363 |
| TC09000601.hg.1 | NM_003266; NM_138554; NM_138557; ENST00000355622; ENST00000394487; ENST00000472304; BC117422; OTTHUMT00000055549; OTTHUMT00000055550; OTTHUMT00000055551; OTTHUMT00000055552 | *TLR4* | toll-like receptor 4 | 8.1 | 7.66 | 1.36 | 0.0363 |
| TC06000425.hg.1 | NM_152735; ENST00000395064; BC014978; OTTHUMT00000276533; uc003oeq.3 | *ZBTB9* | zinc finger and BTB domain containing 9 | 4.81 | 4.9 | -1.07 | 0.0363 |
| TC05000462.hg.1 | NR_026936; AY168789 | *LINC01554* | long intergenic non-protein coding RNA 1554 | 4.83 | 4.77 | 1.05 | 0.0364 |
| TC22000845.hg.1 | NM_145912; ENST00000329021; BC038241; OTTHUMT00000320541; OTTHUMT00000320542; uc003bcn.4 | *NFAM1* | NFAT activating protein with ITAM motif 1 | 7.89 | 7.69 | 1.15 | 0.0364 |
| TC04001508.hg.1 | NM_018699; ENST00000264808; ENST00000394435; ENST00000428209; ENST00000506065; ENST00000515109; AK310958; BC121037; BC121038; OTTHUMT00000256528; OTTHUMT00000363927; OTTHUMT00000363928; OTTHUMT00000363929; OTTHUMT00000363930; OTTHUMT00000363931; OTTHUMT00000363932; OTTHUMT00000363933; OTTHUMT00000363934; OTTHUMT00000363935; OTTHUMT00000363936; OTTHUMT00000363937; uc003idn.3; uc010ine.3; uc010inf.3 | *PRDM5* | PR domain containing 5 | 3.44 | 3.3 | 1.1 | 0.0364 |
| TC11002183.hg.1 | NR_038146 | *TRIM51EP* | tripartite motif-containing 51E, pseudogene | 4.68 | 4.24 | 1.36 | 0.0364 |
| TC10000781.hg.1 | NM_001191002; NM_001191003; NM_004832; ENST00000369710; ENST00000369713; ENST00000493946; ENST00000539281; BC000127; OTTHUMT00000050193; OTTHUMT00000050194; OTTHUMT00000050195; OTTHUMT00000050196; OTTHUMT00000050197; uc001kya.3; uc021pxr.1; uc021pxs.1 | *GSTO1* | glutathione S-transferase omega 1 | 5.01 | 4.93 | 1.06 | 0.0366 |
| TC10001098.hg.1 | NM_001199938; uc021pob.1 | *EBLN1* | endogenous Bornavirus-like nucleoprotein 1 | 1.96 | 1.86 | 1.07 | 0.0367 |
| TC05002400.hg.1 | AY429552 | *KIF2A* | kinesin heavy chain member 2A | 7.85 | 7.79 | 1.05 | 0.0369 |
| TC02001549.hg.1 | NR_015405; ENST00000426969; ENST00000456681; BC104747 | *LINC00298* | long intergenic non-protein coding RNA 298 | 4.47 | 4.57 | -1.08 | 0.0369 |
| TC17001775.hg.1 | NR_027486; BC058890; uc002izq.2; uc010woz.2 | *TBC1D3P2* | TBC1 domain family, member 3 pseudogene 2 | 5.39 | 5.31 | 1.05 | 0.0369 |
| TC11002439.hg.1 | NM_001143820; NM_001162422; NM_005238; ENST00000319397; ENST00000392668; ENST00000525404; ENST00000526145; ENST00000531611; ENST00000535549; BC017314; uc001qej.2; uc009zcg.2; uc009zch.2; uc010sbs.1 | *ETS1* | v-ets avian erythroblastosis virus E26 oncogene homolog 1 | 8 | 8.05 | -1.03 | 0.037 |
| TC15002760.hg.1 | NM_001039841; ENST00000428041; AK310030; BC105788; uc001zet.1 | *ARHGAP11B* | Rho GTPase activating protein 11B | 4.81 | 4.64 | 1.12 | 0.0372 |
| TC08000006.hg.1 | ENST00000520524; ENST00000522989 | *LOC105379627* | uncharacterized LOC105379627 | 4.26 | 4.29 | -1.02 | 0.0372 |
| TC04000023.hg.1 | NM_001017405; NM_005882; ENST00000264750; ENST00000303400; ENST00000505177; ENST00000505839; ENST00000510794; ENST00000512289; ENST00000514708; AK022515; AK094253; AK296174; AK298944; AK301155; AK308901; AY236486; BC001225; OTTHUMT00000359511; OTTHUMT00000359512; OTTHUMT00000359513; OTTHUMT00000359514; OTTHUMT00000359515; OTTHUMT00000359516; OTTHUMT00000359517; OTTHUMT00000359518; OTTHUMT00000359519; OTTHUMT00000359520; OTTHUMT00000359521; OTTHUMT00000359522; OTTHUMT00000359523; OTTHUMT00000359524; OTTHUMT00000359525; OTTHUMT00000359526; OTTHUMT00000359527; OTTHUMT00000359528; OTTHUMT00000359529; OTTHUMT00000359530; uc003gda.3; uc003gdb.3; uc003gdc.3; uc003gdd.3; uc010ibs.1; uc010ibt.3; uc011bvb.2; uc011bvc.2; uc011bvd.2 | *MAEA* | macrophage erythroblast attacher | 5.95 | 5.88 | 1.05 | 0.0372 |
| TC05001179.hg.1 | NM_001102562; ENST00000332432; ENST00000505509; BC150514; OTTHUMT00000366096; OTTHUMT00000366097; OTTHUMT00000366098; uc003jfo.2; uc010itw.1 | *Mar-11* | membrane associated ring finger 11 | 2.5 | 2.57 | -1.05 | 0.0373 |
| TC01000746.hg.1 | NR_040077; ENST00000420587; AK096081; uc001deb.2 | *GNG12-AS1* | GNG12 antisense RNA 1 | 2.85 | 2.76 | 1.06 | 0.0374 |
| TC01004604.hg.1 | NR_034073 | *SARS* | seryl-tRNA synthetase | 5.84 | 5.75 | 1.06 | 0.0374 |
| TC13000050.hg.1 | NM_001014442; NM_001135816; uc001uoy.3; uc009zzx.3 | *C1QTNF9B-AS1* | C1QTNF9B antisense RNA 1 | 3.38 | 3.45 | -1.05 | 0.0375 |
| TC02004405.hg.1 | AL137712; NR_003366 | *FAM95A; ANKRD20A8P* | family with sequence similarity 95, member A; ankyrin repeat domain 20 family, member A8, pseudogene | 3.66 | 3.55 | 1.08 | 0.0375 |
| TC14002104.hg.1 | AK130854 | *LINC01467* | long intergenic non-protein coding RNA 1467 | 1.68 | 1.62 | 1.04 | 0.0375 |
| TC03000312.hg.1 | NM_007022; ENST00000232508; ENST00000418577; ENST00000419046; ENST00000424512; ENST00000425346; AF040704; BC047691; OTTHUMT00000345970; OTTHUMT00000345971; OTTHUMT00000345972; OTTHUMT00000345973; OTTHUMT00000346206; OTTHUMT00000346207; uc003dal.3; uc003dam.3 | *CYB561D2* | cytochrome b561 family, member D2 | 5.24 | 5.18 | 1.04 | 0.0376 |
| TC11000924.hg.1 | NM_130847; ENST00000317829; ENST00000433060; ENST00000539727; BC037539; uc001pfb.3; uc001pfc.3 | *AMOTL1* | angiomotin like 1 | 4.79 | 4.87 | -1.06 | 0.0377 |
| TC06001428.hg.1 | NM_003546; BC069392; OTTHUMT00000043513; uc003njz.3 | *HIST1H4L* | histone cluster 1, H4l | 4.31 | 4.09 | 1.16 | 0.0378 |
| TC02003384.hg.1 | DQ786261 | *LOC285074* | anaphase promoting complex subunit 1 pseudogene | 5.54 | 5.38 | 1.12 | 0.0378 |
| TC17000156.hg.1 | NR_030613; uc021tqg.1 | *MIR744* | microRNA 744 | 4.66 | 4.56 | 1.07 | 0.0378 |
| TC01001124.hg.1 | ENST00000459025 | *RNA5SP58* | RNA, 5S ribosomal pseudogene 58 | 1.9 | 1.78 | 1.08 | 0.0378 |
| TC15000101.hg.1 | NR_003346; ENST00000365318; uc001yzz.1 | *SNORD115-31* | small nucleolar RNA, C/D box 115-31 | 1.17 | 1.05 | 1.09 | 0.0378 |
| TC0X000628.hg.1 | NM_001042452; NM_001042453; NM_016542; ENST00000354719; ENST00000394334; ENST00000394335; ENST00000481105; ENST00000496850; AK075150; AK303583; BC098315; BC099843; OTTHUMT00000058306; OTTHUMT00000058308; OTTHUMT00000355065; OTTHUMT00000355066; OTTHUMT00000355067 | *STK26* | serine/threonine protein kinase 26 | 6.98 | 7.03 | -1.03 | 0.0378 |
| TC11000889.hg.1 | NM_001146162; uc010rtw.2 | *TRIM77* | tripartite motif containing 77 | 2.14 | 2.24 | -1.08 | 0.0378 |
| TC14001155.hg.1 | NM_001146015; NM_014750; ENST00000247191; ENST00000395425; ENST00000554067; BC010658; BC016276; OTTHUMT00000276908; OTTHUMT00000276909; uc001xbs.3; uc001xbt.3 | *DLGAP5* | discs, large (Drosophila) homolog-associated protein 5 | 2.74 | 2.64 | 1.07 | 0.0379 |
| TC6_cox_hap2000114.hg.1 | NR_033379 | *LINC01623* | long intergenic non-protein coding RNA 1623 | 2.54 | 2.42 | 1.09 | 0.0379 |
| TC09000340.hg.1 | ENST00000516350 | *RNY4P1* | RNA, Ro-associated Y4 pseudogene 1 | 2.79 | 2.35 | 1.35 | 0.0379 |
| TC15000356.hg.1 | NM_024063; NR_027635; ENST00000305560; ENST00000533841; ENST00000559860; BC000981; OTTHUMT00000254218; uc001zve.3; uc001zvf.3 | *SPATA5L1* | spermatogenesis associated 5-like 1 | 4.58 | 4.47 | 1.08 | 0.0379 |
| TC17000488.hg.1 | NM_001190918; NM_001190919; NM_003250; NM_199334; ENST00000264637; ENST00000394121; ENST00000450525; ENST00000546243; AK311344; BC000261; BC008851; BC035137; OTTHUMT00000257160; OTTHUMT00000257161; uc002htv.3; uc002htw.3; uc002htx.3; uc010cwp.1; uc021twy.1 | *THRA* | thyroid hormone receptor, alpha | 6.49 | 6.55 | -1.04 | 0.0379 |
| TC01002163.hg.1 | NM_001561; ENST00000377507; ENST00000492571; BC006196; OTTHUMT00000003622; OTTHUMT00000003623; OTTHUMT00000083600; uc001aot.3 | *TNFRSF9* | tumor necrosis factor receptor superfamily, member 9 | 5.11 | 4.79 | 1.25 | 0.0379 |
| TC20001537.hg.1 | D83784 | *PLAGL2* | pleiomorphic adenoma gene-like 2 | 6.82 | 6.94 | -1.08 | 0.038 |
| TC02002499.hg.1 | NM_002977; ENST00000303354; ENST00000409435; ENST00000409672; AY682084; AY682085; AY682086; OTTHUMT00000333636; OTTHUMT00000333637; OTTHUMT00000333638; OTTHUMT00000333639; OTTHUMT00000333640; uc002udr.1; uc002uds.1; uc002udt.1; uc010fpl.3 | *SCN9A* | sodium channel, voltage gated, type IX alpha subunit | 2.19 | 2.09 | 1.07 | 0.0381 |
| TC11002355.hg.1 | NM_182557; ENST00000334801; ENST00000526143; ENST00000527266; AY296059; uc001pug.3; uc009zal.3 | *BCL9L* | B-cell CLL/lymphoma 9-like | 7.1 | 7.2 | -1.07 | 0.0382 |
| TC02001775.hg.1 | NM_001242815; ENST00000378711; ENST00000403980; OTTHUMT00000325755; OTTHUMT00000325756; uc002rsf.1 | *C2orf91* | chromosome 2 open reading frame 91 | 2.19 | 2.1 | 1.07 | 0.0382 |
| TC01002763.hg.1 | NM_001002292; NM_001193334; NM_024911; ENST00000262348; ENST00000354777; ENST00000370971; ENST00000370976; ENST00000491811; AB097018; AK309779; BC110826; BC137109; OTTHUMT00000025368; OTTHUMT00000025369; OTTHUMT00000025370; OTTHUMT00000025371; OTTHUMT00000025373; OTTHUMT00000025374; OTTHUMT00000025375; OTTHUMT00000025376; OTTHUMT00000091982; uc001dee.3; uc001def.2; uc001deg.2; uc001deh.2; uc009wbf.1 | *WLS* | wntless Wnt ligand secretion mediator | 6.31 | 5.71 | 1.51 | 0.0384 |
| TC10001445.hg.1 | NM_004747; ENST00000372391; ENST00000459739; AK055172; AK122931; BC146794; BX649131; OTTHUMT00000048900; OTTHUMT00000048901; OTTHUMT00000048902; OTTHUMT00000048903; OTTHUMT00000048904; OTTHUMT00000048905; OTTHUMT00000048906; OTTHUMT00000048907; OTTHUMT00000048909; U61843; uc001jzi.3; uc001jzj.3; uc001jzk.3; uc001jzl.4; uc009xru.1 | *DLG5* | discs, large homolog 5 (Drosophila) | 5.15 | 5.26 | -1.07 | 0.0385 |
| TC02000395.hg.1 | NM_020143; ENST00000263657; BC008304; OTTHUMT00000251756; OTTHUMT00000326799; OTTHUMT00000326800; uc002seh.3 | *PNO1* | partner of NOB1 homolog | 4.12 | 4.17 | -1.03 | 0.0385 |
| TC15000875.hg.1 | ENST00000554388; ENST00000556200 | *PRC1-AS1* | PRC1 antisense RNA 1 | 2.86 | 2.77 | 1.06 | 0.0385 |
| TC02000551.hg.1 | OTTHUMT00000323040 | *IGKV5-2* | immunoglobulin kappa variable 5-2 | 4.43 | 4.26 | 1.13 | 0.0386 |
| TC01005124.hg.1 | AJ421679 | *LINC00184* | long intergenic non-protein coding RNA 184 | 2.18 | 2.27 | -1.06 | 0.0386 |
| TC03001395.hg.1 | NR_029948; ENST00000362162; uc011bcb.1 | *MIR425* | microRNA 425 | 3.96 | 3.79 | 1.13 | 0.0386 |
| TC17000953.hg.1 | NM_001038618; NM_001083608; NM_012336; NM_031968; ENST00000309794; ENST00000345415; ENST00000374611; ENST00000390006; ENST00000412079; ENST00000457415; AK297875; AK303155; BC000438; BC016440; uc002kff.4; uc002kfg.4; uc002kfj.4; uc010dit.3; uc010wvo.1; uc010wvp.1 | *NARF* | nuclear prelamin A recognition factor | 7 | 6.93 | 1.05 | 0.0386 |
| TC02001974.hg.1 | NM_020459; ENST00000244221; OTTHUMT00000330547; uc002shu.2 | *PAIP2B* | poly(A) binding protein interacting protein 2B | 3.91 | 4.01 | -1.07 | 0.0387 |
| TC01000537.hg.1 | NR_033967; ENST00000416689; uc001cil.3 | *SLC2A1-AS1* | SLC2A1 antisense RNA 1 | 3.76 | 3.68 | 1.05 | 0.0387 |
| TC17000922.hg.1 | NM_024591; ENST00000325167; BC010108; uc002jyw.4 | *CHMP6* | charged multivesicular body protein 6 | 5.76 | 5.83 | -1.05 | 0.0388 |
| TC02001128.hg.1 | NM_001031716; NM_001254736; NR_045622; NR_045623; ENST00000409510; ENST00000410026; ENST00000425611; AK023686; BC017114; BC107723; OTTHUMT00000256060; OTTHUMT00000256061; OTTHUMT00000334781; OTTHUMT00000334782; OTTHUMT00000334783; OTTHUMT00000334784; OTTHUMT00000334785; OTTHUMT00000334786; OTTHUMT00000334787; OTTHUMT00000334788; uc002usw.3; uc002usx.3; uc002usy.3; uc021vuf.1; uc021vug.1 | *NABP1* | nucleic acid binding protein 1 | 8.06 | 7.88 | 1.13 | 0.0388 |
| TC11002776.hg.1 | AK125753 | *ARAP1-AS2* | ARAP1 antisense RNA 2 | 4.73 | 4.54 | 1.14 | 0.0389 |
| TC19000272.hg.1 | NM_173483; ENST00000269703; BC069351; BC093894; uc002nbh.4 | *CYP4F22* | cytochrome P450, family 4, subfamily F, polypeptide 22 | 4.11 | 4.18 | -1.05 | 0.0389 |
| TC17002755.hg.1 | BC056895 | *HELZ* | helicase with zinc finger | 7.51 | 7.65 | -1.1 | 0.0389 |
| TC04001803.hg.1 | NR_040108; uc003iwd.2 | *LOC728175* | uncharacterized LOC728175 | 4.61 | 4.55 | 1.04 | 0.0389 |
| TC01001172.hg.1 | NM_000566; ENST00000369168; ENST00000489479; BC032634; BC152383; OTTHUMT00000033446; OTTHUMT00000033449; OTTHUMT00000095777; OTTHUMT00000095779; uc001esp.4 | *FCGR1A* | Fc fragment of IgG, high affinity Ia, receptor (CD64) | 6.58 | 6.2 | 1.3 | 0.039 |
| TC04001529.hg.1 | AK057455 | *LOC101927087* | uncharacterized LOC101927087 | 1.9 | 1.94 | -1.03 | 0.039 |
| TC09001276.hg.1 | NM_152573; ENST00000340717; ENST00000376447; AK056176; BC023566; OTTHUMT00000052825; OTTHUMT00000052826; uc004amp.1 | *RASEF* | RAS and EF-hand domain containing | 1.95 | 1.92 | 1.03 | 0.039 |
| TC19000320.hg.1 | NM_001130524; NM_032493; ENST00000291439; ENST00000429941; ENST00000444449; AK297824; BC017469; uc002ndu.2; uc002ndv.2; uc010xpd.1 | *AP1M1* | adaptor-related protein complex 1, mu 1 subunit | 6.96 | 7.02 | -1.04 | 0.0391 |
| TC12001933.hg.1 | NM_001142343; NM_001142344; NM_001142345; NM_004072; ENST00000312143; ENST00000412676; ENST00000550402; ENST00000552995; BC106927; uc001tmv.3; uc001tmw.3; uc009zuv.3; uc009zuw.3 | *CMKLR1* | chemerin chemokine-like receptor 1 | 5.25 | 5.44 | -1.14 | 0.0391 |
| TC04000856.hg.1 | NM_003864; ENST00000296504; BC016757; OTTHUMT00000362360; uc003itd.3 | *SAP30* | Sin3A associated protein 30kDa | 4.65 | 4.48 | 1.12 | 0.0391 |
| TC19001399.hg.1 | NR_027620; uc010xry.2 | *SCGB1B2P* | secretoglobin, family 1B, member 2, pseudogene | 4.6 | 4.71 | -1.07 | 0.0391 |
| TC07000914.hg.1 | NM_001190487; uc003vzz.2 | *MTRNR2L6* | MT-RNR2-like 6 | 3.66 | 3.98 | -1.25 | 0.0392 |
| TC01001645.hg.1 | NM_003822; NM_205860; ENST00000236914; ENST00000367362; ENST00000544748; AF124248; AK304344; AK304365; BC118571; OTTHUMT00000086497; OTTHUMT00000086498; OTTHUMT00000086499; OTTHUMT00000086500; OTTHUMT00000086501; uc009wzh.3 | *NR5A2* | nuclear receptor subfamily 5, group A, member 2 | 2.5 | 2.46 | 1.03 | 0.0392 |
| TC02004955.hg.1 | NM_002709; NM_206876; ENST00000296122; ENST00000358506; ENST00000395366; AK295957; AK303682; AK316532; BC002697; OTTHUMT00000324841; OTTHUMT00000324843; OTTHUMT00000324844; OTTHUMT00000324845; OTTHUMT00000324846; OTTHUMT00000324847; OTTHUMT00000324848; OTTHUMT00000324849; uc002rmg.3; uc002rmh.3; uc010ymj.2; uc010ymk.2; uc010yml.2 | *PPP1CB* | protein phosphatase 1, catalytic subunit, beta isozyme | 8.18 | 8.08 | 1.07 | 0.0392 |
| TC09002488.hg.1 | NR_024872 | *SLC25A51* | solute carrier family 25, member 51 | 6.39 | 6.51 | -1.08 | 0.0392 |
| TC15002763.hg.1 | NR_022014; AK096745; AY271965; AY271966; AY271967; BC062412; uc001zvk.3; uc001zvm.1; uc010beg.1; uc010beh.1; uc010bei.1 | *HMGN2P46* | high mobility group nucleosomal binding domain 2 pseudogene 46 | 3.5 | 3.56 | -1.05 | 0.0393 |
| TC01003115.hg.1 | NM_001201326; NR_003377; uc001eov.1 | *PDZK1; PDZK1P1* | PDZ domain containing 1; PDZ domain containing 1 pseudogene 1 | 3.18 | 3.29 | -1.08 | 0.0393 |
| TC14000666.hg.1 | NR_003230; ENST00000391082; uc001yij.3 | *SNORD113-2* | small nucleolar RNA, C/D box 113-2 | 1.03 | 0.95 | 1.06 | 0.0393 |
| TC05001976.hg.1 | NM_001146726; NM_138379; ENST00000274532; ENST00000406964; ENST00000407087; AK131025; BC008988; OTTHUMT00000252568; OTTHUMT00000317782; OTTHUMT00000317783; uc003lwg.2; uc003lwh.2; uc010jii.2 | *TIMD4* | T-cell immunoglobulin and mucin domain containing 4 | 3.03 | 2.81 | 1.16 | 0.0393 |
| TC12001282.hg.1 | NM_004447; ENST00000281172; ENST00000540613; ENST00000542903; ENST00000543523; ENST00000543612; AK292931; AK301834; BC005836; BC030010; uc001rdb.3; uc009zif.3; uc009zig.3; uc010shv.2 | *EPS8* | epidermal growth factor receptor pathway substrate 8 | 3.05 | 3.1 | -1.04 | 0.0394 |
| TC07000844.hg.1 | NM_001130929; ENST00000422968; ENST00000507606; BC040872; OTTHUMT00000340356; OTTHUMT00000366971; OTTHUMT00000366972; OTTHUMT00000367013; uc003vsy.3; uc003vsz.4 | *C7orf73; SLC13A4* | chromosome 7 open reading frame 73; solute carrier family 13 (sodium/sulfate symporter), member 4 | 6.83 | 6.65 | 1.13 | 0.0396 |
| TC19001393.hg.1 | NM_032816; ENST00000305768; AK001375; AK027546; AL832158; BC020195; BC032307; BC136328; uc002ntx.3; uc002nty.3; uc002nua.3; uc002nub.1; uc010edg.3 | *CEP89* | centrosomal protein 89kDa | 4.72 | 4.8 | -1.06 | 0.0396 |
| TC05001784.hg.1 | NM_020199; ENST00000231512; ENST00000507191; BC020875; OTTHUMT00000251175; OTTHUMT00000371472; OTTHUMT00000371473; uc003kyo.3 | *C5orf15* | chromosome 5 open reading frame 15 | 5.39 | 5.43 | -1.03 | 0.0398 |
| TC15001449.hg.1 | NM_138792; ENST00000299601; ENST00000315141; AK055762; BC018147; OTTHUMT00000254791; uc002abo.3; uc010bfd.3 | *LEO1* | LEO1 homolog, Paf1/RNA polymerase II complex component | 5.97 | 6.09 | -1.08 | 0.0398 |
| TC16001919.hg.1 | AL110259 | *LOC101927650* | uncharacterized LOC101927650 | 1.77 | 1.69 | 1.06 | 0.0398 |
| TC6_mann_hap4000215.hg.1 | NM_000247; NM_001177519; BC016929; NR_036523; NR_036524; OTTHUMT00000311095; OTTHUMT00000311096; OTTHUMT00000311097; OTTHUMT00000311098; OTTHUMT00000311099; OTTHUMT00000311100; uc021zpf.1 | *MICA* | MHC class I polypeptide-related sequence A | 5.61 | 5.48 | 1.09 | 0.0398 |
| TC13000264.hg.1 | NR_002717; OTTHUMT00000045232; OTTHUMT00000045233; uc010aej.1 | *ATXN8OS* | ATXN8 opposite strand (non-protein coding) | 2.05 | 1.98 | 1.05 | 0.0399 |
| TC0Y000179.hg.1 | NM_001001877; NM_033108; NM_152584; NM_153716; NR_003509; NR_003510; ENST00000304790; ENST00000344884; ENST00000382852; ENST00000491902; ENST00000505047; BC036567; BC055414; BC117380; OTTHUMT00000100000; OTTHUMT00000100001; OTTHUMT00000100002; OTTHUMT00000100003; uc004ftu.3; uc004ftv.3; uc004ftw.3 | *HSFY2; HSFY1* | heat shock transcription factor, Y-linked 2; heat shock transcription factor, Y-linked 1 | 1.55 | 1.53 | 1.02 | 0.0399 |
| TC03003263.hg.1 | BC042414 | *LPP-AS2* | LPP antisense RNA 2 | 3.61 | 3.46 | 1.11 | 0.0399 |
| TC19000193.hg.1 | NM_012466; ENST00000316737; ENST00000337994; BC029908; uc002mqu.1; uc002mqv.1 | *TSPAN16* | tetraspanin 16 | 4.32 | 4.18 | 1.1 | 0.0399 |
| TC08001575.hg.1 | NM_001134671; NM_024295; ENST00000259512; ENST00000405944; ENST00000419562; ENST00000519018; ENST00000523036; AK023846; AK303826; BC002457; OTTHUMT00000381714; OTTHUMT00000381715; OTTHUMT00000381716; OTTHUMT00000381717; OTTHUMT00000381718; OTTHUMT00000381719 | *DERL1* | derlin 1 | 6.08 | 6.18 | -1.07 | 0.04 |
| TC19001644.hg.1 | BC062328 | *LOC645553* | uncharacterized LOC645553 | 2.16 | 2.35 | -1.14 | 0.04 |
| TC01002391.hg.1 | AL050085 | *LOC101928324* | uncharacterized LOC101928324 | 4.88 | 4.69 | 1.14 | 0.04 |
| TC13001322.hg.1 | NR_024609 | *LINC00452* | long intergenic non-protein coding RNA 452 | 4.84 | 4.91 | -1.05 | 0.0401 |
| TC15001248.hg.1 | NM_007280; ENST00000220514; BC015050; OTTHUMT00000252576; uc001znp.3 | *OIP5* | Opa interacting protein 5 | 2.49 | 2.4 | 1.06 | 0.0401 |
| TC06000360.hg.1 | FM177967 | *POU5F1* | POU class 5 homeobox 1 | 6.12 | 6.02 | 1.08 | 0.0401 |
| TC6_mann_hap4000051.hg.1 | FM177967 | *POU5F1* | POU class 5 homeobox 1 | 6.12 | 6.02 | 1.08 | 0.0401 |
| TC6_mcf_hap5000044.hg.1 | FM177967 | *POU5F1* | POU class 5 homeobox 1 | 6.12 | 6.02 | 1.08 | 0.0401 |
| TC6_qbl_hap6000050.hg.1 | FM177967 | *POU5F1* | POU class 5 homeobox 1 | 6.12 | 6.02 | 1.08 | 0.0401 |
| TC6_ssto_hap7000044.hg.1 | FM177967 | *POU5F1* | POU class 5 homeobox 1 | 6.12 | 6.02 | 1.08 | 0.0401 |
| TC01003615.hg.1 | NR_040063; uc001gqe.3 | *SMG7-AS1* | SMG7 antisense RNA 1 | 2.48 | 2.39 | 1.07 | 0.0401 |
| TC22000393.hg.1 | NM_017931; ENST00000381031; AK300606; BC018918; OTTHUMT00000318469; OTTHUMT00000318470; OTTHUMT00000318471; OTTHUMT00000318472; OTTHUMT00000318473; OTTHUMT00000318474; uc003bhi.3; uc011aqx.2 | *TTC38* | tetratricopeptide repeat domain 38 | 5.84 | 5.95 | -1.08 | 0.0401 |
| TC20000474.hg.1 | NM_021810; NM_177980; ENST00000244049; ENST00000348616; ENST00000350849; ENST00000497614; AF169690; BC062570; BC136306; BC143931; OTTHUMT00000079933; OTTHUMT00000079934; OTTHUMT00000079935; OTTHUMT00000079936; OTTHUMT00000079937; OTTHUMT00000079938; OTTHUMT00000268141; uc002ybe.3; uc002ybf.1; uc002ybh.3; uc002ybi.3; uc010zzy.2 | *CDH26* | cadherin 26 | 3.93 | 3.87 | 1.04 | 0.0402 |
| TC14002201.hg.1 | NM_033426; ENST00000361786; ENST00000555437; ENST00000555611; AK096603; BC037300 | *CIPC* | CLOCK-interacting pacemaker | 6.24 | 6.27 | -1.02 | 0.0402 |
| TC13000189.hg.1 | NM_021999; ENST00000378549; ENST00000378565; ENST00000463839; BC000554; BC016148; OTTHUMT00000044870; OTTHUMT00000044871; OTTHUMT00000044872; uc001vbz.3 | *ITM2B* | integral membrane protein 2B | 9.97 | 9.85 | 1.08 | 0.0403 |
| TC05001731.hg.1 | NM_020747; ENST00000306315; ENST00000504926; ENST00000513985; AK056247; AL117587; BC103742; BC131507; BC151226; OTTHUMT00000371300; OTTHUMT00000371301; OTTHUMT00000371302; OTTHUMT00000371303; OTTHUMT00000371304; OTTHUMT00000371305; OTTHUMT00000371306; OTTHUMT00000371307; OTTHUMT00000371308; uc003ktp.1; uc003ktq.1; uc003ktr.1; uc003kts.1; uc003ktt.1 | *ZNF608* | zinc finger protein 608 | 4.45 | 4.35 | 1.07 | 0.0403 |
| TC02002931.hg.1 | NM_001080835; ENST00000408934; OTTHUMT00000353696; uc010zod.2 | *PRR21* | proline rich 21 | 4.32 | 4.52 | -1.15 | 0.0404 |
| TC08001496.hg.1 | NM_001172477; NM_001172478; NM_015713; ENST00000251810; ENST00000395912; ENST00000519962; ENST00000522394; AB163438; AB166669; AB166670; BC108261; BC117496; BC130628; CR627376; OTTHUMT00000380191; OTTHUMT00000380192; OTTHUMT00000380193; OTTHUMT00000380194; OTTHUMT00000380304; OTTHUMT00000380305; OTTHUMT00000380306; OTTHUMT00000380307; OTTHUMT00000380308; OTTHUMT00000380326; uc003ykn.3; uc003yko.3; uc010mbv.2; uc010mbw.1; uc010mbx.1; uc010mby.1; uc022azl.1 | *RRM2B* | ribonucleotide reductase M2 B (TP53 inducible) | 6.18 | 6.08 | 1.07 | 0.0404 |
| TC14000345.hg.1 | NM_018477; ENST00000254286; ENST00000554402; AK022248; BC011997; BX161491; BX248282; uc001xdf.3; uc010apc.3; uc010trp.2; uc021rtr.1 | *ACTR10* | actin-related protein 10 homolog (S. cerevisiae) | 5.59 | 5.54 | 1.04 | 0.0405 |
| TC10001217.hg.1 | NM_001039380; BC130369; OTTHUMT00000047763; uc001jbv.2 | *C10orf25* | chromosome 10 open reading frame 25 | 4.13 | 4.23 | -1.08 | 0.0405 |
| TC08002582.hg.1 | NM_032664; ENST00000327671; ENST00000518076; ENST00000518672; ENST00000524021; AJ431184; AJ512465; AJ535838; AJ535839; AK297132; AK303231; BC004884; BC063462; OTTHUMT00000376540; OTTHUMT00000376541; OTTHUMT00000376542; OTTHUMT00000376543; OTTHUMT00000376545; OTTHUMT00000376546; OTTHUMT00000376548; OTTHUMT00000376549; OTTHUMT00000376550; OTTHUMT00000376551; OTTHUMT00000376688; uc003xjc.3; uc003xjd.3; uc003xje.3; uc003xjf.3; uc003xjg.3; uc003xjh.3; uc003xji.1; uc011lbi.2 | *FUT10* | fucosyltransferase 10 (alpha (1,3) fucosyltransferase) | 4.32 | 4.55 | -1.17 | 0.0405 |
| TC15002174.hg.1 | NR_027513 | *CCNDBP1* | cyclin D-type binding-protein 1 | 7.57 | 7.31 | 1.2 | 0.0406 |
| TC01003057.hg.1 | NM_001004340; NM_001017986; NM_001244910; NR_045213; ENST00000369383; ENST00000369384; ENST00000472543; AK308886; OTTHUMT00000098241; OTTHUMT00000098242; OTTHUMT00000098243; OTTHUMT00000098244; OTTHUMT00000098245; OTTHUMT00000098246; uc001eip.3; uc001eiq.3; uc009whr.2; uc009whs.2; uc010oxl.2 | *FCGR1B* | Fc fragment of IgG, high affinity Ib, receptor (CD64) | 7.04 | 6.68 | 1.29 | 0.0406 |
| TC09000760.hg.1 | NM_012204; ENST00000372146; BC060821; BC094774; BC104755; OTTHUMT00000054792; OTTHUMT00000054793; uc010mzv.3; uc010mzw.3 | *GTF3C4* | general transcription factor IIIC subunit 4 | 5.2 | 5.26 | -1.05 | 0.0407 |
| TC19000817.hg.1 | NR_031568; ENST00000408090; uc021uzl.1 | *MIR1323* | microRNA 1323 | 2.43 | 2.56 | -1.09 | 0.0407 |
| TC15002607.hg.1 | BC039397 | *MYO9A* | myosin IXA | 3.39 | 3.56 | -1.13 | 0.0407 |
| TC08000546.hg.1 | NM_001126111; NM_004337; ENST00000297438; ENST00000451899; ENST00000520659; OTTHUMT00000375691; OTTHUMT00000375692; OTTHUMT00000375693; uc003yeg.3; uc003yeh.3 | *OSGIN2* | oxidative stress induced growth inhibitor family member 2 | 5.88 | 5.71 | 1.13 | 0.0407 |
| TC04000962.hg.1 | NM_001128325; NM_001199021; NM_012445; ENST00000290902; ENST00000400762; ENST00000431380; AK024499; BC002707; BC036341; OTTHUMT00000202080; OTTHUMT00000359490; OTTHUMT00000359491; OTTHUMT00000359492; OTTHUMT00000359493; OTTHUMT00000359494; OTTHUMT00000359495; OTTHUMT00000359496; OTTHUMT00000359497; OTTHUMT00000359498; OTTHUMT00000359499; OTTHUMT00000359500; OTTHUMT00000359501; OTTHUMT00000359502; OTTHUMT00000359503; OTTHUMT00000359504; OTTHUMT00000359505; OTTHUMT00000359506; OTTHUMT00000359507; OTTHUMT00000359508; uc003gcm.1; uc003gco.4; uc010ibr.3; uc021xkj.1 | *SPON2* | spondin 2, extracellular matrix protein | 5.9 | 6 | -1.07 | 0.0407 |
| TC02002070.hg.1 | ENST00000365142 | *RNY4P15* | RNA, Ro-associated Y4 pseudogene 15 | 3.82 | 3.63 | 1.14 | 0.0408 |
| TC08000902.hg.1 | NM_001118887; NM_001118888; NM_001147; ENST00000325203; ENST00000338312; ENST00000523120; AK310171; BC126200; BC126202; BC143902; OTTHUMT00000206737; OTTHUMT00000374670; OTTHUMT00000374671; uc003wqj.4; uc003wqk.4; uc003wql.4; uc010lri.3 | *ANGPT2* | angiopoietin 2 | 2.87 | 2.92 | -1.04 | 0.0409 |
| TC03000003.hg.1 | ENST00000420823; AK126307 | *LINC01266* | long intergenic non-protein coding RNA 1266 | 1.93 | 1.9 | 1.03 | 0.0409 |
| TC22000345.hg.1 | NM_024821; ENST00000255784; ENST00000402061; AK299311; BC017693; OTTHUMT00000321964; OTTHUMT00000321966; uc003bbh.1; uc011apg.1 | *CCDC134* | coiled-coil domain containing 134 | 6.21 | 6.27 | -1.05 | 0.041 |
| TC0X000538.hg.1 | NM_017698; NM_032227; ENST00000288381; ENST00000372068; ENST00000372072; ENST00000372073; ENST00000464177; AK026332; BC085605; OTTHUMT00000057896; OTTHUMT00000057898; OTTHUMT00000057899; OTTHUMT00000057900; OTTHUMT00000057901; OTTHUMT00000057902; uc004eom.3; uc004eon.2; uc010npq.3 | *TMEM164* | transmembrane protein 164 | 7.32 | 6.99 | 1.26 | 0.041 |
| TC02004761.hg.1 | AK021632 | *ANKRD44* | ankyrin repeat domain 44 | 8.19 | 8.04 | 1.11 | 0.0411 |
| TC04002187.hg.1 | S47380 | *FGF2* | fibroblast growth factor 2 (basic) | 3.39 | 3.28 | 1.07 | 0.0411 |
| TC19000498.hg.1 | NR_029389; uc021utf.1 | *LOC100134317* | uncharacterized LOC100134317 | 3.91 | 4.05 | -1.1 | 0.0411 |
| TC06003152.hg.1 | AJ420452 | *SNX9* | sorting nexin 9 | 5.83 | 5.63 | 1.15 | 0.0411 |
| TC02005008.hg.1 | OTTHUMT00000332013 | *AC009950.1* |  | 2.77 | 2.47 | 1.23 | 0.0412 |
| TC06000983.hg.1 | NM_000045; NM_001244438; ENST00000356962; ENST00000368087; BC005321; BC020653; BT006741; OTTHUMT00000042223; OTTHUMT00000042224; OTTHUMT00000042225; OTTHUMT00000042226; OTTHUMT00000042228; OTTHUMT00000042229; uc003qco.2; uc003qcp.2; uc010kfm.2 | *ARG1* | arginase 1 | 4.5 | 4.25 | 1.19 | 0.0413 |
| TC10000050.hg.1 | NM_032807; NM_178150; ENST00000362091; ENST00000379999; ENST00000397269; ENST00000470089; AK122753; BC110884; BC113375; BC113377; OTTHUMT00000046588; OTTHUMT00000046589; OTTHUMT00000046590; OTTHUMT00000046591; OTTHUMT00000046592; OTTHUMT00000046594; OTTHUMT00000046595; OTTHUMT00000046596; OTTHUMT00000046597; OTTHUMT00000046599; OTTHUMT00000046600; OTTHUMT00000046601; OTTHUMT00000046602; OTTHUMT00000046603; OTTHUMT00000046604 | *FBXO18* | F-box protein, helicase, 18 | 6.37 | 6.3 | 1.05 | 0.0413 |
| TC03003125.hg.1 | BC040051; NR_034032 | *LOC100289361* | uncharacterized LOC100289361 | 4.62 | 4.51 | 1.08 | 0.0413 |
| TC20000044.hg.1 | NM_024960; NM_153638; NM_153640; ENST00000316562; ENST00000336066; ENST00000497424; AK310190; BC009421; BC107724; OTTHUMT00000077787; OTTHUMT00000077788; OTTHUMT00000077789; OTTHUMT00000077790; OTTHUMT00000077791; OTTHUMT00000077793; uc002wkb.3; uc002wkc.3; uc002wkd.3; uc002wke.3; uc002wkf.3; uc010gbd.1 | *PANK2* | pantothenate kinase 2 | 6.49 | 6.42 | 1.05 | 0.0413 |
| TC01005382.hg.1 | NR_033686; NR_033688 | *PEF1* | penta-EF-hand domain containing 1 | 5.87 | 5.78 | 1.06 | 0.0413 |
| TC16000553.hg.1 | NM_012320; ENST00000219345; ENST00000413021; ENST00000444212; AK127898; AK296263; AK298494; AK300596; BC062605; OTTHUMT00000268888; uc002evr.3; uc002evs.3; uc010vld.2; uc010vle.2; uc010vlf.2 | *PLA2G15* | phospholipase A2, group XV | 5.45 | 5.52 | -1.05 | 0.0413 |
| TC15001666.hg.1 | NM_001145357; NM_001145358; NM_015477; ENST00000360439; ENST00000394947; ENST00000394949; BC066364; BC137098; OTTHUMT00000286469; OTTHUMT00000286470; uc002bai.3; uc002baj.3; uc002bak.4; uc010uml.2 | *SIN3A* | SIN3 transcription regulator family member A | 7.54 | 7.59 | -1.03 | 0.0413 |
| TC08001440.hg.1 | NM_001135733; NM_033285; ENST00000342697; ENST00000448464; BC074813; uc003yhg.3; uc003yhh.3 | *TP53INP1* | tumor protein p53 inducible nuclear protein 1 | 7.43 | 7.2 | 1.17 | 0.0413 |
| TC02002480.hg.1 | NM_004460; ENST00000188790; ENST00000443424; AK297118; AK309260; AL832166; BC026250; OTTHUMT00000332852; OTTHUMT00000332854; OTTHUMT00000332855; OTTHUMT00000332856; OTTHUMT00000332857; OTTHUMT00000332858; OTTHUMT00000332976; OTTHUMT00000332977; OTTHUMT00000332978; OTTHUMT00000332979; OTTHUMT00000332980; OTTHUMT00000332982; OTTHUMT00000338544; uc002ucd.3; uc010fpc.3; uc010fpe.1; uc010zct.2 | *FAP* | fibroblast activation protein alpha | 1.46 | 1.43 | 1.02 | 0.0414 |
| TC10001347.hg.1 | NM_001080449; ENST00000358410; ENST00000399179; ENST00000399180; ENST00000551118; BC028188; BC063664; BC111740; OTTHUMT00000048334; OTTHUMT00000048335; OTTHUMT00000048336 | *DNA2* | DNA replication helicase/nuclease 2 | 3.36 | 3.2 | 1.12 | 0.0415 |
| TC21001069.hg.1 | NM_022136; ENST00000400564; ENST00000400566; ENST00000463807; AF519621; BC029112; OTTHUMT00000157914; OTTHUMT00000157915; OTTHUMT00000157916; OTTHUMT00000157917 | *SAMSN1* | SAM domain, SH3 domain and nuclear localization signals 1 | 5.81 | 5.6 | 1.16 | 0.0415 |
| TC11002196.hg.1 | NM_001144871; ENST00000409977; OTTHUMT00000334162; OTTHUMT00000334163; OTTHUMT00000334164; uc010ruc.1 | *VSTM5* | V-set and transmembrane domain containing 5 | 4.09 | 4.15 | -1.04 | 0.0415 |
| TC17000619.hg.1 | NM_003204; ENST00000357480; ENST00000361665; ENST00000362042; ENST00000536222; AK294553; AK302387; AL833530; BC010623; BX647976; L24123; uc002imz.4; uc002ina.4; uc002inb.4; uc002inc.1; uc010wle.2; uc010wlf.2 | *NFE2L1* | nuclear factor, erythroid 2-like 1 | 6.21 | 6.26 | -1.04 | 0.0416 |
| TC11001195.hg.1 | NM_021978; ENST00000278742; AK302454; BC030532; uc001qfw.3; uc010sca.1 | *ST14* | suppression of tumorigenicity 14 (colon carcinoma) | 5.65 | 5.72 | -1.05 | 0.0416 |
| TC02000826.hg.1 | NM_207364; ENST00000309926; BC105013; BC105041; OTTHUMT00000254552; uc002trv.2 | *GPR148* | G protein-coupled receptor 148 | 2.11 | 1.96 | 1.1 | 0.0417 |
| TC08000459.hg.1 | ENST00000501104 | *LOC101929759* | uncharacterized LOC101929759 | 3.16 | 3 | 1.12 | 0.0417 |
| TC19001242.hg.1 | NM_013447; NM_152916; NM_152917; NM_152918; NM_152919; NM_152920; NM_152921; ENST00000315576; ENST00000360222; ENST00000392962; ENST00000392965; AK024426; AK298690; AK298700; AK301458; BC127004 | *ADGRE2* | adhesion G protein-coupled receptor E2 | 8.91 | 8.58 | 1.26 | 0.0418 |
| TC03001907.hg.1 | NM_022788; NM_176876; ENST00000302632; AK303714; BC017898; uc003eyw.1; uc003eyx.1; uc011boa.2 | *P2RY12* | purinergic receptor P2Y, G-protein coupled, 12 | 2.79 | 2.58 | 1.16 | 0.0418 |
| TC19000585.hg.1 | NM_001815; ENST00000344550; ENST00000357396; BC106728; L00693; OTTHUMT00000316509; OTTHUMT00000316510; OTTHUMT00000316511 | *CEACAM3* | carcinoembryonic antigen-related cell adhesion molecule 3 | 7.31 | 7.15 | 1.12 | 0.0419 |
| TC17002192.hg.1 | AJ308027 | *ORMDL3* | ORMDL sphingolipid biosynthesis regulator 3 | 5.15 | 5.06 | 1.06 | 0.0419 |
| TC19001582.hg.1 | NM_001130014; NM_002781; ENST00000342951; ENST00000366175; ENST00000401992; ENST00000404580; ENST00000407356; ENST00000407568; BC012607; OTTHUMT00000323055; OTTHUMT00000323056; OTTHUMT00000323057; OTTHUMT00000323058; OTTHUMT00000323059; OTTHUMT00000323061; OTTHUMT00000323063; uc002ovu.3; uc002ovx.3 | *PSG5; PSG3* | pregnancy specific beta-1-glycoprotein 5; pregnancy specific beta-1-glycoprotein 3 | 2.29 | 2.23 | 1.05 | 0.0419 |
| TC17001750.hg.1 | NM_016077; ENST00000393038; ENST00000409433; ENST00000470557; AK057033; BC006807; OTTHUMT00000335784; OTTHUMT00000335785; uc002ixs.3; uc002ixt.3 | *PTRH2* | peptidyl-tRNA hydrolase 2 | 5.14 | 5.04 | 1.08 | 0.0419 |
| TC06002306.hg.1 | NM_145169; ENST00000361731; ENST00000487841; BC018969; OTTHUMT00000043060; OTTHUMT00000043061; OTTHUMT00000043062; OTTHUMT00000043063; OTTHUMT00000043064; OTTHUMT00000043065; uc003qux.3 | *SFT2D1* | SFT2 domain containing 1 | 5.35 | 5.25 | 1.07 | 0.0419 |
| TC03000637.hg.1 | NR_024618; uc010hrn.3 | *LOC100129550* | uncharacterized LOC100129550 | 5.95 | 5.75 | 1.15 | 0.042 |
| TC03000793.hg.1 | ENST00000490465 | *LOC100507461* | uncharacterized LOC100507461 | 1.23 | 1.12 | 1.08 | 0.042 |
| TC6_cox_hap2000234.hg.1 | NM_000247; NM_001177519; AK094237; BC016929; NR_036523; NR_036524; OTTHUMT00000076728; OTTHUMT00000108726; OTTHUMT00000108727; OTTHUMT00000314780; OTTHUMT00000314781; OTTHUMT00000314782; uc011fcu.1; uc011fcv.1 | *MICA* | MHC class I polypeptide-related sequence A | 5.5 | 5.41 | 1.07 | 0.042 |
| TC6_ssto_hap7000188.hg.1 | NM_000247; NM_001177519; AK094237; BC016929; NR_036523; NR_036524; OTTHUMT00000089760; OTTHUMT00000089761; OTTHUMT00000258837; OTTHUMT00000315402; OTTHUMT00000315403; OTTHUMT00000315404; uc011jin.1; uc011jip.1 | *MICA* | MHC class I polypeptide-related sequence A | 5.5 | 5.41 | 1.07 | 0.042 |
| TC06002285.hg.1 | NR_024277; uc003qtu.4 | *AGPAT4-IT1* | AGPAT4 intronic transcript 1 | 4.64 | 4.43 | 1.16 | 0.0421 |
| TC01000738.hg.1 | NM_001077700; NM_001077701; NM_001077702; NM_001077703; NM_001077704; NM_001146110; NM_001146111; NM_001146112; NM_001146113; NM_020948; ENST00000355356; ENST00000355977; ENST00000357692; ENST00000371012; ENST00000371014; ENST00000371016; ENST00000371018; ENST00000401041; ENST00000401042; ENST00000479067; BC066898; BC108726; BC125217; OTTHUMT00000025490; OTTHUMT00000025491; OTTHUMT00000025938; OTTHUMT00000025939; OTTHUMT00000025940; OTTHUMT00000025941; OTTHUMT00000025942; OTTHUMT00000025943; OTTHUMT00000025944; uc001ddc.2; uc001dde.2; uc001ddf.2; uc001ddg.2; uc001ddh.2; uc001ddi.2; uc001ddj.1; uc009way.2; uc010opf.1; uc010opg.1 | *MIER1* | mesoderm induction early response 1, transcriptional regulator | 6.43 | 6.34 | 1.06 | 0.0421 |
| TC04001066.hg.1 | NM_000320; ENST00000281243; ENST00000428702; ENST00000508623; ENST00000513615; AK124382; AK296622; BC000576; OTTHUMT00000250372; OTTHUMT00000359705; OTTHUMT00000359706; OTTHUMT00000359708; OTTHUMT00000359709; OTTHUMT00000359992; OTTHUMT00000359993; OTTHUMT00000359994; OTTHUMT00000359995; uc003gpd.3; uc003gpe.3; uc021xmo.1 | *QDPR* | quinoid dihydropteridine reductase | 4.67 | 4.71 | -1.03 | 0.0421 |
| TC07001844.hg.1 | NM_001166135; NM_018077; ENST00000223073; ENST00000415472; ENST00000481788; BC013889; OTTHUMT00000349442; OTTHUMT00000349443; OTTHUMT00000349444; OTTHUMT00000349445; OTTHUMT00000349446; OTTHUMT00000349447; OTTHUMT00000349448; OTTHUMT00000349449; uc003vmp.2; uc011koj.1 | *RBM28* | RNA binding motif protein 28 | 5.2 | 5.29 | -1.06 | 0.0421 |
| TC04001854.hg.1 | AL390128 | *UVSSA* | UV stimulated scaffold protein A | 5.88 | 5.98 | -1.07 | 0.0421 |
| TC12000089.hg.1 | NM_001164093; NM_001164094; NM_001164095; NM_016319; ENST00000229251; ENST00000534877; ENST00000534947; ENST00000536872; ENST00000538410; ENST00000539735; ENST00000543155; BC011789; BC093015; uc001qqh.3; uc001qqi.3; uc001qqj.3; uc001qqn.4 | *COPS7A* | COP9 signalosome subunit 7A | 6.57 | 6.62 | -1.03 | 0.0422 |
| TC17000829.hg.1 | NM_181790; ENST00000335666; uc010wqy.2; uc021ucp.1 | *GPR142* | G protein-coupled receptor 142 | 4.58 | 4.66 | -1.06 | 0.0422 |
| TC01001161.hg.1 | NR_027484; ENST00000453173; uc010pbh.2 | *FCGR1C; FCGR1B* | Fc fragment of IgG, high affinity Ic, receptor (CD64), pseudogene; Fc fragment of IgG, high affinity Ib, receptor (CD64) | 6.87 | 6.49 | 1.3 | 0.0423 |
| TC15001352.hg.1 | NR_026891; uc001zyg.3 | *FLJ10038* | uncharacterized protein FLJ10038 | 5.92 | 5.63 | 1.22 | 0.0423 |
| TC14001006.hg.1 | NR_038356; ENST00000468444; ENST00000555108; ENST00000555421; ENST00000556786; uc001wqs.3 | *LOC100506071* | uncharacterized LOC100506071 | 2.8 | 2.64 | 1.11 | 0.0423 |
| TC01003990.hg.1 | NR_002956; ENST00000384452; uc001hwm.1 | *SNORA14B* | small nucleolar RNA, H/ACA box 14B | 4.06 | 3.75 | 1.24 | 0.0423 |
| TC01004629.hg.1 | AJ227918 | *CAPZA1* | capping protein (actin filament) muscle Z-line, alpha 1 | 9.24 | 9.04 | 1.15 | 0.0424 |
| TC06002570.hg.1 | AY914050 | *HULC* | hepatocellular carcinoma up-regulated long non-coding RNA | 1.92 | 1.86 | 1.04 | 0.0424 |
| TC03001869.hg.1 | NM_021105; ENST00000342435; ENST00000448787; ENST00000484560; ENST00000487389; AK300181; BC017901; BC021100; BC032718; BC070251; OTTHUMT00000355257; OTTHUMT00000355258; OTTHUMT00000355259; OTTHUMT00000355260; OTTHUMT00000355261; OTTHUMT00000355339; OTTHUMT00000355340; OTTHUMT00000355341; OTTHUMT00000355342; OTTHUMT00000355343; OTTHUMT00000355344; OTTHUMT00000355345; OTTHUMT00000355346; OTTHUMT00000355347; OTTHUMT00000355348; OTTHUMT00000355349; OTTHUMT00000355351; OTTHUMT00000355352; OTTHUMT00000355353; uc003evx.4; uc003evz.4; uc003ewa.2; uc011bnn.2 | *PLSCR1* | phospholipid scramblase 1 | 5.62 | 5.27 | 1.28 | 0.0424 |
| TC03001899.hg.1 | NM_014445; ENST00000239944; ENST00000479209; ENST00000487153; ENST00000490945; ENST00000491660; AK125413; BC039424; BC108314; BC112364; OTTHUMT00000357239; OTTHUMT00000357243; OTTHUMT00000357244; OTTHUMT00000357245; OTTHUMT00000357246; OTTHUMT00000357247; OTTHUMT00000357249; uc003exy.3; uc003exz.3 | *SERP1* | stress-associated endoplasmic reticulum protein 1 | 4.93 | 4.86 | 1.05 | 0.0424 |
| TC0X000520.hg.1 | NM_017752; NM_198881; ENST00000276175; ENST00000310452; ENST00000357242; ENST00000481617; BC122564; BX648230; OTTHUMT00000057807; OTTHUMT00000057808; OTTHUMT00000057809; OTTHUMT00000057810; OTTHUMT00000057811; OTTHUMT00000359366; OTTHUMT00000359367; uc004emm.3; uc004emn.3; uc004emo.3 | *TBC1D8B* | TBC1 domain family, member 8B (with GRAM domain) | 1.65 | 1.6 | 1.04 | 0.0424 |
| TC12000076.hg.1 | NM_001769; ENST00000009180; ENST00000382515; ENST00000382518; ENST00000481267; AK296332; AK296894; BC011988; OTTHUMT00000103345; OTTHUMT00000103346; OTTHUMT00000103347; OTTHUMT00000103348; uc001qnq.2; uc010seu.2; uc010sev.2 | *CD9* | CD9 molecule | 5.76 | 5.62 | 1.1 | 0.0425 |
| TC17000834.hg.1 | NM_004252; NR_037409; ENST00000262613; ENST00000413388; AK094467; BC001443; BC003361; BC011777; BC053350; uc002jln.1; uc021ucr.1 | *SLC9A3R1; MIR3615* | solute carrier family 9, subfamily A (NHE3, cation proton antiporter 3), member 3 regulator 1; microRNA 3615 | 8.51 | 8.63 | -1.09 | 0.0425 |
| TC05000840.hg.1 | NM_078483; ENST00000243389; ENST00000429484; ENST00000520701; ENST00000521351; ENST00000521925; AK057340; AK309323; BC136437; OTTHUMT00000252433; OTTHUMT00000373834; OTTHUMT00000373835; OTTHUMT00000373836; OTTHUMT00000373839; OTTHUMT00000373840; OTTHUMT00000373841; OTTHUMT00000373842; OTTHUMT00000373843; OTTHUMT00000373844; uc003lub.1; uc003luc.3; uc010jhw.1 | *SLC36A1* | solute carrier family 36 (proton/amino acid symporter), member 1 | 6.03 | 5.96 | 1.05 | 0.0425 |
| TC17000398.hg.1 | NR_003037; ENST00000384567; uc002hjo.1 | *SNORD7* | small nucleolar RNA, C/D box 7 | 4.72 | 4.59 | 1.09 | 0.0425 |
| TC19001625.hg.1 | NM_001193268; NM_001193269; NM_012155; NR_034098; ENST00000245925; ENST00000399594; ENST00000536630; AK302961; AK304299; AK308403; AK308580; BC032630; uc002pcn.3; uc002pco.3; uc002pcp.3; uc010ekj.3; uc010ekl.3; uc010xxl.2; uc010xxm.2; uc010xxn.1; uc010xxo.2 | *EML2* | echinoderm microtubule associated protein like 2 | 5.43 | 5.46 | -1.03 | 0.0426 |
| TC16001330.hg.1 | NM_024735; ENST00000311635; AF318348; BC012748; NR_024568; uc002fjv.3; uc002fjw.3 | *FBXO31* | F-box protein 31 | 6.08 | 6.1 | -1.02 | 0.0426 |
| TC07000034.hg.1 | NM_001040167; NM_001040168; NM_001166355; NM_002304; NR_039791; ENST00000222725; ENST00000338732; ENST00000359574; ENST00000402045; ENST00000402506; BC014851; OTTHUMT00000325021; OTTHUMT00000325022; OTTHUMT00000325023; OTTHUMT00000325024; OTTHUMT00000325025; uc003smf.3; uc003smg.3; uc021zyw.1; uc021zyx.1; uc021zyy.1 | *LFNG; MIR4648* | LFNG O-fucosylpeptide 3-beta-N-acetylglucosaminyltransferase; microRNA 4648 | 6.38 | 6.46 | -1.06 | 0.0426 |
| TC14001185.hg.1 | BC035195 | *LOC101927702* | uncharacterized LOC101927702 | 4.16 | 4.22 | -1.05 | 0.0426 |
| TC09000620.hg.1 | NM_001005236; ENST00000373686; OTTHUMT00000053949; uc022bmz.1 | *OR1L1* | olfactory receptor, family 1, subfamily L, member 1 | 1.53 | 1.35 | 1.13 | 0.0426 |
| TC16000677.hg.1 | NM_001173539; NM_001173540; NM_001173541; NM_001173542; NM_001173543; NM_017869; NM_079837; ENST00000286122; ENST00000355022; ENST00000355163; ENST00000393207; ENST00000393208; ENST00000479780; ENST00000481948; ENST00000538234; BC009424; BC018247; OTTHUMT00000269165; OTTHUMT00000269166; OTTHUMT00000269167; OTTHUMT00000312872; OTTHUMT00000312873; OTTHUMT00000312874; OTTHUMT00000312876; OTTHUMT00000312877; OTTHUMT00000312878; OTTHUMT00000312879; OTTHUMT00000312880; OTTHUMT00000312881; OTTHUMT00000312882; OTTHUMT00000312883; OTTHUMT00000312884; OTTHUMT00000312886; OTTHUMT00000312887; OTTHUMT00000312888; uc002fko.1; uc002fkp.3; uc002fkq.3; uc002fkr.3; uc002fks.4; uc010vov.2; uc010vow.2; uc021tml.1 | *BANP* | BTG3 associated nuclear protein | 6.5 | 6.47 | 1.02 | 0.0427 |
| TC01003882.hg.1 | NR_031574; ENST00000408479; uc021pjo.1 | *MIR320B2* | microRNA 320b-2 | 1.17 | 1.1 | 1.05 | 0.0427 |
| TC10000679.hg.1 | NM_001134375; NM_001134376; NM_019084; ENST00000265992; ENST00000403870; BC043175; OTTHUMT00000090166; uc001klm.3; uc001kln.3; uc010qoq.2 | *CCNJ* | cyclin J | 4.94 | 4.85 | 1.07 | 0.0428 |
| TC10001366.hg.1 | NM_001083116; NM_005041; ENST00000373209; ENST00000441259; BC047695; BC063043; OTTHUMT00000048517; uc001jrf.4; uc009xqg.3 | *PRF1* | perforin 1 (pore forming protein) | 8.61 | 9.01 | -1.32 | 0.0428 |
| TC0X001551.hg.1 | NM_001289; ENST00000369449; ENST00000465553; AY191592; BC022305; OTTHUMT00000058793; OTTHUMT00000058794; OTTHUMT00000058795; OTTHUMT00000316587; uc004fnf.3; uc010nvj.1 | *CLIC2* | chloride intracellular channel 2 | 4.58 | 4.28 | 1.23 | 0.043 |
| TC04000989.hg.1 | NM_001145725; NM_017816; ENST00000343470; ENST00000452476; BC015796; OTTHUMT00000246800; OTTHUMT00000358646; OTTHUMT00000359601; uc003ght.3; uc011bvy.2 | *LYAR* | Ly1 antibody reactive | 5.16 | 5.25 | -1.06 | 0.043 |
| TC12001718.hg.1 | NM_001109754; NM_001206971; NM_001206972; NM_002837; ENST00000261266; ENST00000334414; ENST00000451516; ENST00000538174; ENST00000538708; ENST00000550358; ENST00000550857; ENST00000551525; AK126121; AK128562; BC101679; BC113463; BC143356; BC143360; BX647238; CR749546; uc001swa.4; uc001swb.4; uc001swc.4; uc001swd.4; uc001swe.3; uc009zrr.2; uc010sto.2; uc010stp.2 | *PTPRB* | protein tyrosine phosphatase, receptor type, B | 2.42 | 2.48 | -1.04 | 0.0431 |
| TC01001619.hg.1 | NM_130782; ENST00000367460; ENST00000481707; BC020632; OTTHUMT00000086382; OTTHUMT00000086383; OTTHUMT00000086384; OTTHUMT00000086385; uc001gsg.3 | *RGS18* | regulator of G-protein signaling 18 | 8.33 | 8.05 | 1.22 | 0.0431 |
| TC01000813.hg.1 | AF306347; BC064144; BC119642; BC127634; uc001djx.3; uc001djy.2; uc021opb.1 | *SPATA1* | spermatogenesis associated 1 | 2.76 | 2.72 | 1.03 | 0.0431 |
| TC06001340.hg.1 | NM_005325; ENST00000244573; BC069492; BC101593; BC112140; OTTHUMT00000043884; uc003nfo.3 | *HIST1H1A* | histone cluster 1, H1a | 2.9 | 2.83 | 1.05 | 0.0432 |
| TC09001210.hg.1 | ENST00000423171; ENST00000449235; ENST00000453787 | *LINC01474* | long intergenic non-protein coding RNA 1474 | 2.53 | 2.39 | 1.1 | 0.0432 |
| TC01005461.hg.1 | BC006113 | *FOXD2-AS1* | FOXD2 antisense RNA 1 (head to head) | 5.38 | 5.55 | -1.12 | 0.0433 |
| TC13000179.hg.1 | ENST00000446175; BC030276; OTTHUMT00000044802; uc001vbc.3 | *LRRC63* | leucine rich repeat containing 63 | 1.66 | 1.56 | 1.07 | 0.0433 |
| TC16000477.hg.1 | NM_001242795; NM_001242796; NM_014669; ENST00000308159; ENST00000542526; BC034346; OTTHUMT00000257058; uc002eka.3; uc002ekb.3; uc010vhi.2 | *NUP93* | nucleoporin 93kDa | 5.69 | 5.79 | -1.07 | 0.0433 |
| TC17000064.hg.1 | NR_034082; AK021878; uc002gbg.2 | *LOC100130950* | uncharacterized LOC100130950 | 4.56 | 4.63 | -1.05 | 0.0434 |
| TC20000762.hg.1 | NM_002657; ENST00000246229; BC023655; OTTHUMT00000078615; uc002wxn.2 | *PLAGL2* | pleiomorphic adenoma gene-like 2 | 7.19 | 7.31 | -1.08 | 0.0434 |
| TC11001244.hg.1 | NM_021008; ENST00000382409; ENST00000525904; AB209831; BC053322; FJ985253; uc001lqq.1; uc009ycf.1; uc021qbn.1 | *DEAF1* | DEAF1 transcription factor | 6.45 | 6.39 | 1.04 | 0.0435 |
| TC12001453.hg.1 | NR_002968; NR_031623; ENST00000408564 | *SNORA2C; MIR1291* | small nucleolar RNA, H/ACA box 2C; microRNA 1291 | 7.32 | 6.83 | 1.4 | 0.0435 |
| TC17000500.hg.1 | NM_001195386; NM_001195387; NM_145274; ENST00000301665; ENST00000496847; BC015365; OTTHUMT00000257681; OTTHUMT00000257682; OTTHUMT00000257683; uc002hvj.1; uc021txc.1; uc021txd.1 | *TMEM99* | transmembrane protein 99 | 3.09 | 2.99 | 1.07 | 0.0435 |
| TC04000581.hg.1 | NM_001102406; NM_001253884; NM_025144; ENST00000177648; ENST00000458497; ENST00000504176; ENST00000505912; AK097246; AK131090; AK303489; BC028117; OTTHUMT00000256421; OTTHUMT00000363624; OTTHUMT00000363625; OTTHUMT00000363627; OTTHUMT00000363628; OTTHUMT00000363629; OTTHUMT00000363630; OTTHUMT00000363631; OTTHUMT00000363632; OTTHUMT00000363633; OTTHUMT00000363634; OTTHUMT00000363635; OTTHUMT00000363636; OTTHUMT00000363637; OTTHUMT00000363638; OTTHUMT00000363639; uc003iam.3; uc003ian.4; uc003iao.4; uc003iap.4; uc010imo.3; uc011cfw.1; uc011cfx.2 | *ALPK1* | alpha kinase 1 | 6.59 | 6.37 | 1.17 | 0.0437 |
| TC12003152.hg.1 | ENST00000541348; ENST00000544727 | *LOC440117* | uncharacterized LOC440117 | 2.36 | 2.43 | -1.05 | 0.0437 |
| TC01000491.hg.1 | NM_001136275; NM_024595; ENST00000372984; ENST00000432648; ENST00000446189; AK298890; BC119745; OTTHUMT00000019687; uc001ccw.3; uc010oip.2; uc010oiq.2 | *AKIRIN1* | akirin 1 | 7.37 | 7.31 | 1.04 | 0.0438 |
| TC12001774.hg.1 | NR_030349; ENST00000385287; uc021rbc.1 | *MIR618* | microRNA 618 | 4.14 | 3.79 | 1.28 | 0.0438 |
| TC13000383.hg.1 | NM_001145645; NM_006573; ENST00000375887; ENST00000430559; ENST00000479435; ENST00000542136; BC020674; DQ857727; OTTHUMT00000045739; OTTHUMT00000045740; OTTHUMT00000045741; OTTHUMT00000045742; uc001vqr.3; uc010agj.3 | *TNFSF13B* | tumor necrosis factor (ligand) superfamily, member 13b | 6.63 | 6.38 | 1.19 | 0.0438 |
| TC22000311.hg.1 | NM_001166002; NM_001166003; NM_001166004; NM_181773; ENST00000348946; ENST00000401756; ENST00000421988; ENST00000442487; BC069023; OTTHUMT00000321229; OTTHUMT00000321230; OTTHUMT00000321568; uc021wps.1; uc021wpt.1; uc021wpu.1; uc021wpv.1 | *APOBEC3H* | apolipoprotein B mRNA editing enzyme, catalytic polypeptide-like 3H | 3.58 | 3.64 | -1.04 | 0.0439 |
| TC03000722.hg.1 | NM_001134422; NM_001134423; NM_017548; ENST00000264993; ENST00000420115; ENST00000431519; ENST00000508481; ENST00000511392; ENST00000515421; BC007338; OTTHUMT00000357203; OTTHUMT00000357204; OTTHUMT00000357205; OTTHUMT00000357206; OTTHUMT00000357207; OTTHUMT00000357208; OTTHUMT00000357209; OTTHUMT00000357210; OTTHUMT00000357211; uc003epp.4; uc003epq.3; uc003epr.3 | *CDV3* | CDV3 homolog (mouse) | 7.92 | 7.96 | -1.03 | 0.0439 |
| TC01006312.hg.1 | NM_005747; ENST00000290122; ENST00000374663; BC005918; BC007028; BC008383; BC015103; OTTHUMT00000007791; OTTHUMT00000007793; OTTHUMT00000096533; uc001bfl.3 | *CELA3A* | chymotrypsin-like elastase family, member 3A | 4.66 | 4.72 | -1.04 | 0.0439 |
| TC0X001829.hg.1 | AY007113 | *AMMECR1* | Alport syndrome, mental retardation, midface hypoplasia and elliptocytosis chromosomal region gene 1 | 5.37 | 4.78 | 1.51 | 0.044 |
| TC07001649.hg.1 | NM_001202855; NM_017460; ENST00000336411; ENST00000354593; AK298451; BC069418; BC101631; OTTHUMT00000345059; OTTHUMT00000345060; OTTHUMT00000345061; uc003urv.2; uc003urw.2; uc011kiz.2 | *CYP3A4* | cytochrome P450, family 3, subfamily A, polypeptide 4 | 2.06 | 2.01 | 1.04 | 0.044 |
| TC05000731.hg.1 | NM_017706; ENST00000358337; ENST00000520764; AK293417; BC068485; OTTHUMT00000251680; OTTHUMT00000372888; OTTHUMT00000372889; OTTHUMT00000372890; OTTHUMT00000374268; uc003lgr.4; uc011czl.1 | *WDR55* | WD repeat domain 55 | 5.81 | 5.87 | -1.05 | 0.044 |
| TC16001019.hg.1 | NM_001145524; NM_031477; ENST00000398838; ENST00000398841; AK022409; BC005009; BC050664; uc002dwl.3; uc002dwm.3; uc002dwn.1 | *YPEL3* | yippee like 3 | 7.69 | 7.54 | 1.11 | 0.044 |
| TC03002458.hg.1 | NR_033977 | *FLJ22763* | uncharacterized LOC401081 | 2.17 | 2.36 | -1.14 | 0.0441 |
| TC06002966.hg.1 | AJ431619 | *LOC105377925* | uncharacterized LOC105377925 | 1.03 | 0.97 | 1.05 | 0.0441 |
| TC02000582.hg.1 | NM_015341; ENST00000240423; ENST00000427946; ENST00000455200; AK299248; AK303725; AK308050; AK310649; BC024211; OTTHUMT00000252842; OTTHUMT00000338872; OTTHUMT00000338873; OTTHUMT00000338874; OTTHUMT00000338875; OTTHUMT00000338876; OTTHUMT00000338877; uc002svz.1; uc010fhu.1; uc010fhv.1; uc010yum.1; uc010yun.1 | *NCAPH* | non-SMC condensin I complex subunit H | 3.82 | 3.69 | 1.1 | 0.0442 |
| TC20000072.hg.1 | NM_015192; NM_182734; ENST00000338037; ENST00000378637; ENST00000378641; ENST00000494924; AK023689; AK127693; AK299195; AK310597; BC069420; BC117231; OTTHUMT00000077936; OTTHUMT00000077937; OTTHUMT00000077938; OTTHUMT00000077939; OTTHUMT00000077940; OTTHUMT00000077941; OTTHUMT00000077942; OTTHUMT00000077943; OTTHUMT00000077944; uc002wnc.1; uc002wnd.1; uc010zrb.1 | *PLCB1* | phospholipase C, beta 1 (phosphoinositide-specific) | 5.46 | 5.59 | -1.09 | 0.0442 |
| TC0X002329.hg.1 | NM_001037735; NM_001190255; ENST00000276054; ENST00000409324; ENST00000442455; BC112139; NR_033730; OTTHUMT00000056456; OTTHUMT00000327254; OTTHUMT00000327255; OTTHUMT00000327257; uc004div.4; uc022bvr.1 | *ZNF630* | zinc finger protein 630 | 4.17 | 4.02 | 1.1 | 0.0442 |
| TC17001137.hg.1 | NM_001004313; ENST00000341871; ENST00000455996; BC127705; BC136838; BC136840; EF126147; uc002gmx.3; uc002gmy.3 | *TMEM220* | transmembrane protein 220 | 3.8 | 3.74 | 1.04 | 0.0443 |
| TC07001241.hg.1 | NR_027347 | *ZNRF2P2* | zinc and ring finger 2 pseudogene 2 | 5.63 | 5.69 | -1.04 | 0.0443 |
| TC10000813.hg.1 | NM_016234; NM_203379; NM_203380; ENST00000354273; ENST00000354655; ENST00000356116; ENST00000393081; ENST00000433418; ENST00000479936; AK301786; AM262166; BC007985; OTTHUMT00000050386; OTTHUMT00000050387; OTTHUMT00000050388; OTTHUMT00000050389; OTTHUMT00000050390; OTTHUMT00000050391; uc001kzs.3; uc001kzt.3; uc001kzu.3; uc009xxz.3; uc010qrj.2 | *ACSL5* | acyl-CoA synthetase long-chain family member 5 | 6.32 | 6.47 | -1.11 | 0.0444 |
| TC06000960.hg.1 | NM_012259; ENST00000368364; ENST00000368365; AK303890; BC007707; OTTHUMT00000042077; OTTHUMT00000042078; uc003qad.3; uc011ebr.2 | *HEY2* | hes-related family bHLH transcription factor with YRPW motif 2 | 4.2 | 4.3 | -1.08 | 0.0444 |
| TC16000592.hg.1 | NM_001166395; NM_005769; ENST00000338482; ENST00000539698; BC035282; OTTHUMT00000268992; uc002fan.3; uc002fao.3 | *CHST4* | carbohydrate (N-acetylglucosamine 6-O) sulfotransferase 4 | 3.79 | 3.9 | -1.08 | 0.0446 |
| TC03001445.hg.1 | NM_001947; ENST00000495880; BC104880; BC128064; OTTHUMT00000349697; OTTHUMT00000349698; uc003dct.3; uc010hma.2 | *DUSP7* | dual specificity phosphatase 7 | 7.16 | 7.24 | -1.06 | 0.0446 |
| TC08000014.hg.1 | NM_003970; ENST00000262113; ENST00000520298; ENST00000523438; AK297890; BC052969; OTTHUMT00000251249; OTTHUMT00000374603; OTTHUMT00000374605; OTTHUMT00000374606; OTTHUMT00000374607; OTTHUMT00000374608; OTTHUMT00000374609; OTTHUMT00000374610; OTTHUMT00000374611; OTTHUMT00000374612; OTTHUMT00000374613; OTTHUMT00000374614; OTTHUMT00000374615; OTTHUMT00000374616; OTTHUMT00000375059; uc003wpx.4; uc011kwi.2 | *MYOM2* | myomesin 2 | 4.14 | 4.22 | -1.06 | 0.0446 |
| TC05000472.hg.1 | NM_005575; NM_175920; ENST00000231368; ENST00000395770; ENST00000395784; OTTHUMT00000250624; OTTHUMT00000317161; OTTHUMT00000317164; OTTHUMT00000317165; OTTHUMT00000370863; uc003kmv.1; uc003kmw.1 | *LNPEP* | leucyl/cystinyl aminopeptidase | 6.73 | 6.89 | -1.12 | 0.0447 |
| TC05001788.hg.1 | NM_080656; ENST00000395009; ENST00000458198; BC008293; BC018086; OTTHUMT00000251171; OTTHUMT00000371547; uc011cxs.2 | *CDKN2AIPNL* | CDKN2A interacting protein N-terminal like | 4.23 | 4.29 | -1.04 | 0.0448 |
| TC06001714.hg.1 | NM_024807; ENST00000483722; BC125078; OTTHUMT00000043756; uc010jxm.1 | *TREML2* | triggering receptor expressed on myeloid cells-like 2 | 7.93 | 7.73 | 1.14 | 0.0448 |
| TC19000955.hg.1 | NM_001164527; NM_001164529; NM_001164530; NM_003436; NM_007134; ENST00000313434; ENST00000359978; ENST00000401053; ENST00000506786; ENST00000511556; ENST00000515535; AK094202; AK297226; BC046434; OTTHUMT00000361897; OTTHUMT00000361899; OTTHUMT00000361900; OTTHUMT00000361901; OTTHUMT00000361902; uc002qrd.2; uc002qre.3; uc002qrf.3; uc002qrg.3; uc010yhq.2; uc010yhr.2; uc021vct.1; uc021vcu.1 | *ZNF135* | zinc finger protein 135 | 4.66 | 4.8 | -1.1 | 0.0448 |
| TC02004990.hg.1 | NM_144706; ENST00000302513; ENST00000409684; BC021264; OTTHUMT00000253122; OTTHUMT00000329945; OTTHUMT00000329946; uc002szk.3 | *C2orf15* | chromosome 2 open reading frame 15 | 2.36 | 2.44 | -1.06 | 0.0449 |
| TC06001216.hg.1 | NM_001012418; ENST00000274643; AK122581; BC132831; OTTHUMT00000039632; uc003mtx.4; uc003mty.4 | *MYLK4* | myosin light chain kinase family member 4 | 3.38 | 3.43 | -1.04 | 0.0449 |
| TC01004072.hg.1 | NM_001004492; ENST00000318749; BC137181; BC137205; OTTHUMT00000097620; uc010pyx.2 | *OR2B11* | olfactory receptor, family 2, subfamily B, member 11 | 2.24 | 2.38 | -1.1 | 0.045 |
| TC08001205.hg.1 | NM_052937; ENST00000360540; ENST00000519559; ENST00000521344; ENST00000522514; ENST00000544451; AK304196; AK304332; BC010693; BC032670; OTTHUMT00000377908; OTTHUMT00000377909; OTTHUMT00000377910; OTTHUMT00000377911; OTTHUMT00000377912; OTTHUMT00000377913; OTTHUMT00000377946; uc003xqx.4; uc010lya.3; uc011ldm.2; uc011ldn.2 | *PCMTD1* | protein-L-isoaspartate (D-aspartate) O-methyltransferase domain containing 1 | 6.65 | 6.53 | 1.09 | 0.045 |
| TC01003383.hg.1 | NM_004833; ENST00000368130; ENST00000481829; BC010940; OTTHUMT00000090341; OTTHUMT00000090342; OTTHUMT00000090343; OTTHUMT00000090344; uc001ftj.1 | *AIM2* | absent in melanoma 2 | 4.88 | 4.67 | 1.15 | 0.0451 |
| TC04001608.hg.1 | NM_014885; ENST00000309439; ENST00000451299; ENST00000507656; ENST00000510270; ENST00000512680; AF132794; AL080090; BC005217; OTTHUMT00000365088; OTTHUMT00000365089; OTTHUMT00000365090; OTTHUMT00000365091; OTTHUMT00000365092; OTTHUMT00000365093; OTTHUMT00000365094; OTTHUMT00000365095; OTTHUMT00000365096; OTTHUMT00000365097; OTTHUMT00000365098; OTTHUMT00000365099; OTTHUMT00000365100; OTTHUMT00000365101; OTTHUMT00000365102; OTTHUMT00000365103 | *ANAPC10* | anaphase promoting complex subunit 10 | 2.65 | 2.58 | 1.05 | 0.0451 |
| TC0X000282.hg.1 | NR_030224; ENST00000385051; uc022bwg.1 | *MIR500A* | microRNA 500a | 4.61 | 4.76 | -1.11 | 0.0451 |
| TC11000459.hg.1 | NM_001005210; ENST00000497933; BC136737; BC136739; OTTHUMT00000354503; uc001njl.2 | *LRRC55* | leucine rich repeat containing 55 | 2.64 | 2.81 | -1.12 | 0.0452 |
| TC01003552.hg.1 | NR_002998; ENST00000517097; uc001gkx.1 | *SCARNA3* | small Cajal body-specific RNA 3 | 5.83 | 5.66 | 1.13 | 0.0452 |
| TC03001575.hg.1 | NM_000158; ENST00000429644; ENST00000477426; ENST00000489715; AK300620; BC012098; OTTHUMT00000352760; OTTHUMT00000352852; OTTHUMT00000352854; OTTHUMT00000352855; OTTHUMT00000352856; OTTHUMT00000352858; uc021xav.1; uc021xax.1 | *GBE1* | glucan (1,4-alpha-), branching enzyme 1 | 4.58 | 4.5 | 1.06 | 0.0453 |
| TC22000334.hg.1 | NM_031488; ENST00000216237; ENST00000489136; AJ305227; AK097052; BC017191; OTTHUMT00000320613; OTTHUMT00000320614; OTTHUMT00000320615; OTTHUMT00000320616; OTTHUMT00000320617; OTTHUMT00000320618; OTTHUMT00000320619; OTTHUMT00000320620; OTTHUMT00000320621; uc003azn.3; uc003azo.3; uc010gyi.1 | *L3MBTL2* | l(3)mbt-like 2 (Drosophila) | 5.74 | 5.84 | -1.07 | 0.0453 |
| TC22000883.hg.1 | BC009388; uc003bgs.3 | *LOC554174* | uncharacterized LOC554174 | 4.59 | 4.71 | -1.09 | 0.0453 |
| TC01003751.hg.1 | NM_022731; ENST00000367142; ENST00000464938; BC000805; OTTHUMT00000087729; OTTHUMT00000087730; uc001hdb.3 | *NUCKS1* | nuclear casein kinase and cyclin-dependent kinase substrate 1 | 6.02 | 6.13 | -1.08 | 0.0453 |
| TC15001596.hg.1 | NR_026808 | *ANP32A-IT1* | ANP32A intronic transcript 1 | 7.25 | 7.11 | 1.11 | 0.0454 |
| TC01002745.hg.1 | NR_037443; uc021ook.1 | *MIR3671* | microRNA 3671 | 8.81 | 8.54 | 1.2 | 0.0454 |
| TC03002161.hg.1 | NM_152617; ENST00000318037; BC017442; BC033791; OTTHUMT00000340778; OTTHUMT00000340779; uc003fwq.3; uc010iah.3 | *RNF168* | ring finger protein 168, E3 ubiquitin protein ligase | 6.14 | 6.23 | -1.06 | 0.0454 |
| TC18000818.hg.1 | uc010wzm.1 | *LOC100192426* | uncharacterized LOC100192426 | 5.44 | 5.51 | -1.06 | 0.0455 |
| TC06002263.hg.1 | NM_006519; ENST00000367085; ENST00000367088; ENST00000367089; BC029412; BC105588; OTTHUMT00000042881; OTTHUMT00000042882; OTTHUMT00000042883; uc003qrn.2 | *DYNLT1* | dynein, light chain, Tctex-type 1 | 6.52 | 6.25 | 1.2 | 0.0456 |
| TC14000034.hg.1 | NM_172194; ENST00000331723; uc010tkt.2 | *OR4Q3* | olfactory receptor, family 4, subfamily Q, member 3 | 1.82 | 1.75 | 1.05 | 0.0456 |
| TC12002824.hg.1 | NR_004854 | *AMN1* | antagonist of mitotic exit network 1 homolog | 5.32 | 5.19 | 1.09 | 0.0457 |
| TC06001193.hg.1 | NM_001029863; ENST00000332290; AK295962; BC051700; OTTHUMT00000043214; uc003qxb.3; uc011egx.2 | *C6orf120* | chromosome 6 open reading frame 120 | 6.03 | 5.95 | 1.06 | 0.0457 |
| TC19001472.hg.1 | NM_001242800; NM_001242801; NM_001242802; NM_206894; ENST00000356725; BC057245; uc002oew.3; uc021utk.1; uc021utl.1; uc021utm.1 | *ZNF790* | zinc finger protein 790 | 4.9 | 4.94 | -1.03 | 0.0457 |
| TC05000186.hg.1 | NM_032587; ENST00000254691; ENST00000381677; BC093825; OTTHUMT00000211584; OTTHUMT00000367557; uc003jmg.3 | *CARD6* | caspase recruitment domain family, member 6 | 5.47 | 5.37 | 1.07 | 0.0458 |
| TC19001159.hg.1 | NM_001130823; NM_001379; ENST00000340748; ENST00000359526; ENST00000540357; AB209413; AF290894; AK122759; AY927518; BC092517; BC126227; uc002mnf.3; uc002mng.3; uc002mnh.3; uc002mnk.3; uc010dxb.1; uc010xlc.2; uc010xld.2 | *DNMT1* | DNA (cytosine-5-)-methyltransferase 1 | 6.85 | 6.92 | -1.05 | 0.0458 |
| TC18000166.hg.1 | NM_138443; NR_026978; ENST00000282058; BC005958; BC014003; OTTHUMT00000255885; uc002lbu.3; uc002lbv.3 | *HAUS1* | HAUS augmin like complex subunit 1 | 3.64 | 3.48 | 1.12 | 0.0458 |
| TC08002336.hg.1 | AK055621; NR_038236 | *LINC00968* | long intergenic non-protein coding RNA 968 | 2.17 | 2.13 | 1.03 | 0.0459 |
| TC11002383.hg.1 | NM_001001786; ENST00000560104; BC130361; uc001pyf.3 | *BLID* | BH3-like motif containing, cell death inducer | 1.38 | 1.27 | 1.08 | 0.046 |
| TC14002071.hg.1 | AK123430 | *MAP3K9* | mitogen-activated protein kinase kinase kinase 9 | 2.94 | 3.12 | -1.13 | 0.046 |
| TC21000364.hg.1 | NM_001085455; ENST00000340345; OTTHUMT00000246806; uc002ynv.3 | *KRTAP24-1* | keratin associated protein 24-1 | 2.07 | 2.15 | -1.06 | 0.0461 |
| TC01003319.hg.1 | FM178012 | *POU5F1P4* | POU class 5 homeobox 1 pseudogene 4 | 6.11 | 6.07 | 1.03 | 0.0462 |
| TC11000644.hg.1 | NM_025128; ENST00000308110; ENST00000525006; ENST00000533035; AL353934; BC009999; uc001ofv.4; uc001ofx.4 | *MUS81* | MUS81 structure-specific endonuclease subunit | 5.87 | 5.93 | -1.04 | 0.0463 |
| TC11000645.hg.1 | NM_001335; ENST00000307886; ENST00000528419; BC048255; uc001ogc.1 | *CTSW* | cathepsin W | 8.01 | 8.41 | -1.32 | 0.0464 |
| TC0Y000094.hg.1 | NR_001555; NR_002195; AF332229; uc004fwv.3; uc011nbw.2 | *GOLGA2P2Y; GOLGA2P3Y* | golgin A2 pseudogene 2, Y-linked; golgin A2 pseudogene 3, Y-linked | 5.68 | 5.71 | -1.03 | 0.0464 |
| TC0Y000209.hg.1 | NR_001555; NR_002195; ENST00000398377; uc004fwd.3 | *GOLGA2P2Y; GOLGA2P3Y* | golgin A2 pseudogene 2, Y-linked; golgin A2 pseudogene 3, Y-linked | 5.68 | 5.71 | -1.03 | 0.0464 |
| TC14000751.hg.1 | NR_030408; uc021sdl.1 | *MIR668* | microRNA 668 | 4.72 | 4.89 | -1.13 | 0.0464 |
| TC19000800.hg.1 | NM_001099694; AB091373; AB091374; AB091375; AB091376; uc002pzp.4 | *ZNF578* | zinc finger protein 578 | 4.65 | 4.69 | -1.03 | 0.0464 |
| TC05001608.hg.1 | NM_001118890; NM_001243658; NM_001243659; NM_002064; ENST00000237858; ENST00000379979; ENST00000505427; ENST00000507605; ENST00000508780; ENST00000512469; BC005304; BC010965; BC106075; OTTHUMT00000241653; OTTHUMT00000370267; OTTHUMT00000370268; OTTHUMT00000370269; OTTHUMT00000370270; OTTHUMT00000370271; OTTHUMT00000370272; uc003kln.4; uc003klo.4; uc021ybn.1; uc021ybo.1 | *GLRX* | glutaredoxin | 6.06 | 5.94 | 1.09 | 0.0465 |
| TC02000852.hg.1 | ENST00000415552 | *RHOQP2* | ras homolog family member Q pseudogene 2 | 5.86 | 5.7 | 1.12 | 0.0465 |
| TC01002265.hg.1 | ENST00000383925 | *RNU1-1* | RNA, U1 small nuclear 1 | 10.58 | 10.3 | 1.21 | 0.0465 |
| TC01000225.hg.1 | ENST00000384278 | *RNU1-2* | RNA, U1 small nuclear 2 | 10.58 | 10.3 | 1.21 | 0.0465 |
| TC01002274.hg.1 | ENST00000384782 | *RNU1-3* | RNA, U1 small nuclear 3 | 10.58 | 10.3 | 1.21 | 0.0465 |
| TC01000218.hg.1 | ENST00000384659 | *RNU1-4* | RNA, U1 small nuclear 4 | 10.58 | 10.3 | 1.21 | 0.0465 |
| TC14000214.hg.1 | ENST00000383869 | *RNU1-27P* | RNA, U1 small nuclear 27, pseudogene | 10.58 | 10.3 | 1.21 | 0.0465 |
| TC14001027.hg.1 | ENST00000383861 | *RNU1-28P* | RNA, U1 small nuclear 28, pseudogene | 10.58 | 10.3 | 1.21 | 0.0465 |
| TC01003162.hg.1 | ENST00000384010 | *RNVU1-18* | RNA, variant U1 small nuclear 18 | 10.58 | 10.3 | 1.21 | 0.0465 |
| TC06001002.hg.1 | NM_001253676; NM_004865; ENST00000237264; ENST00000367871; ENST00000477527; BC000381; BC017559; OTTHUMT00000042294; OTTHUMT00000042295; OTTHUMT00000042296; OTTHUMT00000042297; OTTHUMT00000042298; OTTHUMT00000042299; OTTHUMT00000042300; uc003qel.3; uc010kgg.3 | *TBPL1* | TBP-like 1 | 5.64 | 5.55 | 1.06 | 0.0465 |
| TC13000292.hg.1 | NM_006493; ENST00000377453; ENST00000485938; OTTHUMT00000045318; OTTHUMT00000045319; uc001vkc.3 | *CLN5* | ceroid-lipofuscinosis, neuronal 5 | 4.69 | 4.64 | 1.04 | 0.0466 |
| TC02000447.hg.1 | NM_006463; NM_201647; NM_213622; ENST00000339566; ENST00000394070; ENST00000394073; ENST00000409707; ENST00000486458; ENST00000536064; BC007682; BC065574; BC101467; BC101469; OTTHUMT00000252048; OTTHUMT00000328094; OTTHUMT00000328095; OTTHUMT00000328096; OTTHUMT00000328097; OTTHUMT00000328098; OTTHUMT00000328101; OTTHUMT00000328103; uc002sjs.3; uc002sju.3; uc002sjv.3 | *STAMBP* | STAM binding protein | 5.62 | 5.55 | 1.05 | 0.0466 |
| TC09001486.hg.1 | NM_001080551; NM_173521; ENST00000318737; ENST00000374283; ENST00000374287; ENST00000394777; ENST00000394779; BC041858; BC112357; BC144680; OTTHUMT00000053656; OTTHUMT00000053657; OTTHUMT00000053658; OTTHUMT00000316570; uc004bfq.3; uc004bfr.3; uc004bfs.1; uc011lwt.2 | *C9orf84* | chromosome 9 open reading frame 84 | 2.24 | 2.1 | 1.11 | 0.0467 |
| TC03002771.hg.1 | NR_026786 | *LMLN* | leishmanolysin-like (metallopeptidase M8 family) | 4.03 | 4.13 | -1.07 | 0.0467 |
| TC17001556.hg.1 | NR_027412; NR_027413; AK123585; AK311685; uc010czf.3; uc010wib.1; uc010wid.2; uc010wie.2 | *LINC00910* | long intergenic non-protein coding RNA 910 | 4.98 | 4.92 | 1.05 | 0.0468 |
| TC20000135.hg.1 | NM_016100; NM_181527; NM_181528; ENST00000310450; ENST00000334982; ENST00000398602; ENST00000484480; BC005181; BC008446; OTTHUMT00000078217; OTTHUMT00000127782; OTTHUMT00000127783; OTTHUMT00000127784; OTTHUMT00000127785; OTTHUMT00000127786; OTTHUMT00000334102; uc002wrp.3; uc002wrq.3; uc002wrr.3 | *NAA20* | N(alpha)-acetyltransferase 20, NatB catalytic subunit | 4.9 | 4.97 | -1.04 | 0.0468 |
| TC02004563.hg.1 | BC005401 | *WDR33* | WD repeat domain 33 | 6.82 | 6.94 | -1.09 | 0.0468 |
| TC0X001201.hg.1 | NR_003539 | *BRDTP1* | bromodomain, testis-specific pseudogene 1 | 1.32 | 1.23 | 1.06 | 0.0469 |
| TC13001195.hg.1 | BC036310 | *LINC00382* | long intergenic non-protein coding RNA 382 | 1.64 | 1.62 | 1.02 | 0.0469 |
| TC14000146.hg.1 | NM_001037288; NM_138460; ENST00000339180; ENST00000342473; ENST00000359320; ENST00000382809; ENST00000397227; ENST00000555731; AF527413; AF527414; AF527948; AF527949; BC013109; uc001wjs.3; uc001wjt.3; uc001wju.3; uc010akm.3; uc010akn.3; uc010ako.3 | *CMTM5* | CKLF-like MARVEL transmembrane domain containing 5 | 5.65 | 5.4 | 1.19 | 0.047 |
| TC04001719.hg.1 | NM_001012967; ENST00000260184; ENST00000505890; ENST00000511577; ENST00000515088; AK096369; CR933601; OTTHUMT00000364839; OTTHUMT00000364840; OTTHUMT00000364841; OTTHUMT00000364842; OTTHUMT00000364843; OTTHUMT00000364844; OTTHUMT00000364845; OTTHUMT00000364846; OTTHUMT00000364847; OTTHUMT00000364848; OTTHUMT00000364849; OTTHUMT00000364850; OTTHUMT00000364851; OTTHUMT00000365134; OTTHUMT00000365135; uc003irq.4; uc003irr.1; uc003irt.1 | *DDX60L* | DEAD (Asp-Glu-Ala-Asp) box polypeptide 60-like | 7.06 | 6.86 | 1.14 | 0.047 |
| TC01001027.hg.1 | NR_030640; ENST00000401111; uc021osm.1 | *MIR942* | microRNA 942 | 3.36 | 3.52 | -1.12 | 0.047 |
| TC07000926.hg.1 | NM_001001667; ENST00000418316; BC137044; OTTHUMT00000350860; uc011ksv.2 | *OR6V1* | olfactory receptor, family 6, subfamily V, member 1 | 4.28 | 4.41 | -1.1 | 0.047 |
| TC03001158.hg.1 | NM_001134941; NM_001134944; NM_001134945; NM_001134946; NM_016362; NR_024137; NR_024138; ENST00000287656; ENST00000335542; ENST00000422159; ENST00000429122; ENST00000430179; ENST00000437422; ENST00000439975; ENST00000446937; ENST00000449238; ENST00000457360; ENST00000476283; AY184207; BC025791; EF139854; EF139856; EF549557; EF549563; NR_024132; NR_024133; NR_024134; NR_024135; NR_024136; OTTHUMT00000339625; OTTHUMT00000339626; OTTHUMT00000339627; OTTHUMT00000339628; OTTHUMT00000339629; OTTHUMT00000339630; OTTHUMT00000339632; OTTHUMT00000339633; OTTHUMT00000339636; OTTHUMT00000339637; OTTHUMT00000339638; OTTHUMT00000339639; OTTHUMT00000339640; OTTHUMT00000379272; OTTHUMT00000379273; uc003bvj.1; uc010hda.1; uc010hdb.1; uc010hdc.2; uc010hdd.2; uc010hdj.2; uc010hdk.2; uc021wsz.1 | *GHRL* | ghrelin/obestatin prepropeptide | 5.24 | 5.19 | 1.04 | 0.0471 |
| TC21000466.hg.1 | NR_039917; uc021wjk.1 | *MIR4760* | microRNA 4760 | 0.88 | 0.84 | 1.03 | 0.0471 |
| TC06004150.hg.1 | NM_001145775; NM_001145776; NM_001145777; NM_004117; ENST00000357266; ENST00000536438; ENST00000539068; ENST00000542713; AK302704; BC042605; BC111050; OTTHUMT00000040309; uc003okx.2; uc003oky.2; uc003okz.2; uc011dte.1; uc011dtf.1 | *FKBP5* | FK506 binding protein 5 | 7.22 | 6.91 | 1.24 | 0.0474 |
| TC07001537.hg.1 | NM_031925; BC029487; BC040694; BC051850; uc003ueb.1; uc003uec.2; uc003ued.3 | *TMEM120A* | transmembrane protein 120A | 6.24 | 6.16 | 1.06 | 0.0474 |
| TC19000247.hg.1 | NM_017721; ENST00000318003; AF536205; AK023399; AK123155; BC006556; BC064981; uc002mxn.2; uc002mxo.2; uc002mxp.2; uc002mxq.1; uc010dzh.2 | *CC2D1A* | coiled-coil and C2 domain containing 1A | 5.73 | 5.78 | -1.04 | 0.0475 |
| TC06000936.hg.1 | NR_037662; uc021zeg.1 | *LOC100287632* | selenoprotein K pseudogene | 1.49 | 1.36 | 1.09 | 0.0475 |
| TC17000836.hg.1 | NM_178160; ENST00000331427; AK000063; uc002jmf.1; uc010wrp.2 | *OTOP2* | otopetrin 2 | 4.32 | 4.4 | -1.06 | 0.0475 |
| TC15000325.hg.1 | NM_012142; NR_027513; NR_027514; NR_045998; NR_045999; ENST00000300213; ENST00000444658; AF113535; AK075146; AK290707; AK308817; BC009689; OTTHUMT00000253203; uc001zqv.3; uc001zqy.3; uc010bdb.3; uc010udl.2; uc021sjs.1; uc021sjt.1; uc021sju.1 | *CCNDBP1* | cyclin D-type binding-protein 1 | 6.78 | 6.58 | 1.15 | 0.0476 |
| TC19002657.hg.1 | NM_013289; ENST00000326542; ENST00000358178; ENST00000391728; AF262973; BC028206; BC101540; L76664; OTTHUMT00000141238; OTTHUMT00000141239; OTTHUMT00000156165; U33328; uc002qhk.4; uc010esf.3; uc021vbm.1; uc021vbo.1 | *KIR3DL1* | killer cell immunoglobulin-like receptor, three domains, long cytoplasmic tail, 1 | 5.26 | 5.65 | -1.31 | 0.0476 |
| TC12000668.hg.1 | NM_001145026; ENST00000547485; uc001sze.2 | *PTPRQ* | protein tyrosine phosphatase, receptor type, Q | 1.55 | 1.53 | 1.02 | 0.0476 |
| TC22001260.hg.1 | AJ867757; AJ867758 | *GSTT1* | glutathione S-transferase theta 1 | 4.92 | 5.3 | -1.31 | 0.0477 |
| TC01004818.hg.1 | BC037864; NR_037163 | *POU2F1* | POU class 2 homeobox 1 | 6.28 | 6.38 | -1.08 | 0.0477 |
| TC01003067.hg.1 | OTTHUMT00000193207 | *RP11-423O2.7* |  | 1.4 | 1.31 | 1.06 | 0.0477 |
| TC04000140.hg.1 | NM_001177381; NM_001177382; NM_001177383; NM_001177384; NM_182485; NM_182646; ENST00000259997; ENST00000345451; ENST00000382395; ENST00000382401; ENST00000442003; ENST00000507071; ENST00000538197; BC103939; BC103940; BC103941; BC103942; BC105925; OTTHUMT00000207349; OTTHUMT00000207350; OTTHUMT00000359343; OTTHUMT00000359344; OTTHUMT00000359345; OTTHUMT00000359346; OTTHUMT00000359347; uc003gni.2; uc003gnj.2; uc003gnk.2; uc003gnl.2; uc003gnm.2; uc003gnn.2 | *CPEB2* | cytoplasmic polyadenylation element binding protein 2 | 6.44 | 6.27 | 1.12 | 0.0478 |
| TC13001534.hg.1 | AK092024 | *DIAPH3* | diaphanous-related formin 3 | 3.18 | 2.87 | 1.24 | 0.0479 |
| TC07000516.hg.1 | ENST00000416443 | *HMGN2P11* | high mobility group nucleosomal binding domain 2 pseudogene 11 | 5.08 | 5.19 | -1.08 | 0.0479 |
| TC04001455.hg.1 | NM_005443; ENST00000265174; ENST00000511304; AK297981; BC011392; BC050627; OTTHUMT00000253946; OTTHUMT00000363703; OTTHUMT00000363704; OTTHUMT00000363705; OTTHUMT00000363706; OTTHUMT00000363707; OTTHUMT00000363708; OTTHUMT00000363709; uc003hyk.3; uc011cfh.1 | *PAPSS1* | 3-phosphoadenosine 5-phosphosulfate synthase 1 | 6.13 | 5.89 | 1.18 | 0.0479 |
| TC05000365.hg.1 | ENST00000504514; ENST00000506596; AK307154; OTTHUMT00000369147; OTTHUMT00000369148 | *ANKDD1B* | ankyrin repeat and death domain containing 1B | 2.48 | 2.42 | 1.04 | 0.048 |
| TC06000546.hg.1 | ENST00000373404 | *FLJ45825* | uncharacterized LOC100505530 | 4.33 | 3.91 | 1.33 | 0.048 |
| TC20000070.hg.1 | BC043288 | *LOC101929312* | uncharacterized LOC101929312 | 1.83 | 1.92 | -1.06 | 0.048 |
| TC06003566.hg.1 | AJ459855 | *OR11A1* | olfactory receptor, family 11, subfamily A, member 1 | 1.85 | 1.75 | 1.08 | 0.048 |
| TC01001326.hg.1 | NM_001142560; NM_015997; ENST00000368216; ENST00000368218; ENST00000476229; ENST00000481920; ENST00000524343; BC011382; OTTHUMT00000098973; OTTHUMT00000098974; OTTHUMT00000098975; OTTHUMT00000098976; OTTHUMT00000098977; OTTHUMT00000098978; OTTHUMT00000145386; OTTHUMT00000378735; OTTHUMT00000378736; OTTHUMT00000378840; OTTHUMT00000378862; uc001fpu.3; uc001fpv.3 | *RRNAD1* | ribosomal RNA adenine dimethylase domain containing 1 | 5.19 | 5.12 | 1.05 | 0.048 |
| TC07000993.hg.1 | NM_001099220; ENST00000223210; ENST00000478024; BC045757; OTTHUMT00000350165; OTTHUMT00000350166; OTTHUMT00000350167; OTTHUMT00000350168; OTTHUMT00000350169; OTTHUMT00000350170; uc010lpn.3 | *ZNF862* | zinc finger protein 862 | 5.93 | 5.86 | 1.05 | 0.048 |
| TC04000209.hg.1 | NM_001136536; NM_001170700; ENST00000357504; ENST00000456874; ENST00000503528; ENST00000507598; AK094684; BC157885; OTTHUMT00000360377; OTTHUMT00000360378; OTTHUMT00000360379; OTTHUMT00000360380 | *DTHD1* | death domain containing 1 | 4.41 | 4.64 | -1.18 | 0.0482 |
| TC01003880.hg.1 | ENST00000436706 | *LOC101927143* | uncharacterized LOC101927143 | 4.13 | 4.21 | -1.06 | 0.0482 |
| TC03001590.hg.1 | NM_000313; ENST00000394236; ENST00000407433; BC015801; OTTHUMT00000317762; OTTHUMT00000317763; OTTHUMT00000317764; OTTHUMT00000352896; OTTHUMT00000352897; uc003dqz.4; uc003drb.4; X12892 | *PROS1* | protein S (alpha) | 2.78 | 2.66 | 1.09 | 0.0482 |
| TC06000876.hg.1 | NM_003080; ENST00000258052; AK310148; BC000038; OTTHUMT00000041755; OTTHUMT00000041756; OTTHUMT00000041757; uc003pti.3; uc010kdq.3 | *SMPD2* | sphingomyelin phosphodiesterase 2, neutral membrane (neutral sphingomyelinase) | 5.83 | 5.78 | 1.04 | 0.0482 |
| TC01002370.hg.1 | NM_001031680; NM_004350; ENST00000308873; ENST00000338888; ENST00000399916; ENST00000496967; AF022726; AK301044; AK310945; BC013362; OTTHUMT00000009284; OTTHUMT00000009285; OTTHUMT00000009286; OTTHUMT00000009287; uc001bjq.3; uc001bjr.3; uc009vrj.3; uc009vrk.3; uc009vrl.1; uc010oen.2 | *RUNX3* | runt-related transcription factor 3 | 7.29 | 7.34 | -1.04 | 0.0483 |
| TC20000839.hg.1 | NR_002911; ENST00000364729; uc002xim.1 | *SNORA71A* | small nucleolar RNA, H/ACA box 71A | 8.12 | 7.68 | 1.36 | 0.0484 |
| TC17000387.hg.1 | NM_005408; ENST00000225844; BC008621; OTTHUMT00000256389; uc002hic.3 | *CCL13* | chemokine (C-C motif) ligand 13 | 3.41 | 3.48 | -1.05 | 0.0486 |
| TC19001775.hg.1 | NM_152353; ENST00000291715; BC029518; uc002pwi.1 | *CLDND2* | claudin domain containing 2 | 5.8 | 5.88 | -1.06 | 0.0486 |
| TC19002658.hg.1 | NM_001242867; NM_006737; ENST00000270442; ENST00000326321; BC105678; OTTHUMT00000141241; OTTHUMT00000141242; uc002qho.4; uc010esh.3 | *KIR3DL2* | killer cell immunoglobulin-like receptor, three domains, long cytoplasmic tail, 2 | 5.63 | 6.04 | -1.33 | 0.0486 |
| TC08000505.hg.1 | NM_006823; NM_181839; ENST00000352966; ENST00000396418; ENST00000518467; AK311857; BC022265; OTTHUMT00000379420; OTTHUMT00000379421; OTTHUMT00000379422; uc003yba.3; uc003ybb.3; uc010lzo.3 | *PKIA* | protein kinase (cAMP-dependent, catalytic) inhibitor alpha | 4.4 | 4.34 | 1.04 | 0.0486 |
| TC05000591.hg.1 | OTTHUMT00000372357 | *RP11-436H11.4* |  | 1.77 | 1.6 | 1.13 | 0.0487 |
| TC17001391.hg.1 | NM_001001418; NM_001123392; AK122833; AK302064; BC047739; BC071680; BC075809; uc002hlk.2; uc002hll.1; uc002hlm.2; uc002hmb.2; uc002hmc.3; uc002hmd.3; uc010wcv.1; uc010wcx.1; uc021tvh.1 | *TBC1D3C; TBC1D3H; TBC1D3L; TBC1D3; TBC1D3E; TBC1D3G; TBC1D3F* | TBC1 domain family, member 3C; TBC1 domain family, member 3H; TBC1 domain family, member 3L; TBC1 domain family, member 3; TBC1 domain family, member 3E; TBC1 domain family, member 3G; TBC1 domain family, member 3F | 6.23 | 6.13 | 1.07 | 0.0487 |
| TC16000456.hg.1 | NM_032330; ENST00000457326; BC005397; BC006000; uc002eid.1 | *CAPNS2* | calpain, small subunit 2 | 3.76 | 3.49 | 1.2 | 0.0488 |
| TC09001726.hg.1 | NM_181701; ENST00000358701; OTTHUMT00000055046; OTTHUMT00000055047; uc010nbi.2 | *QSOX2* | quiescin Q6 sulfhydryl oxidase 2 | 5.3 | 5.15 | 1.11 | 0.0488 |
| TC12002026.hg.1 | ENST00000538405 | *LOC105370024* | uncharacterized LOC105370024 | 4.33 | 4.41 | -1.06 | 0.0489 |
| TC06002103.hg.1 | NM_001017373; NM_152552; ENST00000324172; ENST00000368134; ENST00000437477; ENST00000439090; ENST00000457563; ENST00000532763; ENST00000533296; AK310075; BC127654; OTTHUMT00000042197; OTTHUMT00000042198; OTTHUMT00000042199; OTTHUMT00000042200; uc003qbz.1 | *SAMD3* | sterile alpha motif domain containing 3 | 5.08 | 5.39 | -1.24 | 0.0489 |
| TC04002537.hg.1 | BC036474 | *GABRA4* | gamma-aminobutyric acid (GABA) A receptor, alpha 4 | 1.21 | 1.12 | 1.06 | 0.0491 |
| TC04001995.hg.1 | BC014384 | *TMEM33* | transmembrane protein 33 | 5.92 | 5.64 | 1.21 | 0.0491 |
| TC10002490.hg.1 | AK055012 | *SLC39A12-AS1* | SLC39A12 antisense RNA 1 | 1.32 | 1.27 | 1.03 | 0.0492 |
| TC0X001753.hg.1 | U23863 | *MED12* | mediator complex subunit 12 | 7.91 | 7.97 | -1.04 | 0.0493 |
| TC01006105.hg.1 | NR_037850 | *PROX1-AS1* | PROX1 antisense RNA 1 | 2.35 | 2.42 | -1.05 | 0.0493 |
| TC11002894.hg.1 | M83106 | *TAGLN* | transgelin | 5.16 | 5.29 | -1.09 | 0.0493 |
| TC12001880.hg.1 | NM_024312; ENST00000299314; ENST00000392919; ENST00000549165; ENST00000549940; AL832172; BC071687; BC094884; BC131787; uc001tit.3; uc001tiu.2; uc001tiv.4; uc001tiw.3 | *GNPTAB* | N-acetylglucosamine-1-phosphate transferase, alpha and beta subunits | 6.2 | 6.37 | -1.12 | 0.0494 |
| TC17000783.hg.1 | NM_002737; ENST00000284384; ENST00000413366; AB209475; BC109273; BC109274; uc002jfo.1; uc002jfp.1 | *PRKCA* | protein kinase C, alpha | 6.59 | 6.44 | 1.11 | 0.0494 |
| TC0X001251.hg.1 | NM_001012755; ENST00000467290; BC140812; BC140814; OTTHUMT00000057761; OTTHUMT00000057762; uc004elu.3 | *SLC25A53* | solute carrier family 25, member 53 | 4.74 | 4.81 | -1.05 | 0.0494 |
| TC20000458.hg.1 | NM_001195677; NM_004738; NR_036633; ENST00000265619; ENST00000395802; ENST00000475243; AK127252; BC001712; OTTHUMT00000079875; OTTHUMT00000267817; OTTHUMT00000267818; OTTHUMT00000267819; OTTHUMT00000267820; OTTHUMT00000381405; uc002xza.3; uc002xzb.3; uc002xzd.2; uc010zzo.2 | *VAPB* | VAMP (vesicle-associated membrane protein)-associated protein B and C | 6.18 | 6.07 | 1.08 | 0.0494 |
| TC07000723.hg.1 | NM_006136; ENST00000361183; ENST00000466023; ENST00000490693; AK294409; BC005338; DQ655956; OTTHUMT00000059506; OTTHUMT00000141323; OTTHUMT00000141324; OTTHUMT00000141325; OTTHUMT00000141326; OTTHUMT00000141327; OTTHUMT00000141328; OTTHUMT00000268238; OTTHUMT00000268239; OTTHUMT00000315776; OTTHUMT00000316600; OTTHUMT00000316601; OTTHUMT00000316732; uc003vik.1; uc003vil.3; uc011knk.2 | *CAPZA2* | capping protein (actin filament) muscle Z-line, alpha 2 | 6.37 | 6.27 | 1.07 | 0.0495 |
| TC06001422.hg.1 | NM_021066; ENST00000333151; BC133048; BC133050; OTTHUMT00000040154; uc003njn.1 | *HIST1H2AJ* | histone cluster 1, H2aj | 1.64 | 1.52 | 1.08 | 0.0495 |
| TC19002559.hg.1 | S69115 | *NKG7* | natural killer cell granule protein 7 | 8.19 | 8.58 | -1.31 | 0.0495 |
| TC02001477.hg.1 | NM_001008491; NM_001008492; NM_004404; NM_006155; ENST00000360051; ENST00000391971; ENST00000391973; ENST00000401990; ENST00000402092; ENST00000407971; ENST00000461048; AK294563; BC014455; BC033559; BC040676; OTTHUMT00000323176; OTTHUMT00000323177; OTTHUMT00000323184; OTTHUMT00000323185; OTTHUMT00000323186; OTTHUMT00000323187; OTTHUMT00000323188; OTTHUMT00000323199; OTTHUMT00000323200; OTTHUMT00000323202; OTTHUMT00000323203; OTTHUMT00000323204; OTTHUMT00000323206; OTTHUMT00000323207; OTTHUMT00000323208; OTTHUMT00000323209; OTTHUMT00000323210; OTTHUMT00000323211; OTTHUMT00000323212; OTTHUMT00000323213; OTTHUMT00000323214; OTTHUMT00000323215; OTTHUMT00000323216; OTTHUMT00000323217; OTTHUMT00000323218; OTTHUMT00000323219; OTTHUMT00000323220; OTTHUMT00000323221; OTTHUMT00000323222; OTTHUMT00000323223; OTTHUMT00000323224; OTTHUMT00000323225; OTTHUMT00000323226; OTTHUMT00000323227; OTTHUMT00000323228; OTTHUMT00000323229; OTTHUMT00000323230; OTTHUMT00000323231; OTTHUMT00000323232; OTTHUMT00000323233; OTTHUMT00000323234; OTTHUMT00000323235; OTTHUMT00000323236; OTTHUMT00000323237; uc002wbc.3; uc002wbd.3; uc002wbe.1; uc002wbf.3; uc002wbg.3; uc002wbh.3; uc010zop.2 | *Sep-02* | septin 2 | 6.6 | 6.64 | -1.02 | 0.0495 |
| TC6_qbl_hap6000022.hg.1 | NM_014596; NM_170783; BC010898; BC050608; OTTHUMT00000035766; OTTHUMT00000035767; OTTHUMT00000035768; OTTHUMT00000035769; OTTHUMT00000253194; OTTHUMT00000253195; uc011ihi.2; uc011ihj.2 | *ZNRD1* | zinc ribbon domain containing 1 | 4.78 | 4.72 | 1.05 | 0.0495 |
| TC21000361.hg.1 | NM_012131; ENST00000286808; BC101503; BC101505; OTTHUMT00000182261; uc011acv.2 | *CLDN17* | claudin 17 | 1.59 | 1.46 | 1.09 | 0.0496 |
| TC01003209.hg.1 | NM_001199829; NM_032132; ENST00000322343; ENST00000361824; ENST00000368995; ENST00000476530; BC033014; BC047406; OTTHUMT00000084722; OTTHUMT00000084723; OTTHUMT00000084724; OTTHUMT00000084725; OTTHUMT00000084726; OTTHUMT00000084727; OTTHUMT00000084728; OTTHUMT00000084729; uc001evk.2; uc001evl.2; uc001evm.2 | *HORMAD1* | HORMA domain containing 1 | 2.94 | 2.77 | 1.13 | 0.0496 |
| TC13000162.hg.1 | NR_026955; ENST00000423211; ENST00000439707; uc001uzg.3 | *LINC00284* | long intergenic non-protein coding RNA 284 | 2.5 | 2.62 | -1.08 | 0.0496 |
| TC0X001414.hg.1 | NM_001173516; NM_001173517; NM_024597; ENST00000316077; ENST00000370661; ENST00000370663; ENST00000495432; BC064350; OTTHUMT00000058487; OTTHUMT00000058488; OTTHUMT00000058489; OTTHUMT00000058490; OTTHUMT00000313965; OTTHUMT00000313966; OTTHUMT00000313967; uc004ezs.3; uc004ezt.3; uc010nsa.2; uc011mwc.2 | *MAP7D3* | MAP7 domain containing 3 | 4.81 | 4.86 | -1.03 | 0.0497 |
| TC0X001436.hg.1 | NR_031725; ENST00000408154; uc022cfg.1 | *MIR320D2* | microRNA 320d-2 | 2.68 | 2.77 | -1.06 | 0.0497 |
| TC19001985.hg.1 | NR_028594 | *ZNF833P* | zinc finger protein 833, pseudogene | 3.94 | 4.06 | -1.09 | 0.0497 |
| TC19001686.hg.1 | NM_017708; ENST00000263266; BC111970; uc002pjn.2 | *FAM83E* | family with sequence similarity 83, member E | 5.74 | 5.84 | -1.07 | 0.0498 |
| TC15001350.hg.1 | NM_002041; NM_005254; NM_016654; NM_016655; NM_181427; ENST00000220429; ENST00000359031; ENST00000380877; ENST00000396464; ENST00000429662; ENST00000543881; ENST00000560825; AK303901; BC016910; BC036080; BC050702; D13316; OTTHUMT00000254537; OTTHUMT00000254538; uc001zya.3; uc001zyb.3; uc001zyc.3; uc001zyd.3; uc001zye.3; uc001zyf.3; uc010ufg.2 | *GABPB1* | GA binding protein transcription factor, beta subunit 1 | 5.73 | 5.62 | 1.08 | 0.0498 |
| TC09002588.hg.1 | BC043510 | *MAMDC2-AS1* | MAMDC2 antisense RNA 1 | 2.77 | 2.69 | 1.06 | 0.0498 |
| TC02004131.hg.1 | uc002rkn.1 | *FTH1P3* | ferritin, heavy polypeptide 1 pseudogene 3 | 8.3 | 8.15 | 1.11 | 0.0499 |

**Note:** The FDR adjusted *p*-value was 0.9999 for all the genes.
